# On the Importance of the Distance Measures Used to Train and Test Knowledge-Based Potentials for Proteins

**DOI:** 10.1371/journal.pone.0109335

**Published:** 2014-11-20

**Authors:** Martin Carlsen, Patrice Koehl, Peter Røgen

**Affiliations:** 1 Department of Applied Mathematics and Computer Science, Technical University of Denmark, Kongens Lyngby, Denmark; 2 Department of Computer Science and Genome Center, University of California Davis, Davis, CA, United States of America; University of Michigan, United States of America

## Abstract

Knowledge-based potentials are energy functions derived from the analysis of databases of protein structures and sequences. They can be divided into two classes. Potentials from the first class are based on a direct conversion of the distributions of some geometric properties observed in native protein structures into energy values, while potentials from the second class are trained to mimic quantitatively the geometric differences between incorrectly folded models and native structures. In this paper, we focus on the relationship between energy and geometry when training the second class of knowledge-based potentials. We assume that the difference in energy between a decoy structure and the corresponding native structure is linearly related to the distance between the two structures. We trained two distance-based knowledge-based potentials accordingly, one based on all inter-residue distances (PPD), while the other had the set of all distances filtered to reflect consistency in an ensemble of decoys (PPE). We tested four types of metric to characterize the distance between the decoy and the native structure, two based on extrinsic geometry (RMSD and GTD-TS*), and two based on intrinsic geometry (Q* and MT). The corresponding eight potentials were tested on a large collection of decoy sets. We found that it is usually better to train a potential using an intrinsic distance measure. We also found that PPE outperforms PPD, emphasizing the benefits of capturing consistent information in an ensemble. The relevance of these results for the design of knowledge-based potentials is discussed.

## Introduction

Proteins are the essential macromolecules inside cells that perform nearly all cellular functions. Just like macroscopic tools, their shapes is a key feature for defining their functions. Structural biologists have embarked upon the challenge of finding the structures of all proteins, in hopes of unraveling this relationship between geometry and biological activity and learn in the process how cells function. Determining experimentally the structure of a protein at the atomic level however is not yet an easy task: this can be indirectly deduced from the fact that we currently know millions of protein sequences but less than hundred thousand protein structures. Predicting the structure of a protein from first principles is not much easier: direct applications of the ideas that have been used for modeling small molecules have not yet been successful on these much larger molecules. Recent reports on the advancements of *ab initio* techniques clearly show that the protein structure prediction community is making progress, but that the quality of the models they generate do not meet yet the stringent accuracy requirements to become useful to the biologists [1]. Interestingly, the series of Critical Assessment of protein Structure Prediction (CASP) meetings have highlighted that while the methods for generating models of protein structures have improved significantly [2], identifying the native-like conformations among the large collections of model structures (also called decoys) remains a significant challenge [3], [4]. In this paper we focus on this problem.

Anfinsen's thermodynamics hypothesis states that the native structure of a protein is determined only by its amino acid sequence [5]. Structural and computational biologists translate this postulate into the statement, that under physiological conditions, the native state of a protein is a unique, stable minimum of the free energy. The key to solving the protein structure prediction problem amounts therefore to finding an accurate representation of this free energy function and several methods have been proposed to construct reasonable approximations of it. The two most common approaches rely on semiempirical and statistical potentials, respectively. Semiempirical methods are derived from knowledge of the basic physical principles whereas statistical potentials are based on the nonrandom statistics of known protein structures [6]. Statistical energy functions are either residue based or atom based and the most recent statistical potentials include pairwise interactions, orientations of side-chains [7], secondary structural preferences, solvent-exposure, and other geometric properties of proteins [8]. We note that there have been attempts to combine physics-based and statistics-based potentials to improve protein structure refinement [9]–[13].

Current protein structure prediction methods require potentials that ideally should assign “scores” to a protein structure model such that the higher the score, the less native-like the model is, where native-like is measured in terms of a distance *d* from the model to the native structure. If this condition is satisfied then the potential is expected to detect near native conformations even when the native conformation is not present; in addition, such an ideal potential could then be used for model refinement. In mathematical terms this can be expressed as the score function *f* satisfying
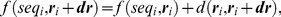
(1)


for any sequence *seq_i_* and all deformations ***dr*** of its native structure ***r*** *_i_*.

Several methods have been developed to optimize potentials towards this goal [14]–[17]. The choice of the distance measure *d* is critical to the success of these methods. The standard distance measure when comparing protein structural models is RMSD, i.e. the root mean square distance between the two models after optimal translation and rotation. RMSD however has been replaced in recent CASP experiments by the global distance test (GDT-TS [18]) due to its undesirable sensitivity towards local changes in a protein structure; GDT-TS has become one of the most commonly used distance measures in protein structure prediction. A less commonly used distance measure is the fraction of known native contacts, Q. Q quantifies the changes in the number of “contacts” found in the native structure compared to the model structure that is evaluated, where a contact corresponds to two residues being within a given threshold distance from each other. All the distance measures mentioned above identify geometric differences between two structural models but do not attempt to assess if these differences could be assigned to fluctuations due to the dynamics of the protein. Such differences would be less of a concern if they were related to geometric differences that can be explained by dynamics. As an attempt to identify the role of dynamics, Perez *et al.* recently introduced FlexE, a method based on a simple elastic network model that uses the deformation energy as a measure of the similarity between two structures [19]. As such, FlexE is expected to distinguish biologically relevant conformational changes from random changes.

In this work, we investigate the importance of the distance function *d* when optimizing an energy function *f* towards satisfying equation 1. We train two new 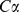-based pairwise potentials, PPD and PPE, to mimic the distance between the model structure considered and its corresponding native structure, using four different definitions of the distance measure, namely RMSD, GDT-TS, Q, and MT, where MT is an anharmonic version of FlexE. These energy functions are trained and tested on sets extracted from the high resolution decoy dataset Titan-HRD [20], as well as on well known decoy datasets from DecoysRUs [21] and Rosetta [22]. We have also analyzed the performance of our potentials on the server generated Stage_1 and Stage_2 decoy sets from CASP 10 [48].

The paper is organized as follows. The next section introduces the different distance measures and describes our procedures for training and testing the potentials PPD and PPE. The following section shows the results on different decoy sets as well as a comparison between PPD, PPE, two statistical knowledge-based potentials and a semi-empirical physical potential. We conclude with a discussion of the importance of the choice of the distance measure and describe potential future work.

## Materials and Methods

### Geometrical distances between two structural models of the same protein

Let us consider two structural models *A* and *B* of the same protein *P* with *N* amino acids. We represent the two models as discrete sets of *N* points, 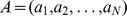 and 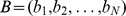 where the points 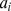 and 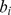 correspond to the positions of the 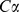 atoms *i* in the two structures. We assume that the correspondence table between *A* and *B* is known and set such that 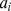 corresponds to 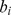 for all 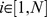. We measure the distance between the two models either based on the Euclidean distance between the two sets of points (RMSD and GDT-TS), on differences between contact maps within each set (Q), or on an elastic network (MT).

RMSD, i.e. root mean square deviation, is the Euclidean distance between the corresponding points 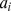 and 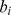 after one of the two sets of points (usually set *B*) has been optimally transformed by a rigid body transformation *G*:
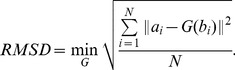
(2)


The rigid body transformation *G* is a transformation that does not produce changes in the size, shape, or topology of the protein. Such transformations are compositions of rotations and translations. Many closed-form solutions to the problem of finding the optimal *G* have been derived [23]–[25]. We note that RMSD as defined above is a metric [26].

RMSD is a distance measure based on the *L*_2_ norm; as such, it is highly sensitive to outliers, for example due to the presence of large albeit local differences between the two structures. The global distance test (GDT) was developed to decrease this sensitivity [18]. GDT focuses on the regions of the structures that can be correctly aligned by counting the number of residues that can be superimposed within a given cutoff distance. GDT-TS (where TS stands for Total Score), combines this information for multiple cutoffs:
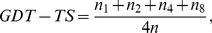
(3)


where *n*_1_, *n*_2_, *n*_4_, and *n*_8_ are the numbers of aligned residues within 1, 2, 4, and 8 Ångströms, respectively, and *n* is the total aligned length. Note that GDT-TS is a quantity between 0 and 1 that represents similarity, with low values corresponding to bad correspondences, and high values (close to or equal to 1) indicating that the two models are highly similar. We have converted this similarity measure into a distance by considering GDT-TS*  =  1-GDT-TS.

RMSD and GDT-TS* are computed after the two model structures have been optimally superposed. An alternative approach is to consider the intrinsic geometry of the two structures, as captured for example by a distance matrix that contains all 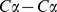 distances internal to one structure. Q and MT are two examples of distance measures that use this alternate approach.

The fraction of native contacts, Q, is a distance measure that quantifies the changes of a contact map between two models for the same structure. A contact map is usually defined as
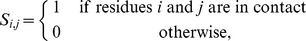



where two residues are in contact if they are within a given distance threshold. In this paper, we set this threshold to 9 Å. Q is then defined by
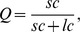



where *sc* is the number of shared contacts and *lc* is the number of lost contacts. Just like GDT-TS, Q is a measure of similarity. We convert it into a distance measure by defining Q* = 1-Q.

Q* quantifies changes in the contact map of a structure with no consideration of what could have been the reasons for these changes. FlexE is a new measure of similarity between protein structures that was introduced as an attempt to distinguish those changes that are biologically relevant [19]. It is based on the concept of elastic network that assigns virtual isotropic springs between pairs of residues. Elastic network models are used in normal mode analysis [27], [28] for example to reconstruct proteins [29], to generate decoy sets [30], or to investigate thermal fluctuations about the native or equilibrium structure [31], [32]. In the formalism introduced by Perez et al [19], the distance measure FlexE between two structures *N* and *D* is assimilated to the energetic cost of deforming one of the structures into the other:
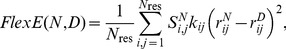
(4)


where 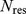 is the number of residues in *N* and *D*, 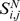 is a contact map for structure *N*, 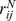 and 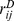 are the distances between the 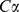 atoms of residues *i* and *j* in structures *N* and 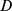, respectively, and *k_ij_* is a force constant associated to the link between *i* and *j*. In our implementation of FlexE, we set all force constants to 1. We modify the quadratic term in equation 4 with a term congruent to the potential introduced by Toda [33] to study chains of particles interacting with non-linear forces.

The corresponding variant of FlexE, which we name MT, is defined as:
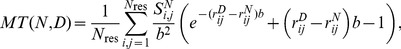
(5)


where *b* is a parameter which we set to 0.5. We note that MT is equal to FlexE for small perturbations of the distances between residues; for large perturbations however, it penalizes compression more than extension. Finally the use of the fixed native contact map for all native-decoy comparisons ensures that both FlexE(N,D) and MT(N,D) are well-defined.

### Two new parametric potentials

#### A smooth, pairwise potential, PPD

We design a smooth knowledge based residue pair potential as done in [34]. For each of the 210 pairs of amino acids types we assume a potential that is determined by the corresponding C*α*-C*α* distance. We model the interaction as a uniform cubic b-spline with compact support within 1 Å to 12 Å and 8 degrees of freedom, see e.g. [35]. With this model an interaction tends smoothly to zero energy at distances greater than 12 Å and is modeled freely within 4 Å–9 Å. The pair potential has 8×210 = 1680 parameters in total. The corresponding potential, PPD, is defined as
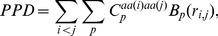
(6)


where 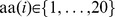 is the amino acid type of the i-th residue and 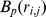 is the p-th b-spline basis function evaluated on the distance between the i-th and j-th residues. 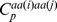 are the model parameters determined by the optimization procedure described below.

#### A consensus potential, PPE

We introduce a novel smooth ensemble based pair potential (PPE) that forms an artificial funnel relative to a pre-calculated contact map:
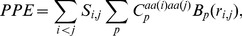
(7)


where *S_i,j_* is an consensus contact map. The method to calculate the consensus contact map is described below. It is based on a similar consensus method that constructs the reference contact map from an ensemble of decoys [36].

#### A consensus contact map

We introduce an iterative method to compute a consensus contact map of an ensemble of decoys. The first step is to construct a contact map from the most common contacts in the ensemble. Let *M_i,j_* be the fraction of contacts in the ensemble for the *i,j* -th residue pair. The contact map is then calculated as
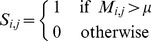
(8)


where *μ* is a cut-off fixed at 0.25. At each step, we select the 25% closest decoys to this contact map, where “closest” refers to the Hamming-distance to the contact map. This leads to a reduced ensemble from which a new contact map is computed, and the procedure is iterated. The algorithm usually converges in a few steps.

### Optimizing the potentials

We design an energy landscape using a sculpting procedure. We assume that we possess a set of natives structures 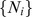 and that a set 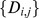 of decoy structures is known for each of these native structures. Let 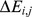 be the energy difference between the i-th native structure, 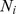, and its j-th decoy, 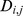, and let 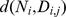 be the corresponding distance between 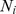 and 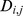. Our method for optimizing a statistical potential [34] attempts to establish a funnel-shaped energy function by calculating the parameters that minimizes the sum of squared errors between 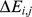 and 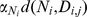 where 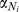 is a constant of proportionality. The problem can be stated as a quadratic programming (QP) problem with affine constraints,
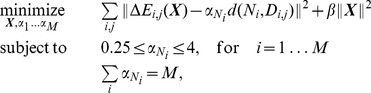
(9)


where *β* is a fixed parameter used for regularization. The variables in this QP problem are ***X***, i.e. the vector of coefficients 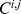 introduced above, and the constants of proportionality 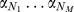, where *M* is the number of proteins in the training set. The last term 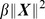 is a regularization term that adds a penalty onto the modulus of ***X***. The preprocessing is trivially parallelizable since each of the terms, 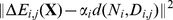, can be calculated individually. As a consequence, the QP requires little memory and is fast to compute. We use the optimization package cplex to solve it.

### Training and test sets

It is a nontrivial task to construct a “good” set of decoy structures. Any such decoy set relies on a sampling of the conformational space accessible to the protein structure of interest. The specific techniques used to generate such sampling are prone to biases [37], leading to poor sampling of the corresponding free energy surfaces. These approximate energy surfaces may not adopt a funnel like geometry in the neighborhood of the native structure and may contain many artificial potential energy barriers. To avoid the risk of learning from a specific bias introduced by one sampling technique, we have considered a variety of test sets to train and measure the performances of our energy functions. Of particular interest to us are near-native test sets since we design energy functions to mimic the neighborhoods of native structures.

We have chosen part of the Titan High Resolution Decoy set [20] as our training set. The list of proteins included in this set was originally proposed by Zhou and Skolnik [17]; it was selected on the basis that it is composed of a representative set of nonhomologous single domain proteins with maximum pairwise sequence similarity reported to be 35%. The models included in the decoy sets were generated using the torsion angle dynamics program DYANA [38] subject to distance constraints that are set to preserve the hydrophobic core of a protein. It is assumed that the hydrophobic core includes all residues within a *β* strand as well as all hydrophobic residues within an *α*-helix. The set includes 1400 proteins in total (compared to 1489 proteins in the original set of Zhou and Skolnik [17]). We eliminated all short proteins with a large radius of gyration as these proteins are overfitted by the optimization and are usually separate stretched secondary structures. We divided the remaining proteins into a training set of 1155 proteins with an average of 994 decoys per native structure (Titan-HRD*) and a test set of 142 proteins with an average of 854 decoys per native structure (Titan-HRD). The average GDT-TS distances between native and decoys over the training and test sets are 0.75 and 0.76 with a mean absolute deviation of 0.1, respectively. Note that we will use the mean absolute deviation (the *l*_1_-norm) instead of the standard deviation (the *l*_2_-norm) as it puts less weight on outliers.

Apart from the Titan-HRD set we use 10 freely available decoy sets that were generated using different procedures. These include 6 sets taken from DecoysRUs [21] (4 state reduced [39], hg structal [21], fisa [40], fisa casp3 [40], lmds [41] and lattice ssfit [42], [43]). We also included two older versions of the Rosetta decoy sets (Rosetta-All [44], Rosetta-Tsai [22]), the newest version Rosetta-Baker available at http://depts.washington.edu/bakerpg/decoys/ and the I-Tasser Set II [45].

The different CASP meetings have highlighted successes and failures in generating model structures that resemble the native structures of proteins. A repository of all models that have been proposed as answers to the prediction challenges that were part of these meetings is available on the CASP web page (http://predictioncenter.org). This repository provides a wealth of information on protein structure modeling, as well as useful test cases to assess the quality of new potential energy functions. We have therefore considered five CASP sets each containing models predicted by a variety of methods from the different CASP meetings (302 ensembles in total). We also generated CASP-HRD, a high resolution decoy subset of CASP 5–9, which includes models that have a TM score [46] larger than 0.5 and a RMSD less than 4 Å to the native structures. This cutoff was chosen based on the observation made by Xu and Zhang, which states that two decoys belong to the same fold when their TM-score to a native structure is higher than 0.5 [47]. CASP-HRD is constructed to have nearly the same average distance measure value as Titan-HRD but we find smaller variations of the distance measures for CASP-HRD. In that sense, it does include variations with different structural characteristics compared to Titan-HRD as it is generated by many different methods, while Titan-HRD is more homogeneous.

The total number of ensembles excluding Titan-HRD, Titan-HRD*, and CASP-HRD is 546 with an average GDT-TS between its decoys and their corresponding native structures of 0.47 with a average mean absolute deviation of 0.16. We refer to this set as “Test Set All” (TSA).

Finally, we include decoys from the latest CASP experiment, CASP10. A critical component of the CASP experiment is the assessment of the predictions that are submitted as putative models for the target proteins considered. This assessment is performed by the CASP assessors but also by the CASP community, with considerable enthusiasm, as observed in CASP10 [48]. The procedure for assessing the predictions in CASP10 differed from that of previous CASPs. The main difference was the introduction of two stages, labeled Stage_1 and Stage_2. For the former, twenty of the supposedly best predictions for each CASP target were released for assessment. Subsequently, hundred and fifty decoys were released for each target, defining Stage_2. Stage_1 ensembles are designed to survey single model assessment methods, while stage_2 allows for the survey of methods that rely on ensembles for the assessment of models. We have considered 93 targets from CASP10 for which both Stage_1 and Stage_2 test sets are available from the CASP web site (http://www.predictioncenter.org/casp10/). Compared to the other decoy sets described above, these sets contain longer protein chains. The models they include are usually as distant from their native counterparts as observed for the datasets from the previous CASP meetings. These sets however are more compact, i.e. with less diversity in distances, especially for the Stage_2 sets that resemble the CASP-HRD sets in that respect.

In table 1, we report the mean characteristics of these decoy sets (size, diversity, …) as well as information about their availability.

**Table 1:** Properties of the different protein decoy sets used in this study.

Decoy set	Nprot ^h^	Nres ^h^	Ndecoys ^h^	RMSD	MT	GDT-TS	Q
Titan-HRD a	142	127 (35)	854 (119)	2.4 (0.5)	2.7 (1)	0.76 (0.1)	0.85 (0.04)
Titan-HRD^*^ a	1155	111 (35)	994 (138)	2.6 (0.6)	2.7 (1)	0.75 (0.1)	0.85 (0.04)
TASSER Set II b	55	80 (17)	438 (98)	6.3 (1.5)	9.3 (3.2)	0.54 (0.05)	0.77 (0.03)
hg Structal c	28	150 (7)	29 (0)	4.1 (1.2)	4.4 (1.5)	0.71 (0.07)	0.85 (0.04)
4-state c	7	64 (4.9)	664 (15)	5.2 (1.4))	8.3 (2.9)	0.53 (0.11)	0.75 (0.05)
fisa c	4	60 (10)	500 (0.4)	7.5 (1.8)	8.6 (1.7)	0.47 (0.06)	0.75 (0.06)
fisa CASP3 c	5	88 (15)	1437 (390)	12 (1.6)	21 (4.1)	0.3 (0.03)	0.67 (0.02)
lmds c	10	53 (10)	433 (79)	7.7 (1.1)	12 (2.6)	0.46 (0.04)	0.72 (0.03)
lattice ssfit c	8	71 (10)	1997 (1.5)	9.9 (1.0)	17 (2.4)	0.3 (0.03)	0.64 (0.02)
Rosetta-All d	41	82 (25))	999 (0.5)	12 (1.4)	29 (5.6)	0.27 (0.03)	0.61 (0.02)
Rosetta-Tsai d	29	63 (9.4)	1862 (43)	7.4 (2.1)	11 (3.9)	0.46 (0.08)	0.73 (0.04)
Rosetta-Baker d	57	88 (20)	100 (0)	8.5 (1.4)	15 (3.3)	0.45 (0.05)	0.76 (0.03)
CASP5 e	41	202 (78)	117 (41)	13 (3.7)	29 (14)	0.38 (0.12)	0.68 (0.08)
CASP6 e	39	172 (71)	216 (34)	13 (4.9)	27 (16)	0.39 (0.12)	0.70 (0.08)
CASP7 e	64	183 (80)	349 (40)	10 (3.4)	17 (10)	0.47 (0.11)	0.75 (0.07)
CASP8 e	77	187 (81)	334 (67)	8.8 (3.1)	13 (8.6)	0.54 (0.11)	0.79 (0.06)
CASP9 e	81	180 (81)	402 (95)	11 (4.9)	19 (14)	0.49 (0.12)	0.77 (0.07)
CASP-HRD e	109	188 (79)	192 (72)	2.8 (0.4)	2.2 (0.6)	0.76 (0.03)	0.89 (0.02)
CASP10-stage1 f	93	232 (102)	18 (1.9)	13 (4.3)	20 (9.4)	0.46 (0.08)	0.76 (0.05)
CASP10-stage2 f	93	232 (102)	132 (7.6)	11 (3.7)	17 (8.2)	0.55 (0.03)	0.80 (0.03)
TSA TM>0.5 g	242	179 (77)	291 (119)	6.3 (2.67)	9.4 (5.5)	0.63 (0.09)	0.82 (0.05)
TSA TM <0.5 g	303	110 (48)	602 (436)	12 (3.9)	23 (12)	0.34 (0.1)	0.68 (0.07)

a Training set (Titan HRD) and test set (Titan HRD*) from the Titan High resolution decoy set [20], available at http://titan.princeton.edu/2010-10-11/Decoys/.

b Tasser Set II is a structurally non-redundant set of protein structures and decoys derived with the program TASSER. It is available at http://zhanglab.ccmb.med.umich.edu/decoys/.

c Decoy sets from the Decoys ‘R’ us repository http://dd.compbio.washington.edu.

d Different decoy Rosetta-based decoy sets (see text for details), available at http://depts.washington.edu/bakerpg/decoys/.

e Collection of models from the successive CASP5 to CASP9 experiments, available from the CASP web site http://predictioncenter.org. CASP-HRD is a high resolution subset of the union of the five sets CASP5 to CASP9, which includes models that have a TM-score larger than 0.5 and a RMSD less than 4 Å to the native structures.

f The Stage_1 and Stage_2 decoy sets used in the CASP10 quality assessment category, available from the CASP web site http://predictioncenter.org. For details on how these sets are prepared, see [48].

g All high and low resolution targets (TSA TM-score>0.5)/(TSA TM-score <0.5) are listed in Files S1 and S2 respectively found in the supporting information.

h Nprot is the number of different proteins in the dataset, Nres is the average number of residues computed over all proteins in a dataset, and Ndecoys is the average number of decoys per proteins, averaged over the dataset. RMSD, MT, GDT-TS, and Q are the distance measures between the decoys and the corresponding native structures, averaged over all decoys and all proteins. We provide both the average values and the average mean absolute deviations (in parenthesis).

#### Preprocessing the decoy sets

To guarantee that the decoys included in a set are consistent in length with their corresponding native structure, we performed the following two-step preprocessing. First, we removed all residues in the decoys with missing backbone atoms (C*α*, N, C, and O). Second, we extracted the sequences from the decoy structure files and aligned these sequences with the native sequence of the protein of interest (where the native sequence is derived from the ATOM record in the corresponding PDB file). If these alignments include trailing unmatched residues either in the decoys or in the native structure, these residues are removed until all sequences are identical. We found that this procedure was necessary for some of the decoy sets described above.

### Assessing the quality of decoy selection: R-score

Given a distance measure and an energy function, an ensemble of decoy protein conformations contains a “best” distance model, i.e. the conformation that is closest geometrically to the native structure, as well as a “best” energy model, i.e. the model whose energy is the lowest. Ideally, these two “best” models should be the same; in practice however, they are different due to shortcomings of the potential energy function. To quantify this difference we introduce the R-score as follows. Let 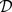 be the ensemble of decoys and let 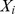 be one of its elements. The corresponding native structure is 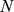. We define the mapping 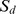 from 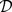 to 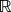 as 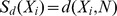, i.e. the distance between the decoy 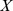 and 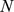, where 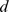 can be any of the four distance measures defined above. We name 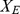 the decoy with the lowest energy, i.e. 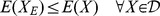. In parallel, we name 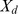 the decoy closest to 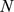 with respect of the distance 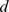, i.e. 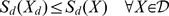. The R score for 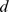 and 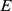 is defined as:
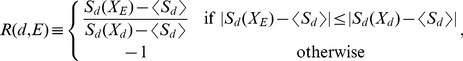
(10)


where 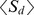 is the average value for 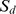 over the decoy set 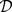. 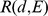 is designed to assess how well 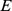 mimics 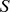 in finding the best decoy. It takes values between -1 and 1 where 1 indicates that the energy has picked the best decoy. We fix the lower limit at -1 to avoid having outliers being assigned very low negative values. Note, that if an ensemble does not contain outliers then 0 is the random expectation. If we furthermore assume that the distances 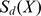 are uniformly distributed then 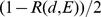 is the fraction of decoys with a distance to the native structure better than 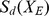. The 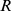 score can also be seen as the ratio between the 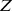-score of the best energy model, 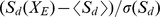, and the 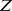-score of the best distance model, 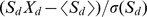, where 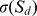 is the standard deviation for 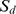 over the decoy set 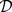.

### Assessing how well the energy functions mimic a funnel in the neighborhood of the native structure

To measure how far the energy 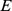 is from the desired linear funnel shape given by Equation 1 relative to the distance measure 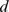 we report the Pearson's correlation coefficient 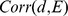 between the energy values 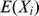 and distance measures 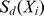 over all decoys 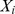 in the decoy set:
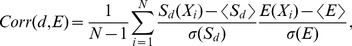
(11)


where 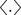 and 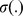 stand for the mean and standard deviation over the decoy set considered.

### Comparing two distance measures 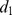 and 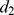


In the two previous subsections, we have defined a R-score 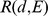 and a correlation coefficient 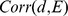 to measure how well an energy function *E* mimics a distance measure 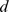. Both quantities can be used as is to compare two distance measures *d*_1_ and *d*_2_. Indeed, *d*_2_ can be assimilated to a pseudo energy function, akin to the definition of FlexE given in equation 4. The R-score and correlation coefficient between *d*_1_ and *d*_2_ are then simply 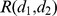 and 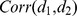, respectively. 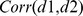 measures the dependence between 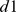 and 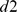 over a decoy set, while 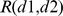 checks the “quality” of the best decoy identified by *d*_2_, as measured by *d*_1_. Note that this R-score between distance measures may not be symmetric.

## Results and Discussion

### The diversity of the distance measures

There is no unique way to compare three dimensional shapes. When comparing protein structures, two main classes of distance measures have been proposed, those based on a Euclidean distance between the positions of the atoms of the two proteins (after proper translation and rotation of one of them), and those based on the intrinsic geometry of the structures. We have considered two examples in each class, namely RMSD and GDT-TS* for the former, and MT and Q* for the latter. A full description of these four distance metrics is given in Material and Methods. As these measures capture changes of different geometric properties of the protein structures, there is no reason to believe that they are equivalent. To test the degrees to which these distances differ, we have compared them on three different sets of decoys, namely Titan-HRD, CASP-HRD, and TSA, using two different report scores, *Corr* and *R*, where *Corr* is the Pearson's correlation coefficient that measures how well *d*_1_ mimics *d*_2_ over a large range of distance values while *R* measures how (metrically) wrong the best candidate of one distance measure (i.e. the decoy with the smallest distance to its corresponding native structure) is when measured by another distance (see Materials and Methods for details). Results for *Corr* and *R* are given in tables 2 and 3, respectively.

**Table 2:** Correlations between the four distance measures.

		Distance *d*_2_			
Test set	Distance *d*_1_	RMSD	MT	GDT-TS*	Q*
Titan-HRD	RMSD	1^a^	0.92 (0.06)	0.92 (0.04)	0.87 (0.08)
	MT	0.92 (0.06)	1	0.92 (0.03)	0.94 (0.03)
	GDT-TS*	0.92 (0.04)	0.92 (0.03)	1	0.95 (0.03)
	Q*	0.87 (0.08)	0.94 (0.03)	0.95 (0.03)	1
CASP-HRD	RMSD	1	0.74 (0.16)	0.73 (0.14)	0.6 (0.19)
	MT	0.74 (0.16)	1	0.72 (0.13)	0.83 (0.07)
	GDT-TS*	0.73 (0.14)	0.72 (0.13)	1	0.74 (0.13)
	Q*	0.6 (0.19)	0.83 (0.07)	0.74 (0.13)	1
CASP10-stage1	RMSD	1	0.83 (0.16)	0.71 (0.24)	0.68 (0.24)
	MT	0.83 (0.16)	1	0.73 (0.2)	0.82 (0.14)
	GDT-TS*	0.71 (0.24)	0.73 (0.2)	1	0.86 (0.12)
	Q*	0.68 (0.24)	0.82 (0.14)	0.86 (0.12)	1
CASP10-stage2	RMSD	1	0.78 (0.16)	0.51 (0.22)	0.49 (0.19)
	MT	0.78 (0.16)	1	0.52 (0.2)	0.69 (0.14)
	GDT-TS*	0.51 (0.22)	0.52 (0.2)	1	0.64 (0.17)
	Q*	0.49 (0.19)	0.69 (0.14)	0.64 (0.17)	1
TSA	RMSD	1	0.92 (0.06)	0.8 (0.15)	0.82 (0.11)
	MT	0.92 (0.06)	1	0.78 (0.14)	0.85(0.08)
TM-score> 0.5	GDT-TS*	0.8 (0.15)	0.78 (0.14)	1	0.89 (0.12)
	Q*	0.82 (0.11)	0.85 (0.08)	0.89 (0.12)	1
TSA	RMSD	1	0.8 (0.12)	0.59 (0.24)	0.56 (0.18)
	MT	0.8 (0.12)	1	0.54 (0.2)	0.68(0.14)
TM-score <0.5	GDT-TS*	0.59 (0.24)	0.54 (0.2)	1	0.67 (0.22)
	Q*	0.56 (0.18)	0.68 (0.14)	0.67 (0.22)	1

a Pearson's correlation coefficient *Corr*(*d_1_*,*d_2_*) between the two distance measures *d*_1_ and *d*_2_. We provide both the average value and the mean absolute deviation (in parenthesis) over the data set considered.

**Table 3:** Comparing the best models picked by different distance measures.

		Distance *d*_2_			
Test set	Distance *d*_1_	RMSD	MT	GDT-TS*	Q*
Titan-HRD	RMSD	1^a^	0.88 (0.12)	0.91 (0.09)	0.76 (0.17)
	MT	0.94 (0.06)	1	0.92 (0.08)	0.91 (0.07)
	GDT-TS*	0.96 (0.04)	0.94 (0.07)	1	0.91 (0.08)
	Q*	0.87 (0.09)	0.92 (0.07)	0.89 (0.09)	1
CASP-HRD	RMSD	1	0.71 (0.26)	0.79 (0.22)	0.49 (0.38)
	MT	0.76 (0.22)	1	0.76 (0.22)	0.76 (0.23)
	GDT-TS*	0.8 (0.22)	0.68 (0.27)	1	0.48 (0.39)
	Q*	0.57 (0.33)	0.81 (0.16)	0.66 (0.24)	1
CASP10-stage1	RMSD	1	0.81 (0.24)	0.75 (0.31)	0.79 (0.23)
	MT	0.9 (0.13)	1	0.85 (0.19)	0.94 (0.09)
	GDT-TS*	0.79 (0.24)	0.78 (0.24)	1	0.82 (0.2)
	Q*	0.78 (0.22)	0.88 (0.14)	0.8 (0.23)	1
CASP10-stage2	RMSD	1	0.76 (0.22)	0.71 (0.3)	0.63 (0.29)
	MT	0.83 (0.18)	1	0.73 (0.24)	0.83 (0.19)
	GDT-TS*	0.73 (0.26)	0.65 (0.24)	1	0.59 (0.29)
	Q*	0.62 (0.29)	0.82 (0.18)	0.62 (0.23)	1
TSA	RMSD	1	0.9 (0.11)	0.84 (0.19)	0.81 (0.18)
	MT	0.94 (0.07)	1	0.88 (0.14)	0.92 (0.09)
TM-score> 0.5	GDT-TS*	0.85 (0.16)	0.79 (0.21)	1	0.73 (0.24)
	Q*	0.79 (0.18)	0.89 (0.11)	0.81 (0.16)	1
TSA	RMSD	1	0.83 (0.19)	0.73 (0.27)	0.71 (0.27)
	MT	0.87 (0.14)	1	0.74 (0.27)	0.88 (0.14)
TM-score <0.5	GDT-TS*	0.74 (0.27)	0.7 (0.27)	1	0.67 (0.27)
	Q*	0.68 (0.27)	0.85 (0.16)	0.68 (0.27)	1

a R-score *R*(*d_1_*,*d_2_*) between the two distance measures *d*_1_ and *d*_1_. We provide both the average value and the mean absolute deviation (in parenthesis) over the data set considered.

The correlations between the distance measures are high on the Titan-HRD set of decoys, with values above 0.87 for the correlation coefficients. The corresponding R-scores are above 0.76. If we assume uniform distributions of the native-decoy distances over a decoy set, the best decoy by one distance measure on average is ranked within the top 5% and within the top 12% by another distance measure for R scores of 0.9 and 0.76, respectively. These high scores are expected, as the Titan-HRD decoys are high resolution, usually very close to their native structure counterparts (see Table 1). It is interesting however that the R score between RMSD and Q* is relatively low (0.76), even on this high resolution data set. This low value indicates that a “good” decoy defined by Q* may explore a range of RMSD values. In contrast, a decoy that is close to the native structure with respect to RMSD usually has a high percentage of native contacts, as highlighted by the R score between Q* and RMSD of 0.87. In fact, we observe that the best RMSD decoy is generally scored better by the three other distance measures.

While CASP-HRD also contains high resolution decoys that are close to their corresponding native structures (with RMSD <4 Å and TM scores above 0.5), the four distance measures we tested are less dependent on this dataset than on Titan-HRD, both globally as scored by correlation coefficients and locally (i.e. in picking a “best” decoy), as highlighted by the R scores. We see two possible reasons for these differences between the two groups of decoy sets. First, the decoys in Titan-HRD are homogeneous, as they all contain the same hydrophobic cores as the native structures. In contrast, the CASP decoys were derived with many different methods, leading to heterogeneity in their geometry. Second, we cannot exclude an effect of sample size, as on average the sets included in Titan-HRD contain four times more decoys and larger average mean absolute deviation of distance measures than the sets included in CASP-HRD (see Table 1).

TSA, which stands for “Test Sets All” is a large heterogeneous collection of decoy sets that were generated by many different techniques (see Materials and methods for details). Some of these decoy sets are high-resolution, i.e. contains mostly native-like structures, while others are more diverse, containing decoys that are very different from their corresponding native structures, both in terms of secondary structure content and three-dimensional organization. To assess the importance of this diversity, we selected within the TSA group of decoy sets two subgroups, those for which the decoys have average TM score larger than 0.5, and those with average TM score smaller than 0.5. This 0.5 cutoff was again chosen based on the observation made by Xu and Zhang that two decoys belong to the same fold when their TM-scores to a native structure is higher than 0.5 [47]. Table 1 shows that TSA TM-score> 0.5 generally contain longer chains with fewer decoys when compared to the TSA TM-score <0.5 set. The two sets are fully listed in File S1 and File S2. Tables 2 and 3 show that the distance measures behave on the high-resolution subgroup (TM>0.5) as on the Titan-HRD test set, i.e. with high correlations and high R scores, meaning that they are very similar to each other. On the low-resolution subgroup (TM <0.5) however, the distance measures are poorly correlated with each other, with most correlation coefficients in the range 0.5 to 0.7. Both results confirm that when two structures are very close to each other, different distance measures quantify their differences in a similar manner. When the two structures however are very different, different distance measures will focus on different geometric differences, leading to differences in their behaviors. We observe however one exception in Table 2, in that RMSD and MT clearly remains correlated (0.80) even for the diverse subgroup of TSA with TM <0.5. The reason for this exception is unclear.

The CASP 10 Stage_1 and Stage_2 test sets usually include longer proteins than the other sets considered here, with decoys that are far from their native counterparts. In the Stage_1 sets there are very few decoys per target (by construction, see Methods above) and relatively large average mean deviations of the distance measures. For the Stage_2 test sets there are more decoys per target; these decoys however are usually very similar to each other, leading to very low mean absolute deviations for the GDT-TS* and Q* distance measures, and consequently to low correlations and R scores between the measures. As an example, the correlation between RMSD and GDT-TS* for the Stage_2 decoy sets is only 0.51 and their non symmetric R scores are R(RMSD,GDT-TS*) = 0.71 and R(GDT-TS*,RMSD) = 0.73, respectively. These low values are good indicators of significant differences between their ranking of the decoys included in CASP10 Stage_2 test sets.

### Training knowledge-based potentials with different distance measures

We have derived two new smooth knowledge-based residue pair potentials, PPD and PPE. Both potentials are based on distances between the 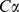 atoms of the protein structure of interest. For each of the 210 types of amino acid pairs, the two potentials are written as a weighted sum of smooth spline functions, whose weights are optimized so that the total energy of a protein model resembles the distance between the model and a reference structure (usually taken to be the native structure), as described by equation 1. The two potentials differ however on which pairs of residues are taken into account. While PPD includes all pairs of residues from the protein structure *P* considered, PPE only include those pairs whose inter 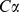 distance is consistently below a cutoff value in an ensemble of protein models similar to *P*. The idea behind PPE, derived from Eickholt et al. [36], is that the various models in the ensemble contain complementary information which can be pooled together to build a contact map of consistent residue-residue contacts that are more likely to be informative. Our interest here is to assess the influence of the distance measure used to train the two potentials. We have trained PPD and PPE on the Titan-HRD* training set with the four distance measures introduced above separately, and tested the corresponding four versions of the potentials against the Titan-HRD, CASP-HRD, and TSA test sets in their abilities to mimic any of the four distance measures. All parameters describing the amino acid pair spline potentials are listed in the file Force Field S1. The encoding used and the spline basis used is described in Readme Force Field S1. Both files are in the supporting information.

**Figure 1:**
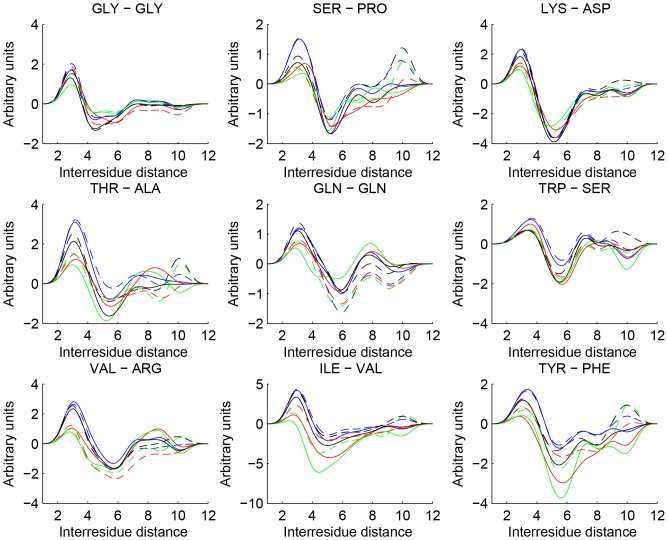
Showing nine different types of residue pair interactions for our single model method PPD (continuous lines) and our consensus method PPE (dotted lines) when trained on RMSD (blue), MT(red), GDT-TS(green) and Q(black).

Figure 1 shows some examples of the b-spline expanded pair potentials. As expected, the pair potentials are repulsive for short inter-residue distances and have a first minimum between 4 Å and 6 Å and this preferred distance relatively independent of the training metric. For longer pair distances it is seen that most PPD pair potentials have a local minimum around 10 Å whereas the PPE pair potentials tend to have a local maximum at this distance. One plausible explanation is that as PPE does not identify new contacts for these large distances; it may then set higher energy values for remote decoys. The exact placement of the minimum as well as the depth of the potential differs for the different pair potentials. While these differences may seem small, they add up when we sum over all the interactions.

**Figure 2:**
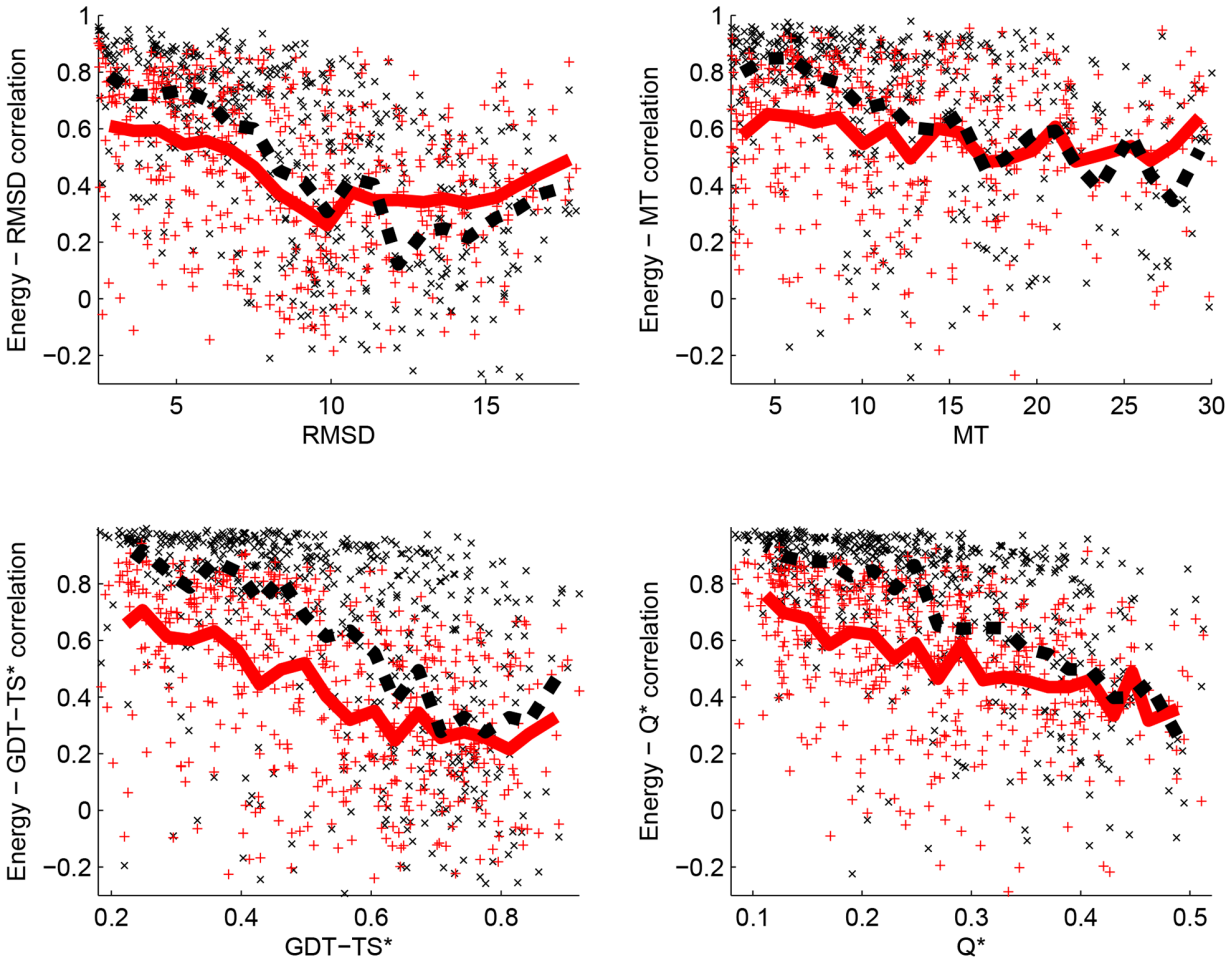
Energy-distance correlations as a function of the quality of the decoy set. For each decoy set in Titan-HRD, CASP-HRD, and TSA (a total of 797 sets), we plot the correlation Corr(E, *d*_1_) as a function of the mean value of *d*_1_ over the decoy set, where *E* is either the PPD energy (red, plus sign +) or the PPE energy (black, cross sign x) trained on the set Titan-HRD with the distance measure *d*_1_, and *d*_1_ is one of the fourth distance measures considered, namely RMSD (panel A), MT (panel B), GDT-TS* (panel C), and Q* (panel D). The corresponding running means computed over 20 equidistant intervals for PPD (red, solid line) and PPE (black, dashed line) are shown. Clearly, the quality of the correlation energy-distance decreases as the diversity of the decoy set increases.

**Figure 3:**
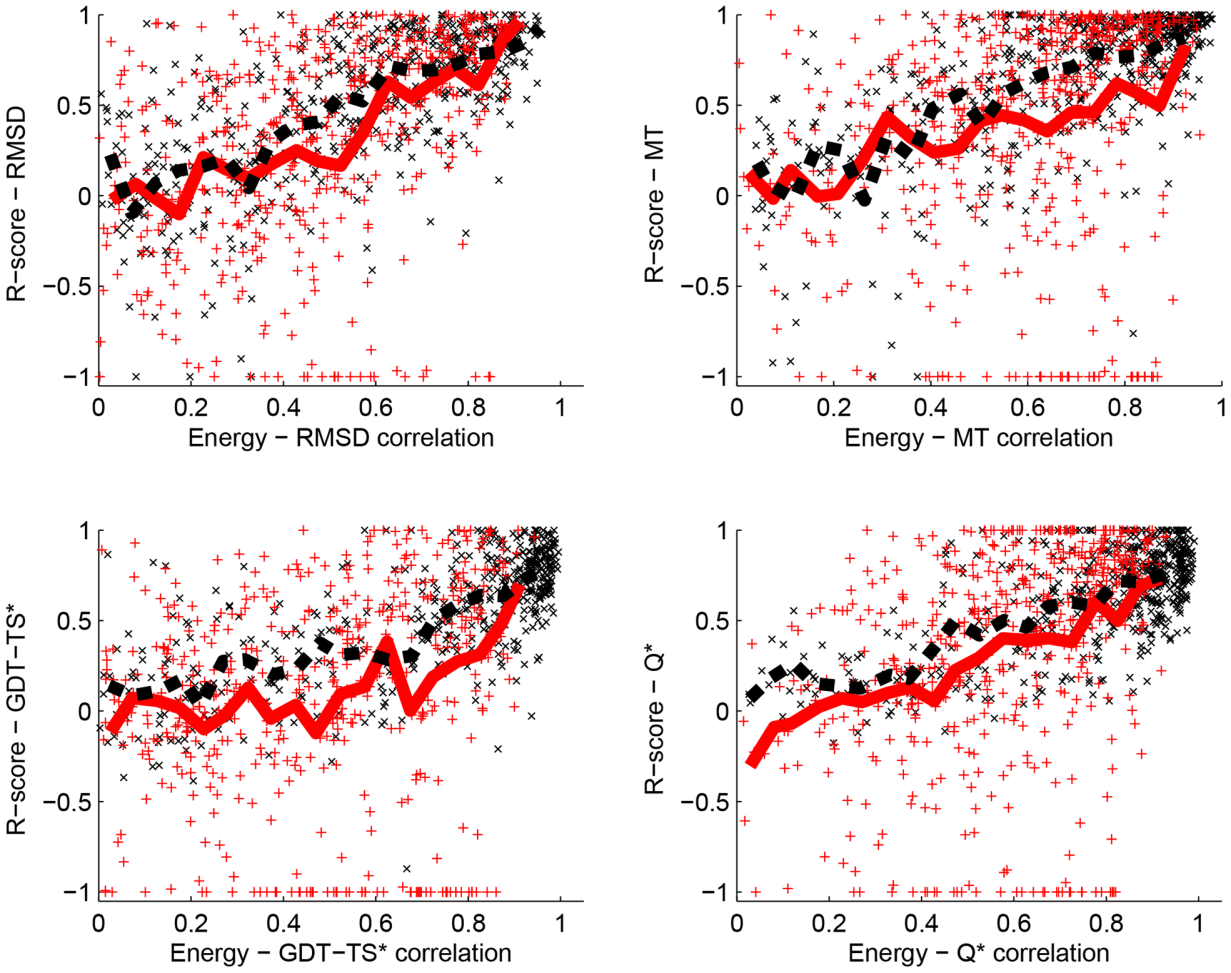
R scores versus Energy-distance correlations. For each decoy set in Titan-HRD, CASP-HRD, and TSA, we plot the R score 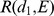 as a function of the correlation coefficient 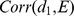, where *E* is either the PPD energy (red, plus sign +) or the PPE energy (black, cross sign x) trained on the set Titan-HRD with the distance measure *d*_1_, and *d*_1_ is one of the fourth distance measures considered, namely RMSD (panel A), MT (panel B), GDT-TS* (panel C), and Q* (panel D). The corresponding running means computed over 20 equidistant intervals for PPD (red, solid line) and PPE (black, dashed line) are shown. Note that 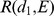 compares the best decoy picked based on the energy value *E* with the decoy closest to the native structure according to the distance measure *d*_1_. There is a clear correlation between these two values for all four distance measures.

We computed both the correlations between energy and the distance measure, and the R scores that compare the best decoys picked based on energy with the decoys closest to their corresponding native structures. Results are given in Table 4 for the correlation coefficients, Table 5 for the R scores, and in Figures 2 and 3 for a comparison of these scores. We draw from these tables and figures the four main conclusions described below.

**Table 4:** Energy-distance correlations.

		PPD				PPE				RAPDF a	GOAP^b^	AMBER c
		Training distance *d*_1_ d				Training distance *d*_1_ d						
Decoy set	Test Distance *d*_2_ d	RMSD	MT	GDT-TS*	Q*	RMSD	MT	GDT-TS*	Q*			
Titan-HRD	RMSD	0.77 (0.05) e	0.82 (0.04)	0.81 (0.05)	0.79 (0.04)	0.81 (0.05)	0.88 (0.03)	0.85 (0.03)	0.82 (0.04)	0.5 (0.14)	0.64(0.11)	0.01 (0.02)
	MT	0.82 (0.04)	0.89 (0.03)	0.87 (0.03)	0.87 (0.03)	0.86 (0.04)	0.95 (0.01)	0.92 (0.02)	0.89 (0.02)	0.47 (0.16)	0.63(0.11)	0.01 (0.02)
	GDT-TS*	0.83 (0.06)	0.91 (0.02)	0.91 (0.02)	0.9 (0.03)	0.86 (0.04)	0.93 (0.02)	0.95 (0.01)	0.92 (0.02)	0.43 (0.18)	0.63(0.12)	0.001 (0.02)
	Q*	0.82 (0.05)	0.92 (0.2)	0.92 (0.02)	0.93 (0.02)	0.87 (0.03)	0.95 (0.01)	0.97 (0.01)	0.95 (0.01)	0.37 (0.22)	0.57(0.13)	0.002 (0.02)
CASP-HRD	RMSD	0.32 (0.16)	0.31 (0.16)	0.31 (0.16)	0.26 (0.16)	0.42 (0.16)	0.51 (0.17)	0.51 (0.18)	0.45 (0.17)	0.31(0.15)	0.3 (0.15)	0.01 (0)
	MT	0.45 (0.11)	0.49 (0.11)	0.49 (0.12)	0.43 (0.12)	0.55 (0.13)	0.69 (0.11)	0.68 (0.13)	0.61 (0.13)	0.37 (0.14)	0.41(0.13)	0.02 (0)
	GDT-TS*	0.38 (0.14)	0.39 (0.13)	0.39 (0.13)	0.33 (0.14)	0.51 (0.15)	0.6 (0.14)	0.65 (0.14)	0.58 (0.17)	0.39 (0.14)	0.43(0.11)	0.02 (0)
	Q*	0.46 (0.12)	0.6(0.11)	0.64 (0.1)	0.57 (0.1)	0.54 (0.12)	0.69 (0.12)	0.75 (0.12)	0.71 (0.1)	0.32 (0.15)	0.42(0.12)	0.02 (0)
CASP10-stage1	RMSD	0.44 (0.22)	0.53 (0.18)	0.53 (0.18)	0.48 (0.2)	0.5 (0.22)	0.54 (0.22)	0.52 (0.21)	0.5 (0.21)	0.18(0.24)	0.32 (0.26)	−0.03 (0)
	MT	0.47 (0.19)	0.61(0.17)	0.62 (0.17)	0.55 (0.18)	0.56 (0.16)	0.63 (0.13)	0.61 (0.13)	0.57 (0.13)	0.13 (0.22	0.34(0.24)	−0.06 (0)
	GDT-TS*	0.4 (0.21)	0.49 (0.23)	0.51 (0.2)	0.43 (0.21)	0.57 (0.21)	0.63 (0.16)	0.63 (0.16)	0.59 (0.2)	0.22 (0.28)	0.4(0.2)	−0.05 (0)
	Q*	0.51 (0.22)	0.63(0.16)	0.63 (0.16)	0.56 (0.18)	0.68 (0.12)	0.75 (0.06)	0.75 (0.07)	0.72 (0.1)	0.24 (0.3)	0.41(0.2)	−0.05 (0)
CASP10-stage2	RMSD	0.33 (0.18)	0.34 (0.2)	0.34 (0.2)	0.31 (0.2)	0.31 (0.18)	0.37 (0.19)	0.34 (0.16)	0.3 (0.21)	0.15(0.13)	0.2 (0.14)	−0.005 (0)
	MT	0.42 (0.16)	0.49 (0.16)	0.49 (0.15)	0.45 (0.14)	0.4 (0.16)	0.5 (0.19)	0.48 (0.15)	0.42 (0.16)	0.19 (0.14)	0.29(0.14)	0.003 (0)
	GDT-TS*	0.31 (0.15)	0.29 (0.14)	0.29 (0.13)	0.25 (0.14)	0.37 (0.16)	0.42 (0.19)	0.44 (0.18)	0.38 (0.18)	0.29 (0.14)	0.37(0.17)	0.007 (0)
	Q*	0.45 (0.22)	0.56(0.14)	0.58 (0.12)	0.52 (0.15)	0.51 (0.18)	0.62 (0.17)	0.66 (0.14)	0.62 (0.15)	0.28(0.13)	0.41(0.13)	−0.006 (0)
TSA	RMSD	0.62 (0.12)	0.62 (0.13)	0.63 (0.13)	0.59 (0.14)	0.74 (0.09)	0.8 (0.07)	0.78 (0.08)	0.73 (0.09)	0.5 (0.14)	0.58(0.13)	0.02 (0.01)
	MT	0.65 (0.11)	0.69 (0.1)	0.7 (0.1)	0.65 (0.12)	0.75 (0.08)	0.83 (0.06)	0.8 (0.06)	0.74 (0.07)	0.5 (0.16)	0.58(0.12)	0.03 (0.01)
TM-score > 0.5	GDT-TS*	0.6 (0.15)	0.59 (0.14)	0.6 (0.13)	0.54 (0.16)	0.78 (0.06)	0.85 (0.04)	0.84 (0.04)	0.79 (0.06)	0.61 (0.11)	0.7(0.1)	0.03 (0.01)
	Q*	0.69 (0.11)	0.71 (0.09)	0.72 (0.1)	0.68 (0.1)	0.87 (0.04)	0.94 (0.02)	0.93 (0.02)	0.9 (0.03)	0.57 (0.13)	0.67(0.11)	0.03 (0.01)
TSA	RMSD	0.3 (0.16)	0.34 (0.18)	0.34 (0.18)	0.32 (0.18)	0.29 (0.23)	0.36 (0.27)	0.34 (0.27)	0.29 (0.23)	0.16 (0.13)	0.25(0.15)	0 (0.01)
	MT	0.38 (0.14)	0.47 (0.15)	0.47 (0.15)	0.45 (0.17)	0.34 (0.23)	0.45 (0.24)	0.41 (0.23)	0.35 (0.22)	0.19 (0.14)	0.29(0.13)	−0.003(0.01)
TM-score < 0.5	GDT-TS*	0.27 (0.19)	0.27 (0.2)	0.28 (0.19)	0.24 (0.18)	0.36 (0.29)	0.44 (0.33)	0.42 (0.32)	0.36 (0.29)	0.26 (0.16)	0.33(0.19)	0.004 (0.02)
	Q*	0.41 (0.17)	0.47 (0.16)	0.46 (0.17)	0.45 (0.15)	0.53 (0.19)	0.63 (0.18)	0.61 (0.18)	0.57 (0.19)	0.23 (0.17)	0.3(0.19)	−0.004 (0.02)

a All-atom statistical distance-based potential [49].

b All-atom orientation-dependent statistical potential [7].

c The semi-empirical physical potential AMBER99SB-ILDN[50].

d PPD and PPE have been trained on the distance measure *d*_1_ and tested against the distance measure *d*_2_.

e Average value, and mean absolute deviation (in parenthesis) over the data set.

**Table 5:** Energy-distance Rvalues.

		PPD				PPE				RAPDF a	GOAP b	AMBER c
		Training distance *d*_1_ d				Training distance *d*_1_ d						
Decoy set	Test Distance *d*_2_ d	RMSD	MT	GDT-TS*	Q*	RMSD	MT	GDT-TS*	Q*			
Titan-HRD	RMSD	0.57 (0.17)^e^	0.52 (0.17)	0.51 (0.19)	0.48 (0.2)	0.61 (0.14)	0.63 (0.17)	0.62 (0.14)	0.61 (0.15)	0.43 (0.18)	0.56 (0.15)	0.26 (0.47)
	MT	0.73 (0.1)	0.71 (0.11)	0.69 (0.13)	0.69 (0.15)	0.76 (0.11)	0.8 (0.11)	0.79 (0.12)	0.8 (0.1)	0.5 (0.18)	0.6(0.17)	0.23 (0.53)
	GDT-TS*	0.78 (0.08)	0.74 (0.09)	0.74 (0.08)	0.73 (0.09)	0.82 (0.07)	0.86 (0.06)	0.86 (0.07)	0.86 (0.07)	0.52 (0.22)	0.67(0.12)	0.18 (0.58)
	Q*	0.73 (0.11)	0.76 (0.12)	0.73 (0.1)	0.77 (0.1)	0.77 (0.09)	0.84 (0.09)	0.85 (0.09)	0.86 (0.08)	0.37 (0.19)	0.53(0.17)	0.16 (0.51)
CASP-HRD	RMSD	0.19 (0.31)	0.00 (0.42)	−0.04 (0.48)	0.03 (0.4)	0.27 (0.3)	0.33 (0.31)	0.34 (0.27)	0.3 (0.31)	0.14 (0.37)	0.22(0.37)	−0.11 (0.34)
	MT	0.31 (0.26)	0.24 (0.3)	0.14 (0.31)	0.16 (0.36)	0.38 (0.24)	0.43 (0.22)	0.43 (0.21)	0.38 (0.25)	0.24 (0.4)	0.46(0.32)	−0.09 (0.47)
	GDT-TS*	0.14 (0.3)	−0.09 (0.4)	−0.08 (0.42)	−0.06 (0.43)	0.28 (0.23)	0.31 (0.25)	0.32 (0.24)	0.28 (0.24)	0.12 (0.44)	0.34(0.33)	−0.22 (0.38)
	Q*	0.27 (0.25)	0.35 (0.33)	0.34 (0.32)	0.33 (0.31)	0.28 (0.2)	0.39 (0.24)	0.4 (0.25)	0.41 (0.26)	0.13 (0.4)	0.43(0.26)	−0.17(0.42)
CASP10-stage1	RMSD	0.55 (0.23)	0.53 (0.3)	0.57(0.26)	0.48 (0.39)	0.5 (0.29)	0.52 (0.26)	0.53 (0.26)	0.51 (0.27)	0.32 (0.34)	0.44(0.26)	0.13 (0.6)
	MT	0.69(0.1)	0.7(0.12)	0.72(0.12)	0.64(0.14)	0.58(0.15)	0.62(0.12)	0.63(0.13)	0.6(0.15)	0.37(0.28)	0.52(0.2)	0.16(0.6)
	GDT-TS*	0.52(0.32)	0.47(0.39)	0.52(0.37)	0.42(0.43)	0.43(0.4)	0.46(0.37)	0.51(0.33)	0.46(0.36)	0.27(0.44)	0.53(0.22)	0.15(0.37)
	Q*	0.6(0.24)	0.64(0.15)	0.67(0.14)	0.57(0.19)	0.57(0.22)	0.6(0.18)	0.63(0.17)	0.61(0.18)	0.32(0.38)	0.47(0.31)	0.19(0.5)
CASP10-stage2	RMSD	0.38(0.29)	0.23(0.36)	0.29(0.3)	0.26(0.35)	0.36(0.31)	0.35(0.32)	0.32(0.32)	0.35(0.34)	0.29(0.28)	0.39(0.29)	0.11(0.42)
	MT	0.55(0.23)	0.46(0.31)	0.5(0.29)	0.49(0.33)	0.45(0.32)	0.47(0.32)	0.47(0.32)	0.48(0.3)	0.44(0.32)	0.52(0.24)	0.17(0.45)
	GDT-TS*	0.23(0.35)	0.11(0.33)	0.14(0.36)	0.14(0.34)	0.25(0.32)	0.23(0.28)	0.29(0.32)	0.25(0.32)	0.23(0.3)	0.39(0.3)	0.01(0.27)
	Q*	0.45(0.32)	0.46(0.31)	0.51(0.29)	0.48(0.3)	0.44(0.3)	0.47(0.24)	0.51(0.27)	0.53(0.27)	0.33(0.28)	0.41(0.27)	0.13(0.43)
TSA	RMSD	0.47 (0.24)	0.22 (0.41)	0.21 (0.41)	0.22 (0.41)	0.69 (0.14)	0.69(0.13)	0.69 (0.13)	0.68 (0.14)	0.44 (0.25)	0.59(0.19)	0.24 (0.38)
	MT	0.62 (0.14)	0.4 (0.24)	0.39 (0.27)	0.41 (0.24)	0.8 (0.08)	0.81 (0.07)	0.82 (0.08)	0.8 (0.08)	0.54 (0.18)	0.74(0.1)	0.32 (0.33)
TM-score> 0.5	GDT-TS*	0.29 (0.28)	0.09 (0.43)	0.08 (0.47)	0.09 (0.45)	0.58 (0.16)	0.6 (0.16)	0.61 (0.16)	0.57 (0.18)	0.37 (0.31)	0.56(0.41)	0.09 (0.26)
	Q*	0.49 (0.19)	0.32 (0.3)	0.3 (0.33)	0.33 (0.28)	0.68 (0.14)	0.72 (0.14)	0.74 (0.13)	0.74 (0.13)	0.38 (0.29)	0.59(0.43)	0.14 (0.2)
TSA	RMSD	0.16 (0.35)	0.19 (0.3)	0.19 (0.34)	0.16 (0.32)	0.26 (0.35)	0.33 (0.37)	0.32 (0.36)	0.29 (0.35)	0.19 (0.35)	0.27(0.4)	0.04 (0.41)
	MT	0.27 (0.34)	0.38 (0.3)	0.39 (0.33)	0.37 (0.29)	0.36 (0.33)	0.44 (0.31)	0.42 (0.32)	0.41 (0.31)	0.28 (0.34)	0.4(0.33)	0.04 (0.46)
TM-score <0.5	GDT-TS*	0.07 (0.3)	0.07 (0.28)	0.1 (0.3)	0.06 (0.29)	0.26 (0.3)	0.32 (0.3)	0.32 (0.3)	0.29 (0.3)	0.19 (0.3)	0.28(0.36)	0.05 (0.3)
	Q*	0.18 (0.35)	0.28 (0.34)	0.29 (0.35)	0.3 (0.31)	0.42 (0.27)	0.5 (0.27)	0.49 (0.29)	0.49 (0.27)	0.19 (0.29)	0.31(0.32)	0.01 (0.31)

a All-atom statistical distance-based potential [49].

b All-atom orientation-dependent statistical potential [7].

c The semi-empirical physical potential AMBER99SB-ILDN [50].

d PPD and PPE have been trained on the distance measure *d*_1_ and tested against the distance measure *d*_2_.

e Average value, and mean absolute deviation (in parenthesis) over the data set.

First, we find that both potentials PPD and PPE perform very well on the Titan-HRD test set, for all distance measures used for training and testing the potential. The corresponding mean correlation coefficients (averaged over all decoys sets in Titan-HRD) are usually above 0.8, indicating that the energy functions order the decoys in the same manner as the distance measures. In parallel, the R scores are also high, with most values well above 0.65, indicating that the decoys with the lowest energies are usually among the decoys that are close to the corresponding native structures. We should note however that PPD and PPE were trained on Titan-HRD*. While Titan-HRD and Titan-HRD* are different (see Methods), they both contain decoys that were generated with the same principles, with the significant constraint that they maintain the hydrophobic cores of the corresponding native structures. The exceptional performance of PPD and PPE may therefore not be surprising in light of this comment. Indeed, as we test these potentials on different decoy sets with more diverse populations of decoys, we observe a decrease in performance that follows the increase in diversity (in the order Titan-HRD - TSA (TM 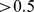) - CASP-HRD - TSA (TM 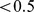). This decrease in performance is illustrated in Figure 2.

Second, the ensemble potential PPE performs better than the single structure potential PPD, again for all the distance measures used to train and test the potentials. The differences between the two potentials are large for the high resolution decoys sets in Titan-HRD and TSA (TM>0.5), but become statistically insignificant for very diverse decoy sets such as those in TSA (TM <0.5). We believe that these differences illustrate the power of generating consensus information from an ensemble. In PPE, we only consider those contacts there are consistently below a given distance cutoff in the whole decoy set to which the protein of interest belongs. This initial filtering is clearly an advantage for Titan-HRD, as it will select the contacts in the hydrophobic cores which are native, and will ignore the contacts that fluctuate significantly due to the sampling procedure used to generate the decoys. It remains an advantage for high quality decoy but becomes less pertinent for highly diverse decoys.

Third, the performances of the two potentials PPD and PPE depend on the choice of the distance used in the training step. For example, the correlations between PPE and any of the four distance measures increase on average by 0.09 when it is trained on MT instead of RMSD (Table 4). Similar differences are observed for the R scores between PPE and the four distance measures (Table 5). More generally, it is best to train the potentials on a distance measure that is directly based on intrinsic inter-residue distances, such as MT that follows the elastic network of the protein of interest, or Q* that counts the number of contacts that fall below a given distance cutoff, than on a distance measure based on extrinsic Euclidean distances, such as RMSD. Interestingly, we find that GDT-TS* behaves more like the intrinsic distance measures MT and Q* than RMSD, even though it is also based on extrinsic distances. The reason for this discrepancy is unclear.

Finally, we observe that the ability of an energy function to pick a “good” decoy (i.e. with native-like characteristics) is contingent to how well this energy function correlates with a distance measure between decoys and native structure. This is illustrated in Figure 2. This observation validates the approach of sculpting (training) a potential to mimic a distance measure.

### Comparison with other energy functions

We have compared the two energy functions PPD and PPE with two well established all-atom statistical potentials RAPDF [49] and GOAP [7] and with a semi-empirical physical potential, AMBER99SB-ILDN [50], for all decoy sets in Titan-HRD, CASP-HRD, and TSA. Results for correlations between energy and distance measures and for R scores are given in Tables 4 and 5, respectively.

As intuitively expected, the performances of AMBER99SB-ILDN are very poor. This is most likely an artifact due to the presence of a few steric clashes in the decoys, and not a reflection of the quality of this potential. While it would be possible to improve this performance by applying an initial energy minimization on all decoys, this result by itself highlights that such a physical potential cannot be used directly to order a set of decoys, unless some pre-processing is applied.

RAPDF is a knowledge-based statistical potential that is based on a direct conversion of the distributions of inter-atomic distances observed in native protein structures into energy values that are then used to assess how native-like a model is [49]. It is not based on any information from existing decoy sets, and it is not trained to mimic some differences between decoys and native structures. It is therefore not surprising that it does not perform as well as PPD and PPE, especially on the Titan-HRD as both PPD and PPE were trained on decoys resembling those included in this data set.

GOAP is an all-atom orientation-dependent knowledge-based statistical potential that includes a distance-based term and an angle-dependent contribution [7]. The distance-based term is an all-atom statistical potential that is based on the reference state that was introduced with the DFIRE potential [51]. The angle dependent component of GOAP is based on the geometric orientation of local planes. GOAP is found to perform significantly better than RAPDF on all datasets tested in this study. This is not a surprise, as GOAP includes much more information than RAPDF due to its angle term. We find however that GOAP performs only marginally better than PPD and worse than PPE. This illustrates the benefit of training a potential on a decoy set. PPD and PPE are only Ca based potentials; they have been trained however to mimic distances between non-native models and native structures of proteins.

The performances of RAPDF and GOAP depend on the distance measure used for testing. We observe that they are particularly good when the statistical potentials are tested on GDT-TS*, reflecting the differences between these distance measures (see Table 2 and 3).

### Performance in the CASP 10 quality assessment category

As part of the CASP experiment, state-of-the-art methods for protein structure assessment are judged on their ability to evaluate the quality of the predictions submitted as models for the targets considered in that specific experiment: this is the quality assessment category (QA). In 2012 as part of CASP10, 37 groups participated [48]. They were asked to evaluate the quality of sets of predictions (decoys) in two rounds designated as Stage_1 (20 decoys with a large variation in quality as measured by GDT-TS) and Stage_2 (150 decoys with homogeneous quality as measured by GDT-TS). The main reason for providing a small number of decoys in Stage_1 was to allow for judging assessment methods that rely on a single model independently from methods that rely on an ensemble of decoys (consensus methods), that would be tested extensively with the Stage_2 decoy sets. The three main conclusions drawn from these experiments were [48]: 1) The performances of the single model methods are usually worse than the the performances of consensus methods, 2) The Stage_2 sets are usually more difficult to rank than the Stage_1 sets, and 3) No methods were able to consistently pick the best decoy in an ensemble. The results for the participating groups can be seen in Figure 2 (average correlation) and Figure 3 (ability to pick the best decoy) in [48]. We note that the single model method GOAP used in this study differs from the quasi-single model method GOAPQA used in CASP10QA. For the latter, the TM-score [46] to the top 5 ranked models is used as a measure of model quality.

The CASP 10 datasets have average native-decoy RMSDs of 11–13 Å. These differences are significantly larger than the 2.4 Å RMSD found in our training sets (see Table 1). Our analyses of the performances of PPD (single model) and PPE (ensemble of decoys) on the other datasets considered in this study have shown that for decoys that are far from their native counterparts, the two methods perform similarly, and in fact poorly (see top left panel of Figure 2 and Table Table 4). We observe the same behavior when PPD and PPE are applied on the CASP10 datasets (Tables 4 and 5). Similarly we expect and indeed find that the ensemble method PPE is ineffective in ranking the decoys of the CASP10 datasets when its performance is measured against the MT distance measure, and shows some prospects when its performance is measured against the GDT-TS* and Q* distance measures. The energy-GDT-TS correlations of 0.51(0.63) and 0.29(0.44) for PPD(resp. PPE) on Stage_1 and Stage_2 respectively are amongst the lowest reported for single model(resp. ensemble) methods in CASP10QA [48]. The low energy-distance correlations reported usually leads to a bad pick for the best decoy, see Figure 3. It is therefore surprising that the average 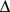
GDT-TS* of 0.07 between the GDT-TS*-closest decoy and the lowest energy decoy picked by PPE on the CASP10 Stage_2 data sets places PPE in the middle of the CASP10 participating methods (see [48] Figure 2(A)).

The results for PPD, PPE, AMBER99SB-ILDN, RAPDF and GOAP on CASP 10 stages 1 and 2 are given in Tables 4 - 6 where PPD and PPE were trained and tested on the same distance measure. Clearly, GOAP has a better performance than PPD when GDT-TS* is chosen as a measure of distance. It is however noteworthy that PPD performs better than GOAP when measured by RMSD and MT instead. It is encouraging that the distance dependent C-alpha potential, PPD, as a single model method has a performance that is comparable to the state-of-the-art orientation-dependent all-atom potential, GOAP. We find that PPD is good at selecting a decoy that is close to the native structure (Table 6).

**Table 6:** Assessing the best decoys selected by energy functions on different decoy datasets.

		Best	PPD	PPE	RAPDF a	AMBER b	GOAP c
Titan-HRD	RMSD	1.1(0.21)^d^	1.7(0.29)	1.6(0.27)	1.9(0.4)	2.1(0.55)	1.7(0.3)
	MT	0.75(0.22)	1.4(0.38)	1.2(0.38)	1.8(0.62)	2.3(0.89)	1.6(0.54)
	GDT-TS	0.94(0.02)	0.89(0.03)	0.92(0.03)	0.85(0.05)	0.8(0.09)	0.88(0.03)
	Q	0.94(0.01)	0.92(0.02)	0.93(0.02)	0.88(0.03)	0.86(0.04)	0.89(0.03)
4-state	RMSD	1.1(0.1)	3.8(0.44)	2.2(0.21)	2.1(0.22)	3.6(1.5)	1.6(0.24)
	MT	0.9(0.33)	5.8(2.1)	1.2(0.31)	2.6(0.52)	6.2(3.4)	1.5(0.38)
	GDT-TS	0.91(0.03)	0.55(0.06)	0.86(0.08)	0.8(0.04)	0.67(0.1)	0.86(0.04)
	Q	0.94(0.02)	0.75(0.03)	0.92(0.02)	0.87(0.04)	0.79(0.1)	0.9(0.02)
fisa	RMSD	3.7(0.76)	5.7(0.78)	6.5(1.4)	4.4(0.72)	8.5(1.5)	4.5(0.45)
	MT	3.8(1.5)	7.9(3.7)	5.5(2)	5.4(2.5)	10(4.2)	4.9(1.9)
	GDT-TS	0.65(0.07)	0.51(0.14)	0.54(0.08)	0.6(0.06)	0.46(0.08)	0.59(0.06)
	Q	0.82(0.02)	0.79(0.03)	0.78(0.03)	0.78(0.02)	0.73(0.02)	0.79(0.03)
fisa CASP3	RMSD	6(2)	12(1.6)	12(2.4)	12(4)	12(1)	11(1.6)
	MT	8.7(4.2)	21(7.3)	17(8.2)	23(4.5)	19(2.5)	18(7.7)
	GDT-TS	0.47(0.12)	0.32(0.01)	0.34(0.02)	0.32(0.02)	0.29(0.01)	0.33(0.04)
	Q	0.76(0.06)	0.72(0.04)	0.72(0.04)	0.68(0.04)	0.67(0.07)	0.69(0.06)
hg Structal	RMSD	1.9(0.5)	2.6(1)	2.5(0.56)	2.2(0.5)	3.3(0.71)	2.4(0.6)
	MT	1.8(0.3)	2.5(0.61)	3(0.28)	2.4(0.35)	3.7(0.8)	2.7(0.28)
	GDT-TS	0.86(0.06)	0.82(0.14)	0.85(0.07)	0.84(0.07)	0.77(0.08)	0.84(0.08)
	Q	0.93(0.03)	0.92(0.03)	0.92(0.03)	0.92(0.04)	0.89(0.03)	0.92(0.04)
lmds	RMSD	5.7(0.33)	9.9(0.72)	9.8(0.89)	9.8(0.92)	10(0.61)	10(0.65)
	MT	8(0.78)	14(3.7)	17(5.5)	16(2.5)	19(1.6)	19(4.5)
	GDT-TS	0.45(0.04)	0.29(0.04)	0.32(0.05)	0.31(0.05)	0.28(0.03)	0.3(0.03)
	Q	0.74(0.02)	0.67(0.05)	0.67(0.04)	0.65(0.04)	0.63(0.03)	0.63(0.05)
lattice ssfit	RMSD	3.8(0.46)	7.6(1.3)	7.4(1.6)	7.7(1.9)	8(2.6)	8.5(1.2)
	MT	5.2(2.2)	9.8(4.5)	10(5.1)	11(4.8)	12(6.4)	12(5)
	GDT-TS	0.62(0.06)	0.45(0.07)	0.48(0.07)	0.49(0.12)	0.45(0.07)	0.44(0.04)
	Q	0.8(0.06)	0.74(0.07)	0.75(0.04)	0.74(0.05)	0.72(0.07)	0.72(0.06)
CASP5	RMSD	6.7(2.9)	13(6.2)	11(6)	10(5.2)	11(6.1)	10(5.2)
	MT	8.5(4.2)	20(11)	18(7.7)	20(12)	22(11)	20(9.9)
	GDT-TS	0.58(0.19)	0.36(0.17)	0.48(0.24)	0.44(0.19)	0.46(0.21)	0.5(0.23)
	Q	0.82(0.09)	0.72(0.13)	0.77(0.09)	0.7(0.1)2	0.72(0.13)	0.75(0.1)
CASP6	RMSD	4.8(1.5)	10(5.1)	11(4.5)	9.7(5.1)	12(5.9)	8(3.1)
	MT	5.4(1.9)	23(11)	18(4.3)	19(5.7)	24(15)	12(4)
	GDT-TS	0.64(0.14)	0.33(0.14)	0.52(0.18)	0.49(0.27)	0.38(0.17)	0.54(0.17)
	Q	0.85(0.06)	0.7(0.07)	0.79(0.08)	0.75(0.11)	0.68(0.11)	0.79(0.09)
CASP7	RMSD	4.5(1.8)	8.8(4.9)	7.1(3.1)	7.9(3.9)	11(5.1)	7.8(3.4)
	MT	3.8(1.6)	9.5(3.8)	6.6(2.8)	10(4.3)	18(9.1)	8.3(3.5)
	GDT-TS	0.66(0.13)	0.49(0.21)	0.56(0.14)	0.56(0.17)	0.43(0.21)	0.58(0.13)
	Q	0.88(0.04)	0.81(0.08)	0.85(0.06)	0.81(0.06)	0.74(0.08)	0.82(0.06)
CASP8	RMSD	4.1(1.3)	7.4(2.7)	6.4(1.8)	9.8(5.5)	9.7(5.2)	7.5(3.1)
	MT	3.2(1.3)	10(4.1)	6.1(2.2)	14(6.4)	15(8)	8.7(2.7)
	GDT-TS	0.7(0.1)	0.53(0.17)	0.63(0.13)	0.51(0.22)	0.47(0.19)	0.61(0.16)
	Q	0.89(0.04)	0.81(0.07)	0.85(0.06)	0.79(0.09)	0.75(0.1)	0.83(0.07)
CASP9	RMSD	4.8(1.4)	9.7(4.9)	7.5(2.6)	9.4(4.7)	9.8(4.9)	8.2(3.3)
	MT	3.7(1.3)	14(7.8)	7.3(2.6)	12(3.9)	13(5.2)	8.5(2.6)
	GDT-TS	0.68(0.1)	0.4(0.15)	0.6(0.12)	0.52(0.19)	0.51(0.21)	0.57(0.15)
	Q	0.88(0.04)	0.75(0.12)	0.85(0.04)	0.8(0.09)	0.79(0.09)	0.83(0.07)
TASSER Set II	RMSD	3.2(1)	5.6(1.9)	5.2(1.4)	5.2(1.6)	6.4(2.1)	5.4(1.9)
	MT	3.7(1.2)	7.1(2.3)	6.6(2.5)	6.7(2.2)	11(5.4)	6.8(2.2)
	GDT-TS	0.69(0.09)	0.57(0.12)	0.59(0.12)	0.59(0.1)	0.52(0.13)	0.59(0.12)
	Q	0.85(0.05)	0.81(0.06)	0.82(0.06)	0.8(0.05)	0.75(0.08)	0.79(0.05)
Rosetta-All	RMSD	6.4(1.3)	11(2.1)	11(2.2)	12(3.2)	16(4.7)	11(2.6)
	MT	12(4.7)	22(8.9)	26(7.2)	25(9.9)	47(14)	24(7.5)
	GDT-TS	0.41(0.06)	0.29(0.04)	0.29(0.04)	0.28(0.04)	0.25(0.04)	0.28(0.04)
	Q	0.72(0.06)	0.64(0.07)	0.64(0.07)	0.62(0.07)	0.59(0.07)	0.62(0.07)
Rosetta-Baker	RMSD	4.7(2.2)	7.5(3.4)	8.4(4.3)	7.6(4.1)	8.2(2.7)	6.9(3.6)
	MT	6.9(3)	13(7.5)	15(9.2)	13(7.8)	13(6.8)	11(5.4)
	GDT-TS	0.6(0.21)	0.47(0.15)	0.46(0.13)	0.48(0.15)	0.46(0.13)	0.5(0.18)
	Q	0.84(0.08)	0.77(0.1)	0.77(0.11)	0.77(0.01)	0.76(0.07)	0.79(0.09)
Rosetta-Tsai	RMSD	2.8(0.8)	6.9(2.8)	5(1.4)	7.3(1.8)	5.7(2)	6.2(2.1)
	MT	3(1.1)	9.3(4.9)	5.5(2.2)	10(5.8)	8.3(3)	7.1(2.3)
	GDT-TS	0.72(0.08)	0.47(0.08)	0.59(0.09)	0.45(0.06)	0.54(0.09)	0.52(0.12)
	Q	0.86(0.05)	0.77(0.08)	0.81(0.08)	0.74(0.07)	0.77(0.07)	0.77(0.06)
CASP-HRD	RMSD	2(0.56)	2.6(0.73)	2.6(0.71)	2.6(0.76)	2.8(0.7)	2.6(0.74)
	MT	1.1(0.5)	2(0.8)	1.7(0.61)	2(0.83)	2.3(0.11)	1.7(0.79)
	GDT-TS	0.83(0.07)	0.75(0.06)	0.78(0.07)	0.77(0.07)	0.75(0.06)	0.78(0.07)
	Q	0.93(0.03)	0.9(0.03)	0.91(0.03)	0.89(0.04)	0.88(0.04)	0.91(0.03)
CASP10-stage1 e	RMSD	4.7(1.2)	6.6(2.3)	7.1(2.4)	8.2(3.2)	12(4.7)	7.3(2.4)
	MT	4(2)	6.6(2)	8(1.6)	10(3.5)	20(3.4)	7.9(2.2)
	GDT-TS	0.71(0.1)	0.62(0.13)	0.63(0.15)	0.57(0.16)	0.55(0.19)	0.63(0.14)
	Q	0.88(0.04)	0.84(0.04)	0.85(0.04)	0.82(0.06)	0.8(0.06)	0.84(0.06)
CASP10-stage2 e	RMSD	4(1.1)	5.9(1.7)	5.9(1.6)	6.7(1.9)	9(2.2)	6.2(1.6)
	MT	3.1(1)	5.4(1.6)	5.8(1.6)	6.6(1.8)	14(1.9)	5.4(1.5)
	GDT-TS	0.73(0.09)	0.64(0.14)	0.66(0.11)	0.65(0.12)	0.65(0.11)	0.67(0.11)
	Q	0.89(0.04)	0.86(0.03)	0.87(0.04)	0.86(0.05)	0.85(0.04)	0.86(0.04)

a All-atom statistical distance-based potential [49].

b The semi-empirical physical potential AMBER99SB-ILDN [50].

c All-atom orientation-dependent statistical potential [7].

d Average value, and mean absolute deviation (in parenthesis) over the data set.

e Only ensembles who contains a decoy with a *GDT–TS*> 0.4 are included. Compare with Figure 2 in [48].

## Concluding Remarks

The recent literature on generating knowledge-based potentials for protein structure modeling makes no secrets of their limitations and problems. Knowledge-based potentials are energy functions derived primarily from databases of protein structures and sequences. They can be divided into two classes. Potentials from the first class are based on a direct conversion of the distributions of some geometric properties observed in native protein structures into energy values, while potentials from the second class are trained to mimic quantitatively the geometric differences between incorrectly folded models (also called decoys) and native structures. Both potentials are designed to assess how native-like a model structure is. There is no consensus however on which geometric property should be considered, on how to convert a statistical distribution into an energy for the first class, and on how energy and geometry should be related in the second class.

In this paper, we focused on the relationship between energy and geometry when training knowledge-based potentials from the second class. We assumed that the difference between the energy of a decoy and the energy of its corresponding native structure must be linearly related to the distance between the decoy and the native structure. We trained two distance-based C*α* potentials accordingly, one based on all inter-residue distances (PPD), while the other had the set of all these distances filtered to reflect consistency in an ensemble of decoys (PPE). Compared to other methods that follow the same approach however, we did not assume that the distance between a decoy and the native structure is the traditional RMSD. Instead, we tested four different distance measures, two based on extrinsic geometry (RMSD and GTD-TS*), and two based on intrinsic geometry (Q* and MT). We found that it is usually better to train the potentials using the latter type of distances.

We have found that both PPD and PPE perform extremely well on the high resolution decoy set Titan-HRD, with correlation coefficients between energy and distance usually well above 0.8. PPE always performs better than PPD on this set, emphasizing the benefits of capturing consistent information in an ensemble. While we trust the general trends highlighted by these results, we tone down the importance of In extensive testing on available decoy sets and models from the Critical Assessment of protheir exceptional character as they may only reflect the specificity of the Titan-HRD data set. tein Structure Prediction (CASP) experiments we find that PPD yields better energy-distance correlations than one of the state of the art single model potentials, GOAP [7]. We note however that the sophisticated distance-based and orientation-based statistical potential GOAP is better at picking the best decoys and has a better though comparable performance for fixed energy-distance correlation. It should be noted that PPD and PPE are C*α*-based, while GOAP is an all-atom potential. We believe that this demonstrates that a very efficient training of a simple distance-based pair potential can generate a very effective measure for assessing protein structure models.

There is still room for improvement in training knowledge-based potentials. We limited our study to pairwise potentials; we will test different geometric properties of protein structures in future studies. We plan to include the potentials described here into a structure minimization package, to assess their performances in improving non-native protein structure models.

## Supporting Information

File S1 (TXT)

File S2 (TXT)

Force Field S1 (DOC)

Readme Force Field S1 (RTF)
