# Supplementary material for: On the Importance of the Distance Measures Used to Train and Test Knowledge-Based Potentials for Proteins
Source: PLoS One. 2014 Nov 20;9(11):e109335. doi: 10.1371/journal.pone.0109335 (PMC4239004; doi:10.1371/journal.pone.0109335)
Supplement: Force Field S1 — (DOC) [file pone.0109335.s003.doc]

1,1,1,1,1,0.091102

1,1,1,1,2,-0.041141

1,1,1,1,3,-0.015525

1,1,1,1,4,-0.025654

1,1,1,1,5,0.013974

1,1,1,1,6,-0.0011638

1,1,1,1,7,0.0083938

1,1,1,1,8,-0.0090104

1,1,1,2,1,0.019995

1,1,1,2,2,-0.01305

1,1,1,2,3,-0.043062

1,1,1,2,4,0.027601

1,1,1,2,5,0.024036

1,1,1,2,6,0.044776

1,1,1,2,7,0.0041509

1,1,1,2,8,0.014319

1,1,1,3,1,0.12782

1,1,1,3,2,0.0096711

1,1,1,3,3,-0.12714

1,1,1,3,4,-0.036339

1,1,1,3,5,0.017934

1,1,1,3,6,-0.0027757

1,1,1,3,7,0.0072163

1,1,1,3,8,-0.017459

1,1,1,4,1,0.11324

1,1,1,4,2,0.059615

1,1,1,4,3,-0.07883

1,1,1,4,4,0.0024221

1,1,1,4,5,0.02153

1,1,1,4,6,0.020027

1,1,1,4,7,0.0099521

1,1,1,4,8,-0.009433

1,1,1,5,1,0.13759

1,1,1,5,2,-0.037097

1,1,1,5,3,-0.037184

1,1,1,5,4,-0.039953

1,1,1,5,5,0.031236

1,1,1,5,6,0.010535

1,1,1,5,7,0.0079976

1,1,1,5,8,-0.00547

1,1,1,6,1,0.088524

1,1,1,6,2,0.015913

1,1,1,6,3,-0.076667

1,1,1,6,4,-0.045774

1,1,1,6,5,0.040782

1,1,1,6,6,0.0056544

1,1,1,6,7,0.010534

1,1,1,6,8,-0.019528

1,1,1,7,1,0.061859

1,1,1,7,2,0.0076676

1,1,1,7,3,-0.067382

1,1,1,7,4,-0.060251

1,1,1,7,5,0.031096

1,1,1,7,6,0.00035811

1,1,1,7,7,0.011756

1,1,1,7,8,-0.017311

1,1,1,8,1,0.10917

1,1,1,8,2,0.022642

1,1,1,8,3,-0.069565

1,1,1,8,4,-0.0056162

1,1,1,8,5,0.029473

1,1,1,8,6,-0.0066818

1,1,1,8,7,0.011757

1,1,1,8,8,-0.013252

1,1,1,9,1,0.12411

1,1,1,9,2,0.0069237

1,1,1,9,3,-0.075928

1,1,1,9,4,-0.0053713

1,1,1,9,5,0.017375

1,1,1,9,6,0.022958

1,1,1,9,7,0.0022317

1,1,1,9,8,-0.0013614

1,1,1,10,1,0.11617

1,1,1,10,2,-0.03771

1,1,1,10,3,-0.0578

1,1,1,10,4,-0.047846

1,1,1,10,5,0.017759

1,1,1,10,6,0.012005

1,1,1,10,7,0.017035

1,1,1,10,8,-0.017926

1,1,1,11,1,0.13286

1,1,1,11,2,0.065042

1,1,1,11,3,-0.079497

1,1,1,11,4,-0.066804

1,1,1,11,5,0.021147

1,1,1,11,6,-0.002486

1,1,1,11,7,0.014893

1,1,1,11,8,-0.018821

1,1,1,12,1,0.0060607

1,1,1,12,2,-0.052316

1,1,1,12,3,-0.054395

1,1,1,12,4,-0.063825

1,1,1,12,5,0.047383

1,1,1,12,6,-0.0046083

1,1,1,12,7,0.013644

1,1,1,12,8,-0.021071

1,1,1,13,1,0.1751

1,1,1,13,2,0.017716

1,1,1,13,3,-0.043855

1,1,1,13,4,-0.00043935

1,1,1,13,5,0.015633

1,1,1,13,6,0.053033

1,1,1,13,7,-0.0048295

1,1,1,13,8,0.0056917

1,1,1,14,1,0.16877

1,1,1,14,2,0.030914

1,1,1,14,3,-0.065955

1,1,1,14,4,0.012882

1,1,1,14,5,0.029208

1,1,1,14,6,0.033102

1,1,1,14,7,0.0075981

1,1,1,14,8,-0.0068969

1,1,1,15,1,0.075254

1,1,1,15,2,0.018125

1,1,1,15,3,-0.048997

1,1,1,15,4,0.0061084

1,1,1,15,5,0.0051789

1,1,1,15,6,0.030697

1,1,1,15,7,0.0034569

1,1,1,15,8,-0.010578

1,1,1,16,1,0.046917

1,1,1,16,2,0.053097

1,1,1,16,3,-0.04208

1,1,1,16,4,-0.0055475

1,1,1,16,5,0.020198

1,1,1,16,6,0.018859

1,1,1,16,7,-0.000414

1,1,1,16,8,-0.035711

1,1,1,17,1,0.18285

1,1,1,17,2,0.052651

1,1,1,17,3,-0.045321

1,1,1,17,4,-0.0095556

1,1,1,17,5,0.013355

1,1,1,17,6,0.034823

1,1,1,17,7,0.0056214

1,1,1,17,8,-0.011346

1,1,1,18,1,0.11836

1,1,1,18,2,0.058933

1,1,1,18,3,-0.052918

1,1,1,18,4,-0.0174

1,1,1,18,5,0.042551

1,1,1,18,6,0.017462

1,1,1,18,7,0.020574

1,1,1,18,8,-0.0026149

1,1,1,19,1,0.095616

1,1,1,19,2,0.057495

1,1,1,19,3,-0.043308

1,1,1,19,4,-0.016587

1,1,1,19,5,0.015071

1,1,1,19,6,0.024234

1,1,1,19,7,0.020493

1,1,1,19,8,0.0017559

1,1,1,20,1,0.059515

1,1,1,20,2,0.041471

1,1,1,20,3,-0.036699

1,1,1,20,4,0.0074926

1,1,1,20,5,0.0087144

1,1,1,20,6,0.025413

1,1,1,20,7,0.018072

1,1,1,20,8,0.010239

1,1,2,2,1,0.028687

1,1,2,2,2,-0.026404

1,1,2,2,3,0.0073326

1,1,2,2,4,-0.011477

1,1,2,2,5,0.0049168

1,1,2,2,6,-0.012671

1,1,2,2,7,0.0016981

1,1,2,2,8,0.00092585

1,1,2,3,1,0.034946

1,1,2,3,2,0.041284

1,1,2,3,3,-0.051169

1,1,2,3,4,-0.034175

1,1,2,3,5,0.01787

1,1,2,3,6,-0.0013923

1,1,2,3,7,-0.014823

1,1,2,3,8,-0.0061479

1,1,2,4,1,0.004146

1,1,2,4,2,0.085608

1,1,2,4,3,-0.038414

1,1,2,4,4,-0.014582

1,1,2,4,5,0.0028973

1,1,2,4,6,-0.007725

1,1,2,4,7,0.0094067

1,1,2,4,8,-0.019119

1,1,2,5,1,0.034067

1,1,2,5,2,0.0077424

1,1,2,5,3,-0.046945

1,1,2,5,4,-0.027399

1,1,2,5,5,0.0018569

1,1,2,5,6,-0.008868

1,1,2,5,7,-0.0044145

1,1,2,5,8,-0.011106

1,1,2,6,1,0.03203

1,1,2,6,2,-0.019655

1,1,2,6,3,-0.039163

1,1,2,6,4,-0.0070364

1,1,2,6,5,1.7208e-05

1,1,2,6,6,0.017328

1,1,2,6,7,0.0022362

1,1,2,6,8,-0.01231

1,1,2,7,1,1.8282e-06

1,1,2,7,2,0.037934

1,1,2,7,3,-0.044045

1,1,2,7,4,-0.057825

1,1,2,7,5,-0.0044219

1,1,2,7,6,0.0019744

1,1,2,7,7,-0.012985

1,1,2,7,8,-0.010932

1,1,2,8,1,0.064329

1,1,2,8,2,0.028348

1,1,2,8,3,-0.07801

1,1,2,8,4,-0.024044

1,1,2,8,5,0.0031403

1,1,2,8,6,-0.017496

1,1,2,8,7,0.0045635

1,1,2,8,8,-0.0045206

1,1,2,9,1,0.021633

1,1,2,9,2,0.016589

1,1,2,9,3,-0.040155

1,1,2,9,4,0.0051458

1,1,2,9,5,-0.0080854

1,1,2,9,6,0.014098

1,1,2,9,7,0.015327

1,1,2,9,8,-0.011568

1,1,2,10,1,4.3892e-11

1,1,2,10,2,0.063315

1,1,2,10,3,-0.0099981

1,1,2,10,4,-0.042415

1,1,2,10,5,0.030691

1,1,2,10,6,-0.023587

1,1,2,10,7,0.020328

1,1,2,10,8,-0.0097558

1,1,2,11,1,0.0099215

1,1,2,11,2,-0.022428

1,1,2,11,3,-0.062762

1,1,2,11,4,-0.030263

1,1,2,11,5,0.0049701

1,1,2,11,6,-0.010032

1,1,2,11,7,0.012308

1,1,2,11,8,-0.028626

1,1,2,12,1,0.011516

1,1,2,12,2,0.016509

1,1,2,12,3,-0.027193

1,1,2,12,4,-0.067112

1,1,2,12,5,-0.016043

1,1,2,12,6,-0.0099252

1,1,2,12,7,-0.022566

1,1,2,12,8,-0.019434

1,1,2,13,1,0.03613

1,1,2,13,2,-0.031812

1,1,2,13,3,-0.048693

1,1,2,13,4,-0.040143

1,1,2,13,5,0.015302

1,1,2,13,6,0.0071775

1,1,2,13,7,0.014643

1,1,2,13,8,-0.0041124

1,1,2,14,1,0.017076

1,1,2,14,2,-0.013124

1,1,2,14,3,-0.045387

1,1,2,14,4,-0.016553

1,1,2,14,5,-0.0064319

1,1,2,14,6,0.018834

1,1,2,14,7,-0.013176

1,1,2,14,8,0.0011263

1,1,2,15,1,0.025913

1,1,2,15,2,0.0083201

1,1,2,15,3,-0.036928

1,1,2,15,4,-0.025851

1,1,2,15,5,0.01551

1,1,2,15,6,0.01502

1,1,2,15,7,0.0036063

1,1,2,15,8,-0.0022686

1,1,2,16,1,0.028503

1,1,2,16,2,0.013383

1,1,2,16,3,-0.040352

1,1,2,16,4,0.033635

1,1,2,16,5,-0.0009144

1,1,2,16,6,0.0070221

1,1,2,16,7,0.0040017

1,1,2,16,8,-0.0013508

1,1,2,17,1,0.035442

1,1,2,17,2,0.036756

1,1,2,17,3,0.034759

1,1,2,17,4,-0.02441

1,1,2,17,5,0.027891

1,1,2,17,6,0.0079514

1,1,2,17,7,0.022353

1,1,2,17,8,-0.0089949

1,1,2,18,1,3.608e-09

1,1,2,18,2,0.033778

1,1,2,18,3,-0.0052784

1,1,2,18,4,-0.01992

1,1,2,18,5,0.0060925

1,1,2,18,6,0.0063547

1,1,2,18,7,0.0094993

1,1,2,18,8,-0.010105

1,1,2,19,1,1.3667e-11

1,1,2,19,2,0.040154

1,1,2,19,3,-0.037509

1,1,2,19,4,-3.9007e-05

1,1,2,19,5,-0.00070025

1,1,2,19,6,-0.002519

1,1,2,19,7,0.010513

1,1,2,19,8,-0.007676

1,1,2,20,1,3.5429e-11

1,1,2,20,2,0.040765

1,1,2,20,3,-0.024955

1,1,2,20,4,-0.027546

1,1,2,20,5,-0.034298

1,1,2,20,6,-0.0074582

1,1,2,20,7,-0.028667

1,1,2,20,8,-0.00011826

1,1,3,3,1,0.068063

1,1,3,3,2,0.045296

1,1,3,3,3,-0.036013

1,1,3,3,4,-0.025521

1,1,3,3,5,0.020827

1,1,3,3,6,0.014075

1,1,3,3,7,-0.002975

1,1,3,3,8,-0.0082113

1,1,3,4,1,0.14687

1,1,3,4,2,0.044625

1,1,3,4,3,-0.035099

1,1,3,4,4,-0.0052646

1,1,3,4,5,0.054161

1,1,3,4,6,0.019062

1,1,3,4,7,0.040294

1,1,3,4,8,-0.0055272

1,1,3,5,1,0.12972

1,1,3,5,2,0.059789

1,1,3,5,3,-0.069843

1,1,3,5,4,-0.034472

1,1,3,5,5,0.031892

1,1,3,5,6,0.010122

1,1,3,5,7,0.0039059

1,1,3,5,8,-0.014393

1,1,3,6,1,0.071785

1,1,3,6,2,0.08979

1,1,3,6,3,-0.12123

1,1,3,6,4,-0.064653

1,1,3,6,5,0.037346

1,1,3,6,6,0.0054337

1,1,3,6,7,0.018434

1,1,3,6,8,-0.011887

1,1,3,7,1,0.088229

1,1,3,7,2,0.029352

1,1,3,7,3,-0.022444

1,1,3,7,4,-0.035085

1,1,3,7,5,0.041051

1,1,3,7,6,0.028123

1,1,3,7,7,0.016717

1,1,3,7,8,-0.0044468

1,1,3,8,1,0.14373

1,1,3,8,2,0.041347

1,1,3,8,3,-0.18169

1,1,3,8,4,-0.05788

1,1,3,8,5,-0.0073049

1,1,3,8,6,-0.0079636

1,1,3,8,7,0.0081734

1,1,3,8,8,-0.029492

1,1,3,9,1,0.11239

1,1,3,9,2,0.058352

1,1,3,9,3,-0.054581

1,1,3,9,4,-0.032545

1,1,3,9,5,0.019409

1,1,3,9,6,0.02723

1,1,3,9,7,0.029473

1,1,3,9,8,-0.0068114

1,1,3,10,1,0.11901

1,1,3,10,2,-0.029779

1,1,3,10,3,-0.14194

1,1,3,10,4,-0.086469

1,1,3,10,5,-0.0012958

1,1,3,10,6,-0.024171

1,1,3,10,7,-0.0050469

1,1,3,10,8,-0.033622

1,1,3,11,1,0.10188

1,1,3,11,2,0.020373

1,1,3,11,3,-0.11642

1,1,3,11,4,-0.044667

1,1,3,11,5,-0.0021115

1,1,3,11,6,-0.018678

1,1,3,11,7,-0.016211

1,1,3,11,8,-0.03431

1,1,3,12,1,0.050533

1,1,3,12,2,0.037299

1,1,3,12,3,-0.074869

1,1,3,12,4,-0.047712

1,1,3,12,5,0.010789

1,1,3,12,6,-0.0018879

1,1,3,12,7,0.0014408

1,1,3,12,8,-0.027417

1,1,3,13,1,0.14371

1,1,3,13,2,0.087167

1,1,3,13,3,0.0048163

1,1,3,13,4,-0.0061101

1,1,3,13,5,0.042389

1,1,3,13,6,0.043417

1,1,3,13,7,0.01404

1,1,3,13,8,-0.0060745

1,1,3,14,1,0.11974

1,1,3,14,2,0.081083

1,1,3,14,3,-0.032649

1,1,3,14,4,-0.012431

1,1,3,14,5,0.060196

1,1,3,14,6,0.0094853

1,1,3,14,7,0.029134

1,1,3,14,8,-0.01764

1,1,3,15,1,0.040485

1,1,3,15,2,0.0096639

1,1,3,15,3,-0.064679

1,1,3,15,4,-0.044512

1,1,3,15,5,0.030928

1,1,3,15,6,-0.0084922

1,1,3,15,7,0.024199

1,1,3,15,8,-0.027311

1,1,3,16,1,0.041271

1,1,3,16,2,0.051698

1,1,3,16,3,-0.062437

1,1,3,16,4,-0.02831

1,1,3,16,5,0.049574

1,1,3,16,6,0.010349

1,1,3,16,7,0.0036659

1,1,3,16,8,0.0016793

1,1,3,17,1,0.17903

1,1,3,17,2,0.12861

1,1,3,17,3,-0.038951

1,1,3,17,4,-0.007771

1,1,3,17,5,0.034104

1,1,3,17,6,0.032315

1,1,3,17,7,0.020437

1,1,3,17,8,-0.014101

1,1,3,18,1,0.085065

1,1,3,18,2,0.039916

1,1,3,18,3,-0.052937

1,1,3,18,4,-0.040587

1,1,3,18,5,0.056166

1,1,3,18,6,0.00075797

1,1,3,18,7,0.012319

1,1,3,18,8,-0.010567

1,1,3,19,1,0.057755

1,1,3,19,2,0.028207

1,1,3,19,3,-0.010644

1,1,3,19,4,-0.047356

1,1,3,19,5,0.026212

1,1,3,19,6,-0.008465

1,1,3,19,7,-0.013383

1,1,3,19,8,-0.025065

1,1,3,20,1,0.028195

1,1,3,20,2,0.013394

1,1,3,20,3,-0.0065782

1,1,3,20,4,-0.041943

1,1,3,20,5,0.032202

1,1,3,20,6,0.0010149

1,1,3,20,7,0.028934

1,1,3,20,8,-0.030906

1,1,4,4,1,0.13954

1,1,4,4,2,-0.0081694

1,1,4,4,3,0.015847

1,1,4,4,4,-0.0029506

1,1,4,4,5,0.0045682

1,1,4,4,6,0.0072856

1,1,4,4,7,0.00083594

1,1,4,4,8,-0.0010007

1,1,4,5,1,0.15768

1,1,4,5,2,0.028313

1,1,4,5,3,-0.0025951

1,1,4,5,4,-0.053772

1,1,4,5,5,0.02102

1,1,4,5,6,-0.0026326

1,1,4,5,7,0.015592

1,1,4,5,8,-0.01323

1,1,4,6,1,0.13676

1,1,4,6,2,0.045998

1,1,4,6,3,-0.023959

1,1,4,6,4,-0.040636

1,1,4,6,5,0.03207

1,1,4,6,6,0.013043

1,1,4,6,7,0.02346

1,1,4,6,8,-0.016571

1,1,4,7,1,0.094956

1,1,4,7,2,0.058067

1,1,4,7,3,-0.038681

1,1,4,7,4,-0.038953

1,1,4,7,5,0.014966

1,1,4,7,6,0.014507

1,1,4,7,7,0.0027455

1,1,4,7,8,-0.014212

1,1,4,8,1,0.10731

1,1,4,8,2,0.029132

1,1,4,8,3,-0.091856

1,1,4,8,4,-0.030574

1,1,4,8,5,0.0031233

1,1,4,8,6,0.0079456

1,1,4,8,7,0.0025411

1,1,4,8,8,0.0031714

1,1,4,9,1,0.11835

1,1,4,9,2,0.033559

1,1,4,9,3,-0.029581

1,1,4,9,4,-0.0020149

1,1,4,9,5,0.0036772

1,1,4,9,6,0.018539

1,1,4,9,7,0.0064909

1,1,4,9,8,0.0084358

1,1,4,10,1,0.15556

1,1,4,10,2,-0.020867

1,1,4,10,3,-0.078227

1,1,4,10,4,-0.074174

1,1,4,10,5,-0.0054311

1,1,4,10,6,0.00047309

1,1,4,10,7,0.0055123

1,1,4,10,8,-0.0060072

1,1,4,11,1,0.079534

1,1,4,11,2,0.048878

1,1,4,11,3,-0.097752

1,1,4,11,4,-0.051023

1,1,4,11,5,-0.014719

1,1,4,11,6,-0.0054294

1,1,4,11,7,0.0011918

1,1,4,11,8,-0.020485

1,1,4,12,1,0.059586

1,1,4,12,2,0.05094

1,1,4,12,3,-0.013147

1,1,4,12,4,-0.040849

1,1,4,12,5,0.028641

1,1,4,12,6,-0.0076951

1,1,4,12,7,0.033792

1,1,4,12,8,-0.025312

1,1,4,13,1,0.15269

1,1,4,13,2,0.018273

1,1,4,13,3,-0.041098

1,1,4,13,4,-0.059617

1,1,4,13,5,0.0060643

1,1,4,13,6,0.010503

1,1,4,13,7,0.0047526

1,1,4,13,8,-0.0091577

1,1,4,14,1,0.15122

1,1,4,14,2,0.024883

1,1,4,14,3,-0.016516

1,1,4,14,4,-0.027358

1,1,4,14,5,0.0091767

1,1,4,14,6,0.00044314

1,1,4,14,7,0.0041469

1,1,4,14,8,-0.024273

1,1,4,15,1,0.056682

1,1,4,15,2,0.00047627

1,1,4,15,3,0.01267

1,1,4,15,4,-0.052138

1,1,4,15,5,0.021953

1,1,4,15,6,0.012894

1,1,4,15,7,0.026332

1,1,4,15,8,-0.0019256

1,1,4,16,1,0.012497

1,1,4,16,2,0.0064384

1,1,4,16,3,-0.0068671

1,1,4,16,4,0.030837

1,1,4,16,5,0.0098855

1,1,4,16,6,0.018093

1,1,4,16,7,-0.0038983

1,1,4,16,8,-0.01941

1,1,4,17,1,0.20154

1,1,4,17,2,0.078738

1,1,4,17,3,-0.0081295

1,1,4,17,4,-0.030295

1,1,4,17,5,0.024817

1,1,4,17,6,0.0029745

1,1,4,17,7,0.0043634

1,1,4,17,8,-0.0083298

1,1,4,18,1,0.096939

1,1,4,18,2,0.024748

1,1,4,18,3,-0.024039

1,1,4,18,4,-0.035368

1,1,4,18,5,0.017443

1,1,4,18,6,-0.017084

1,1,4,18,7,0.0081505

1,1,4,18,8,-0.015526

1,1,4,19,1,0.079851

1,1,4,19,2,0.064412

1,1,4,19,3,-0.010874

1,1,4,19,4,-0.0046995

1,1,4,19,5,0.022521

1,1,4,19,6,-0.010232

1,1,4,19,7,0.0032898

1,1,4,19,8,-0.025871

1,1,4,20,1,0.038117

1,1,4,20,2,0.0057078

1,1,4,20,3,-0.0062407

1,1,4,20,4,-0.026827

1,1,4,20,5,0.0089471

1,1,4,20,6,-0.006232

1,1,4,20,7,-0.0011542

1,1,4,20,8,-0.016984

1,1,5,5,1,0.20613

1,1,5,5,2,-0.0090194

1,1,5,5,3,0.0055506

1,1,5,5,4,-0.033915

1,1,5,5,5,0.018118

1,1,5,5,6,-0.010192

1,1,5,5,7,0.0064079

1,1,5,5,8,-0.0089861

1,1,5,6,1,0.1175

1,1,5,6,2,0.052527

1,1,5,6,3,-0.0578

1,1,5,6,4,-0.041216

1,1,5,6,5,0.035691

1,1,5,6,6,0.0016841

1,1,5,6,7,0.015842

1,1,5,6,8,-0.02097

1,1,5,7,1,0.13025

1,1,5,7,2,0.076841

1,1,5,7,3,-0.079472

1,1,5,7,4,-0.068581

1,1,5,7,5,0.016406

1,1,5,7,6,0.0062484

1,1,5,7,7,0.0096975

1,1,5,7,8,-0.024682

1,1,5,8,1,0.11634

1,1,5,8,2,0.041596

1,1,5,8,3,-0.073087

1,1,5,8,4,-0.049018

1,1,5,8,5,0.028054

1,1,5,8,6,0.010653

1,1,5,8,7,-0.00096953

1,1,5,8,8,-0.010573

1,1,5,9,1,0.1325

1,1,5,9,2,0.05748

1,1,5,9,3,-0.032962

1,1,5,9,4,-0.02304

1,1,5,9,5,0.015512

1,1,5,9,6,0.014288

1,1,5,9,7,0.0052991

1,1,5,9,8,0.0038301

1,1,5,10,1,0.18482

1,1,5,10,2,0.078317

1,1,5,10,3,-0.066081

1,1,5,10,4,-0.061129

1,1,5,10,5,0.02327

1,1,5,10,6,-0.0063725

1,1,5,10,7,-0.003477

1,1,5,10,8,-0.02901

1,1,5,11,1,0.094232

1,1,5,11,2,0.068146

1,1,5,11,3,-0.06961

1,1,5,11,4,-0.039246

1,1,5,11,5,0.016104

1,1,5,11,6,0.0074645

1,1,5,11,7,0.011682

1,1,5,11,8,-0.0054022

1,1,5,12,1,0.075046

1,1,5,12,2,0.039996

1,1,5,12,3,-0.050677

1,1,5,12,4,-0.028507

1,1,5,12,5,0.036901

1,1,5,12,6,-0.0025742

1,1,5,12,7,0.0069444

1,1,5,12,8,-0.0042693

1,1,5,13,1,0.22451

1,1,5,13,2,0.034295

1,1,5,13,3,-0.048181

1,1,5,13,4,-0.024169

1,1,5,13,5,0.0014403

1,1,5,13,6,0.017865

1,1,5,13,7,0.0083718

1,1,5,13,8,-0.01109

1,1,5,14,1,0.15255

1,1,5,14,2,0.011357

1,1,5,14,3,-0.078475

1,1,5,14,4,-0.040417

1,1,5,14,5,-0.025733

1,1,5,14,6,-0.0030802

1,1,5,14,7,-0.0024299

1,1,5,14,8,-0.012028

1,1,5,15,1,0.060252

1,1,5,15,2,-0.0021688

1,1,5,15,3,-0.032038

1,1,5,15,4,-0.036959

1,1,5,15,5,-0.0021378

1,1,5,15,6,-0.012588

1,1,5,15,7,-0.0066834

1,1,5,15,8,-0.0091323

1,1,5,16,1,0.032509

1,1,5,16,2,-0.0030398

1,1,5,16,3,0.014415

1,1,5,16,4,0.024969

1,1,5,16,5,0.030803

1,1,5,16,6,0.0026317

1,1,5,16,7,0.023425

1,1,5,16,8,-0.0029692

1,1,5,17,1,0.23864

1,1,5,17,2,0.084345

1,1,5,17,3,-0.018468

1,1,5,17,4,-0.059754

1,1,5,17,5,-0.0044409

1,1,5,17,6,-0.014604

1,1,5,17,7,0.0062161

1,1,5,17,8,-0.015615

1,1,5,18,1,0.072105

1,1,5,18,2,0.050898

1,1,5,18,3,-0.050152

1,1,5,18,4,-0.055692

1,1,5,18,5,0.003324

1,1,5,18,6,-0.033326

1,1,5,18,7,-0.0061474

1,1,5,18,8,-0.0321

1,1,5,19,1,0.060887

1,1,5,19,2,0.042871

1,1,5,19,3,-0.043213

1,1,5,19,4,-0.050498

1,1,5,19,5,0.012941

1,1,5,19,6,-0.013869

1,1,5,19,7,-0.0034347

1,1,5,19,8,-0.022318

1,1,5,20,1,0.041935

1,1,5,20,2,0.043384

1,1,5,20,3,-0.039444

1,1,5,20,4,-0.035747

1,1,5,20,5,-0.024639

1,1,5,20,6,-0.011483

1,1,5,20,7,-0.015457

1,1,5,20,8,-0.030179

1,1,6,6,1,0.08527

1,1,6,6,2,0.052965

1,1,6,6,3,-0.065704

1,1,6,6,4,-0.035904

1,1,6,6,5,-0.0059764

1,1,6,6,6,-0.015304

1,1,6,6,7,0.013276

1,1,6,6,8,-0.015053

1,1,6,7,1,0.076749

1,1,6,7,2,0.039535

1,1,6,7,3,-0.082064

1,1,6,7,4,-0.071903

1,1,6,7,5,0.042823

1,1,6,7,6,-0.0087948

1,1,6,7,7,-0.0056939

1,1,6,7,8,-0.010828

1,1,6,8,1,0.091091

1,1,6,8,2,0.040381

1,1,6,8,3,-0.11477

1,1,6,8,4,-0.064359

1,1,6,8,5,0.0046873

1,1,6,8,6,-0.0098609

1,1,6,8,7,0.0055696

1,1,6,8,8,-0.022354

1,1,6,9,1,0.098536

1,1,6,9,2,0.061507

1,1,6,9,3,-0.079703

1,1,6,9,4,-0.012536

1,1,6,9,5,-0.0026669

1,1,6,9,6,0.031924

1,1,6,9,7,-0.012684

1,1,6,9,8,0.0061278

1,1,6,10,1,0.087731

1,1,6,10,2,0.0083677

1,1,6,10,3,-0.092154

1,1,6,10,4,-0.055097

1,1,6,10,5,0.062251

1,1,6,10,6,-0.0026708

1,1,6,10,7,0.025274

1,1,6,10,8,-0.027055

1,1,6,11,1,0.083395

1,1,6,11,2,0.024732

1,1,6,11,3,-0.091097

1,1,6,11,4,-0.060692

1,1,6,11,5,0.021259

1,1,6,11,6,0.0072135

1,1,6,11,7,0.0057495

1,1,6,11,8,-0.013731

1,1,6,12,1,0.032558

1,1,6,12,2,0.072696

1,1,6,12,3,-0.020204

1,1,6,12,4,-0.077636

1,1,6,12,5,0.0073058

1,1,6,12,6,-0.0012984

1,1,6,12,7,0.012319

1,1,6,12,8,-0.018456

1,1,6,13,1,0.12253

1,1,6,13,2,0.039712

1,1,6,13,3,0.0014169

1,1,6,13,4,-0.019952

1,1,6,13,5,0.026418

1,1,6,13,6,0.043064

1,1,6,13,7,0.035876

1,1,6,13,8,-0.009755

1,1,6,14,1,0.10559

1,1,6,14,2,0.050631

1,1,6,14,3,-0.010765

1,1,6,14,4,-0.035555

1,1,6,14,5,0.035938

1,1,6,14,6,0.0066494

1,1,6,14,7,0.016683

1,1,6,14,8,-0.019389

1,1,6,15,1,0.046701

1,1,6,15,2,0.034874

1,1,6,15,3,-0.044801

1,1,6,15,4,-0.030652

1,1,6,15,5,0.057405

1,1,6,15,6,-0.0011863

1,1,6,15,7,-0.010663

1,1,6,15,8,-0.039847

1,1,6,16,1,0.0214

1,1,6,16,2,0.042378

1,1,6,16,3,-0.021864

1,1,6,16,4,-0.0051219

1,1,6,16,5,0.014464

1,1,6,16,6,0.0028642

1,1,6,16,7,0.02685

1,1,6,16,8,-0.018448

1,1,6,17,1,0.12259

1,1,6,17,2,0.073364

1,1,6,17,3,-0.014516

1,1,6,17,4,-0.047838

1,1,6,17,5,0.05241

1,1,6,17,6,0.019455

1,1,6,17,7,0.016365

1,1,6,17,8,-0.0052703

1,1,6,18,1,0.074982

1,1,6,18,2,0.068984

1,1,6,18,3,-0.027153

1,1,6,18,4,-0.048595

1,1,6,18,5,0.02378

1,1,6,18,6,0.018303

1,1,6,18,7,0.012896

1,1,6,18,8,-0.0082004

1,1,6,19,1,0.068536

1,1,6,19,2,0.011259

1,1,6,19,3,-0.010654

1,1,6,19,4,-0.031822

1,1,6,19,5,0.018848

1,1,6,19,6,0.013843

1,1,6,19,7,0.007976

1,1,6,19,8,-0.008027

1,1,6,20,1,0.028996

1,1,6,20,2,0.01984

1,1,6,20,3,-0.015968

1,1,6,20,4,-0.060255

1,1,6,20,5,0.023745

1,1,6,20,6,0.035821

1,1,6,20,7,0.00046618

1,1,6,20,8,-0.0084262

1,1,7,7,1,0.052331

1,1,7,7,2,0.020234

1,1,7,7,3,-0.00612

1,1,7,7,4,-0.047718

1,1,7,7,5,0.0032102

1,1,7,7,6,0.019178

1,1,7,7,7,-0.0078023

1,1,7,7,8,-0.010387

1,1,7,8,1,0.091215

1,1,7,8,2,0.023791

1,1,7,8,3,-0.07319

1,1,7,8,4,-0.043705

1,1,7,8,5,0.0089268

1,1,7,8,6,0.0093872

1,1,7,8,7,0.009754

1,1,7,8,8,-0.020805

1,1,7,9,1,0.051738

1,1,7,9,2,0.036627

1,1,7,9,3,-0.12011

1,1,7,9,4,-0.049888

1,1,7,9,5,-0.0092473

1,1,7,9,6,0.01696

1,1,7,9,7,0.011284

1,1,7,9,8,-0.023951

1,1,7,10,1,0.10105

1,1,7,10,2,0.027123

1,1,7,10,3,-0.07223

1,1,7,10,4,-0.029789

1,1,7,10,5,0.013658

1,1,7,10,6,0.027262

1,1,7,10,7,0.010342

1,1,7,10,8,-0.0026257

1,1,7,11,1,0.043253

1,1,7,11,2,0.01183

1,1,7,11,3,-0.042213

1,1,7,11,4,-0.049277

1,1,7,11,5,0.0038739

1,1,7,11,6,-0.0031987

1,1,7,11,7,0.022479

1,1,7,11,8,-0.013688

1,1,7,12,1,0.027513

1,1,7,12,2,0.0041185

1,1,7,12,3,-0.016134

1,1,7,12,4,-0.050991

1,1,7,12,5,0.042482

1,1,7,12,6,0.039996

1,1,7,12,7,-0.0087309

1,1,7,12,8,-0.015733

1,1,7,13,1,0.11115

1,1,7,13,2,0.036595

1,1,7,13,3,-0.04764

1,1,7,13,4,-0.030912

1,1,7,13,5,0.02784

1,1,7,13,6,0.036614

1,1,7,13,7,0.02814

1,1,7,13,8,-0.016464

1,1,7,14,1,0.086469

1,1,7,14,2,0.014371

1,1,7,14,3,-0.032251

1,1,7,14,4,-0.08245

1,1,7,14,5,0.010752

1,1,7,14,6,0.01526

1,1,7,14,7,-0.00040223

1,1,7,14,8,-0.021573

1,1,7,15,1,0.032475

1,1,7,15,2,0.030648

1,1,7,15,3,-0.045544

1,1,7,15,4,-0.042154

1,1,7,15,5,0.013612

1,1,7,15,6,0.0018018

1,1,7,15,7,0.010468

1,1,7,15,8,-0.010375

1,1,7,16,1,0.034149

1,1,7,16,2,-0.01181

1,1,7,16,3,-0.043597

1,1,7,16,4,-0.023764

1,1,7,16,5,-0.0094588

1,1,7,16,6,0.037685

1,1,7,16,7,-0.0086238

1,1,7,16,8,-0.008797

1,1,7,17,1,0.13061

1,1,7,17,2,0.078405

1,1,7,17,3,-0.047335

1,1,7,17,4,-0.043315

1,1,7,17,5,0.03245

1,1,7,17,6,-0.0050852

1,1,7,17,7,0.020039

1,1,7,17,8,-0.032765

1,1,7,18,1,0.056453

1,1,7,18,2,0.029219

1,1,7,18,3,-0.042047

1,1,7,18,4,-0.053719

1,1,7,18,5,-0.002676

1,1,7,18,6,0.033013

1,1,7,18,7,0.0056253

1,1,7,18,8,-0.0098205

1,1,7,19,1,0.06834

1,1,7,19,2,0.034576

1,1,7,19,3,-0.058374

1,1,7,19,4,-0.064357

1,1,7,19,5,0.0050425

1,1,7,19,6,-0.025668

1,1,7,19,7,-0.0032873

1,1,7,19,8,-0.020279

1,1,7,20,1,0.013336

1,1,7,20,2,-0.028565

1,1,7,20,3,-0.034468

1,1,7,20,4,-0.08896

1,1,7,20,5,0.01184

1,1,7,20,6,0.0064118

1,1,7,20,7,-0.010755

1,1,7,20,8,-0.019957

1,1,8,8,1,0.14232

1,1,8,8,2,0.084932

1,1,8,8,3,-0.043653

1,1,8,8,4,-0.003344

1,1,8,8,5,0.011692

1,1,8,8,6,0.012094

1,1,8,8,7,0.011525

1,1,8,8,8,-0.0054187

1,1,8,9,1,0.12849

1,1,8,9,2,-0.024888

1,1,8,9,3,-0.076275

1,1,8,9,4,-0.0034685

1,1,8,9,5,0.014957

1,1,8,9,6,0.00069101

1,1,8,9,7,0.014086

1,1,8,9,8,-0.017916

1,1,8,10,1,0.13298

1,1,8,10,2,-0.0035557

1,1,8,10,3,-0.059665

1,1,8,10,4,-0.027656

1,1,8,10,5,0.0073008

1,1,8,10,6,0.019642

1,1,8,10,7,0.0042732

1,1,8,10,8,-0.01049

1,1,8,11,1,0.10359

1,1,8,11,2,0.021628

1,1,8,11,3,-0.10973

1,1,8,11,4,-0.058884

1,1,8,11,5,0.017355

1,1,8,11,6,-0.0047923

1,1,8,11,7,0.010264

1,1,8,11,8,-0.013732

1,1,8,12,1,0.078234

1,1,8,12,2,0.063238

1,1,8,12,3,-0.053901

1,1,8,12,4,-0.044276

1,1,8,12,5,0.030467

1,1,8,12,6,0.017886

1,1,8,12,7,0.012355

1,1,8,12,8,-0.006556

1,1,8,13,1,0.14338

1,1,8,13,2,0.016827

1,1,8,13,3,-0.053432

1,1,8,13,4,-0.029483

1,1,8,13,5,0.020063

1,1,8,13,6,0.018445

1,1,8,13,7,0.011666

1,1,8,13,8,-0.015651

1,1,8,14,1,0.11531

1,1,8,14,2,0.028299

1,1,8,14,3,-0.040842

1,1,8,14,4,-0.02525

1,1,8,14,5,0.043974

1,1,8,14,6,0.025352

1,1,8,14,7,0.013336

1,1,8,14,8,-0.020351

1,1,8,15,1,0.043785

1,1,8,15,2,0.0048296

1,1,8,15,3,-0.080122

1,1,8,15,4,-0.047772

1,1,8,15,5,0.0033405

1,1,8,15,6,-0.0025261

1,1,8,15,7,-0.0073057

1,1,8,15,8,-0.005126

1,1,8,16,1,0.046595

1,1,8,16,2,0.033544

1,1,8,16,3,-0.016236

1,1,8,16,4,0.0040825

1,1,8,16,5,0.02479

1,1,8,16,6,-0.0099206

1,1,8,16,7,0.018028

1,1,8,16,8,-0.019354

1,1,8,17,1,0.15248

1,1,8,17,2,0.078775

1,1,8,17,3,-0.020375

1,1,8,17,4,-0.021698

1,1,8,17,5,0.048207

1,1,8,17,6,0.018557

1,1,8,17,7,0.019276

1,1,8,17,8,-0.002223

1,1,8,18,1,0.10279

1,1,8,18,2,0.04801

1,1,8,18,3,-0.033736

1,1,8,18,4,-0.032602

1,1,8,18,5,0.062364

1,1,8,18,6,-0.0044835

1,1,8,18,7,0.01978

1,1,8,18,8,-0.024858

1,1,8,19,1,0.058646

1,1,8,19,2,0.0087484

1,1,8,19,3,-0.076794

1,1,8,19,4,-0.010244

1,1,8,19,5,0.024869

1,1,8,19,6,0.02402

1,1,8,19,7,0.006366

1,1,8,19,8,-0.020492

1,1,8,20,1,0.034208

1,1,8,20,2,0.049079

1,1,8,20,3,-0.031067

1,1,8,20,4,-0.044747

1,1,8,20,5,0.030161

1,1,8,20,6,-0.0043131

1,1,8,20,7,0.0058783

1,1,8,20,8,-0.0366

1,1,9,9,1,0.10925

1,1,9,9,2,-0.019644

1,1,9,9,3,-0.0029015

1,1,9,9,4,-0.012409

1,1,9,9,5,-0.0043213

1,1,9,9,6,-0.0051982

1,1,9,9,7,0.012307

1,1,9,9,8,-0.011032

1,1,9,10,1,0.094417

1,1,9,10,2,0.023826

1,1,9,10,3,-0.03247

1,1,9,10,4,-0.022233

1,1,9,10,5,0.0067837

1,1,9,10,6,0.023287

1,1,9,10,7,-0.0039809

1,1,9,10,8,-0.001899

1,1,9,11,1,0.10995

1,1,9,11,2,0.024358

1,1,9,11,3,-0.063239

1,1,9,11,4,-0.041605

1,1,9,11,5,-0.011294

1,1,9,11,6,0.013073

1,1,9,11,7,-0.011119

1,1,9,11,8,-0.0042435

1,1,9,12,1,0.056653

1,1,9,12,2,0.043754

1,1,9,12,3,-0.068843

1,1,9,12,4,0.010794

1,1,9,12,5,0.014005

1,1,9,12,6,0.014348

1,1,9,12,7,0.0039729

1,1,9,12,8,-0.0095408

1,1,9,13,1,0.15921

1,1,9,13,2,0.052477

1,1,9,13,3,-0.008094

1,1,9,13,4,0.0025695

1,1,9,13,5,0.016936

1,1,9,13,6,0.0086276

1,1,9,13,7,0.0057587

1,1,9,13,8,0.0024014

1,1,9,14,1,0.12221

1,1,9,14,2,-0.035286

1,1,9,14,3,-0.036311

1,1,9,14,4,-0.046586

1,1,9,14,5,0.0073372

1,1,9,14,6,-0.008622

1,1,9,14,7,-0.0053518

1,1,9,14,8,-0.01969

1,1,9,15,1,0.039333

1,1,9,15,2,0.059344

1,1,9,15,3,-0.034866

1,1,9,15,4,-0.020396

1,1,9,15,5,0.023768

1,1,9,15,6,-0.0075058

1,1,9,15,7,0.02562

1,1,9,15,8,-0.0058839

1,1,9,16,1,0.065251

1,1,9,16,2,-0.042918

1,1,9,16,3,-0.031425

1,1,9,16,4,0.010377

1,1,9,16,5,-0.005661

1,1,9,16,6,0.017172

1,1,9,16,7,-0.0021373

1,1,9,16,8,0.026418

1,1,9,17,1,0.19508

1,1,9,17,2,0.058598

1,1,9,17,3,-0.02662

1,1,9,17,4,-0.03438

1,1,9,17,5,0.0072375

1,1,9,17,6,-0.0070854

1,1,9,17,7,0.0065394

1,1,9,17,8,0.0019502

1,1,9,18,1,0.066097

1,1,9,18,2,0.030472

1,1,9,18,3,-0.028413

1,1,9,18,4,-0.043706

1,1,9,18,5,0.02222

1,1,9,18,6,-0.012817

1,1,9,18,7,0.010645

1,1,9,18,8,-0.0074713

1,1,9,19,1,0.055987

1,1,9,19,2,0.023601

1,1,9,19,3,-0.034632

1,1,9,19,4,-0.0017344

1,1,9,19,5,0.034902

1,1,9,19,6,-0.0045858

1,1,9,19,7,0.016625

1,1,9,19,8,-0.0086956

1,1,9,20,1,0.023689

1,1,9,20,2,0.016118

1,1,9,20,3,-0.0086494

1,1,9,20,4,-0.021357

1,1,9,20,5,-0.0029294

1,1,9,20,6,-0.021518

1,1,9,20,7,-0.0024404

1,1,9,20,8,-0.0015266

1,1,10,10,1,0.12296

1,1,10,10,2,-0.057738

1,1,10,10,3,-0.0081547

1,1,10,10,4,-0.0030913

1,1,10,10,5,0.021742

1,1,10,10,6,0.0092597

1,1,10,10,7,0.005718

1,1,10,10,8,0.003989

1,1,10,11,1,0.10927

1,1,10,11,2,0.069992

1,1,10,11,3,0.034684

1,1,10,11,4,-0.060225

1,1,10,11,5,0.046429

1,1,10,11,6,0.011638

1,1,10,11,7,0.027624

1,1,10,11,8,0.0078849

1,1,10,12,1,0.049457

1,1,10,12,2,0.0060068

1,1,10,12,3,-0.027891

1,1,10,12,4,-0.049392

1,1,10,12,5,0.005856

1,1,10,12,6,0.015555

1,1,10,12,7,-0.0015936

1,1,10,12,8,-0.0079065

1,1,10,13,1,0.15195

1,1,10,13,2,0.037899

1,1,10,13,3,-0.055122

1,1,10,13,4,-0.025478

1,1,10,13,5,0.01693

1,1,10,13,6,0.014317

1,1,10,13,7,0.011202

1,1,10,13,8,-0.017649

1,1,10,14,1,0.14356

1,1,10,14,2,0.069212

1,1,10,14,3,-0.055499

1,1,10,14,4,-0.027151

1,1,10,14,5,0.015408

1,1,10,14,6,0.014969

1,1,10,14,7,0.024133

1,1,10,14,8,-0.021875

1,1,10,15,1,0.057623

1,1,10,15,2,0.0093592

1,1,10,15,3,-0.037975

1,1,10,15,4,-0.076934

1,1,10,15,5,0.019459

1,1,10,15,6,0.0027297

1,1,10,15,7,-0.021577

1,1,10,15,8,-0.0039075

1,1,10,16,1,0.04699

1,1,10,16,2,0.020312

1,1,10,16,3,-0.061993

1,1,10,16,4,-0.034102

1,1,10,16,5,0.010261

1,1,10,16,6,-0.0065058

1,1,10,16,7,0.018945

1,1,10,16,8,-0.023824

1,1,10,17,1,0.20979

1,1,10,17,2,0.11583

1,1,10,17,3,-0.058196

1,1,10,17,4,-0.02909

1,1,10,17,5,0.020915

1,1,10,17,6,0.025172

1,1,10,17,7,0.012503

1,1,10,17,8,-0.0053615

1,1,10,18,1,0.083675

1,1,10,18,2,0.028682

1,1,10,18,3,-0.046613

1,1,10,18,4,-0.085871

1,1,10,18,5,0.0161

1,1,10,18,6,-0.0087696

1,1,10,18,7,0.0036056

1,1,10,18,8,-0.021537

1,1,10,19,1,0.10703

1,1,10,19,2,0.0044675

1,1,10,19,3,-0.06052

1,1,10,19,4,-0.046394

1,1,10,19,5,-0.0002272

1,1,10,19,6,0.010767

1,1,10,19,7,0.0087909

1,1,10,19,8,-0.018387

1,1,10,20,1,0.051224

1,1,10,20,2,-0.0039486

1,1,10,20,3,-0.036753

1,1,10,20,4,-0.076672

1,1,10,20,5,0.011906

1,1,10,20,6,-0.014383

1,1,10,20,7,-0.009522

1,1,10,20,8,-0.004218

1,1,11,11,1,0.11143

1,1,11,11,2,0.0364

1,1,11,11,3,-0.017229

1,1,11,11,4,-0.017132

1,1,11,11,5,0.019235

1,1,11,11,6,0.020243

1,1,11,11,7,0.00021655

1,1,11,11,8,0.00098021

1,1,11,12,1,0.045132

1,1,11,12,2,-0.015371

1,1,11,12,3,-0.064817

1,1,11,12,4,-0.069941

1,1,11,12,5,0.01437

1,1,11,12,6,-0.001325

1,1,11,12,7,0.0018002

1,1,11,12,8,-0.020721

1,1,11,13,1,0.12797

1,1,11,13,2,0.030511

1,1,11,13,3,-0.033239

1,1,11,13,4,-0.054714

1,1,11,13,5,0.018729

1,1,11,13,6,0.002561

1,1,11,13,7,0.02325

1,1,11,13,8,-0.024328

1,1,11,14,1,0.10476

1,1,11,14,2,0.059071

1,1,11,14,3,-0.05431

1,1,11,14,4,-0.036228

1,1,11,14,5,0.022612

1,1,11,14,6,0.045311

1,1,11,14,7,0.0047618

1,1,11,14,8,-0.0050129

1,1,11,15,1,0.033248

1,1,11,15,2,0.022186

1,1,11,15,3,-0.060181

1,1,11,15,4,-0.049514

1,1,11,15,5,0.013653

1,1,11,15,6,0.011393

1,1,11,15,7,0.0063936

1,1,11,15,8,-0.018138

1,1,11,16,1,0.053278

1,1,11,16,2,-0.008319

1,1,11,16,3,-0.029188

1,1,11,16,4,-0.032779

1,1,11,16,5,0.0043729

1,1,11,16,6,-0.0061049

1,1,11,16,7,0.010465

1,1,11,16,8,0.0020131

1,1,11,17,1,0.16705

1,1,11,17,2,0.072694

1,1,11,17,3,-0.045389

1,1,11,17,4,-0.057508

1,1,11,17,5,0.02281

1,1,11,17,6,0.0067802

1,1,11,17,7,0.01029

1,1,11,17,8,-0.011052

1,1,11,18,1,0.06068

1,1,11,18,2,0.022146

1,1,11,18,3,-0.086976

1,1,11,18,4,-0.03688

1,1,11,18,5,-0.015224

1,1,11,18,6,0.017023

1,1,11,18,7,0.0051676

1,1,11,18,8,-0.020847

1,1,11,19,1,0.06839

1,1,11,19,2,0.043327

1,1,11,19,3,-0.098935

1,1,11,19,4,-0.01838

1,1,11,19,5,0.011412

1,1,11,19,6,0.018861

1,1,11,19,7,-0.0075855

1,1,11,19,8,-0.01474

1,1,11,20,1,0.031051

1,1,11,20,2,0.00013616

1,1,11,20,3,-0.051622

1,1,11,20,4,-0.082832

1,1,11,20,5,-0.0008935

1,1,11,20,6,0.020048

1,1,11,20,7,0.010832

1,1,11,20,8,-0.029158

1,1,12,12,1,0.029093

1,1,12,12,2,0.025152

1,1,12,12,3,-0.011458

1,1,12,12,4,-0.052683

1,1,12,12,5,-0.015165

1,1,12,12,6,-0.0048888

1,1,12,12,7,-0.022451

1,1,12,12,8,-0.0086312

1,1,12,13,1,0.030749

1,1,12,13,2,-0.00297

1,1,12,13,3,-0.053365

1,1,12,13,4,-0.055727

1,1,12,13,5,-0.0036117

1,1,12,13,6,0.010642

1,1,12,13,7,0.016857

1,1,12,13,8,-0.012475

1,1,12,14,1,0.052204

1,1,12,14,2,0.036637

1,1,12,14,3,-0.068966

1,1,12,14,4,-0.030792

1,1,12,14,5,0.0169

1,1,12,14,6,0.027334

1,1,12,14,7,-0.0032646

1,1,12,14,8,-0.02509

1,1,12,15,1,0.028895

1,1,12,15,2,0.0063539

1,1,12,15,3,-0.066125

1,1,12,15,4,-0.065503

1,1,12,15,5,0.0053947

1,1,12,15,6,0.024178

1,1,12,15,7,-0.0021187

1,1,12,15,8,-0.017842

1,1,12,16,1,0.030932

1,1,12,16,2,0.021617

1,1,12,16,3,-0.062883

1,1,12,16,4,-0.086198

1,1,12,16,5,0.030815

1,1,12,16,6,-0.044578

1,1,12,16,7,-0.042869

1,1,12,16,8,-0.014523

1,1,12,17,1,0.077374

1,1,12,17,2,0.084902

1,1,12,17,3,-0.045876

1,1,12,17,4,-0.059282

1,1,12,17,5,0.0069277

1,1,12,17,6,0.0018707

1,1,12,17,7,0.030258

1,1,12,17,8,-0.0095191

1,1,12,18,1,0.040173

1,1,12,18,2,0.019746

1,1,12,18,3,-0.071222

1,1,12,18,4,-0.068231

1,1,12,18,5,0.014639

1,1,12,18,6,-0.0019771

1,1,12,18,7,-0.016655

1,1,12,18,8,-0.030551

1,1,12,19,1,0.039695

1,1,12,19,2,0.019449

1,1,12,19,3,-0.048148

1,1,12,19,4,-0.028317

1,1,12,19,5,0.037244

1,1,12,19,6,-0.0069541

1,1,12,19,7,0.0040174

1,1,12,19,8,-0.031385

1,1,12,20,1,0.012722

1,1,12,20,2,0.029264

1,1,12,20,3,-0.026311

1,1,12,20,4,-0.033041

1,1,12,20,5,0.014219

1,1,12,20,6,-0.019175

1,1,12,20,7,0.022915

1,1,12,20,8,-0.063633

1,1,13,13,1,0.22096

1,1,13,13,2,-0.0016944

1,1,13,13,3,-0.027223

1,1,13,13,4,-0.013512

1,1,13,13,5,-0.0071747

1,1,13,13,6,-0.0079729

1,1,13,13,7,0.0096744

1,1,13,13,8,-0.014911

1,1,13,14,1,0.20715

1,1,13,14,2,-0.012127

1,1,13,14,3,-0.089949

1,1,13,14,4,-0.038357

1,1,13,14,5,-0.03225

1,1,13,14,6,-0.014484

1,1,13,14,7,-0.0024829

1,1,13,14,8,-0.030912

1,1,13,15,1,0.048676

1,1,13,15,2,0.017448

1,1,13,15,3,-0.06699

1,1,13,15,4,-0.042231

1,1,13,15,5,-0.0046643

1,1,13,15,6,-0.016329

1,1,13,15,7,-0.010614

1,1,13,15,8,-0.025702

1,1,13,16,1,0.033932

1,1,13,16,2,-0.022737

1,1,13,16,3,-0.042409

1,1,13,16,4,-0.026107

1,1,13,16,5,0.0041601

1,1,13,16,6,0.0016849

1,1,13,16,7,0.0095923

1,1,13,16,8,0.0061291

1,1,13,17,1,0.25091

1,1,13,17,2,0.043659

1,1,13,17,3,-0.058058

1,1,13,17,4,-0.053013

1,1,13,17,5,-0.026407

1,1,13,17,6,-0.012147

1,1,13,17,7,-0.0029596

1,1,13,17,8,-0.016777

1,1,13,18,1,0.12591

1,1,13,18,2,0.0050965

1,1,13,18,3,-0.084356

1,1,13,18,4,-0.031112

1,1,13,18,5,-0.020258

1,1,13,18,6,-0.028194

1,1,13,18,7,0.0045436

1,1,13,18,8,-0.028255

1,1,13,19,1,0.069446

1,1,13,19,2,0.026813

1,1,13,19,3,-0.080239

1,1,13,19,4,-0.0079913

1,1,13,19,5,-0.00059213

1,1,13,19,6,-0.018331

1,1,13,19,7,0.006645

1,1,13,19,8,-0.021148

1,1,13,20,1,0.03907

1,1,13,20,2,-0.013004

1,1,13,20,3,-0.031718

1,1,13,20,4,-0.032677

1,1,13,20,5,-0.0015164

1,1,13,20,6,0.012924

1,1,13,20,7,0.0058992

1,1,13,20,8,-0.0036767

1,1,14,14,1,0.13682

1,1,14,14,2,-0.0039023

1,1,14,14,3,-0.057125

1,1,14,14,4,-0.01496

1,1,14,14,5,-0.027384

1,1,14,14,6,-0.010219

1,1,14,14,7,-0.0010306

1,1,14,14,8,-0.01545

1,1,14,15,1,0.04821

1,1,14,15,2,-0.0071238

1,1,14,15,3,-0.074646

1,1,14,15,4,-0.077239

1,1,14,15,5,-0.043089

1,1,14,15,6,-0.017618

1,1,14,15,7,0.01401

1,1,14,15,8,-0.0045587

1,1,14,16,1,0.052635

1,1,14,16,2,-0.00028831

1,1,14,16,3,-0.039158

1,1,14,16,4,-0.018516

1,1,14,16,5,-0.026015

1,1,14,16,6,-0.015591

1,1,14,16,7,0.0038277

1,1,14,16,8,-0.025024

1,1,14,17,1,0.1803

1,1,14,17,2,0.029127

1,1,14,17,3,-0.085776

1,1,14,17,4,-0.067201

1,1,14,17,5,-0.029781

1,1,14,17,6,-0.03614

1,1,14,17,7,-0.0030885

1,1,14,17,8,-0.023468

1,1,14,18,1,0.091249

1,1,14,18,2,0.04127

1,1,14,18,3,-0.1114

1,1,14,18,4,-0.034769

1,1,14,18,5,-0.034913

1,1,14,18,6,-0.012041

1,1,14,18,7,-0.01373

1,1,14,18,8,-0.026753

1,1,14,19,1,0.060603

1,1,14,19,2,-0.039983

1,1,14,19,3,-0.090522

1,1,14,19,4,-0.047313

1,1,14,19,5,-0.027131

1,1,14,19,6,-0.0080159

1,1,14,19,7,-0.013757

1,1,14,19,8,-0.01061

1,1,14,20,1,0.03475

1,1,14,20,2,0.01723

1,1,14,20,3,-0.017069

1,1,14,20,4,-0.028031

1,1,14,20,5,0.0072595

1,1,14,20,6,-0.0029356

1,1,14,20,7,-0.012358

1,1,14,20,8,-0.0061178

1,1,15,15,1,0.031938

1,1,15,15,2,0.019023

1,1,15,15,3,-0.0096871

1,1,15,15,4,-0.0135

1,1,15,15,5,0.011323

1,1,15,15,6,0.0011347

1,1,15,15,7,-0.00012832

1,1,15,15,8,-0.0055143

1,1,15,16,1,0.011434

1,1,15,16,2,0.0034034

1,1,15,16,3,-0.038633

1,1,15,16,4,-0.0071904

1,1,15,16,5,0.0012683

1,1,15,16,6,-0.015577

1,1,15,16,7,0.0074838

1,1,15,16,8,-0.033983

1,1,15,17,1,0.070216

1,1,15,17,2,0.039362

1,1,15,17,3,-0.081478

1,1,15,17,4,-0.072846

1,1,15,17,5,-0.021138

1,1,15,17,6,-0.0079904

1,1,15,17,7,-0.019929

1,1,15,17,8,-0.021793

1,1,15,18,1,0.022957

1,1,15,18,2,0.012198

1,1,15,18,3,-0.024228

1,1,15,18,4,-0.084461

1,1,15,18,5,-0.020288

1,1,15,18,6,-0.015156

1,1,15,18,7,-0.0095533

1,1,15,18,8,-0.034574

1,1,15,19,1,0.021593

1,1,15,19,2,0.010393

1,1,15,19,3,-0.037468

1,1,15,19,4,-0.0014894

1,1,15,19,5,0.0043843

1,1,15,19,6,-0.031971

1,1,15,19,7,-0.010806

1,1,15,19,8,-0.028617

1,1,15,20,1,0.0076215

1,1,15,20,2,-0.0039399

1,1,15,20,3,-0.013476

1,1,15,20,4,-0.029518

1,1,15,20,5,-0.013964

1,1,15,20,6,-0.019843

1,1,15,20,7,-0.028961

1,1,15,20,8,0.019789

1,1,16,16,1,1.0867e-11

1,1,16,16,2,-0.1059

1,1,16,16,3,-0.0080501

1,1,16,16,4,-0.03738

1,1,16,16,5,0.021746

1,1,16,16,6,-0.019686

1,1,16,16,7,0.011102

1,1,16,16,8,0.012314

1,1,16,17,1,0.052231

1,1,16,17,2,-0.018466

1,1,16,17,3,-0.047323

1,1,16,17,4,-0.03928

1,1,16,17,5,-0.00036301

1,1,16,17,6,-0.0097889

1,1,16,17,7,0.017786

1,1,16,17,8,-0.023487

1,1,16,18,1,0.026015

1,1,16,18,2,-0.014451

1,1,16,18,3,-0.044452

1,1,16,18,4,-0.0050054

1,1,16,18,5,0.00041327

1,1,16,18,6,-0.011095

1,1,16,18,7,0.0019706

1,1,16,18,8,-0.01267

1,1,16,19,1,0.014321

1,1,16,19,2,0.026114

1,1,16,19,3,-0.030333

1,1,16,19,4,0.012797

1,1,16,19,5,0.014482

1,1,16,19,6,-0.0179

1,1,16,19,7,-0.00075309

1,1,16,19,8,-0.0068556

1,1,16,20,1,0.015676

1,1,16,20,2,0.01605

1,1,16,20,3,-0.086861

1,1,16,20,4,0.0003952

1,1,16,20,5,0.010203

1,1,16,20,6,-0.0026563

1,1,16,20,7,-0.017526

1,1,16,20,8,0.0021625

1,1,17,17,1,0.25183

1,1,17,17,2,0.025407

1,1,17,17,3,-0.020713

1,1,17,17,4,-0.039026

1,1,17,17,5,-0.020979

1,1,17,17,6,-0.016029

1,1,17,17,7,-0.005364

1,1,17,17,8,-0.013302

1,1,17,18,1,0.12126

1,1,17,18,2,0.036549

1,1,17,18,3,-0.0717

1,1,17,18,4,-0.049651

1,1,17,18,5,-0.043158

1,1,17,18,6,-0.024912

1,1,17,18,7,-0.026574

1,1,17,18,8,-0.01633

1,1,17,19,1,0.066081

1,1,17,19,2,-0.0057902

1,1,17,19,3,-0.070621

1,1,17,19,4,-0.038852

1,1,17,19,5,-0.017121

1,1,17,19,6,-0.02891

1,1,17,19,7,-0.0039909

1,1,17,19,8,-0.015047

1,1,17,20,1,0.044366

1,1,17,20,2,0.0051229

1,1,17,20,3,-0.047872

1,1,17,20,4,-0.033268

1,1,17,20,5,-0.044156

1,1,17,20,6,-0.023503

1,1,17,20,7,-0.013568

1,1,17,20,8,-0.040008

1,1,18,18,1,0.071461

1,1,18,18,2,-0.0059498

1,1,18,18,3,-0.036939

1,1,18,18,4,-0.014962

1,1,18,18,5,-0.012363

1,1,18,18,6,-0.0061542

1,1,18,18,7,-0.0037581

1,1,18,18,8,-0.0098266

1,1,18,19,1,0.062644

1,1,18,19,2,0.054298

1,1,18,19,3,-0.048143

1,1,18,19,4,-0.036607

1,1,18,19,5,-0.0031569

1,1,18,19,6,-0.025665

1,1,18,19,7,-0.00079352

1,1,18,19,8,-0.019378

1,1,18,20,1,0.019056

1,1,18,20,2,0.0077738

1,1,18,20,3,-0.078291

1,1,18,20,4,-0.036473

1,1,18,20,5,-0.0051244

1,1,18,20,6,-0.018151

1,1,18,20,7,-0.0079101

1,1,18,20,8,-0.015678

1,1,19,19,1,0.058718

1,1,19,19,2,-0.0033402

1,1,19,19,3,-0.035188

1,1,19,19,4,-0.031935

1,1,19,19,5,-0.023312

1,1,19,19,6,-0.011103

1,1,19,19,7,0.0051607

1,1,19,19,8,-0.018415

1,1,19,20,1,0.024262

1,1,19,20,2,0.00030704

1,1,19,20,3,-0.061385

1,1,19,20,4,-0.019259

1,1,19,20,5,-0.011182

1,1,19,20,6,-0.0020604

1,1,19,20,7,-0.0017856

1,1,19,20,8,-0.028997

1,1,20,20,1,0.010852

1,1,20,20,2,0.0038865

1,1,20,20,3,-0.011166

1,1,20,20,4,-0.027748

1,1,20,20,5,-0.019114

1,1,20,20,6,-0.014389

1,1,20,20,7,-0.004495

1,1,20,20,8,-0.036613

1,2,1,1,1,0.064208

1,2,1,1,2,-0.013795

1,2,1,1,3,-0.017286

1,2,1,1,4,-0.030892

1,2,1,1,5,-0.0019047

1,2,1,1,6,-0.001885

1,2,1,1,7,0.00044641

1,2,1,1,8,-0.011792

1,2,1,2,1,0.031496

1,2,1,2,2,0.0048195

1,2,1,2,3,-0.0098111

1,2,1,2,4,-0.00068876

1,2,1,2,5,-0.0051359

1,2,1,2,6,0.021361

1,2,1,2,7,-0.0094758

1,2,1,2,8,0.010752

1,2,1,3,1,0.065417

1,2,1,3,2,0.0069296

1,2,1,3,3,-0.082224

1,2,1,3,4,-0.038668

1,2,1,3,5,-0.017254

1,2,1,3,6,-0.0018171

1,2,1,3,7,0.0015512

1,2,1,3,8,-0.012344

1,2,1,4,1,0.057443

1,2,1,4,2,0.020168

1,2,1,4,3,-0.034403

1,2,1,4,4,-0.0058291

1,2,1,4,5,0.0016271

1,2,1,4,6,0.015689

1,2,1,4,7,0.012343

1,2,1,4,8,-0.0096435

1,2,1,5,1,0.097054

1,2,1,5,2,-0.0035076

1,2,1,5,3,-0.052961

1,2,1,5,4,-0.032481

1,2,1,5,5,0.00053573

1,2,1,5,6,0.00068923

1,2,1,5,7,0.0085639

1,2,1,5,8,-0.007172

1,2,1,6,1,0.044796

1,2,1,6,2,0.021288

1,2,1,6,3,-0.049156

1,2,1,6,4,-0.043423

1,2,1,6,5,-0.00093316

1,2,1,6,6,0.0012383

1,2,1,6,7,0.00068857

1,2,1,6,8,-0.014822

1,2,1,7,1,0.049615

1,2,1,7,2,-0.012102

1,2,1,7,3,-0.08233

1,2,1,7,4,-0.058241

1,2,1,7,5,-0.017798

1,2,1,7,6,0.0021647

1,2,1,7,7,8.5728e-05

1,2,1,7,8,-0.011507

1,2,1,8,1,0.058306

1,2,1,8,2,0.0043759

1,2,1,8,3,-0.057667

1,2,1,8,4,-0.021437

1,2,1,8,5,-0.0085296

1,2,1,8,6,-0.013426

1,2,1,8,7,0.016014

1,2,1,8,8,-0.024237

1,2,1,9,1,0.054506

1,2,1,9,2,0.0048433

1,2,1,9,3,-0.042261

1,2,1,9,4,-0.027225

1,2,1,9,5,-0.010582

1,2,1,9,6,0.0083303

1,2,1,9,7,0.011198

1,2,1,9,8,-0.010426

1,2,1,10,1,0.062035

1,2,1,10,2,-0.011173

1,2,1,10,3,-0.05821

1,2,1,10,4,-0.034851

1,2,1,10,5,-0.0015685

1,2,1,10,6,0.0018239

1,2,1,10,7,0.0089212

1,2,1,10,8,-0.013598

1,2,1,11,1,0.065823

1,2,1,11,2,0.028129

1,2,1,11,3,-0.043986

1,2,1,11,4,-0.054202

1,2,1,11,5,-0.0034354

1,2,1,11,6,0.0093314

1,2,1,11,7,0.010162

1,2,1,11,8,-0.0084872

1,2,1,12,1,0.023719

1,2,1,12,2,0.011951

1,2,1,12,3,-0.053353

1,2,1,12,4,-0.050812

1,2,1,12,5,-0.00035668

1,2,1,12,6,0.0035803

1,2,1,12,7,0.00037415

1,2,1,12,8,-0.009137

1,2,1,13,1,0.078799

1,2,1,13,2,0.037812

1,2,1,13,3,-0.036952

1,2,1,13,4,-0.0054908

1,2,1,13,5,0.0041873

1,2,1,13,6,0.029249

1,2,1,13,7,0.009758

1,2,1,13,8,-0.0014635

1,2,1,14,1,0.075338

1,2,1,14,2,0.018868

1,2,1,14,3,-0.034694

1,2,1,14,4,0.0020278

1,2,1,14,5,0.012592

1,2,1,14,6,0.020577

1,2,1,14,7,0.012846

1,2,1,14,8,-0.0038292

1,2,1,15,1,0.040132

1,2,1,15,2,0.0056287

1,2,1,15,3,-0.026825

1,2,1,15,4,-0.016404

1,2,1,15,5,-0.0028546

1,2,1,15,6,0.011963

1,2,1,15,7,0.0094442

1,2,1,15,8,-0.01293

1,2,1,16,1,0.024032

1,2,1,16,2,0.04654

1,2,1,16,3,-0.0539

1,2,1,16,4,-0.0082995

1,2,1,16,5,0.0028506

1,2,1,16,6,0.013474

1,2,1,16,7,0.004889

1,2,1,16,8,-0.027901

1,2,1,17,1,0.12267

1,2,1,17,2,0.068055

1,2,1,17,3,-0.031447

1,2,1,17,4,-0.01468

1,2,1,17,5,0.00084214

1,2,1,17,6,0.023678

1,2,1,17,7,0.010498

1,2,1,17,8,-0.0010806

1,2,1,18,1,0.054259

1,2,1,18,2,0.045377

1,2,1,18,3,-0.042714

1,2,1,18,4,-0.01666

1,2,1,18,5,0.0063865

1,2,1,18,6,0.022197

1,2,1,18,7,0.014266

1,2,1,18,8,0.00077048

1,2,1,19,1,0.052181

1,2,1,19,2,0.033941

1,2,1,19,3,-0.043246

1,2,1,19,4,-0.028391

1,2,1,19,5,-0.01492

1,2,1,19,6,0.0027197

1,2,1,19,7,0.0055026

1,2,1,19,8,-0.014169

1,2,1,20,1,0.018732

1,2,1,20,2,-0.0017372

1,2,1,20,3,-0.045403

1,2,1,20,4,-0.0060774

1,2,1,20,5,-0.0092047

1,2,1,20,6,0.0095496

1,2,1,20,7,0.0087863

1,2,1,20,8,-0.0042103

1,2,2,2,1,0.005722

1,2,2,2,2,-0.0020483

1,2,2,2,3,-0.00062247

1,2,2,2,4,-0.011584

1,2,2,2,5,-0.014328

1,2,2,2,6,-0.017227

1,2,2,2,7,-0.0072454

1,2,2,2,8,-0.012331

1,2,2,3,1,2.2889e-07

1,2,2,3,2,0.021011

1,2,2,3,3,-0.046253

1,2,2,3,4,-0.027726

1,2,2,3,5,-0.021144

1,2,2,3,6,-0.0087439

1,2,2,3,7,-0.0081192

1,2,2,3,8,-0.0040555

1,2,2,4,1,0.021966

1,2,2,4,2,-0.0069819

1,2,2,4,3,-0.024134

1,2,2,4,4,-0.03101

1,2,2,4,5,-0.018638

1,2,2,4,6,-0.0067638

1,2,2,4,7,2.2574e-05

1,2,2,4,8,-0.0066044

1,2,2,5,1,2.008e-09

1,2,2,5,2,0.01889

1,2,2,5,3,-0.035043

1,2,2,5,4,-0.029317

1,2,2,5,5,-0.022652

1,2,2,5,6,0.0027927

1,2,2,5,7,0.0031554

1,2,2,5,8,-0.0058261

1,2,2,6,1,0.020307

1,2,2,6,2,0.0025469

1,2,2,6,3,-0.039111

1,2,2,6,4,0.00079459

1,2,2,6,5,-0.018929

1,2,2,6,6,0.0024367

1,2,2,6,7,-0.00549

1,2,2,6,8,-0.0055788

1,2,2,7,1,0.013434

1,2,2,7,2,0.010784

1,2,2,7,3,-0.025596

1,2,2,7,4,-0.046831

1,2,2,7,5,-0.016945

1,2,2,7,6,-0.024594

1,2,2,7,7,-0.012355

1,2,2,7,8,-0.012746

1,2,2,8,1,0.014995

1,2,2,8,2,0.024831

1,2,2,8,3,-0.049618

1,2,2,8,4,-0.027994

1,2,2,8,5,-0.024604

1,2,2,8,6,-0.01221

1,2,2,8,7,-0.013578

1,2,2,8,8,-0.0097218

1,2,2,9,1,0.0038251

1,2,2,9,2,0.016153

1,2,2,9,3,-0.023024

1,2,2,9,4,-0.0053714

1,2,2,9,5,-0.020484

1,2,2,9,6,0.01554

1,2,2,9,7,0.0022882

1,2,2,9,8,-0.0054692

1,2,2,10,1,6.0235e-10

1,2,2,10,2,0.014798

1,2,2,10,3,-0.017383

1,2,2,10,4,-0.014848

1,2,2,10,5,-0.0078602

1,2,2,10,6,-0.0078319

1,2,2,10,7,0.0068951

1,2,2,10,8,-0.010725

1,2,2,11,1,0.0066997

1,2,2,11,2,-0.029991

1,2,2,11,3,-0.048517

1,2,2,11,4,-0.046538

1,2,2,11,5,-0.025535

1,2,2,11,6,-0.015248

1,2,2,11,7,-0.0070572

1,2,2,11,8,-0.030344

1,2,2,12,1,0.007824

1,2,2,12,2,0.0091152

1,2,2,12,3,-0.010486

1,2,2,12,4,-0.031003

1,2,2,12,5,-0.012554

1,2,2,12,6,-0.015841

1,2,2,12,7,-0.0051133

1,2,2,12,8,-0.009558

1,2,2,13,1,0.018547

1,2,2,13,2,-0.015502

1,2,2,13,3,-0.037481

1,2,2,13,4,-0.042506

1,2,2,13,5,-0.026871

1,2,2,13,6,0.0063615

1,2,2,13,7,0.0054111

1,2,2,13,8,-0.0041157

1,2,2,14,1,0.022081

1,2,2,14,2,-0.021169

1,2,2,14,3,-0.019161

1,2,2,14,4,0.0025716

1,2,2,14,5,-0.018915

1,2,2,14,6,0.007616

1,2,2,14,7,0.0033759

1,2,2,14,8,-0.004116

1,2,2,15,1,0.014747

1,2,2,15,2,-0.01445

1,2,2,15,3,-0.032556

1,2,2,15,4,-0.029136

1,2,2,15,5,-0.014725

1,2,2,15,6,-0.0096861

1,2,2,15,7,-0.01558

1,2,2,15,8,-0.01057

1,2,2,16,1,0.0056524

1,2,2,16,2,0.021963

1,2,2,16,3,-0.020448

1,2,2,16,4,0.021684

1,2,2,16,5,-0.023153

1,2,2,16,6,0.011058

1,2,2,16,7,-0.0029327

1,2,2,16,8,0.0013733

1,2,2,17,1,0.020102

1,2,2,17,2,-0.0032767

1,2,2,17,3,-0.012247

1,2,2,17,4,0.0015858

1,2,2,17,5,-0.013297

1,2,2,17,6,0.00644

1,2,2,17,7,0.012098

1,2,2,17,8,-0.010345

1,2,2,18,1,6.2523e-10

1,2,2,18,2,0.020601

1,2,2,18,3,-0.0080653

1,2,2,18,4,-0.031909

1,2,2,18,5,-0.016651

1,2,2,18,6,-0.0022229

1,2,2,18,7,0.0010037

1,2,2,18,8,-0.00096976

1,2,2,19,1,1.8064e-10

1,2,2,19,2,-0.011185

1,2,2,19,3,-0.037844

1,2,2,19,4,-0.012726

1,2,2,19,5,-0.028657

1,2,2,19,6,-0.00974

1,2,2,19,7,0.0061876

1,2,2,19,8,-0.01912

1,2,2,20,1,5.068e-10

1,2,2,20,2,0.00047719

1,2,2,20,3,-0.031611

1,2,2,20,4,-0.036088

1,2,2,20,5,-0.036224

1,2,2,20,6,-0.018449

1,2,2,20,7,-0.022981

1,2,2,20,8,-0.006006

1,2,3,3,1,0.034598

1,2,3,3,2,0.023806

1,2,3,3,3,-0.02764

1,2,3,3,4,-0.014597

1,2,3,3,5,0.012814

1,2,3,3,6,0.0033475

1,2,3,3,7,0.0038189

1,2,3,3,8,0.00012696

1,2,3,4,1,0.061719

1,2,3,4,2,0.046294

1,2,3,4,3,-0.036789

1,2,3,4,4,-0.0086942

1,2,3,4,5,0.026416

1,2,3,4,6,0.02646

1,2,3,4,7,0.023745

1,2,3,4,8,0.012241

1,2,3,5,1,0.090153

1,2,3,5,2,0.045135

1,2,3,5,3,-0.047187

1,2,3,5,4,-0.026724

1,2,3,5,5,0.0086697

1,2,3,5,6,0.0096856

1,2,3,5,7,0.010299

1,2,3,5,8,-0.0092077

1,2,3,6,1,0.032523

1,2,3,6,2,0.029467

1,2,3,6,3,-0.077104

1,2,3,6,4,-0.049839

1,2,3,6,5,0.0068117

1,2,3,6,6,-0.0061425

1,2,3,6,7,0.0060533

1,2,3,6,8,-0.0096866

1,2,3,7,1,0.044808

1,2,3,7,2,0.029458

1,2,3,7,3,-0.025533

1,2,3,7,4,-0.014454

1,2,3,7,5,0.024008

1,2,3,7,6,0.034592

1,2,3,7,7,0.018194

1,2,3,7,8,0.0023339

1,2,3,8,1,0.055823

1,2,3,8,2,-0.010326

1,2,3,8,3,-0.10547

1,2,3,8,4,-0.032124

1,2,3,8,5,-0.020871

1,2,3,8,6,-0.0060071

1,2,3,8,7,0.0092134

1,2,3,8,8,-0.015057

1,2,3,9,1,0.04554

1,2,3,9,2,0.02902

1,2,3,9,3,-0.06195

1,2,3,9,4,-0.029124

1,2,3,9,5,-0.010969

1,2,3,9,6,0.01231

1,2,3,9,7,0.021016

1,2,3,9,8,-0.0094201

1,2,3,10,1,0.052869

1,2,3,10,2,-0.024769

1,2,3,10,3,-0.094184

1,2,3,10,4,-0.069571

1,2,3,10,5,-0.024575

1,2,3,10,6,-0.01994

1,2,3,10,7,-0.0039279

1,2,3,10,8,-0.033194

1,2,3,11,1,0.035374

1,2,3,11,2,-0.010164

1,2,3,11,3,-0.099289

1,2,3,11,4,-0.052297

1,2,3,11,5,-0.023159

1,2,3,11,6,-0.017085

1,2,3,11,7,-0.0034797

1,2,3,11,8,-0.02417

1,2,3,12,1,0.024423

1,2,3,12,2,0.029957

1,2,3,12,3,-0.044075

1,2,3,12,4,-0.055585

1,2,3,12,5,-0.0078917

1,2,3,12,6,-0.01255

1,2,3,12,7,0.0086549

1,2,3,12,8,-0.033692

1,2,3,13,1,0.046356

1,2,3,13,2,0.072799

1,2,3,13,3,-0.016498

1,2,3,13,4,0.0036926

1,2,3,13,5,0.014974

1,2,3,13,6,0.039615

1,2,3,13,7,0.029332

1,2,3,13,8,0.0011926

1,2,3,14,1,0.045144

1,2,3,14,2,0.072938

1,2,3,14,3,-0.038234

1,2,3,14,4,-0.020961

1,2,3,14,5,0.016746

1,2,3,14,6,0.02615

1,2,3,14,7,0.02801

1,2,3,14,8,-0.0026145

1,2,3,15,1,0.023361

1,2,3,15,2,0.021981

1,2,3,15,3,-0.033494

1,2,3,15,4,-0.039555

1,2,3,15,5,0.0033615

1,2,3,15,6,-0.0053415

1,2,3,15,7,0.015757

1,2,3,15,8,-0.014966

1,2,3,16,1,0.017268

1,2,3,16,2,0.020757

1,2,3,16,3,-0.048275

1,2,3,16,4,-0.025164

1,2,3,16,5,-0.0028198

1,2,3,16,6,0.0052866

1,2,3,16,7,0.0028404

1,2,3,16,8,0.0059373

1,2,3,17,1,0.094949

1,2,3,17,2,0.09351

1,2,3,17,3,-0.032843

1,2,3,17,4,-0.0083712

1,2,3,17,5,0.021245

1,2,3,17,6,0.031501

1,2,3,17,7,0.024199

1,2,3,17,8,-0.0009695

1,2,3,18,1,0.034875

1,2,3,18,2,0.047399

1,2,3,18,3,-0.03106

1,2,3,18,4,-0.02836

1,2,3,18,5,0.027415

1,2,3,18,6,0.017608

1,2,3,18,7,0.018629

1,2,3,18,8,-0.0096757

1,2,3,19,1,0.028704

1,2,3,19,2,0.021568

1,2,3,19,3,-0.011968

1,2,3,19,4,-0.030624

1,2,3,19,5,0.0030998

1,2,3,19,6,0.0054742

1,2,3,19,7,0.0017576

1,2,3,19,8,-0.018964

1,2,3,20,1,0.014788

1,2,3,20,2,0.0039291

1,2,3,20,3,-0.029775

1,2,3,20,4,-0.031689

1,2,3,20,5,0.016717

1,2,3,20,6,0.0058017

1,2,3,20,7,0.019769

1,2,3,20,8,-0.011649

1,2,4,4,1,0.070538

1,2,4,4,2,0.0048161

1,2,4,4,3,-0.0021373

1,2,4,4,4,-0.0060043

1,2,4,4,5,0.0047871

1,2,4,4,6,0.011633

1,2,4,4,7,0.0077274

1,2,4,4,8,0.0058241

1,2,4,5,1,0.053534

1,2,4,5,2,-0.0079308

1,2,4,5,3,-0.046913

1,2,4,5,4,-0.058523

1,2,4,5,5,-0.0018723

1,2,4,5,6,0.0039143

1,2,4,5,7,0.013878

1,2,4,5,8,-0.019977

1,2,4,6,1,0.04978

1,2,4,6,2,0.0073046

1,2,4,6,3,-0.040369

1,2,4,6,4,-0.02211

1,2,4,6,5,0.01005

1,2,4,6,6,0.018574

1,2,4,6,7,0.013224

1,2,4,6,8,0.003195

1,2,4,7,1,0.04324

1,2,4,7,2,0.0070281

1,2,4,7,3,-0.039744

1,2,4,7,4,-0.046435

1,2,4,7,5,0.0061441

1,2,4,7,6,0.015128

1,2,4,7,7,0.0093644

1,2,4,7,8,-0.0061292

1,2,4,8,1,0.044404

1,2,4,8,2,0.014619

1,2,4,8,3,-0.071156

1,2,4,8,4,-0.029452

1,2,4,8,5,-0.0041839

1,2,4,8,6,0.0031114

1,2,4,8,7,0.0036146

1,2,4,8,8,-0.009099

1,2,4,9,1,0.043038

1,2,4,9,2,-0.020269

1,2,4,9,3,-0.048229

1,2,4,9,4,-0.017165

1,2,4,9,5,-0.017668

1,2,4,9,6,0.0072022

1,2,4,9,7,0.021795

1,2,4,9,8,-0.0081828

1,2,4,10,1,0.065089

1,2,4,10,2,-0.054831

1,2,4,10,3,-0.085279

1,2,4,10,4,-0.081924

1,2,4,10,5,-0.022334

1,2,4,10,6,-0.013819

1,2,4,10,7,0.0042127

1,2,4,10,8,-0.009741

1,2,4,11,1,0.027881

1,2,4,11,2,0.0041342

1,2,4,11,3,-0.11736

1,2,4,11,4,-0.071712

1,2,4,11,5,-0.028818

1,2,4,11,6,-0.001062

1,2,4,11,7,0.00077984

1,2,4,11,8,-0.016165

1,2,4,12,1,0.03021

1,2,4,12,2,0.016507

1,2,4,12,3,-0.026365

1,2,4,12,4,-0.045511

1,2,4,12,5,0.0057816

1,2,4,12,6,-0.0040941

1,2,4,12,7,0.0083412

1,2,4,12,8,-0.017048

1,2,4,13,1,0.055814

1,2,4,13,2,-0.029853

1,2,4,13,3,-0.081715

1,2,4,13,4,-0.066456

1,2,4,13,5,-0.020881

1,2,4,13,6,0.0073815

1,2,4,13,7,0.023132

1,2,4,13,8,-0.0054564

1,2,4,14,1,0.065861

1,2,4,14,2,-0.012856

1,2,4,14,3,-0.053123

1,2,4,14,4,-0.042308

1,2,4,14,5,-0.0050762

1,2,4,14,6,0.0092395

1,2,4,14,7,0.019862

1,2,4,14,8,-0.01105

1,2,4,15,1,0.017211

1,2,4,15,2,-0.0026426

1,2,4,15,3,-0.024382

1,2,4,15,4,-0.044497

1,2,4,15,5,0.00066826

1,2,4,15,6,0.017825

1,2,4,15,7,0.028743

1,2,4,15,8,-0.0018035

1,2,4,16,1,0.0035257

1,2,4,16,2,-0.039537

1,2,4,16,3,-0.028087

1,2,4,16,4,-0.029201

1,2,4,16,5,-0.0094447

1,2,4,16,6,-0.0054519

1,2,4,16,7,0.0038682

1,2,4,16,8,-0.0017939

1,2,4,17,1,0.083327

1,2,4,17,2,0.029304

1,2,4,17,3,-0.024867

1,2,4,17,4,-0.037333

1,2,4,17,5,0.014666

1,2,4,17,6,0.010129

1,2,4,17,7,0.012855

1,2,4,17,8,-0.007191

1,2,4,18,1,0.042525

1,2,4,18,2,0.0080344

1,2,4,18,3,-0.035557

1,2,4,18,4,-0.039541

1,2,4,18,5,-0.0083731

1,2,4,18,6,0.0093024

1,2,4,18,7,0.0068314

1,2,4,18,8,-0.0084439

1,2,4,19,1,0.028152

1,2,4,19,2,0.031043

1,2,4,19,3,-0.03891

1,2,4,19,4,-0.0095886

1,2,4,19,5,-0.0079968

1,2,4,19,6,0.003425

1,2,4,19,7,0.022584

1,2,4,19,8,-0.025847

1,2,4,20,1,0.014574

1,2,4,20,2,-0.026382

1,2,4,20,3,-0.037913

1,2,4,20,4,-0.044156

1,2,4,20,5,-0.030065

1,2,4,20,6,-0.0066024

1,2,4,20,7,0.0028163

1,2,4,20,8,-0.012436

1,2,5,5,1,0.075476

1,2,5,5,2,-0.0030551

1,2,5,5,3,-0.027496

1,2,5,5,4,-0.035121

1,2,5,5,5,-0.004257

1,2,5,5,6,0.0043727

1,2,5,5,7,0.0032176

1,2,5,5,8,-0.007352

1,2,5,6,1,0.057082

1,2,5,6,2,0.048665

1,2,5,6,3,-0.045726

1,2,5,6,4,-0.02047

1,2,5,6,5,0.0062026

1,2,5,6,6,0.014533

1,2,5,6,7,0.020298

1,2,5,6,8,-0.008336

1,2,5,7,1,0.07058

1,2,5,7,2,0.018611

1,2,5,7,3,-0.060031

1,2,5,7,4,-0.049062

1,2,5,7,5,0.0080992

1,2,5,7,6,0.018769

1,2,5,7,7,0.012864

1,2,5,7,8,-0.015498

1,2,5,8,1,0.06214

1,2,5,8,2,0.056851

1,2,5,8,3,-0.066045

1,2,5,8,4,-0.038746

1,2,5,8,5,-0.0010944

1,2,5,8,6,0.018286

1,2,5,8,7,0.0086547

1,2,5,8,8,-0.0054853

1,2,5,9,1,0.04045

1,2,5,9,2,0.024364

1,2,5,9,3,-0.034251

1,2,5,9,4,-0.029218

1,2,5,9,5,-0.0036704

1,2,5,9,6,0.023139

1,2,5,9,7,0.021276

1,2,5,9,8,-0.0014129

1,2,5,10,1,0.093417

1,2,5,10,2,0.054006

1,2,5,10,3,-0.047628

1,2,5,10,4,-0.044054

1,2,5,10,5,0.0091647

1,2,5,10,6,-0.0019919

1,2,5,10,7,0.012506

1,2,5,10,8,-0.025462

1,2,5,11,1,0.046089

1,2,5,11,2,0.053039

1,2,5,11,3,-0.083907

1,2,5,11,4,-0.034965

1,2,5,11,5,-0.0048151

1,2,5,11,6,0.01229

1,2,5,11,7,0.0072264

1,2,5,11,8,-0.017678

1,2,5,12,1,0.040427

1,2,5,12,2,0.023259

1,2,5,12,3,-0.04542

1,2,5,12,4,-0.035174

1,2,5,12,5,0.007395

1,2,5,12,6,0.011008

1,2,5,12,7,0.005614

1,2,5,12,8,-0.0006698

1,2,5,13,1,0.089957

1,2,5,13,2,0.013842

1,2,5,13,3,-0.079281

1,2,5,13,4,-0.051699

1,2,5,13,5,-0.033562

1,2,5,13,6,-0.0044955

1,2,5,13,7,0.01892

1,2,5,13,8,-0.010288

1,2,5,14,1,0.054079

1,2,5,14,2,-0.031116

1,2,5,14,3,-0.083753

1,2,5,14,4,-0.060522

1,2,5,14,5,-0.042825

1,2,5,14,6,-0.01391

1,2,5,14,7,0.003707

1,2,5,14,8,-0.011675

1,2,5,15,1,0.028181

1,2,5,15,2,-0.0079935

1,2,5,15,3,-0.034471

1,2,5,15,4,-0.062826

1,2,5,15,5,-0.015496

1,2,5,15,6,-0.017558

1,2,5,15,7,-0.0042806

1,2,5,15,8,-0.013193

1,2,5,16,1,0.031661

1,2,5,16,2,0.0098225

1,2,5,16,3,-0.015488

1,2,5,16,4,-0.01035

1,2,5,16,5,0.019552

1,2,5,16,6,-0.01257

1,2,5,16,7,0.02466

1,2,5,16,8,-0.018782

1,2,5,17,1,0.11676

1,2,5,17,2,0.030875

1,2,5,17,3,-0.050823

1,2,5,17,4,-0.065767

1,2,5,17,5,-0.024786

1,2,5,17,6,-0.018131

1,2,5,17,7,-0.0019607

1,2,5,17,8,-0.020465

1,2,5,18,1,0.015148

1,2,5,18,2,-0.013986

1,2,5,18,3,-0.076943

1,2,5,18,4,-0.074907

1,2,5,18,5,-0.039658

1,2,5,18,6,-0.027616

1,2,5,18,7,-0.0080249

1,2,5,18,8,-0.033302

1,2,5,19,1,0.026869

1,2,5,19,2,0.0032513

1,2,5,19,3,-0.074285

1,2,5,19,4,-0.065702

1,2,5,19,5,-0.02804

1,2,5,19,6,-0.017033

1,2,5,19,7,0.0031926

1,2,5,19,8,-0.028919

1,2,5,20,1,0.016632

1,2,5,20,2,-0.009074

1,2,5,20,3,-0.042912

1,2,5,20,4,-0.037638

1,2,5,20,5,-0.034071

1,2,5,20,6,-0.0101

1,2,5,20,7,-0.016896

1,2,5,20,8,-0.035556

1,2,6,6,1,0.029482

1,2,6,6,2,-0.005861

1,2,6,6,3,-0.052826

1,2,6,6,4,-0.032835

1,2,6,6,5,-0.02053

1,2,6,6,6,-0.015625

1,2,6,6,7,-0.00053321

1,2,6,6,8,-0.013862

1,2,6,7,1,0.052455

1,2,6,7,2,0.014892

1,2,6,7,3,-0.065786

1,2,6,7,4,-0.037709

1,2,6,7,5,0.010649

1,2,6,7,6,0.00010265

1,2,6,7,7,0.0048172

1,2,6,7,8,-0.0090749

1,2,6,8,1,0.04876

1,2,6,8,2,0.0085103

1,2,6,8,3,-0.065058

1,2,6,8,4,-0.057711

1,2,6,8,5,-0.013068

1,2,6,8,6,-0.022036

1,2,6,8,7,0.0043208

1,2,6,8,8,-0.024589

1,2,6,9,1,0.039829

1,2,6,9,2,0.035382

1,2,6,9,3,-0.064173

1,2,6,9,4,-0.028851

1,2,6,9,5,-0.027528

1,2,6,9,6,0.015593

1,2,6,9,7,-0.0012868

1,2,6,9,8,-0.0028206

1,2,6,10,1,0.039296

1,2,6,10,2,0.0055391

1,2,6,10,3,-0.073425

1,2,6,10,4,-0.053135

1,2,6,10,5,0.0091439

1,2,6,10,6,0.0016272

1,2,6,10,7,0.008088

1,2,6,10,8,-0.010545

1,2,6,11,1,0.041123

1,2,6,11,2,0.006447

1,2,6,11,3,-0.061987

1,2,6,11,4,-0.054854

1,2,6,11,5,0.0052459

1,2,6,11,6,0.0061543

1,2,6,11,7,0.00093503

1,2,6,11,8,-0.01551

1,2,6,12,1,0.017813

1,2,6,12,2,0.022535

1,2,6,12,3,-0.049055

1,2,6,12,4,-0.053983

1,2,6,12,5,-0.0032523

1,2,6,12,6,-0.008579

1,2,6,12,7,0.014718

1,2,6,12,8,-0.015668

1,2,6,13,1,0.061446

1,2,6,13,2,0.041378

1,2,6,13,3,-0.016756

1,2,6,13,4,-0.023597

1,2,6,13,5,0.0030046

1,2,6,13,6,0.023163

1,2,6,13,7,0.02826

1,2,6,13,8,-0.011852

1,2,6,14,1,0.043981

1,2,6,14,2,0.043138

1,2,6,14,3,-0.023891

1,2,6,14,4,-0.0262

1,2,6,14,5,0.0082955

1,2,6,14,6,0.016216

1,2,6,14,7,0.024689

1,2,6,14,8,-0.0038663

1,2,6,15,1,0.029311

1,2,6,15,2,0.039757

1,2,6,15,3,-0.014685

1,2,6,15,4,-0.041259

1,2,6,15,5,0.013246

1,2,6,15,6,0.0048593

1,2,6,15,7,-0.0029653

1,2,6,15,8,-0.024997

1,2,6,16,1,0.016693

1,2,6,16,2,0.021097

1,2,6,16,3,-0.021452

1,2,6,16,4,-0.022018

1,2,6,16,5,-0.013674

1,2,6,16,6,0.0017422

1,2,6,16,7,0.0051773

1,2,6,16,8,-0.007931

1,2,6,17,1,0.058723

1,2,6,17,2,0.069206

1,2,6,17,3,-0.034434

1,2,6,17,4,-0.025105

1,2,6,17,5,0.033767

1,2,6,17,6,0.026798

1,2,6,17,7,0.023041

1,2,6,17,8,-0.0015875

1,2,6,18,1,0.039344

1,2,6,18,2,0.029669

1,2,6,18,3,-0.024252

1,2,6,18,4,-0.03262

1,2,6,18,5,0.0013633

1,2,6,18,6,0.015702

1,2,6,18,7,0.019471

1,2,6,18,8,0.0024036

1,2,6,19,1,0.029458

1,2,6,19,2,0.017539

1,2,6,19,3,-0.030181

1,2,6,19,4,-0.040809

1,2,6,19,5,0.0055404

1,2,6,19,6,0.022566

1,2,6,19,7,0.0075399

1,2,6,19,8,-0.00034029

1,2,6,20,1,0.013844

1,2,6,20,2,0.0020475

1,2,6,20,3,-0.048859

1,2,6,20,4,-0.059997

1,2,6,20,5,-0.0105

1,2,6,20,6,0.008135

1,2,6,20,7,0.0012837

1,2,6,20,8,-0.015562

1,2,7,7,1,0.023988

1,2,7,7,2,0.0098459

1,2,7,7,3,-0.017128

1,2,7,7,4,-0.030737

1,2,7,7,5,0.0023398

1,2,7,7,6,0.015482

1,2,7,7,7,0.0013411

1,2,7,7,8,-0.0061116

1,2,7,8,1,0.052786

1,2,7,8,2,0.026284

1,2,7,8,3,-0.06045

1,2,7,8,4,-0.033004

1,2,7,8,5,-0.0049349

1,2,7,8,6,0.011247

1,2,7,8,7,0.0046905

1,2,7,8,8,-0.014796

1,2,7,9,1,0.0097688

1,2,7,9,2,-0.016305

1,2,7,9,3,-0.081319

1,2,7,9,4,-0.043467

1,2,7,9,5,-0.01013

1,2,7,9,6,0.014

1,2,7,9,7,0.012945

1,2,7,9,8,-0.012251

1,2,7,10,1,0.040488

1,2,7,10,2,0.01528

1,2,7,10,3,-0.075717

1,2,7,10,4,-0.021466

1,2,7,10,5,0.0053933

1,2,7,10,6,0.01907

1,2,7,10,7,0.011954

1,2,7,10,8,-0.00030451

1,2,7,11,1,0.014989

1,2,7,11,2,-0.0057746

1,2,7,11,3,-0.062233

1,2,7,11,4,-0.069722

1,2,7,11,5,-0.0099322

1,2,7,11,6,0.0061533

1,2,7,11,7,0.0072087

1,2,7,11,8,-0.0074519

1,2,7,12,1,0.014497

1,2,7,12,2,0.019869

1,2,7,12,3,0.0052758

1,2,7,12,4,-0.036479

1,2,7,12,5,0.033496

1,2,7,12,6,0.016754

1,2,7,12,7,0.018159

1,2,7,12,8,-0.018907

1,2,7,13,1,0.061765

1,2,7,13,2,0.003234

1,2,7,13,3,-0.064782

1,2,7,13,4,-0.054792

1,2,7,13,5,0.001316

1,2,7,13,6,0.022762

1,2,7,13,7,0.016757

1,2,7,13,8,-0.021389

1,2,7,14,1,0.038997

1,2,7,14,2,-0.010306

1,2,7,14,3,-0.042997

1,2,7,14,4,-0.068925

1,2,7,14,5,-0.0006

1,2,7,14,6,0.0088448

1,2,7,14,7,0.022695

1,2,7,14,8,-0.010777

1,2,7,15,1,0.016763

1,2,7,15,2,0.03096

1,2,7,15,3,-0.034744

1,2,7,15,4,-0.034162

1,2,7,15,5,0.010031

1,2,7,15,6,-0.00076953

1,2,7,15,7,-0.0035451

1,2,7,15,8,-0.01296

1,2,7,16,1,0.021943

1,2,7,16,2,-0.0095479

1,2,7,16,3,-0.047101

1,2,7,16,4,-0.029356

1,2,7,16,5,-0.026291

1,2,7,16,6,0.011426

1,2,7,16,7,-0.0068737

1,2,7,16,8,-0.012106

1,2,7,17,1,0.068483

1,2,7,17,2,0.057596

1,2,7,17,3,-0.039632

1,2,7,17,4,-0.043144

1,2,7,17,5,0.015309

1,2,7,17,6,0.020854

1,2,7,17,7,0.02066

1,2,7,17,8,-0.018481

1,2,7,18,1,0.020077

1,2,7,18,2,0.010228

1,2,7,18,3,-0.04814

1,2,7,18,4,-0.040679

1,2,7,18,5,-0.0019558

1,2,7,18,6,0.020766

1,2,7,18,7,0.010084

1,2,7,18,8,-0.0029811

1,2,7,19,1,0.023759

1,2,7,19,2,-0.0033808

1,2,7,19,3,-0.066608

1,2,7,19,4,-0.050592

1,2,7,19,5,-0.033325

1,2,7,19,6,0.0070899

1,2,7,19,7,-0.0027395

1,2,7,19,8,-0.01446

1,2,7,20,1,0.001623

1,2,7,20,2,-0.029463

1,2,7,20,3,-0.060692

1,2,7,20,4,-0.050352

1,2,7,20,5,-0.012924

1,2,7,20,6,0.00047522

1,2,7,20,7,-0.021472

1,2,7,20,8,-0.0098665

1,2,8,8,1,0.068693

1,2,8,8,2,0.013168

1,2,8,8,3,-0.033729

1,2,8,8,4,-0.0073703

1,2,8,8,5,-0.0065537

1,2,8,8,6,0.0065624

1,2,8,8,7,0.0007606

1,2,8,8,8,-0.0056529

1,2,8,9,1,0.047846

1,2,8,9,2,-0.026592

1,2,8,9,3,-0.046588

1,2,8,9,4,-0.013688

1,2,8,9,5,0.0012542

1,2,8,9,6,0.0094313

1,2,8,9,7,0.018588

1,2,8,9,8,-0.0023778

1,2,8,10,1,0.072775

1,2,8,10,2,-0.0036817

1,2,8,10,3,-0.061689

1,2,8,10,4,-0.03864

1,2,8,10,5,0.00116

1,2,8,10,6,-0.0047252

1,2,8,10,7,0.0065871

1,2,8,10,8,-0.016978

1,2,8,11,1,0.060109

1,2,8,11,2,0.0054648

1,2,8,11,3,-0.07071

1,2,8,11,4,-0.055456

1,2,8,11,5,-0.015229

1,2,8,11,6,-6.6078e-05

1,2,8,11,7,0.0048337

1,2,8,11,8,-0.014145

1,2,8,12,1,0.043904

1,2,8,12,2,0.029159

1,2,8,12,3,-0.070573

1,2,8,12,4,-0.046867

1,2,8,12,5,0.00012668

1,2,8,12,6,0.010646

1,2,8,12,7,0.006256

1,2,8,12,8,-0.0049489

1,2,8,13,1,0.06805

1,2,8,13,2,0.023081

1,2,8,13,3,-0.048778

1,2,8,13,4,-0.031763

1,2,8,13,5,0.0017448

1,2,8,13,6,0.022296

1,2,8,13,7,0.028279

1,2,8,13,8,-0.0070297

1,2,8,14,1,0.048992

1,2,8,14,2,0.028078

1,2,8,14,3,-0.055586

1,2,8,14,4,-0.035461

1,2,8,14,5,0.012499

1,2,8,14,6,0.020469

1,2,8,14,7,0.0174

1,2,8,14,8,-0.011185

1,2,8,15,1,0.027601

1,2,8,15,2,-0.0032904

1,2,8,15,3,-0.061218

1,2,8,15,4,-0.044304

1,2,8,15,5,-0.012963

1,2,8,15,6,-0.00054817

1,2,8,15,7,0.0075839

1,2,8,15,8,-0.0018705

1,2,8,16,1,0.029258

1,2,8,16,2,0.051124

1,2,8,16,3,-0.032801

1,2,8,16,4,-0.016362

1,2,8,16,5,-0.0039535

1,2,8,16,6,-0.0014347

1,2,8,16,7,0.019163

1,2,8,16,8,-0.014747

1,2,8,17,1,0.06236

1,2,8,17,2,0.065056

1,2,8,17,3,-0.035188

1,2,8,17,4,-0.016803

1,2,8,17,5,0.022667

1,2,8,17,6,0.018601

1,2,8,17,7,0.024444

1,2,8,17,8,-0.0029941

1,2,8,18,1,0.05331

1,2,8,18,2,0.021758

1,2,8,18,3,-0.056124

1,2,8,18,4,-0.041034

1,2,8,18,5,0.01439

1,2,8,18,6,0.016588

1,2,8,18,7,0.027873

1,2,8,18,8,-0.012921

1,2,8,19,1,0.024219

1,2,8,19,2,-0.016639

1,2,8,19,3,-0.060293

1,2,8,19,4,-0.03337

1,2,8,19,5,-0.004532

1,2,8,19,6,0.010272

1,2,8,19,7,0.0097123

1,2,8,19,8,-0.017424

1,2,8,20,1,0.020301

1,2,8,20,2,0.036609

1,2,8,20,3,-0.049588

1,2,8,20,4,-0.061853

1,2,8,20,5,-0.0014353

1,2,8,20,6,0.00039944

1,2,8,20,7,0.0082215

1,2,8,20,8,-0.027966

1,2,9,9,1,0.051696

1,2,9,9,2,-0.040491

1,2,9,9,3,-0.01802

1,2,9,9,4,-0.024731

1,2,9,9,5,-0.01176

1,2,9,9,6,0.0062342

1,2,9,9,7,0.0096802

1,2,9,9,8,-0.0056956

1,2,9,10,1,0.03397

1,2,9,10,2,-0.0078299

1,2,9,10,3,-0.056805

1,2,9,10,4,-0.041519

1,2,9,10,5,-0.019756

1,2,9,10,6,0.016811

1,2,9,10,7,0.0091721

1,2,9,10,8,-0.0044631

1,2,9,11,1,0.035009

1,2,9,11,2,0.0093968

1,2,9,11,3,-0.056652

1,2,9,11,4,-0.047065

1,2,9,11,5,-0.02105

1,2,9,11,6,0.013588

1,2,9,11,7,0.0012609

1,2,9,11,8,-0.0068055

1,2,9,12,1,0.023487

1,2,9,12,2,0.024717

1,2,9,12,3,-0.043535

1,2,9,12,4,-0.032418

1,2,9,12,5,0.00028187

1,2,9,12,6,0.0065187

1,2,9,12,7,0.024698

1,2,9,12,8,-0.011858

1,2,9,13,1,0.064073

1,2,9,13,2,0.0011369

1,2,9,13,3,-0.044741

1,2,9,13,4,-0.054064

1,2,9,13,5,-0.008122

1,2,9,13,6,0.019498

1,2,9,13,7,0.027735

1,2,9,13,8,0.0072399

1,2,9,14,1,0.043307

1,2,9,14,2,-0.029431

1,2,9,14,3,-0.079866

1,2,9,14,4,-0.078931

1,2,9,14,5,-0.030553

1,2,9,14,6,0.0042619

1,2,9,14,7,0.011069

1,2,9,14,8,-0.008628

1,2,9,15,1,0.021286

1,2,9,15,2,0.030536

1,2,9,15,3,-0.038002

1,2,9,15,4,-0.027186

1,2,9,15,5,-0.0069514

1,2,9,15,6,0.0078214

1,2,9,15,7,0.034251

1,2,9,15,8,-0.013496

1,2,9,16,1,0.028676

1,2,9,16,2,-0.021327

1,2,9,16,3,-0.025596

1,2,9,16,4,-0.010333

1,2,9,16,5,-0.031226

1,2,9,16,6,0.0215

1,2,9,16,7,0.00081885

1,2,9,16,8,0.013761

1,2,9,17,1,0.10276

1,2,9,17,2,0.027181

1,2,9,17,3,-0.055281

1,2,9,17,4,-0.045712

1,2,9,17,5,-0.0059899

1,2,9,17,6,0.002853

1,2,9,17,7,0.028537

1,2,9,17,8,-0.0093094

1,2,9,18,1,0.026288

1,2,9,18,2,-0.014446

1,2,9,18,3,-0.064715

1,2,9,18,4,-0.057469

1,2,9,18,5,-0.02266

1,2,9,18,6,0.0065984

1,2,9,18,7,0.018154

1,2,9,18,8,-0.01038

1,2,9,19,1,0.014972

1,2,9,19,2,-0.012178

1,2,9,19,3,-0.067144

1,2,9,19,4,-0.056907

1,2,9,19,5,-0.018563

1,2,9,19,6,0.0047428

1,2,9,19,7,0.0097951

1,2,9,19,8,-0.016387

1,2,9,20,1,0.0094854

1,2,9,20,2,-0.0080659

1,2,9,20,3,-0.04941

1,2,9,20,4,-0.031982

1,2,9,20,5,-0.028295

1,2,9,20,6,-0.0024492

1,2,9,20,7,-0.0078227

1,2,9,20,8,-0.016945

1,2,10,10,1,0.07013

1,2,10,10,2,-0.0057779

1,2,10,10,3,-0.011185

1,2,10,10,4,-0.0085673

1,2,10,10,5,0.0087071

1,2,10,10,6,0.015743

1,2,10,10,7,0.0059847

1,2,10,10,8,0.0070977

1,2,10,11,1,0.061216

1,2,10,11,2,0.025663

1,2,10,11,3,-0.018814

1,2,10,11,4,-0.040063

1,2,10,11,5,0.0061672

1,2,10,11,6,0.025317

1,2,10,11,7,0.030216

1,2,10,11,8,0.0025412

1,2,10,12,1,0.024766

1,2,10,12,2,0.014768

1,2,10,12,3,-0.058427

1,2,10,12,4,-0.048238

1,2,10,12,5,-0.0067903

1,2,10,12,6,0.0097851

1,2,10,12,7,0.0014345

1,2,10,12,8,-0.01869

1,2,10,13,1,0.061035

1,2,10,13,2,0.038807

1,2,10,13,3,-0.067392

1,2,10,13,4,-0.037068

1,2,10,13,5,-0.0044896

1,2,10,13,6,0.013391

1,2,10,13,7,0.019625

1,2,10,13,8,-0.008427

1,2,10,14,1,0.059786

1,2,10,14,2,0.016273

1,2,10,14,3,-0.051328

1,2,10,14,4,-0.033

1,2,10,14,5,0.0045511

1,2,10,14,6,0.022023

1,2,10,14,7,0.02799

1,2,10,14,8,-0.0085793

1,2,10,15,1,0.031107

1,2,10,15,2,0.025123

1,2,10,15,3,-0.031932

1,2,10,15,4,-0.053014

1,2,10,15,5,0.017284

1,2,10,15,6,0.02363

1,2,10,15,7,0.0057676

1,2,10,15,8,-9.2618e-05

1,2,10,16,1,0.021256

1,2,10,16,2,0.016256

1,2,10,16,3,-0.03934

1,2,10,16,4,-0.025198

1,2,10,16,5,-0.0074282

1,2,10,16,6,0.004569

1,2,10,16,7,-0.0036698

1,2,10,16,8,-0.020049

1,2,10,17,1,0.10326

1,2,10,17,2,0.065769

1,2,10,17,3,-0.060157

1,2,10,17,4,-0.03997

1,2,10,17,5,0.0065689

1,2,10,17,6,0.021917

1,2,10,17,7,0.017415

1,2,10,17,8,-0.0035732

1,2,10,18,1,0.032393

1,2,10,18,2,0.0092157

1,2,10,18,3,-0.062371

1,2,10,18,4,-0.067762

1,2,10,18,5,-0.012007

1,2,10,18,6,3.971e-06

1,2,10,18,7,0.00062635

1,2,10,18,8,-0.015871

1,2,10,19,1,0.056164

1,2,10,19,2,-0.01441

1,2,10,19,3,-0.085578

1,2,10,19,4,-0.059941

1,2,10,19,5,-0.027507

1,2,10,19,6,0.00047903

1,2,10,19,7,0.0020755

1,2,10,19,8,-0.012486

1,2,10,20,1,0.015747

1,2,10,20,2,-0.032405

1,2,10,20,3,-0.062607

1,2,10,20,4,-0.070143

1,2,10,20,5,-0.018792

1,2,10,20,6,-0.0014492

1,2,10,20,7,-0.0041315

1,2,10,20,8,0.0020341

1,2,11,11,1,0.067832

1,2,11,11,2,0.017923

1,2,11,11,3,-0.010981

1,2,11,11,4,-0.022522

1,2,11,11,5,0.014102

1,2,11,11,6,0.019879

1,2,11,11,7,0.006389

1,2,11,11,8,0.0040538

1,2,11,12,1,0.017519

1,2,11,12,2,-0.014901

1,2,11,12,3,-0.053647

1,2,11,12,4,-0.068695

1,2,11,12,5,-0.0082506

1,2,11,12,6,-0.0056638

1,2,11,12,7,0.0080664

1,2,11,12,8,-0.018948

1,2,11,13,1,0.043615

1,2,11,13,2,-0.010928

1,2,11,13,3,-0.036142

1,2,11,13,4,-0.051858

1,2,11,13,5,-0.0053864

1,2,11,13,6,0.021369

1,2,11,13,7,0.032786

1,2,11,13,8,-0.012803

1,2,11,14,1,0.054047

1,2,11,14,2,0.034658

1,2,11,14,3,-0.051713

1,2,11,14,4,-0.044196

1,2,11,14,5,-0.009072

1,2,11,14,6,0.027939

1,2,11,14,7,0.02685

1,2,11,14,8,-0.014254

1,2,11,15,1,0.020023

1,2,11,15,2,0.019478

1,2,11,15,3,-0.041886

1,2,11,15,4,-0.056433

1,2,11,15,5,0.0087293

1,2,11,15,6,0.011801

1,2,11,15,7,0.015553

1,2,11,15,8,-0.010037

1,2,11,16,1,0.02528

1,2,11,16,2,0.0043249

1,2,11,16,3,-0.040364

1,2,11,16,4,-0.042674

1,2,11,16,5,-0.0037496

1,2,11,16,6,0.0027832

1,2,11,16,7,0.0073351

1,2,11,16,8,-0.019251

1,2,11,17,1,0.084499

1,2,11,17,2,0.045418

1,2,11,17,3,-0.071198

1,2,11,17,4,-0.04437

1,2,11,17,5,-0.0010612

1,2,11,17,6,0.0085843

1,2,11,17,7,0.0050663

1,2,11,17,8,-0.015339

1,2,11,18,1,0.026859

1,2,11,18,2,0.0035045

1,2,11,18,3,-0.090722

1,2,11,18,4,-0.059594

1,2,11,18,5,-0.033564

1,2,11,18,6,0.016328

1,2,11,18,7,0.0062746

1,2,11,18,8,-0.019983

1,2,11,19,1,0.031867

1,2,11,19,2,-0.013013

1,2,11,19,3,-0.084785

1,2,11,19,4,-0.053256

1,2,11,19,5,-0.027466

1,2,11,19,6,0.0079845

1,2,11,19,7,0.0030347

1,2,11,19,8,-0.010982

1,2,11,20,1,0.013376

1,2,11,20,2,-0.015067

1,2,11,20,3,-0.073162

1,2,11,20,4,-0.070875

1,2,11,20,5,-0.021246

1,2,11,20,6,0.021416

1,2,11,20,7,-0.0013765

1,2,11,20,8,-0.028091

1,2,12,12,1,0.016745

1,2,12,12,2,0.011935

1,2,12,12,3,-0.03952

1,2,12,12,4,-0.013306

1,2,12,12,5,-0.016527

1,2,12,12,6,0.0041714

1,2,12,12,7,-0.0071461

1,2,12,12,8,-0.0068215

1,2,12,13,1,0.0087019

1,2,12,13,2,-0.01561

1,2,12,13,3,-0.058815

1,2,12,13,4,-0.036664

1,2,12,13,5,-0.00042581

1,2,12,13,6,0.019014

1,2,12,13,7,0.017768

1,2,12,13,8,-0.0078068

1,2,12,14,1,0.019202

1,2,12,14,2,0.029018

1,2,12,14,3,-0.0411

1,2,12,14,4,-0.040396

1,2,12,14,5,0.026611

1,2,12,14,6,0.025269

1,2,12,14,7,0.011494

1,2,12,14,8,-0.017672

1,2,12,15,1,0.015015

1,2,12,15,2,0.0023192

1,2,12,15,3,-0.047626

1,2,12,15,4,-0.061934

1,2,12,15,5,-0.006603

1,2,12,15,6,0.0086247

1,2,12,15,7,0.0088577

1,2,12,15,8,-0.019869

1,2,12,16,1,0.019924

1,2,12,16,2,0.02887

1,2,12,16,3,-0.041124

1,2,12,16,4,-0.051554

1,2,12,16,5,-0.025222

1,2,12,16,6,-0.02117

1,2,12,16,7,-0.020855

1,2,12,16,8,-0.019895

1,2,12,17,1,0.044872

1,2,12,17,2,0.06098

1,2,12,17,3,-0.030508

1,2,12,17,4,-0.03744

1,2,12,17,5,0.0061509

1,2,12,17,6,0.013936

1,2,12,17,7,0.018151

1,2,12,17,8,0.0010227

1,2,12,18,1,0.016863

1,2,12,18,2,0.0036714

1,2,12,18,3,-0.07434

1,2,12,18,4,-0.065681

1,2,12,18,5,-0.0058744

1,2,12,18,6,-0.0051324

1,2,12,18,7,-0.0014404

1,2,12,18,8,-0.02978

1,2,12,19,1,0.020542

1,2,12,19,2,-0.0034973

1,2,12,19,3,-0.04756

1,2,12,19,4,-0.027595

1,2,12,19,5,0.0016068

1,2,12,19,6,0.0038958

1,2,12,19,7,0.0016815

1,2,12,19,8,-0.01865

1,2,12,20,1,0.0093565

1,2,12,20,2,0.008869

1,2,12,20,3,-0.023919

1,2,12,20,4,-0.038821

1,2,12,20,5,-0.008486

1,2,12,20,6,-0.021503

1,2,12,20,7,-0.00062742

1,2,12,20,8,-0.036127

1,2,13,13,1,0.075351

1,2,13,13,2,-0.056146

1,2,13,13,3,-0.065603

1,2,13,13,4,-0.052443

1,2,13,13,5,-0.028778

1,2,13,13,6,-0.023718

1,2,13,13,7,0.001875

1,2,13,13,8,-0.014257

1,2,13,14,1,0.058534

1,2,13,14,2,-0.085786

1,2,13,14,3,-0.12066

1,2,13,14,4,-0.10215

1,2,13,14,5,-0.050906

1,2,13,14,6,-0.029418

1,2,13,14,7,-0.0011681

1,2,13,14,8,-0.023179

1,2,13,15,1,0.020204

1,2,13,15,2,-0.0072803

1,2,13,15,3,-0.088239

1,2,13,15,4,-0.058709

1,2,13,15,5,-0.027449

1,2,13,15,6,-0.015982

1,2,13,15,7,0.0071695

1,2,13,15,8,-0.031975

1,2,13,16,1,0.013835

1,2,13,16,2,-0.027385

1,2,13,16,3,-0.068309

1,2,13,16,4,-0.055413

1,2,13,16,5,-0.022105

1,2,13,16,6,-0.015912

1,2,13,16,7,0.0086269

1,2,13,16,8,-0.0069089

1,2,13,17,1,0.11213

1,2,13,17,2,-0.043924

1,2,13,17,3,-0.09689

1,2,13,17,4,-0.089086

1,2,13,17,5,-0.051492

1,2,13,17,6,-0.033841

1,2,13,17,7,-0.0050485

1,2,13,17,8,-0.034074

1,2,13,18,1,0.033451

1,2,13,18,2,-0.062246

1,2,13,18,3,-0.10409

1,2,13,18,4,-0.072594

1,2,13,18,5,-0.059712

1,2,13,18,6,-0.029644

1,2,13,18,7,0.0027883

1,2,13,18,8,-0.0353

1,2,13,19,1,0.024867

1,2,13,19,2,-0.025437

1,2,13,19,3,-0.11411

1,2,13,19,4,-0.065038

1,2,13,19,5,-0.038729

1,2,13,19,6,-0.013352

1,2,13,19,7,0.0094098

1,2,13,19,8,-0.014371

1,2,13,20,1,0.017081

1,2,13,20,2,-0.034892

1,2,13,20,3,-0.073835

1,2,13,20,4,-0.069635

1,2,13,20,5,-0.047732

1,2,13,20,6,-0.016372

1,2,13,20,7,0.0057812

1,2,13,20,8,-0.017953

1,2,14,14,1,0.04193

1,2,14,14,2,-0.056485

1,2,14,14,3,-0.079104

1,2,14,14,4,-0.063761

1,2,14,14,5,-0.04153

1,2,14,14,6,-0.023841

1,2,14,14,7,-0.0063928

1,2,14,14,8,-0.018521

1,2,14,15,1,0.01485

1,2,14,15,2,-0.022888

1,2,14,15,3,-0.084939

1,2,14,15,4,-0.093445

1,2,14,15,5,-0.052002

1,2,14,15,6,-0.029356

1,2,14,15,7,-0.0046903

1,2,14,15,8,-0.026458

1,2,14,16,1,0.016892

1,2,14,16,2,-0.0051754

1,2,14,16,3,-0.040957

1,2,14,16,4,-0.024884

1,2,14,16,5,-0.03541

1,2,14,16,6,-0.028673

1,2,14,16,7,0.0059523

1,2,14,16,8,-0.029755

1,2,14,17,1,0.059309

1,2,14,17,2,-0.063059

1,2,14,17,3,-0.14386

1,2,14,17,4,-0.1214

1,2,14,17,5,-0.066418

1,2,14,17,6,-0.060937

1,2,14,17,7,-0.017128

1,2,14,17,8,-0.040935

1,2,14,18,1,0.018208

1,2,14,18,2,-0.044439

1,2,14,18,3,-0.14028

1,2,14,18,4,-0.094473

1,2,14,18,5,-0.065865

1,2,14,18,6,-0.037449

1,2,14,18,7,-0.016982

1,2,14,18,8,-0.038598

1,2,14,19,1,0.015008

1,2,14,19,2,-0.059483

1,2,14,19,3,-0.12206

1,2,14,19,4,-0.090807

1,2,14,19,5,-0.060211

1,2,14,19,6,-0.020572

1,2,14,19,7,0.0017242

1,2,14,19,8,-0.021605

1,2,14,20,1,0.018321

1,2,14,20,2,-0.0052609

1,2,14,20,3,-0.060361

1,2,14,20,4,-0.070038

1,2,14,20,5,-0.04835

1,2,14,20,6,-0.026664

1,2,14,20,7,-0.014275

1,2,14,20,8,-0.016709

1,2,15,15,1,0.0094487

1,2,15,15,2,-0.0079647

1,2,15,15,3,-0.051031

1,2,15,15,4,-0.03549

1,2,15,15,5,-0.021696

1,2,15,15,6,-0.0074559

1,2,15,15,7,0.00028371

1,2,15,15,8,-0.0041843

1,2,15,16,1,0.0027604

1,2,15,16,2,-0.0029492

1,2,15,16,3,-0.026093

1,2,15,16,4,-0.029654

1,2,15,16,5,-0.019204

1,2,15,16,6,-0.011492

1,2,15,16,7,0.0095115

1,2,15,16,8,-0.018723

1,2,15,17,1,0.029331

1,2,15,17,2,-0.0099624

1,2,15,17,3,-0.09539

1,2,15,17,4,-0.080837

1,2,15,17,5,-0.050652

1,2,15,17,6,-0.033524

1,2,15,17,7,-0.016907

1,2,15,17,8,-0.042977

1,2,15,18,1,7.6763e-08

1,2,15,18,2,-0.021536

1,2,15,18,3,-0.054317

1,2,15,18,4,-0.077481

1,2,15,18,5,-0.046425

1,2,15,18,6,-0.02953

1,2,15,18,7,-0.019459

1,2,15,18,8,-0.042344

1,2,15,19,1,0.010691

1,2,15,19,2,-0.0068882

1,2,15,19,3,-0.064751

1,2,15,19,4,-0.051601

1,2,15,19,5,-0.021817

1,2,15,19,6,-0.0058468

1,2,15,19,7,-0.0099308

1,2,15,19,8,-0.024584

1,2,15,20,1,0.00038568

1,2,15,20,2,-0.0026652

1,2,15,20,3,-0.033275

1,2,15,20,4,-0.056222

1,2,15,20,5,-0.035315

1,2,15,20,6,-0.018621

1,2,15,20,7,-0.0083526

1,2,15,20,8,-0.0011183

1,2,16,16,1,1.1256e-10

1,2,16,16,2,-0.10159

1,2,16,16,3,-0.067455

1,2,16,16,4,-0.083747

1,2,16,16,5,-0.063966

1,2,16,16,6,-0.047591

1,2,16,16,7,-0.019477

1,2,16,16,8,0.0062658

1,2,16,17,1,0.019776

1,2,16,17,2,0.0048691

1,2,16,17,3,-0.076402

1,2,16,17,4,-0.062582

1,2,16,17,5,-0.020914

1,2,16,17,6,-0.020876

1,2,16,17,7,0.013719

1,2,16,17,8,-0.022211

1,2,16,18,1,0.0039447

1,2,16,18,2,-0.04605

1,2,16,18,3,-0.10238

1,2,16,18,4,-0.052845

1,2,16,18,5,-0.05408

1,2,16,18,6,-0.016042

1,2,16,18,7,-0.00091313

1,2,16,18,8,-0.019138

1,2,16,19,1,0.01607

1,2,16,19,2,0.024316

1,2,16,19,3,-0.059925

1,2,16,19,4,-0.048813

1,2,16,19,5,-0.033867

1,2,16,19,6,-0.021411

1,2,16,19,7,-0.0012478

1,2,16,19,8,-0.01791

1,2,16,20,1,0.0052267

1,2,16,20,2,0.0076274

1,2,16,20,3,-0.050849

1,2,16,20,4,-0.028813

1,2,16,20,5,-0.014094

1,2,16,20,6,0.00037069

1,2,16,20,7,-0.0045982

1,2,16,20,8,-0.0066317

1,2,17,17,1,0.11132

1,2,17,17,2,-0.015125

1,2,17,17,3,-0.04931

1,2,17,17,4,-0.052797

1,2,17,17,5,-0.025627

1,2,17,17,6,-0.026921

1,2,17,17,7,-0.0094525

1,2,17,17,8,-0.021758

1,2,17,18,1,0.044038

1,2,17,18,2,-0.036329

1,2,17,18,3,-0.10997

1,2,17,18,4,-0.089564

1,2,17,18,5,-0.056249

1,2,17,18,6,-0.026871

1,2,17,18,7,-0.017269

1,2,17,18,8,-0.033002

1,2,17,19,1,0.022892

1,2,17,19,2,-0.035232

1,2,17,19,3,-0.1051

1,2,17,19,4,-0.078272

1,2,17,19,5,-0.063809

1,2,17,19,6,-0.027797

1,2,17,19,7,-0.014306

1,2,17,19,8,-0.028811

1,2,17,20,1,0.016101

1,2,17,20,2,-0.054632

1,2,17,20,3,-0.088211

1,2,17,20,4,-0.090125

1,2,17,20,5,-0.057612

1,2,17,20,6,-0.045753

1,2,17,20,7,-0.019047

1,2,17,20,8,-0.049998

1,2,18,18,1,0.044824

1,2,18,18,2,-0.043912

1,2,18,18,3,-0.065084

1,2,18,18,4,-0.04955

1,2,18,18,5,-0.042203

1,2,18,18,6,-0.025498

1,2,18,18,7,-0.004419

1,2,18,18,8,-0.021263

1,2,18,19,1,0.018258

1,2,18,19,2,-0.017222

1,2,18,19,3,-0.073658

1,2,18,19,4,-0.084819

1,2,18,19,5,-0.045756

1,2,18,19,6,-0.042313

1,2,18,19,7,-0.014665

1,2,18,19,8,-0.038447

1,2,18,20,1,0.0013688

1,2,18,20,2,-0.031384

1,2,18,20,3,-0.094946

1,2,18,20,4,-0.061006

1,2,18,20,5,-0.048528

1,2,18,20,6,-0.025407

1,2,18,20,7,0.0062317

1,2,18,20,8,-0.038381

1,2,19,19,1,0.025356

1,2,19,19,2,-0.050528

1,2,19,19,3,-0.066706

1,2,19,19,4,-0.055422

1,2,19,19,5,-0.042996

1,2,19,19,6,-0.015968

1,2,19,19,7,0.0081149

1,2,19,19,8,-0.010323

1,2,19,20,1,0.011482

1,2,19,20,2,-0.022713

1,2,19,20,3,-0.086862

1,2,19,20,4,-0.060927

1,2,19,20,5,-0.038792

1,2,19,20,6,-0.013108

1,2,19,20,7,-0.00067853

1,2,19,20,8,-0.032411

1,2,20,20,1,0.0062206

1,2,20,20,2,-0.03331

1,2,20,20,3,-0.054647

1,2,20,20,4,-0.030887

1,2,20,20,5,-0.029781

1,2,20,20,6,-0.016868

1,2,20,20,7,-0.0060534

1,2,20,20,8,-0.033406

1,3,1,1,1,0.0035111

1,3,1,1,2,-0.0014474

1,3,1,1,3,-0.00065475

1,3,1,1,4,-0.0014586

1,3,1,1,5,0.00048518

1,3,1,1,6,0.00035704

1,3,1,1,7,0.00025403

1,3,1,1,8,-0.00056634

1,3,1,2,1,0.0016194

1,3,1,2,2,-0.0022564

1,3,1,2,3,-0.0002445

1,3,1,2,4,0.0011318

1,3,1,2,5,0.0017882

1,3,1,2,6,0.0015725

1,3,1,2,7,0.00061445

1,3,1,2,8,0.00081719

1,3,1,3,1,0.0030466

1,3,1,3,2,-0.0006258

1,3,1,3,3,-0.0058302

1,3,1,3,4,-0.0015337

1,3,1,3,5,0.00042219

1,3,1,3,6,-0.00039765

1,3,1,3,7,9.5636e-05

1,3,1,3,8,-0.00093195

1,3,1,4,1,0.0031036

1,3,1,4,2,-0.00031594

1,3,1,4,3,-0.0034919

1,3,1,4,4,-0.00018377

1,3,1,4,5,0.00011537

1,3,1,4,6,0.0017575

1,3,1,4,7,-0.00011685

1,3,1,4,8,-0.00060029

1,3,1,5,1,0.0029326

1,3,1,5,2,-0.0036318

1,3,1,5,3,-0.0024375

1,3,1,5,4,-0.0016605

1,3,1,5,5,0.0013227

1,3,1,5,6,0.00029074

1,3,1,5,7,0.00043502

1,3,1,5,8,-0.0003486

1,3,1,6,1,0.0016022

1,3,1,6,2,-0.00051492

1,3,1,6,3,-0.0034571

1,3,1,6,4,-0.0033635

1,3,1,6,5,0.0014002

1,3,1,6,6,-0.0008382

1,3,1,6,7,0.00038734

1,3,1,6,8,-0.0014589

1,3,1,7,1,0.002451

1,3,1,7,2,-0.001452

1,3,1,7,3,-0.004148

1,3,1,7,4,-0.0029085

1,3,1,7,5,0.00067312

1,3,1,7,6,0.00075614

1,3,1,7,7,-0.00017939

1,3,1,7,8,-0.00099197

1,3,1,8,1,0.0040032

1,3,1,8,2,-0.0011549

1,3,1,8,3,-0.0013006

1,3,1,8,4,-0.00055788

1,3,1,8,5,0.0016372

1,3,1,8,6,-5.7179e-06

1,3,1,8,7,0.0015229

1,3,1,8,8,-0.00079875

1,3,1,9,1,0.0020967

1,3,1,9,2,-0.0017197

1,3,1,9,3,-0.004088

1,3,1,9,4,-0.001645

1,3,1,9,5,0.00083244

1,3,1,9,6,0.0012624

1,3,1,9,7,4.3172e-05

1,3,1,9,8,-0.00046232

1,3,1,10,1,0.0049784

1,3,1,10,2,-0.0032984

1,3,1,10,3,-0.0035293

1,3,1,10,4,-0.0015203

1,3,1,10,5,0.00068835

1,3,1,10,6,0.00093905

1,3,1,10,7,0.00089734

1,3,1,10,8,-0.0013736

1,3,1,11,1,0.0058023

1,3,1,11,2,0.0019581

1,3,1,11,3,-0.0037731

1,3,1,11,4,-0.0033922

1,3,1,11,5,0.00094399

1,3,1,11,6,0.00028968

1,3,1,11,7,0.0010628

1,3,1,11,8,-0.00047611

1,3,1,12,1,8.437e-12

1,3,1,12,2,-0.003257

1,3,1,12,3,-0.004821

1,3,1,12,4,-0.0024737

1,3,1,12,5,0.00051956

1,3,1,12,6,0.0010423

1,3,1,12,7,0.0007049

1,3,1,12,8,-0.00048002

1,3,1,13,1,0.0048889

1,3,1,13,2,-0.00070429

1,3,1,13,3,-0.0031991

1,3,1,13,4,-0.00075426

1,3,1,13,5,0.00070417

1,3,1,13,6,0.0028043

1,3,1,13,7,0.00020496

1,3,1,13,8,-0.00059479

1,3,1,14,1,0.0054258

1,3,1,14,2,-0.0015414

1,3,1,14,3,-0.0024288

1,3,1,14,4,-0.00010493

1,3,1,14,5,0.0017871

1,3,1,14,6,0.0020728

1,3,1,14,7,0.00022275

1,3,1,14,8,-0.00064165

1,3,1,15,1,0.0012255

1,3,1,15,2,-0.0028371

1,3,1,15,3,-0.002362

1,3,1,15,4,-0.00055325

1,3,1,15,5,-0.00015518

1,3,1,15,6,0.0014383

1,3,1,15,7,0.00058173

1,3,1,15,8,-0.00066761

1,3,1,16,1,0.0022357

1,3,1,16,2,0.0017696

1,3,1,16,3,-0.0021299

1,3,1,16,4,-0.0010868

1,3,1,16,5,0.0022607

1,3,1,16,6,0.0017923

1,3,1,16,7,0.00017369

1,3,1,16,8,-0.0018971

1,3,1,17,1,0.0062404

1,3,1,17,2,0.0012358

1,3,1,17,3,-0.001653

1,3,1,17,4,-0.00094018

1,3,1,17,5,0.0012295

1,3,1,17,6,0.0017143

1,3,1,17,7,0.00066709

1,3,1,17,8,-0.00070188

1,3,1,18,1,0.0027985

1,3,1,18,2,0.0010619

1,3,1,18,3,-0.0032886

1,3,1,18,4,-0.0020277

1,3,1,18,5,0.0018725

1,3,1,18,6,0.00060106

1,3,1,18,7,0.001643

1,3,1,18,8,-0.00078873

1,3,1,19,1,0.0047948

1,3,1,19,2,0.0028595

1,3,1,19,3,-0.0016707

1,3,1,19,4,-0.0020254

1,3,1,19,5,0.00058671

1,3,1,19,6,0.0020213

1,3,1,19,7,0.00079922

1,3,1,19,8,-0.00040509

1,3,1,20,1,0.0017015

1,3,1,20,2,0.00088699

1,3,1,20,3,-0.002572

1,3,1,20,4,0.00013158

1,3,1,20,5,0.00041216

1,3,1,20,6,0.0015391

1,3,1,20,7,0.00058753

1,3,1,20,8,-0.00068219

1,3,2,2,1,0.0011076

1,3,2,2,2,-0.00082964

1,3,2,2,3,-0.001974

1,3,2,2,4,-6.713e-05

1,3,2,2,5,-0.0016184

1,3,2,2,6,-0.00054215

1,3,2,2,7,-0.0011819

1,3,2,2,8,-9.2141e-05

1,3,2,3,1,3.5777e-11

1,3,2,3,2,0.0020513

1,3,2,3,3,-0.0024938

1,3,2,3,4,0.00036809

1,3,2,3,5,-0.00037268

1,3,2,3,6,0.00064482

1,3,2,3,7,-0.0015809

1,3,2,3,8,7.0916e-05

1,3,2,4,1,0.0012374

1,3,2,4,2,-0.0002076

1,3,2,4,3,-0.0042478

1,3,2,4,4,-0.0014013

1,3,2,4,5,-0.0020623

1,3,2,4,6,-0.0011773

1,3,2,4,7,2.1079e-05

1,3,2,4,8,-0.0019728

1,3,2,5,1,0.00034819

1,3,2,5,2,-0.0023258

1,3,2,5,3,-0.0041405

1,3,2,5,4,-0.0024369

1,3,2,5,5,-0.00070809

1,3,2,5,6,0.00024364

1,3,2,5,7,-0.00091976

1,3,2,5,8,-0.0015576

1,3,2,6,1,1.3854e-11

1,3,2,6,2,-0.0033055

1,3,2,6,3,-0.0048792

1,3,2,6,4,0.00013322

1,3,2,6,5,-0.00047097

1,3,2,6,6,0.00059942

1,3,2,6,7,0.00081185

1,3,2,6,8,-0.00056851

1,3,2,7,1,0.00032174

1,3,2,7,2,-0.00082808

1,3,2,7,3,-0.0012943

1,3,2,7,4,-0.0032279

1,3,2,7,5,-0.00071226

1,3,2,7,6,0.00013339

1,3,2,7,7,-0.00088365

1,3,2,7,8,-0.0015616

1,3,2,8,1,0.00077137

1,3,2,8,2,0.0010807

1,3,2,8,3,-0.0053922

1,3,2,8,4,-0.00048836

1,3,2,8,5,-0.0017533

1,3,2,8,6,0.00012974

1,3,2,8,7,-0.00082624

1,3,2,8,8,-0.00058504

1,3,2,9,1,1.3169e-11

1,3,2,9,2,-0.0015222

1,3,2,9,3,-0.0038152

1,3,2,9,4,5.7281e-05

1,3,2,9,5,-0.0021884

1,3,2,9,6,0.0016917

1,3,2,9,7,0.00017234

1,3,2,9,8,-0.0011923

1,3,2,10,1,8.241e-12

1,3,2,10,2,-0.00053731

1,3,2,10,3,-0.00040865

1,3,2,10,4,-0.0012608

1,3,2,10,5,0.0015533

1,3,2,10,6,-0.0010504

1,3,2,10,7,0.00091204

1,3,2,10,8,-0.00086048

1,3,2,11,1,0.00075471

1,3,2,11,2,-0.0048383

1,3,2,11,3,-0.0032171

1,3,2,11,4,-0.0021739

1,3,2,11,5,-0.00061641

1,3,2,11,6,0.00027783

1,3,2,11,7,0.00055019

1,3,2,11,8,-0.0027487

1,3,2,12,1,7.1155e-11

1,3,2,12,2,-0.001361

1,3,2,12,3,-0.0017006

1,3,2,12,4,-0.0035493

1,3,2,12,5,-0.0022577

1,3,2,12,6,-0.00081181

1,3,2,12,7,-0.0018013

1,3,2,12,8,-0.00089615

1,3,2,13,1,1.7897e-05

1,3,2,13,2,-0.004335

1,3,2,13,3,-0.0042319

1,3,2,13,4,-0.004524

1,3,2,13,5,-0.00049155

1,3,2,13,6,0.0013933

1,3,2,13,7,0.0006231

1,3,2,13,8,-0.00060778

1,3,2,14,1,5.2656e-11

1,3,2,14,2,-0.004986

1,3,2,14,3,-0.0047655

1,3,2,14,4,-0.0021714

1,3,2,14,5,-0.0019293

1,3,2,14,6,0.0018276

1,3,2,14,7,-0.0010769

1,3,2,14,8,-0.001608

1,3,2,15,1,0.0012668

1,3,2,15,2,-0.0012339

1,3,2,15,3,-0.0042069

1,3,2,15,4,-0.0020471

1,3,2,15,5,-0.00037033

1,3,2,15,6,0.0014429

1,3,2,15,7,-0.0017936

1,3,2,15,8,-0.0015759

1,3,2,16,1,0.0010016

1,3,2,16,2,0.0011061

1,3,2,16,3,-0.003662

1,3,2,16,4,0.00055061

1,3,2,16,5,-8.9258e-05

1,3,2,16,6,0.0010408

1,3,2,16,7,-0.0012703

1,3,2,16,8,-0.00021297

1,3,2,17,1,1.7372e-11

1,3,2,17,2,0.0015502

1,3,2,17,3,-0.00077852

1,3,2,17,4,-0.00022406

1,3,2,17,5,-8.4435e-05

1,3,2,17,6,0.0011068

1,3,2,17,7,0.00052266

1,3,2,17,8,-0.0013435

1,3,2,18,1,5.0352e-08

1,3,2,18,2,-0.0012155

1,3,2,18,3,-0.00093141

1,3,2,18,4,-0.003232

1,3,2,18,5,-0.0018283

1,3,2,18,6,-0.00043386

1,3,2,18,7,-0.00077664

1,3,2,18,8,-0.0020492

1,3,2,19,1,3.1345e-12

1,3,2,19,2,-0.0015799

1,3,2,19,3,-0.0026049

1,3,2,19,4,0.00053826

1,3,2,19,5,0.00011812

1,3,2,19,6,0.00076681

1,3,2,19,7,0.00023122

1,3,2,19,8,-0.0016597

1,3,2,20,1,6.1639e-12

1,3,2,20,2,-7.7177e-05

1,3,2,20,3,-0.0050523

1,3,2,20,4,-0.0044986

1,3,2,20,5,-0.0032921

1,3,2,20,6,-0.0019734

1,3,2,20,7,-0.0035787

1,3,2,20,8,-0.0022068

1,3,3,3,1,0.0011666

1,3,3,3,2,-0.0018279

1,3,3,3,3,-0.0028956

1,3,3,3,4,-0.0006116

1,3,3,3,5,0.0007296

1,3,3,3,6,0.00016732

1,3,3,3,7,-0.00018724

1,3,3,3,8,-0.00098883

1,3,3,4,1,0.0042951

1,3,3,4,2,-0.0017431

1,3,3,4,3,-0.0021952

1,3,3,4,4,-0.00032733

1,3,3,4,5,0.0026997

1,3,3,4,6,0.0015543

1,3,3,4,7,0.0020642

1,3,3,4,8,-0.00010029

1,3,3,5,1,0.0041572

1,3,3,5,2,-0.0019194

1,3,3,5,3,-0.0037278

1,3,3,5,4,-0.0017731

1,3,3,5,5,0.0014125

1,3,3,5,6,0.0014782

1,3,3,5,7,0.00092782

1,3,3,5,8,-0.0012232

1,3,3,6,1,0.00295

1,3,3,6,2,-0.00055364

1,3,3,6,3,-0.0057588

1,3,3,6,4,-0.0022915

1,3,3,6,5,0.00062842

1,3,3,6,6,0.00057032

1,3,3,6,7,0.0005557

1,3,3,6,8,-0.00054444

1,3,3,7,1,0.0035402

1,3,3,7,2,-0.0023902

1,3,3,7,3,-0.0019352

1,3,3,7,4,-0.0015228

1,3,3,7,5,0.0027473

1,3,3,7,6,0.0024299

1,3,3,7,7,0.00078743

1,3,3,7,8,0.00040267

1,3,3,8,1,0.0044379

1,3,3,8,2,-0.0042797

1,3,3,8,3,-0.0080044

1,3,3,8,4,-0.0015023

1,3,3,8,5,-0.00058804

1,3,3,8,6,0.00024444

1,3,3,8,7,0.00092255

1,3,3,8,8,-0.0019154

1,3,3,9,1,0.0031744

1,3,3,9,2,-0.00052213

1,3,3,9,3,-0.0038357

1,3,3,9,4,-0.0027657

1,3,3,9,5,0.00022376

1,3,3,9,6,0.0010373

1,3,3,9,7,0.0015118

1,3,3,9,8,-0.0012142

1,3,3,10,1,0.0042116

1,3,3,10,2,-0.0052745

1,3,3,10,3,-0.0071917

1,3,3,10,4,-0.0041199

1,3,3,10,5,-0.0010479

1,3,3,10,6,-0.0019899

1,3,3,10,7,0.00044997

1,3,3,10,8,-0.002992

1,3,3,11,1,0.0018485

1,3,3,11,2,-0.0038239

1,3,3,11,3,-0.0058093

1,3,3,11,4,-0.0020084

1,3,3,11,5,0.0013299

1,3,3,11,6,-0.0020677

1,3,3,11,7,0.00016007

1,3,3,11,8,-0.0025675

1,3,3,12,1,0.001942

1,3,3,12,2,-0.00044361

1,3,3,12,3,-0.0037759

1,3,3,12,4,-0.0016932

1,3,3,12,5,-0.00071462

1,3,3,12,6,2.1502e-05

1,3,3,12,7,2.0963e-05

1,3,3,12,8,-0.0019418

1,3,3,13,1,0.0025757

1,3,3,13,2,0.0032167

1,3,3,13,3,-0.001367

1,3,3,13,4,0.00092161

1,3,3,13,5,0.0026575

1,3,3,13,6,0.0035296

1,3,3,13,7,0.0022269

1,3,3,13,8,-0.00062481

1,3,3,14,1,0.003724

1,3,3,14,2,0.002533

1,3,3,14,3,-0.0028762

1,3,3,14,4,-0.0014983

1,3,3,14,5,0.0022896

1,3,3,14,6,0.0019498

1,3,3,14,7,0.0013609

1,3,3,14,8,-0.0013757

1,3,3,15,1,0.00084086

1,3,3,15,2,0.00024404

1,3,3,15,3,-0.0023941

1,3,3,15,4,-0.002972

1,3,3,15,5,0.0025551

1,3,3,15,6,0.00030325

1,3,3,15,7,0.0010964

1,3,3,15,8,-0.0015272

1,3,3,16,1,0.0015215

1,3,3,16,2,0.0013233

1,3,3,16,3,-0.004399

1,3,3,16,4,-0.00066507

1,3,3,16,5,0.0019903

1,3,3,16,6,0.0011147

1,3,3,16,7,0.0001853

1,3,3,16,8,1.4832e-05

1,3,3,17,1,0.0063154

1,3,3,17,2,0.003908

1,3,3,17,3,-0.0034538

1,3,3,17,4,0.00033562

1,3,3,17,5,0.0011642

1,3,3,17,6,0.0021575

1,3,3,17,7,0.0011755

1,3,3,17,8,-0.00091398

1,3,3,18,1,0.0027751

1,3,3,18,2,0.00090889

1,3,3,18,3,-0.0049512

1,3,3,18,4,-0.0039025

1,3,3,18,5,0.0017425

1,3,3,18,6,0.0012603

1,3,3,18,7,0.00093743

1,3,3,18,8,-0.001005

1,3,3,19,1,0.0024501

1,3,3,19,2,-0.00075328

1,3,3,19,3,-0.0011255

1,3,3,19,4,-0.0010893

1,3,3,19,5,0.0013151

1,3,3,19,6,0.00046751

1,3,3,19,7,-0.00065658

1,3,3,19,8,-0.0026454

1,3,3,20,1,0.0013366

1,3,3,20,2,-0.0011335

1,3,3,20,3,-0.0028299

1,3,3,20,4,-0.0031627

1,3,3,20,5,0.00087979

1,3,3,20,6,-0.00013525

1,3,3,20,7,2.8645e-05

1,3,3,20,8,-0.0029849

1,3,4,4,1,0.0035364

1,3,4,4,2,-0.0012741

1,3,4,4,3,-0.00018327

1,3,4,4,4,-0.00060766

1,3,4,4,5,0.0016292

1,3,4,4,6,0.00064047

1,3,4,4,7,-8.8904e-05

1,3,4,4,8,-0.00035162

1,3,4,5,1,0.0048806

1,3,4,5,2,0.0015671

1,3,4,5,3,-0.0048573

1,3,4,5,4,-0.0040873

1,3,4,5,5,0.0012136

1,3,4,5,6,0.00060126

1,3,4,5,7,0.00039485

1,3,4,5,8,-0.0022345

1,3,4,6,1,0.0043865

1,3,4,6,2,0.0016589

1,3,4,6,3,-0.0027601

1,3,4,6,4,-0.0011172

1,3,4,6,5,0.0012269

1,3,4,6,6,0.001734

1,3,4,6,7,0.000894

1,3,4,6,8,-0.00023906

1,3,4,7,1,0.0044039

1,3,4,7,2,6.6123e-05

1,3,4,7,3,-0.00088834

1,3,4,7,4,-0.0037167

1,3,4,7,5,0.0020217

1,3,4,7,6,0.00059223

1,3,4,7,7,0.00071864

1,3,4,7,8,-0.0018153

1,3,4,8,1,0.0018721

1,3,4,8,2,-0.0031616

1,3,4,8,3,-0.005594

1,3,4,8,4,-0.0016682

1,3,4,8,5,-0.00020034

1,3,4,8,6,0.00091062

1,3,4,8,7,-0.00041978

1,3,4,8,8,-0.00074107

1,3,4,9,1,0.0011941

1,3,4,9,2,-0.0043544

1,3,4,9,3,-0.0029748

1,3,4,9,4,-0.0012792

1,3,4,9,5,0.00067777

1,3,4,9,6,0.0020263

1,3,4,9,7,0.00064001

1,3,4,9,8,-0.00018858

1,3,4,10,1,0.002117

1,3,4,10,2,-0.007939

1,3,4,10,3,-0.0042383

1,3,4,10,4,-0.0051488

1,3,4,10,5,6.1524e-05

1,3,4,10,6,-0.00027981

1,3,4,10,7,0.00035454

1,3,4,10,8,-0.0014158

1,3,4,11,1,9.9207e-12

1,3,4,11,2,-0.0038024

1,3,4,11,3,-0.006536

1,3,4,11,4,-0.00459

1,3,4,11,5,-0.0010724

1,3,4,11,6,-1.8752e-05

1,3,4,11,7,5.2921e-05

1,3,4,11,8,-0.0013899

1,3,4,12,1,0.0028579

1,3,4,12,2,-0.00012478

1,3,4,12,3,-0.00079764

1,3,4,12,4,-0.0038361

1,3,4,12,5,0.0029177

1,3,4,12,6,-0.0020929

1,3,4,12,7,0.0026253

1,3,4,12,8,-0.0023713

1,3,4,13,1,0.0026519

1,3,4,13,2,-0.0057495

1,3,4,13,3,-0.0076124

1,3,4,13,4,-0.0072925

1,3,4,13,5,0.00024209

1,3,4,13,6,0.0022135

1,3,4,13,7,0.0013187

1,3,4,13,8,-0.0021092

1,3,4,14,1,0.003992

1,3,4,14,2,-0.0011746

1,3,4,14,3,-0.0046501

1,3,4,14,4,-0.0031875

1,3,4,14,5,0.0008235

1,3,4,14,6,0.001464

1,3,4,14,7,0.001023

1,3,4,14,8,-0.0025559

1,3,4,15,1,0.0010821

1,3,4,15,2,-0.0010844

1,3,4,15,3,-0.0013361

1,3,4,15,4,-0.0031363

1,3,4,15,5,0.0010844

1,3,4,15,6,0.00069529

1,3,4,15,7,0.0019424

1,3,4,15,8,-0.00084188

1,3,4,16,1,3.5941e-09

1,3,4,16,2,0.00071182

1,3,4,16,3,-0.0010767

1,3,4,16,4,0.0011706

1,3,4,16,5,0.0017431

1,3,4,16,6,0.00073506

1,3,4,16,7,-6.3297e-05

1,3,4,16,8,-0.0019602

1,3,4,17,1,0.0079888

1,3,4,17,2,0.0031729

1,3,4,17,3,-0.0019679

1,3,4,17,4,-0.0022112

1,3,4,17,5,0.0017779

1,3,4,17,6,0.0017296

1,3,4,17,7,0.0012725

1,3,4,17,8,-0.0018293

1,3,4,18,1,0.002627

1,3,4,18,2,-0.00095942

1,3,4,18,3,-0.0028057

1,3,4,18,4,-0.0047866

1,3,4,18,5,0.0016506

1,3,4,18,6,9.8972e-05

1,3,4,18,7,0.00039805

1,3,4,18,8,-0.0019852

1,3,4,19,1,0.0022026

1,3,4,19,2,0.00088008

1,3,4,19,3,-0.0039076

1,3,4,19,4,-0.0016684

1,3,4,19,5,0.0026935

1,3,4,19,6,0.00083687

1,3,4,19,7,0.0011213

1,3,4,19,8,-0.0032208

1,3,4,20,1,0.0010996

1,3,4,20,2,-0.0024182

1,3,4,20,3,-0.0046007

1,3,4,20,4,-0.005565

1,3,4,20,5,-0.00046522

1,3,4,20,6,0.00031346

1,3,4,20,7,-0.00037869

1,3,4,20,8,-0.0018558

1,3,5,5,1,0.0029348

1,3,5,5,2,-0.0013998

1,3,5,5,3,-0.003025

1,3,5,5,4,-0.0029965

1,3,5,5,5,0.00030748

1,3,5,5,6,-1.7184e-05

1,3,5,5,7,-0.00026198

1,3,5,5,8,-0.001215

1,3,5,6,1,0.0035475

1,3,5,6,2,-0.00037497

1,3,5,6,3,-0.003911

1,3,5,6,4,-0.0010425

1,3,5,6,5,0.0011422

1,3,5,6,6,0.001325

1,3,5,6,7,0.0011282

1,3,5,6,8,-0.00084818

1,3,5,7,1,0.0042184

1,3,5,7,2,-0.00081078

1,3,5,7,3,-0.0040974

1,3,5,7,4,-0.0028442

1,3,5,7,5,0.00021396

1,3,5,7,6,0.0016481

1,3,5,7,7,0.0012946

1,3,5,7,8,-0.0016116

1,3,5,8,1,0.0026811

1,3,5,8,2,-0.0011033

1,3,5,8,3,-0.0042169

1,3,5,8,4,-0.0020553

1,3,5,8,5,0.00092434

1,3,5,8,6,0.0016547

1,3,5,8,7,0.00012176

1,3,5,8,8,-0.0012078

1,3,5,9,1,0.0033032

1,3,5,9,2,-0.00024326

1,3,5,9,3,-0.0051679

1,3,5,9,4,-0.0032597

1,3,5,9,5,0.0013409

1,3,5,9,6,0.0015019

1,3,5,9,7,0.0010868

1,3,5,9,8,-0.0016582

1,3,5,10,1,0.0062478

1,3,5,10,2,0.00091884

1,3,5,10,3,-0.0038331

1,3,5,10,4,-0.0031953

1,3,5,10,5,0.0011417

1,3,5,10,6,-0.00068446

1,3,5,10,7,0.00038285

1,3,5,10,8,-0.0028617

1,3,5,11,1,0.0031413

1,3,5,11,2,0.0013247

1,3,5,11,3,-0.0035172

1,3,5,11,4,-0.0023034

1,3,5,11,5,0.0013955

1,3,5,11,6,0.0020408

1,3,5,11,7,0.00079125

1,3,5,11,8,-0.0011193

1,3,5,12,1,0.0024312

1,3,5,12,2,-0.0012721

1,3,5,12,3,-0.0043469

1,3,5,12,4,-0.00048908

1,3,5,12,5,0.0010207

1,3,5,12,6,0.00052199

1,3,5,12,7,-0.00081992

1,3,5,12,8,-0.00044129

1,3,5,13,1,0.0050342

1,3,5,13,2,-0.0019851

1,3,5,13,3,-0.005963

1,3,5,13,4,-0.0045123

1,3,5,13,5,-0.00077524

1,3,5,13,6,0.0017365

1,3,5,13,7,0.0013153

1,3,5,13,8,-0.0018915

1,3,5,14,1,0.0030193

1,3,5,14,2,-0.0080663

1,3,5,14,3,-0.0070214

1,3,5,14,4,-0.0061318

1,3,5,14,5,-0.0035212

1,3,5,14,6,-0.0014448

1,3,5,14,7,-0.00035476

1,3,5,14,8,-0.0028622

1,3,5,15,1,0.0017093

1,3,5,15,2,-0.0016246

1,3,5,15,3,-0.0042816

1,3,5,15,4,-0.0053948

1,3,5,15,5,-0.00076585

1,3,5,15,6,-0.0018239

1,3,5,15,7,-0.002587

1,3,5,15,8,-0.0025935

1,3,5,16,1,0.0017973

1,3,5,16,2,-0.00032274

1,3,5,16,3,-0.00041051

1,3,5,16,4,0.00095755

1,3,5,16,5,0.0045605

1,3,5,16,6,-0.00010548

1,3,5,16,7,0.0025525

1,3,5,16,8,-0.0015922

1,3,5,17,1,0.008679

1,3,5,17,2,0.0023445

1,3,5,17,3,-0.0057109

1,3,5,17,4,-0.0064393

1,3,5,17,5,-0.0010469

1,3,5,17,6,-0.0015154

1,3,5,17,7,-0.00054492

1,3,5,17,8,-0.0028165

1,3,5,18,1,0.0018381

1,3,5,18,2,-0.00060489

1,3,5,18,3,-0.0071864

1,3,5,18,4,-0.0069905

1,3,5,18,5,-0.0014053

1,3,5,18,6,-0.002571

1,3,5,18,7,-0.0015274

1,3,5,18,8,-0.0039835

1,3,5,19,1,0.0015785

1,3,5,19,2,-4.0382e-05

1,3,5,19,3,-0.0056057

1,3,5,19,4,-0.0064924

1,3,5,19,5,0.001422

1,3,5,19,6,-0.00058101

1,3,5,19,7,-0.00087766

1,3,5,19,8,-0.003565

1,3,5,20,1,0.0011657

1,3,5,20,2,-0.00045437

1,3,5,20,3,-0.0064535

1,3,5,20,4,-0.0043385

1,3,5,20,5,-0.0037215

1,3,5,20,6,-0.00058141

1,3,5,20,7,-0.0040217

1,3,5,20,8,-0.0030534

1,3,6,6,1,0.0032591

1,3,6,6,2,-0.00053703

1,3,6,6,3,-0.0023659

1,3,6,6,4,-0.0015109

1,3,6,6,5,-0.00038662

1,3,6,6,6,-0.00061439

1,3,6,6,7,0.00054134

1,3,6,6,8,-0.0007014

1,3,6,7,1,0.0032034

1,3,6,7,2,-0.0014628

1,3,6,7,3,-0.0048851

1,3,6,7,4,-0.0020579

1,3,6,7,5,0.0018837

1,3,6,7,6,-0.00056905

1,3,6,7,7,-0.00042966

1,3,6,7,8,-0.001494

1,3,6,8,1,0.0031401

1,3,6,8,2,-0.002258

1,3,6,8,3,-0.005748

1,3,6,8,4,-0.0027913

1,3,6,8,5,-0.00068256

1,3,6,8,6,-0.0014027

1,3,6,8,7,-0.00010238

1,3,6,8,8,-0.0021483

1,3,6,9,1,0.0035925

1,3,6,9,2,0.0013876

1,3,6,9,3,-0.0042648

1,3,6,9,4,-0.0011184

1,3,6,9,5,-0.00091644

1,3,6,9,6,0.0028641

1,3,6,9,7,-0.00094741

1,3,6,9,8,0.00027506

1,3,6,10,1,0.0033919

1,3,6,10,2,-0.0029137

1,3,6,10,3,-0.0050817

1,3,6,10,4,-0.0023616

1,3,6,10,5,0.0031791

1,3,6,10,6,-0.00015809

1,3,6,10,7,0.0011341

1,3,6,10,8,-0.0013587

1,3,6,11,1,0.0029027

1,3,6,11,2,-0.0044628

1,3,6,11,3,-0.0042112

1,3,6,11,4,-0.0031466

1,3,6,11,5,0.0013656

1,3,6,11,6,0.00066987

1,3,6,11,7,0.0010994

1,3,6,11,8,-0.0012398

1,3,6,12,1,0.0016053

1,3,6,12,2,0.00061037

1,3,6,12,3,-0.0015575

1,3,6,12,4,-0.003041

1,3,6,12,5,0.0004894

1,3,6,12,6,0.00039128

1,3,6,12,7,0.0016064

1,3,6,12,8,-0.0011852

1,3,6,13,1,0.0048976

1,3,6,13,2,0.0022158

1,3,6,13,3,0.00039022

1,3,6,13,4,-0.0013758

1,3,6,13,5,0.0021412

1,3,6,13,6,0.0027

1,3,6,13,7,0.0030679

1,3,6,13,8,-0.00076999

1,3,6,14,1,0.0039117

1,3,6,14,2,0.0019366

1,3,6,14,3,-0.0019059

1,3,6,14,4,-0.0013375

1,3,6,14,5,0.0004248

1,3,6,14,6,0.0014623

1,3,6,14,7,0.0010694

1,3,6,14,8,-0.0020972

1,3,6,15,1,0.0016841

1,3,6,15,2,-6.5181e-05

1,3,6,15,3,-0.0023134

1,3,6,15,4,-0.0015334

1,3,6,15,5,0.0032845

1,3,6,15,6,0.00065014

1,3,6,15,7,-0.0010757

1,3,6,15,8,-0.0033951

1,3,6,16,1,4.9895e-11

1,3,6,16,2,0.0013888

1,3,6,16,3,-0.0013341

1,3,6,16,4,-0.00031814

1,3,6,16,5,0.0016575

1,3,6,16,6,0.00065369

1,3,6,16,7,0.0022086

1,3,6,16,8,-0.0014129

1,3,6,17,1,0.0036334

1,3,6,17,2,0.00049488

1,3,6,17,3,-0.0022785

1,3,6,17,4,-0.0014689

1,3,6,17,5,0.00213

1,3,6,17,6,0.0020829

1,3,6,17,7,0.00071403

1,3,6,17,8,-0.00021512

1,3,6,18,1,0.0028324

1,3,6,18,2,0.0034344

1,3,6,18,3,-0.0024488

1,3,6,18,4,-0.0030385

1,3,6,18,5,0.0006002

1,3,6,18,6,0.0010288

1,3,6,18,7,0.001375

1,3,6,18,8,-0.00012023

1,3,6,19,1,0.001857

1,3,6,19,2,-0.0023314

1,3,6,19,3,-0.0033366

1,3,6,19,4,-0.002037

1,3,6,19,5,8.6186e-05

1,3,6,19,6,0.0009864

1,3,6,19,7,0.00016303

1,3,6,19,8,-0.0010661

1,3,6,20,1,0.0010052

1,3,6,20,2,0.00026402

1,3,6,20,3,-0.0025187

1,3,6,20,4,-0.0063908

1,3,6,20,5,0.00027864

1,3,6,20,6,0.00053875

1,3,6,20,7,-2.5599e-05

1,3,6,20,8,-0.0014753

1,3,7,7,1,0.0020573

1,3,7,7,2,-0.0013138

1,3,7,7,3,-0.00075508

1,3,7,7,4,-0.0015132

1,3,7,7,5,0.0007673

1,3,7,7,6,0.0021042

1,3,7,7,7,-0.00014555

1,3,7,7,8,-3.418e-05

1,3,7,8,1,0.0039741

1,3,7,8,2,-0.00048368

1,3,7,8,3,-0.0046906

1,3,7,8,4,-0.0025725

1,3,7,8,5,0.00054905

1,3,7,8,6,0.0007284

1,3,7,8,7,0.00028891

1,3,7,8,8,-0.0011804

1,3,7,9,1,0.00027154

1,3,7,9,2,-0.005371

1,3,7,9,3,-0.0057217

1,3,7,9,4,-0.0028491

1,3,7,9,5,0.0012797

1,3,7,9,6,0.0011285

1,3,7,9,7,0.00068875

1,3,7,9,8,-0.0015592

1,3,7,10,1,0.0030296

1,3,7,10,2,-0.0023694

1,3,7,10,3,-0.0046667

1,3,7,10,4,-0.00040157

1,3,7,10,5,0.0010386

1,3,7,10,6,0.0026016

1,3,7,10,7,0.0011404

1,3,7,10,8,-2.7855e-05

1,3,7,11,1,0.0014726

1,3,7,11,2,-0.0026365

1,3,7,11,3,-0.0038976

1,3,7,11,4,-0.0017858

1,3,7,11,5,0.0010149

1,3,7,11,6,0.0013901

1,3,7,11,7,0.0015374

1,3,7,11,8,-0.00077027

1,3,7,12,1,0.00069137

1,3,7,12,2,-0.0011338

1,3,7,12,3,0.00042172

1,3,7,12,4,-0.0036328

1,3,7,12,5,0.0026264

1,3,7,12,6,0.0026818

1,3,7,12,7,-0.00023734

1,3,7,12,8,-0.00046199

1,3,7,13,1,0.0036763

1,3,7,13,2,-0.0028739

1,3,7,13,3,-0.0049918

1,3,7,13,4,-0.0032476

1,3,7,13,5,0.0011762

1,3,7,13,6,0.0025196

1,3,7,13,7,0.0021326

1,3,7,13,8,-0.001819

1,3,7,14,1,0.0023707

1,3,7,14,2,-0.0040087

1,3,7,14,3,-0.0048486

1,3,7,14,4,-0.0060794

1,3,7,14,5,-4.2426e-05

1,3,7,14,6,0.0016591

1,3,7,14,7,0.00036405

1,3,7,14,8,-0.0020619

1,3,7,15,1,0.0012276

1,3,7,15,2,0.00084792

1,3,7,15,3,-0.0029278

1,3,7,15,4,-0.0030032

1,3,7,15,5,0.00087496

1,3,7,15,6,0.00076041

1,3,7,15,7,-0.00054329

1,3,7,15,8,-0.001305

1,3,7,16,1,0.00039087

1,3,7,16,2,-0.0027199

1,3,7,16,3,-0.0029232

1,3,7,16,4,-0.00010601

1,3,7,16,5,0.001341

1,3,7,16,6,0.0020332

1,3,7,16,7,4.613e-05

1,3,7,16,8,-0.0011678

1,3,7,17,1,0.0046298

1,3,7,17,2,-0.0016342

1,3,7,17,3,-0.0021327

1,3,7,17,4,-0.0034054

1,3,7,17,5,0.0018568

1,3,7,17,6,0.0014624

1,3,7,17,7,0.00081082

1,3,7,17,8,-0.0021429

1,3,7,18,1,4.95e-11

1,3,7,18,2,-0.0017325

1,3,7,18,3,-0.0022696

1,3,7,18,4,-0.002722

1,3,7,18,5,-0.00013149

1,3,7,18,6,0.0025695

1,3,7,18,7,0.00017608

1,3,7,18,8,-0.00070877

1,3,7,19,1,0.0019161

1,3,7,19,2,-0.00077663

1,3,7,19,3,-0.0059856

1,3,7,19,4,-0.0049303

1,3,7,19,5,0.00045973

1,3,7,19,6,0.00047337

1,3,7,19,7,-0.0011148

1,3,7,19,8,-0.00087758

1,3,7,20,1,0.00063999

1,3,7,20,2,-0.0041614

1,3,7,20,3,-0.0030967

1,3,7,20,4,-0.0060233

1,3,7,20,5,0.00068015

1,3,7,20,6,0.00054627

1,3,7,20,7,-0.0020377

1,3,7,20,8,-0.0027225

1,3,8,8,1,0.0051364

1,3,8,8,2,0.0029383

1,3,8,8,3,-0.0009182

1,3,8,8,4,-0.00011975

1,3,8,8,5,0.0011076

1,3,8,8,6,0.00056877

1,3,8,8,7,0.00094111

1,3,8,8,8,-0.00060848

1,3,8,9,1,0.0045625

1,3,8,9,2,-0.0077319

1,3,8,9,3,-0.0036011

1,3,8,9,4,-0.0004317

1,3,8,9,5,0.00080529

1,3,8,9,6,0.0013116

1,3,8,9,7,0.001546

1,3,8,9,8,-0.00089851

1,3,8,10,1,0.0044888

1,3,8,10,2,-0.0037272

1,3,8,10,3,-0.0038786

1,3,8,10,4,-0.001278

1,3,8,10,5,0.00055311

1,3,8,10,6,0.00017524

1,3,8,10,7,0.0005126

1,3,8,10,8,-0.0014057

1,3,8,11,1,0.0041263

1,3,8,11,2,-0.0043145

1,3,8,11,3,-0.0050463

1,3,8,11,4,-0.0041616

1,3,8,11,5,0.00047181

1,3,8,11,6,0.00065914

1,3,8,11,7,0.0011235

1,3,8,11,8,-0.00039213

1,3,8,12,1,0.0036739

1,3,8,12,2,0.00082444

1,3,8,12,3,-0.0041798

1,3,8,12,4,-0.0025141

1,3,8,12,5,0.001029

1,3,8,12,6,0.001996

1,3,8,12,7,0.00049135

1,3,8,12,8,0.0005848

1,3,8,13,1,0.0037457

1,3,8,13,2,-0.0044538

1,3,8,13,3,-0.0039886

1,3,8,13,4,-0.0028956

1,3,8,13,5,0.0013265

1,3,8,13,6,0.0018661

1,3,8,13,7,0.0014244

1,3,8,13,8,-0.0020491

1,3,8,14,1,0.0032217

1,3,8,14,2,-0.0013189

1,3,8,14,3,-0.0044394

1,3,8,14,4,-0.0026328

1,3,8,14,5,0.001853

1,3,8,14,6,0.0021129

1,3,8,14,7,0.0012448

1,3,8,14,8,-0.0012595

1,3,8,15,1,0.0015936

1,3,8,15,2,-0.0022454

1,3,8,15,3,-0.0045808

1,3,8,15,4,-0.0022867

1,3,8,15,5,-0.00042937

1,3,8,15,6,-0.00044869

1,3,8,15,7,2.1109e-05

1,3,8,15,8,-0.0015819

1,3,8,16,1,0.0032515

1,3,8,16,2,0.0012158

1,3,8,16,3,-0.0016039

1,3,8,16,4,-0.00028119

1,3,8,16,5,0.0018691

1,3,8,16,6,-0.00085759

1,3,8,16,7,0.0013619

1,3,8,16,8,-0.0028547

1,3,8,17,1,0.0033759

1,3,8,17,2,0.00095516

1,3,8,17,3,-0.0020624

1,3,8,17,4,-0.0010099

1,3,8,17,5,0.0025243

1,3,8,17,6,0.0014908

1,3,8,17,7,0.0014508

1,3,8,17,8,-0.00053077

1,3,8,18,1,0.0043569

1,3,8,18,2,0.00027213

1,3,8,18,3,-0.0025662

1,3,8,18,4,-0.003178

1,3,8,18,5,0.0024422

1,3,8,18,6,0.0013497

1,3,8,18,7,0.0013839

1,3,8,18,8,-0.0022141

1,3,8,19,1,0.0014444

1,3,8,19,2,-0.0040237

1,3,8,19,3,-0.0041281

1,3,8,19,4,-0.0028261

1,3,8,19,5,0.00089764

1,3,8,19,6,0.0020426

1,3,8,19,7,7.0949e-05

1,3,8,19,8,-0.0019079

1,3,8,20,1,0.001356

1,3,8,20,2,0.0021929

1,3,8,20,3,-0.0045828

1,3,8,20,4,-0.0047027

1,3,8,20,5,0.00022277

1,3,8,20,6,0.00051834

1,3,8,20,7,-1.2808e-05

1,3,8,20,8,-0.0043153

1,3,9,9,1,0.0015215

1,3,9,9,2,-0.0062103

1,3,9,9,3,-0.0014692

1,3,9,9,4,-0.0027473

1,3,9,9,5,-0.0010004

1,3,9,9,6,0.00019686

1,3,9,9,7,0.00071487

1,3,9,9,8,-0.0012375

1,3,9,10,1,0.00077828

1,3,9,10,2,-0.0043162

1,3,9,10,3,-0.0023499

1,3,9,10,4,-0.0016214

1,3,9,10,5,0.00049292

1,3,9,10,6,0.0023348

1,3,9,10,7,0.00034389

1,3,9,10,8,-0.00054833

1,3,9,11,1,0.0026088

1,3,9,11,2,-0.0042051

1,3,9,11,3,-0.0051073

1,3,9,11,4,-0.0036299

1,3,9,11,5,-0.00067508

1,3,9,11,6,0.0011801

1,3,9,11,7,-0.00039725

1,3,9,11,8,-0.00067839

1,3,9,12,1,0.0015751

1,3,9,12,2,-0.0012493

1,3,9,12,3,-0.004894

1,3,9,12,4,-0.00010692

1,3,9,12,5,0.0021656

1,3,9,12,6,0.0001352

1,3,9,12,7,0.0017894

1,3,9,12,8,-0.0022779

1,3,9,13,1,0.0051197

1,3,9,13,2,-0.0005059

1,3,9,13,3,-0.0026072

1,3,9,13,4,-0.0033326

1,3,9,13,5,0.0011151

1,3,9,13,6,0.0023466

1,3,9,13,7,0.00097401

1,3,9,13,8,-0.00071011

1,3,9,14,1,0.003022

1,3,9,14,2,-0.0054287

1,3,9,14,3,-0.0077853

1,3,9,14,4,-0.0067977

1,3,9,14,5,-0.00086093

1,3,9,14,6,0.00051361

1,3,9,14,7,-0.00047289

1,3,9,14,8,-0.0027819

1,3,9,15,1,0.0016479

1,3,9,15,2,0.0041268

1,3,9,15,3,-0.0031139

1,3,9,15,4,-0.0042388

1,3,9,15,5,0.0020868

1,3,9,15,6,0.0018997

1,3,9,15,7,0.00059928

1,3,9,15,8,-0.0024982

1,3,9,16,1,0.0031204

1,3,9,16,2,-0.0043473

1,3,9,16,3,-0.0042984

1,3,9,16,4,0.0012658

1,3,9,16,5,-0.00063049

1,3,9,16,6,0.0019051

1,3,9,16,7,4.4529e-05

1,3,9,16,8,0.0018643

1,3,9,17,1,0.008838

1,3,9,17,2,0.0045723

1,3,9,17,3,-0.0046295

1,3,9,17,4,-0.004742

1,3,9,17,5,0.00012423

1,3,9,17,6,0.00038916

1,3,9,17,7,0.00075393

1,3,9,17,8,-0.0012183

1,3,9,18,1,0.0018257

1,3,9,18,2,-0.0022355

1,3,9,18,3,-0.0052415

1,3,9,18,4,-0.0058399

1,3,9,18,5,0.0013554

1,3,9,18,6,0.0013245

1,3,9,18,7,9.931e-05

1,3,9,18,8,-0.0028586

1,3,9,19,1,0.0014886

1,3,9,19,2,7.5697e-05

1,3,9,19,3,-0.0054684

1,3,9,19,4,-0.0047751

1,3,9,19,5,0.0024389

1,3,9,19,6,0.001017

1,3,9,19,7,0.00086182

1,3,9,19,8,-0.0028303

1,3,9,20,1,0.0010723

1,3,9,20,2,-0.00095107

1,3,9,20,3,-0.0023073

1,3,9,20,4,-0.004628

1,3,9,20,5,-0.00011244

1,3,9,20,6,-0.0003716

1,3,9,20,7,-0.0025478

1,3,9,20,8,-0.0024237

1,3,10,10,1,0.0064115

1,3,10,10,2,-0.0040998

1,3,10,10,3,-0.00050227

1,3,10,10,4,8.5755e-05

1,3,10,10,5,0.0016421

1,3,10,10,6,0.0013282

1,3,10,10,7,0.00044342

1,3,10,10,8,0.0005366

1,3,10,11,1,0.005285

1,3,10,11,2,0.0017299

1,3,10,11,3,-8.7293e-05

1,3,10,11,4,-0.0025817

1,3,10,11,5,0.0017621

1,3,10,11,6,0.001805

1,3,10,11,7,0.0020622

1,3,10,11,8,0.00053022

1,3,10,12,1,0.0021836

1,3,10,12,2,-0.0030725

1,3,10,12,3,-0.0056331

1,3,10,12,4,-0.0016934

1,3,10,12,5,-0.00090858

1,3,10,12,6,0.0010235

1,3,10,12,7,7.1991e-05

1,3,10,12,8,-0.00018791

1,3,10,13,1,0.0035366

1,3,10,13,2,-0.0018848

1,3,10,13,3,-0.0046749

1,3,10,13,4,-0.002416

1,3,10,13,5,8.0405e-05

1,3,10,13,6,0.0023056

1,3,10,13,7,0.0013057

1,3,10,13,8,-0.0018011

1,3,10,14,1,0.0050681

1,3,10,14,2,-0.001492

1,3,10,14,3,-0.0058797

1,3,10,14,4,-0.0027418

1,3,10,14,5,0.00047766

1,3,10,14,6,0.0025217

1,3,10,14,7,0.0016302

1,3,10,14,8,-0.0014167

1,3,10,15,1,0.0019801

1,3,10,15,2,-0.0010456

1,3,10,15,3,-0.0054943

1,3,10,15,4,-0.0052236

1,3,10,15,5,1.4655e-06

1,3,10,15,6,0.00040442

1,3,10,15,7,-0.0010237

1,3,10,15,8,-0.0017808

1,3,10,16,1,0.0018772

1,3,10,16,2,-0.00061791

1,3,10,16,3,-0.0038063

1,3,10,16,4,-0.0020215

1,3,10,16,5,0.001297

1,3,10,16,6,0.00034601

1,3,10,16,7,0.00056752

1,3,10,16,8,-0.0012766

1,3,10,17,1,0.0089578

1,3,10,17,2,0.0028666

1,3,10,17,3,-0.0038659

1,3,10,17,4,-0.0014449

1,3,10,17,5,0.0017578

1,3,10,17,6,0.0019164

1,3,10,17,7,0.0015998

1,3,10,17,8,-0.00066626

1,3,10,18,1,0.0022632

1,3,10,18,2,-0.0011827

1,3,10,18,3,-0.0024864

1,3,10,18,4,-0.006547

1,3,10,18,5,0.00077341

1,3,10,18,6,-0.00021164

1,3,10,18,7,-0.00020865

1,3,10,18,8,-0.0018935

1,3,10,19,1,0.0030384

1,3,10,19,2,-0.0040183

1,3,10,19,3,-0.0043639

1,3,10,19,4,-0.0029276

1,3,10,19,5,0.00061971

1,3,10,19,6,0.00079913

1,3,10,19,7,0.00075827

1,3,10,19,8,-0.0013086

1,3,10,20,1,0.0010335

1,3,10,20,2,-0.0080337

1,3,10,20,3,-0.0044956

1,3,10,20,4,-0.0047687

1,3,10,20,5,-2.7049e-05

1,3,10,20,6,-0.00033007

1,3,10,20,7,-0.0012913

1,3,10,20,8,-0.00041557

1,3,11,11,1,0.0055486

1,3,11,11,2,0.00019191

1,3,11,11,3,-0.00012454

1,3,11,11,4,-0.001867

1,3,11,11,5,0.0016381

1,3,11,11,6,0.0013568

1,3,11,11,7,0.00021204

1,3,11,11,8,-2.9377e-05

1,3,11,12,1,0.0015352

1,3,11,12,2,-0.0041368

1,3,11,12,3,-0.0052455

1,3,11,12,4,-0.0059621

1,3,11,12,5,-0.00035447

1,3,11,12,6,-0.00036283

1,3,11,12,7,0.00035756

1,3,11,12,8,-0.0016212

1,3,11,13,1,0.0032852

1,3,11,13,2,-0.0033878

1,3,11,13,3,-0.0030375

1,3,11,13,4,-0.0047916

1,3,11,13,5,0.00069986

1,3,11,13,6,0.0016991

1,3,11,13,7,0.0026335

1,3,11,13,8,-0.0026536

1,3,11,14,1,0.002454

1,3,11,14,2,-0.0004582

1,3,11,14,3,-0.0034581

1,3,11,14,4,-0.0029559

1,3,11,14,5,0.0022223

1,3,11,14,6,0.0028132

1,3,11,14,7,0.0019131

1,3,11,14,8,-0.0023445

1,3,11,15,1,0.0011903

1,3,11,15,2,-0.0005759

1,3,11,15,3,-0.0030824

1,3,11,15,4,-0.0039791

1,3,11,15,5,0.0010973

1,3,11,15,6,0.00095497

1,3,11,15,7,0.0010222

1,3,11,15,8,-0.001167

1,3,11,16,1,0.0036597

1,3,11,16,2,-0.00038621

1,3,11,16,3,-0.0026263

1,3,11,16,4,-0.0022383

1,3,11,16,5,0.0006534

1,3,11,16,6,-4.0285e-05

1,3,11,16,7,0.00054518

1,3,11,16,8,-0.0009694

1,3,11,17,1,0.0066514

1,3,11,17,2,0.0015015

1,3,11,17,3,-0.003287

1,3,11,17,4,-0.0031242

1,3,11,17,5,0.0024996

1,3,11,17,6,0.0011399

1,3,11,17,7,0.0011344

1,3,11,17,8,-0.0012767

1,3,11,18,1,0.0010282

1,3,11,18,2,-0.00029442

1,3,11,18,3,-0.0051307

1,3,11,18,4,-0.0045846

1,3,11,18,5,-0.0013765

1,3,11,18,6,0.0015112

1,3,11,18,7,-0.00096584

1,3,11,18,8,-0.0027333

1,3,11,19,1,0.0020969

1,3,11,19,2,-0.0020846

1,3,11,19,3,-0.0066072

1,3,11,19,4,-0.0030869

1,3,11,19,5,0.00129

1,3,11,19,6,0.0013393

1,3,11,19,7,-0.00069284

1,3,11,19,8,-0.0019722

1,3,11,20,1,0.00056768

1,3,11,20,2,-0.0041472

1,3,11,20,3,-0.0047157

1,3,11,20,4,-0.0068307

1,3,11,20,5,-0.00053988

1,3,11,20,6,0.0026114

1,3,11,20,7,-0.00062114

1,3,11,20,8,-0.0023943

1,3,12,12,1,0.0014017

1,3,12,12,2,-0.00078512

1,3,12,12,3,-0.00044005

1,3,12,12,4,-0.0016972

1,3,12,12,5,-0.0011228

1,3,12,12,6,0.00010911

1,3,12,12,7,-0.0010632

1,3,12,12,8,-0.0013759

1,3,12,13,1,5.5638e-11

1,3,12,13,2,-0.0032115

1,3,12,13,3,-0.0018314

1,3,12,13,4,-0.0034707

1,3,12,13,5,-0.00022156

1,3,12,13,6,0.0019162

1,3,12,13,7,0.0010952

1,3,12,13,8,-0.0010446

1,3,12,14,1,0.00079788

1,3,12,14,2,-0.00072337

1,3,12,14,3,-0.0072048

1,3,12,14,4,-0.0042327

1,3,12,14,5,0.0022253

1,3,12,14,6,0.002365

1,3,12,14,7,0.00075599

1,3,12,14,8,-0.0019267

1,3,12,15,1,0.00083814

1,3,12,15,2,-0.0017585

1,3,12,15,3,-0.0037518

1,3,12,15,4,-0.0043465

1,3,12,15,5,-0.0013834

1,3,12,15,6,-0.00081912

1,3,12,15,7,0.0012021

1,3,12,15,8,-0.0024561

1,3,12,16,1,0.0013999

1,3,12,16,2,0.00029778

1,3,12,16,3,-0.004361

1,3,12,16,4,-0.0047951

1,3,12,16,5,0.0021444

1,3,12,16,6,-0.0029081

1,3,12,16,7,-0.0039291

1,3,12,16,8,-0.0012334

1,3,12,17,1,0.0034672

1,3,12,17,2,0.00067366

1,3,12,17,3,-0.0028964

1,3,12,17,4,-0.0041968

1,3,12,17,5,0.001174

1,3,12,17,6,0.00076483

1,3,12,17,7,0.0015326

1,3,12,17,8,-0.001523

1,3,12,18,1,0.0014659

1,3,12,18,2,-0.00054129

1,3,12,18,3,-0.0049887

1,3,12,18,4,-0.0044573

1,3,12,18,5,0.00065816

1,3,12,18,6,0.0006037

1,3,12,18,7,-0.0014008

1,3,12,18,8,-0.0024761

1,3,12,19,1,0.001256

1,3,12,19,2,-0.0017694

1,3,12,19,3,-0.0024018

1,3,12,19,4,-0.0024741

1,3,12,19,5,0.0033585

1,3,12,19,6,-0.0012773

1,3,12,19,7,0.00043876

1,3,12,19,8,-0.0030156

1,3,12,20,1,0.00044415

1,3,12,20,2,0.00073306

1,3,12,20,3,-0.00041642

1,3,12,20,4,-0.0020575

1,3,12,20,5,0.00056066

1,3,12,20,6,-0.0014429

1,3,12,20,7,0.00011532

1,3,12,20,8,-0.0038643

1,3,13,13,1,0.0051363

1,3,13,13,2,-0.0056487

1,3,13,13,3,-0.0046475

1,3,13,13,4,-0.0040887

1,3,13,13,5,-0.0011416

1,3,13,13,6,-0.0014567

1,3,13,13,7,0.00028176

1,3,13,13,8,-0.0019648

1,3,13,14,1,0.0025113

1,3,13,14,2,-0.01585

1,3,13,14,3,-0.012483

1,3,13,14,4,-0.0095302

1,3,13,14,5,-0.0046419

1,3,13,14,6,-0.0029879

1,3,13,14,7,-0.0014997

1,3,13,14,8,-0.0047433

1,3,13,15,1,0.00054735

1,3,13,15,2,-0.0032754

1,3,13,15,3,-0.0087829

1,3,13,15,4,-0.0073941

1,3,13,15,5,-0.00253

1,3,13,15,6,-0.0012687

1,3,13,15,7,-0.0020225

1,3,13,15,8,-0.0053823

1,3,13,16,1,0.00029695

1,3,13,16,2,-0.0050434

1,3,13,16,3,-0.0056624

1,3,13,16,4,-0.0045114

1,3,13,16,5,0.0013299

1,3,13,16,6,-0.0013241

1,3,13,16,7,0.00011194

1,3,13,16,8,-0.0016279

1,3,13,17,1,0.0093759

1,3,13,17,2,-0.0057556

1,3,13,17,3,-0.0079643

1,3,13,17,4,-0.008911

1,3,13,17,5,-0.0036937

1,3,13,17,6,-0.0024405

1,3,13,17,7,-0.0015351

1,3,13,17,8,-0.003937

1,3,13,18,1,0.0016313

1,3,13,18,2,-0.0079499

1,3,13,18,3,-0.011194

1,3,13,18,4,-0.0088657

1,3,13,18,5,-0.0037485

1,3,13,18,6,-0.0037805

1,3,13,18,7,-0.00093971

1,3,13,18,8,-0.0053261

1,3,13,19,1,0.00076328

1,3,13,19,2,-0.0034871

1,3,13,19,3,-0.010832

1,3,13,19,4,-0.0065636

1,3,13,19,5,-0.0015142

1,3,13,19,6,-0.0017074

1,3,13,19,7,-0.0007048

1,3,13,19,8,-0.003807

1,3,13,20,1,0.00092576

1,3,13,20,2,-0.0046617

1,3,13,20,3,-0.0065026

1,3,13,20,4,-0.0072152

1,3,13,20,5,-0.0018671

1,3,13,20,6,0.0013392

1,3,13,20,7,-5.8206e-05

1,3,13,20,8,-0.0023587

1,3,14,14,1,0.0037634

1,3,14,14,2,-0.0079537

1,3,14,14,3,-0.0084314

1,3,14,14,4,-0.0049118

1,3,14,14,5,-0.0035918

1,3,14,14,6,-0.0027659

1,3,14,14,7,-0.0013318

1,3,14,14,8,-0.0032971

1,3,14,15,1,0.00062779

1,3,14,15,2,-0.0062865

1,3,14,15,3,-0.00973

1,3,14,15,4,-0.010466

1,3,14,15,5,-0.0045302

1,3,14,15,6,-0.0038042

1,3,14,15,7,-0.00022985

1,3,14,15,8,-0.0043903

1,3,14,16,1,0.0017135

1,3,14,16,2,-0.0037082

1,3,14,16,3,-0.0043416

1,3,14,16,4,-0.0010766

1,3,14,16,5,-0.0010866

1,3,14,16,6,-0.00065911

1,3,14,16,7,0.00037257

1,3,14,16,8,-0.003222

1,3,14,17,1,0.0044978

1,3,14,17,2,-0.0080843

1,3,14,17,3,-0.012892

1,3,14,17,4,-0.010994

1,3,14,17,5,-0.0049887

1,3,14,17,6,-0.0053253

1,3,14,17,7,-0.0022815

1,3,14,17,8,-0.0053147

1,3,14,18,1,0.0015639

1,3,14,18,2,-0.0079304

1,3,14,18,3,-0.01456

1,3,14,18,4,-0.010191

1,3,14,18,5,-0.0053002

1,3,14,18,6,-0.0039105

1,3,14,18,7,-0.0032011

1,3,14,18,8,-0.0064151

1,3,14,19,1,0.00057452

1,3,14,19,2,-0.011062

1,3,14,19,3,-0.011469

1,3,14,19,4,-0.010643

1,3,14,19,5,-0.0042377

1,3,14,19,6,-0.0026673

1,3,14,19,7,-0.0012777

1,3,14,19,8,-0.0040423

1,3,14,20,1,0.00122

1,3,14,20,2,-0.0010948

1,3,14,20,3,-0.0057718

1,3,14,20,4,-0.0061706

1,3,14,20,5,-0.0025758

1,3,14,20,6,-0.0015396

1,3,14,20,7,-0.0037684

1,3,14,20,8,-0.0031928

1,3,15,15,1,0.0010882

1,3,15,15,2,-0.001471

1,3,15,15,3,-0.0032046

1,3,15,15,4,-0.0019974

1,3,15,15,5,0.00043516

1,3,15,15,6,-0.00056109

1,3,15,15,7,-0.0015186

1,3,15,15,8,-0.00046798

1,3,15,16,1,1.538e-05

1,3,15,16,2,-0.00066382

1,3,15,16,3,-0.0033452

1,3,15,16,4,-0.0022494

1,3,15,16,5,0.00029918

1,3,15,16,6,-0.00079709

1,3,15,16,7,-0.00077452

1,3,15,16,8,-0.0047705

1,3,15,17,1,0.001725

1,3,15,17,2,-0.0015057

1,3,15,17,3,-0.0092402

1,3,15,17,4,-0.00803

1,3,15,17,5,-0.0032726

1,3,15,17,6,-0.0024926

1,3,15,17,7,-0.0017399

1,3,15,17,8,-0.0053856

1,3,15,18,1,1.043e-11

1,3,15,18,2,-0.0041882

1,3,15,18,3,-0.00368

1,3,15,18,4,-0.0097424

1,3,15,18,5,-0.0036053

1,3,15,18,6,-0.0029465

1,3,15,18,7,-0.0028646

1,3,15,18,8,-0.0052526

1,3,15,19,1,0.00090399

1,3,15,19,2,-0.0006559

1,3,15,19,3,-0.0063255

1,3,15,19,4,-0.005819

1,3,15,19,5,-0.00064158

1,3,15,19,6,-0.0009808

1,3,15,19,7,-0.0021428

1,3,15,19,8,-0.0036891

1,3,15,20,1,1.5095e-10

1,3,15,20,2,-0.0011381

1,3,15,20,3,-0.0032583

1,3,15,20,4,-0.0045096

1,3,15,20,5,-0.0023392

1,3,15,20,6,-0.0024947

1,3,15,20,7,-0.0029347

1,3,15,20,8,-0.0010199

1,3,16,16,1,3.2871e-12

1,3,16,16,2,-0.0073027

1,3,16,16,3,-0.001822

1,3,16,16,4,-0.0036544

1,3,16,16,5,0.0019789

1,3,16,16,6,-0.00029566

1,3,16,16,7,0.0012096

1,3,16,16,8,0.0014373

1,3,16,17,1,0.001555

1,3,16,17,2,-0.0043003

1,3,16,17,3,-0.0054861

1,3,16,17,4,-0.0062003

1,3,16,17,5,-0.00052865

1,3,16,17,6,-0.00039595

1,3,16,17,7,-0.00018203

1,3,16,17,8,-0.0034546

1,3,16,18,1,5.4359e-05

1,3,16,18,2,-0.0042106

1,3,16,18,3,-0.0043728

1,3,16,18,4,-0.0033044

1,3,16,18,5,-0.00095358

1,3,16,18,6,-0.0020136

1,3,16,18,7,-0.00091129

1,3,16,18,8,-0.0031759

1,3,16,19,1,0.0010636

1,3,16,19,2,0.0014155

1,3,16,19,3,-0.0013187

1,3,16,19,4,-0.00074354

1,3,16,19,5,0.0023931

1,3,16,19,6,-0.00069746

1,3,16,19,7,-0.00035536

1,3,16,19,8,-0.0020676

1,3,16,20,1,0.00039444

1,3,16,20,2,-0.00014416

1,3,16,20,3,-0.0074708

1,3,16,20,4,-0.0027953

1,3,16,20,5,0.0008812

1,3,16,20,6,0.00043762

1,3,16,20,7,-0.0025652

1,3,16,20,8,-0.0016061

1,3,17,17,1,0.010916

1,3,17,17,2,-0.00083008

1,3,17,17,3,-0.00343

1,3,17,17,4,-0.0039011

1,3,17,17,5,-0.0018785

1,3,17,17,6,-0.0019815

1,3,17,17,7,-0.0010437

1,3,17,17,8,-0.0025187

1,3,17,18,1,0.0022083

1,3,17,18,2,-0.0066386

1,3,17,18,3,-0.010905

1,3,17,18,4,-0.01004

1,3,17,18,5,-0.0048304

1,3,17,18,6,-0.0042359

1,3,17,18,7,-0.0044168

1,3,17,18,8,-0.0050642

1,3,17,19,1,7.8345e-05

1,3,17,19,2,-0.0061396

1,3,17,19,3,-0.0088959

1,3,17,19,4,-0.0088916

1,3,17,19,5,-0.003991

1,3,17,19,6,-0.0035222

1,3,17,19,7,-0.0017855

1,3,17,19,8,-0.0042452

1,3,17,20,1,0.0013488

1,3,17,20,2,-0.0078663

1,3,17,20,3,-0.0079995

1,3,17,20,4,-0.0087315

1,3,17,20,5,-0.0047244

1,3,17,20,6,-0.003915

1,3,17,20,7,-0.0023005

1,3,17,20,8,-0.0065617

1,3,18,18,1,0.0027886

1,3,18,18,2,-0.004337

1,3,18,18,3,-0.0062529

1,3,18,18,4,-0.0058805

1,3,18,18,5,-0.0037071

1,3,18,18,6,-0.0020034

1,3,18,18,7,-0.001474

1,3,18,18,8,-0.0035742

1,3,18,19,1,0.0014403

1,3,18,19,2,-6.0568e-05

1,3,18,19,3,-0.0081284

1,3,18,19,4,-0.0093282

1,3,18,19,5,-0.00091051

1,3,18,19,6,-0.0034411

1,3,18,19,7,-0.0019202

1,3,18,19,8,-0.0045078

1,3,18,20,1,4.2149e-05

1,3,18,20,2,-0.0043518

1,3,18,20,3,-0.011409

1,3,18,20,4,-0.0074703

1,3,18,20,5,-0.0026381

1,3,18,20,6,-0.0021243

1,3,18,20,7,-0.0038925

1,3,18,20,8,-0.0055471

1,3,19,19,1,0.0023878

1,3,19,19,2,-0.0017103

1,3,19,19,3,-0.0055374

1,3,19,19,4,-0.0034686

1,3,19,19,5,-0.002254

1,3,19,19,6,-0.0013051

1,3,19,19,7,0.00037477

1,3,19,19,8,-0.0025893

1,3,19,20,1,0.00061081

1,3,19,20,2,-0.0041471

1,3,19,20,3,-0.008844

1,3,19,20,4,-0.00714

1,3,19,20,5,-0.0027391

1,3,19,20,6,-0.0010713

1,3,19,20,7,-0.0011643

1,3,19,20,8,-0.0046131

1,3,20,20,1,0.00025641

1,3,20,20,2,-0.0038423

1,3,20,20,3,-0.0040311

1,3,20,20,4,-0.002143

1,3,20,20,5,-0.0030742

1,3,20,20,6,-0.00050883

1,3,20,20,7,9.7154e-05

1,3,20,20,8,-0.0026416

1,4,1,1,1,0.0036399

1,4,1,1,2,-0.0026337

1,4,1,1,3,-0.0016289

1,4,1,1,4,-0.0011076

1,4,1,1,5,0.00018184

1,4,1,1,6,-0.00012572

1,4,1,1,7,-9.0864e-05

1,4,1,1,8,-0.0002411

1,4,1,2,1,1.634e-09

1,4,1,2,2,-0.00066582

1,4,1,2,3,-0.0025919

1,4,1,2,4,-7.0794e-05

1,4,1,2,5,0.00062316

1,4,1,2,6,0.00098469

1,4,1,2,7,-0.00037967

1,4,1,2,8,0.00053573

1,4,1,3,1,0.0042557

1,4,1,3,2,0.00019009

1,4,1,3,3,-0.006834

1,4,1,3,4,-0.0024336

1,4,1,3,5,0.00026867

1,4,1,3,6,-0.00083218

1,4,1,3,7,0.00017668

1,4,1,3,8,-0.00047371

1,4,1,4,1,0.0047476

1,4,1,4,2,0.00035487

1,4,1,4,3,-0.0031199

1,4,1,4,4,-0.0012065

1,4,1,4,5,0.00081111

1,4,1,4,6,0.0003696

1,4,1,4,7,0.00066545

1,4,1,4,8,-0.00011901

1,4,1,5,1,0.0057137

1,4,1,5,2,-0.0020067

1,4,1,5,3,-0.0024627

1,4,1,5,4,-0.0018532

1,4,1,5,5,0.0010702

1,4,1,5,6,-0.00028582

1,4,1,5,7,0.00021038

1,4,1,5,8,2.6116e-05

1,4,1,6,1,0.0034625

1,4,1,6,2,-0.0012024

1,4,1,6,3,-0.0048207

1,4,1,6,4,-0.0017279

1,4,1,6,5,0.00082125

1,4,1,6,6,-0.00017266

1,4,1,6,7,0.0002778

1,4,1,6,8,-0.00050201

1,4,1,7,1,0.002806

1,4,1,7,2,0.00099108

1,4,1,7,3,-0.0041457

1,4,1,7,4,-0.0035416

1,4,1,7,5,0.00097451

1,4,1,7,6,-0.0011263

1,4,1,7,7,0.00085655

1,4,1,7,8,-0.00043156

1,4,1,8,1,0.004455

1,4,1,8,2,-0.00053982

1,4,1,8,3,-0.0043849

1,4,1,8,4,-0.0012469

1,4,1,8,5,0.00036272

1,4,1,8,6,-0.00081927

1,4,1,8,7,0.0003577

1,4,1,8,8,-0.00086584

1,4,1,9,1,0.0056643

1,4,1,9,2,-0.00071248

1,4,1,9,3,-0.0037998

1,4,1,9,4,-0.001488

1,4,1,9,5,0.00084508

1,4,1,9,6,0.00020636

1,4,1,9,7,0.00061981

1,4,1,9,8,-0.00026322

1,4,1,10,1,0.0049448

1,4,1,10,2,-0.0023305

1,4,1,10,3,-0.0029947

1,4,1,10,4,-0.0027911

1,4,1,10,5,0.0010703

1,4,1,10,6,-0.00072313

1,4,1,10,7,0.0010341

1,4,1,10,8,-0.00038199

1,4,1,11,1,0.0039938

1,4,1,11,2,0.00053938

1,4,1,11,3,-0.0041009

1,4,1,11,4,-0.0028296

1,4,1,11,5,0.0006943

1,4,1,11,6,-8.5482e-05

1,4,1,11,7,0.00050196

1,4,1,11,8,-0.00068631

1,4,1,12,1,0.0016926

1,4,1,12,2,-0.0010311

1,4,1,12,3,-0.0041503

1,4,1,12,4,-0.002892

1,4,1,12,5,0.00092876

1,4,1,12,6,-0.00019447

1,4,1,12,7,0.00045793

1,4,1,12,8,-0.00082149

1,4,1,13,1,0.0068559

1,4,1,13,2,0.00054423

1,4,1,13,3,-0.0031396

1,4,1,13,4,-9.99e-05

1,4,1,13,5,0.0010024

1,4,1,13,6,0.001817

1,4,1,13,7,0.00011077

1,4,1,13,8,0.0001311

1,4,1,14,1,0.0065261

1,4,1,14,2,-0.00027005

1,4,1,14,3,-0.0030946

1,4,1,14,4,-0.00029039

1,4,1,14,5,0.0016159

1,4,1,14,6,0.00069766

1,4,1,14,7,0.00038382

1,4,1,14,8,-0.0001954

1,4,1,15,1,0.0023233

1,4,1,15,2,-0.00051613

1,4,1,15,3,-0.0019666

1,4,1,15,4,-0.00046116

1,4,1,15,5,-2.9064e-05

1,4,1,15,6,2.6402e-05

1,4,1,15,7,0.00057733

1,4,1,15,8,-0.0004019

1,4,1,16,1,0.0029385

1,4,1,16,2,0.00095749

1,4,1,16,3,-0.0028021

1,4,1,16,4,-0.00028554

1,4,1,16,5,0.0010628

1,4,1,16,6,0.00024529

1,4,1,16,7,-4.7681e-05

1,4,1,16,8,-0.00093095

1,4,1,17,1,0.0082685

1,4,1,17,2,0.001798

1,4,1,17,3,-0.0026763

1,4,1,17,4,-0.00047821

1,4,1,17,5,0.00065086

1,4,1,17,6,0.001094

1,4,1,17,7,0.00056126

1,4,1,17,8,-0.00024687

1,4,1,18,1,0.0040804

1,4,1,18,2,0.0019549

1,4,1,18,3,-0.003595

1,4,1,18,4,-0.00095346

1,4,1,18,5,0.0013183

1,4,1,18,6,0.00052473

1,4,1,18,7,0.00065899

1,4,1,18,8,0.00024842

1,4,1,19,1,0.003288

1,4,1,19,2,0.0022314

1,4,1,19,3,-0.0037332

1,4,1,19,4,-0.0016842

1,4,1,19,5,0.00062575

1,4,1,19,6,0.0002931

1,4,1,19,7,0.0008189

1,4,1,19,8,-0.00028716

1,4,1,20,1,0.0015871

1,4,1,20,2,0.00012454

1,4,1,20,3,-0.0025528

1,4,1,20,4,-0.00089502

1,4,1,20,5,-6.2078e-05

1,4,1,20,6,0.00064213

1,4,1,20,7,-0.00019696

1,4,1,20,8,0.00024411

1,4,2,2,1,9.0102e-12

1,4,2,2,2,-0.00052323

1,4,2,2,3,-0.0010676

1,4,2,2,4,-0.00088838

1,4,2,2,5,-0.00032355

1,4,2,2,6,-0.00083702

1,4,2,2,7,-5.0301e-05

1,4,2,2,8,-0.00026913

1,4,2,3,1,0.0012049

1,4,2,3,2,0.00069312

1,4,2,3,3,-0.0038492

1,4,2,3,4,-0.0015199

1,4,2,3,5,7.0099e-05

1,4,2,3,6,-0.00091868

1,4,2,3,7,-0.00042328

1,4,2,3,8,-4.0125e-05

1,4,2,4,1,1.1331e-10

1,4,2,4,2,0.0033199

1,4,2,4,3,-0.0029175

1,4,2,4,4,-0.0022345

1,4,2,4,5,-0.00022528

1,4,2,4,6,-0.00070461

1,4,2,4,7,0.00011746

1,4,2,4,8,-0.00012725

1,4,2,5,1,2.4856e-10

1,4,2,5,2,0.00074191

1,4,2,5,3,-0.0030256

1,4,2,5,4,-0.0021184

1,4,2,5,5,-2.0323e-05

1,4,2,5,6,-0.000792

1,4,2,5,7,-6.6769e-06

1,4,2,5,8,-6.6272e-07

1,4,2,6,1,0.0015242

1,4,2,6,2,-0.0013846

1,4,2,6,3,-0.0035948

1,4,2,6,4,-0.00047703

1,4,2,6,5,-0.0003912

1,4,2,6,6,1.793e-05

1,4,2,6,7,-0.00046419

1,4,2,6,8,-0.00027115

1,4,2,7,1,2.5782e-05

1,4,2,7,2,0.00086512

1,4,2,7,3,-0.0020024

1,4,2,7,4,-0.0026192

1,4,2,7,5,-0.00037519

1,4,2,7,6,-0.0011332

1,4,2,7,7,0.0001136

1,4,2,7,8,-0.00020209

1,4,2,8,1,0.0016524

1,4,2,8,2,0.00022329

1,4,2,8,3,-0.0034106

1,4,2,8,4,-0.001854

1,4,2,8,5,-6.994e-05

1,4,2,8,6,-0.0013904

1,4,2,8,7,-0.0002989

1,4,2,8,8,-4.9858e-05

1,4,2,9,1,0.00042708

1,4,2,9,2,-0.0003589

1,4,2,9,3,-0.002891

1,4,2,9,4,-0.00072009

1,4,2,9,5,-0.00025306

1,4,2,9,6,-0.00047619

1,4,2,9,7,0.000293

1,4,2,9,8,-0.00046423

1,4,2,10,1,5.0318e-12

1,4,2,10,2,0.0013166

1,4,2,10,3,-0.0019741

1,4,2,10,4,-0.0022485

1,4,2,10,5,0.00032998

1,4,2,10,6,-0.0011688

1,4,2,10,7,-0.00010513

1,4,2,10,8,-0.00042862

1,4,2,11,1,9.5686e-05

1,4,2,11,2,-0.00041436

1,4,2,11,3,-0.0032707

1,4,2,11,4,-0.003032

1,4,2,11,5,-0.00048221

1,4,2,11,6,-0.0015081

1,4,2,11,7,8.7817e-05

1,4,2,11,8,-0.0013506

1,4,2,12,1,0.0011551

1,4,2,12,2,0.0001339

1,4,2,12,3,-0.0019635

1,4,2,12,4,-0.0036339

1,4,2,12,5,0.00019003

1,4,2,12,6,-0.0013986

1,4,2,12,7,-0.00057357

1,4,2,12,8,-0.00068401

1,4,2,13,1,0.0025246

1,4,2,13,2,-0.0027671

1,4,2,13,3,-0.003736

1,4,2,13,4,-0.0024298

1,4,2,13,5,-0.00046863

1,4,2,13,6,-0.00036197

1,4,2,13,7,0.00029667

1,4,2,13,8,3.7207e-05

1,4,2,14,1,0.0011219

1,4,2,14,2,0.00014174

1,4,2,14,3,-0.0019524

1,4,2,14,4,-0.002475

1,4,2,14,5,-0.0005968

1,4,2,14,6,-0.00061654

1,4,2,14,7,-0.00029398

1,4,2,14,8,0.00014668

1,4,2,15,1,0.00084416

1,4,2,15,2,-1.9988e-05

1,4,2,15,3,-0.0027318

1,4,2,15,4,-0.0018076

1,4,2,15,5,-0.00064772

1,4,2,15,6,0.0003126

1,4,2,15,7,-0.00031283

1,4,2,15,8,-7.723e-05

1,4,2,16,1,0.00093897

1,4,2,16,2,-0.0006295

1,4,2,16,3,-0.0024959

1,4,2,16,4,0.00010103

1,4,2,16,5,-0.00065721

1,4,2,16,6,-0.00025221

1,4,2,16,7,0.00026519

1,4,2,16,8,0.00023956

1,4,2,17,1,0.0018835

1,4,2,17,2,0.0013394

1,4,2,17,3,-0.0017205

1,4,2,17,4,-0.0022185

1,4,2,17,5,0.00017144

1,4,2,17,6,-0.00071328

1,4,2,17,7,0.00067281

1,4,2,17,8,-0.00016105

1,4,2,18,1,9.5991e-12

1,4,2,18,2,0.0019641

1,4,2,18,3,-0.0024322

1,4,2,18,4,-0.0021194

1,4,2,18,5,-6.0311e-05

1,4,2,18,6,-0.00018323

1,4,2,18,7,0.00022862

1,4,2,18,8,0.00019308

1,4,2,19,1,1.282e-12

1,4,2,19,2,0.0014453

1,4,2,19,3,-0.0028363

1,4,2,19,4,-0.0019565

1,4,2,19,5,-3.2125e-05

1,4,2,19,6,-0.00096428

1,4,2,19,7,0.00061033

1,4,2,19,8,-0.00019346

1,4,2,20,1,1.1346e-11

1,4,2,20,2,-0.00010338

1,4,2,20,3,-0.0020993

1,4,2,20,4,-0.001041

1,4,2,20,5,-0.0010586

1,4,2,20,6,-0.0005039

1,4,2,20,7,-0.00048208

1,4,2,20,8,0.00026118

1,4,3,3,1,0.0041524

1,4,3,3,2,0.0009198

1,4,3,3,3,-0.0022759

1,4,3,3,4,-0.0010471

1,4,3,3,5,0.00073636

1,4,3,3,6,-5.7707e-05

1,4,3,3,7,-0.0002165

1,4,3,3,8,-9.9705e-05

1,4,3,4,1,0.0062125

1,4,3,4,2,0.0024719

1,4,3,4,3,-0.0020617

1,4,3,4,4,-0.00094745

1,4,3,4,5,0.0019223

1,4,3,4,6,1.8386e-05

1,4,3,4,7,0.0012417

1,4,3,4,8,7.4123e-05

1,4,3,5,1,0.0059213

1,4,3,5,2,0.0011676

1,4,3,5,3,-0.0032259

1,4,3,5,4,-0.0023682

1,4,3,5,5,0.0012806

1,4,3,5,6,-0.00044858

1,4,3,5,7,0.00034854

1,4,3,5,8,-0.00036107

1,4,3,6,1,0.0038088

1,4,3,6,2,0.0016689

1,4,3,6,3,-0.0073576

1,4,3,6,4,-0.0041603

1,4,3,6,5,0.0012157

1,4,3,6,6,-0.0006098

1,4,3,6,7,0.00060765

1,4,3,6,8,-0.00029727

1,4,3,7,1,0.0035983

1,4,3,7,2,0.00052349

1,4,3,7,3,-0.0024012

1,4,3,7,4,-0.0021996

1,4,3,7,5,0.0013726

1,4,3,7,6,0.00021933

1,4,3,7,7,0.00060392

1,4,3,7,8,-0.00037058

1,4,3,8,1,0.0052174

1,4,3,8,2,-0.00017043

1,4,3,8,3,-0.0080407

1,4,3,8,4,-0.0028624

1,4,3,8,5,-0.00030216

1,4,3,8,6,-0.00062687

1,4,3,8,7,0.00046839

1,4,3,8,8,-0.00055305

1,4,3,9,1,0.0048244

1,4,3,9,2,0.0013811

1,4,3,9,3,-0.0048983

1,4,3,9,4,-0.0022038

1,4,3,9,5,9.8193e-05

1,4,3,9,6,5.1238e-05

1,4,3,9,7,0.00092265

1,4,3,9,8,-0.00067591

1,4,3,10,1,0.0049299

1,4,3,10,2,-0.0025773

1,4,3,10,3,-0.0073996

1,4,3,10,4,-0.0045236

1,4,3,10,5,-0.00011768

1,4,3,10,6,-0.0018592

1,4,3,10,7,-4.5903e-05

1,4,3,10,8,-0.0014401

1,4,3,11,1,0.0046153

1,4,3,11,2,-0.00057213

1,4,3,11,3,-0.0063019

1,4,3,11,4,-0.0032593

1,4,3,11,5,-0.0003845

1,4,3,11,6,-0.0019821

1,4,3,11,7,-6.1893e-06

1,4,3,11,8,-0.0015291

1,4,3,12,1,0.0023155

1,4,3,12,2,-7.0272e-05

1,4,3,12,3,-0.004883

1,4,3,12,4,-0.003511

1,4,3,12,5,-0.0004206

1,4,3,12,6,-0.0010188

1,4,3,12,7,-0.00030233

1,4,3,12,8,-0.001388

1,4,3,13,1,0.0053357

1,4,3,13,2,0.002223

1,4,3,13,3,-0.00052418

1,4,3,13,4,-0.0015225

1,4,3,13,5,0.0016079

1,4,3,13,6,0.0011558

1,4,3,13,7,0.00099988

1,4,3,13,8,-0.00036499

1,4,3,14,1,0.0049357

1,4,3,14,2,0.001916

1,4,3,14,3,-0.0018235

1,4,3,14,4,-0.0016863

1,4,3,14,5,0.0018259

1,4,3,14,6,0.00029947

1,4,3,14,7,0.0014298

1,4,3,14,8,-0.00049499

1,4,3,15,1,0.0019388

1,4,3,15,2,-0.00012677

1,4,3,15,3,-0.003508

1,4,3,15,4,-0.0020036

1,4,3,15,5,0.00080795

1,4,3,15,6,-0.00049605

1,4,3,15,7,0.00035925

1,4,3,15,8,-0.00056071

1,4,3,16,1,0.0018165

1,4,3,16,2,0.0012216

1,4,3,16,3,-0.0022169

1,4,3,16,4,-0.0015867

1,4,3,16,5,0.0017884

1,4,3,16,6,-0.00039704

1,4,3,16,7,0.00092345

1,4,3,16,8,0.00018673

1,4,3,17,1,0.0076999

1,4,3,17,2,0.0037434

1,4,3,17,3,-0.0016563

1,4,3,17,4,-0.001659

1,4,3,17,5,0.0015707

1,4,3,17,6,0.00063508

1,4,3,17,7,0.0011168

1,4,3,17,8,-0.00066282

1,4,3,18,1,0.003328

1,4,3,18,2,0.0012301

1,4,3,18,3,-0.0026612

1,4,3,18,4,-0.002518

1,4,3,18,5,0.0021626

1,4,3,18,6,-0.00014293

1,4,3,18,7,0.00080776

1,4,3,18,8,-0.00061514

1,4,3,19,1,0.002274

1,4,3,19,2,-0.00091481

1,4,3,19,3,-0.0023741

1,4,3,19,4,-0.0031523

1,4,3,19,5,0.0013773

1,4,3,19,6,-0.0013791

1,4,3,19,7,-0.00050437

1,4,3,19,8,-0.00091248

1,4,3,20,1,0.0014268

1,4,3,20,2,0.00024236

1,4,3,20,3,-0.0021369

1,4,3,20,4,-0.0026954

1,4,3,20,5,0.0012899

1,4,3,20,6,-0.00093839

1,4,3,20,7,0.00080533

1,4,3,20,8,-0.0014429

1,4,4,4,1,0.0076559

1,4,4,4,2,7.8032e-05

1,4,4,4,3,-0.00022127

1,4,4,4,4,-0.0013837

1,4,4,4,5,0.0002736

1,4,4,4,6,-0.00054929

1,4,4,4,7,4.5218e-05

1,4,4,4,8,-0.00010977

1,4,4,5,1,0.0066214

1,4,4,5,2,0.0015008

1,4,4,5,3,-0.0014453

1,4,4,5,4,-0.0031823

1,4,4,5,5,0.00072055

1,4,4,5,6,-0.0013875

1,4,4,5,7,0.00075851

1,4,4,5,8,-0.0005521

1,4,4,6,1,0.0050943

1,4,4,6,2,0.00025718

1,4,4,6,3,-0.0025759

1,4,4,6,4,-0.0018814

1,4,4,6,5,0.0018081

1,4,4,6,6,-0.00034839

1,4,4,6,7,0.0012182

1,4,4,6,8,-0.00046673

1,4,4,7,1,0.0038932

1,4,4,7,2,0.0013328

1,4,4,7,3,-0.0027764

1,4,4,7,4,-0.0027419

1,4,4,7,5,0.0003982

1,4,4,7,6,-0.00049107

1,4,4,7,7,0.00026343

1,4,4,7,8,-0.0008856

1,4,4,8,1,0.0042132

1,4,4,8,2,-5.6025e-05

1,4,4,8,3,-0.0042313

1,4,4,8,4,-0.0023236

1,4,4,8,5,0.00037991

1,4,4,8,6,-0.00050801

1,4,4,8,7,0.00018497

1,4,4,8,8,0.00019645

1,4,4,9,1,0.0050731

1,4,4,9,2,0.00038771

1,4,4,9,3,-0.0033915

1,4,4,9,4,-0.0010544

1,4,4,9,5,-0.00039496

1,4,4,9,6,-0.00080521

1,4,4,9,7,0.0002306

1,4,4,9,8,-0.00048104

1,4,4,10,1,0.0070428

1,4,4,10,2,-0.0024971

1,4,4,10,3,-0.0045579

1,4,4,10,4,-0.0037235

1,4,4,10,5,-0.00083801

1,4,4,10,6,-0.00094464

1,4,4,10,7,-0.00023636

1,4,4,10,8,-3.8161e-05

1,4,4,11,1,0.0038046

1,4,4,11,2,0.00099308

1,4,4,11,3,-0.0045732

1,4,4,11,4,-0.0037279

1,4,4,11,5,-0.00069295

1,4,4,11,6,-0.0013409

1,4,4,11,7,4.6717e-05

1,4,4,11,8,-0.00085101

1,4,4,12,1,0.0019536

1,4,4,12,2,0.0017122

1,4,4,12,3,-0.0021597

1,4,4,12,4,-0.0023988

1,4,4,12,5,0.0008368

1,4,4,12,6,-0.0012344

1,4,4,12,7,0.0010383

1,4,4,12,8,-0.000891

1,4,4,13,1,0.0064026

1,4,4,13,2,-4.3656e-05

1,4,4,13,3,-0.0019656

1,4,4,13,4,-0.0034905

1,4,4,13,5,0.00034525

1,4,4,13,6,-0.00046371

1,4,4,13,7,0.00040843

1,4,4,13,8,-0.00077886

1,4,4,14,1,0.0062257

1,4,4,14,2,-0.00031797

1,4,4,14,3,-0.0024741

1,4,4,14,4,-0.0024968

1,4,4,14,5,-0.00031945

1,4,4,14,6,-0.00048206

1,4,4,14,7,-0.00029295

1,4,4,14,8,-0.0011497

1,4,4,15,1,0.0020932

1,4,4,15,2,0.00056825

1,4,4,15,3,-0.00074267

1,4,4,15,4,-0.0031822

1,4,4,15,5,0.00040972

1,4,4,15,6,-0.00026543

1,4,4,15,7,0.00080109

1,4,4,15,8,-0.00037616

1,4,4,16,1,0.0017214

1,4,4,16,2,0.00098319

1,4,4,16,3,-0.0016525

1,4,4,16,4,-0.0011305

1,4,4,16,5,0.00047128

1,4,4,16,6,-0.00076237

1,4,4,16,7,0.00029012

1,4,4,16,8,-8.9108e-05

1,4,4,17,1,0.0089814

1,4,4,17,2,0.0026683

1,4,4,17,3,-0.0012761

1,4,4,17,4,-0.0033844

1,4,4,17,5,0.00035541

1,4,4,17,6,-0.0014421

1,4,4,17,7,0.00038844

1,4,4,17,8,-0.00080476

1,4,4,18,1,0.0039826

1,4,4,18,2,0.00059655

1,4,4,18,3,-0.0020445

1,4,4,18,4,-0.0022694

1,4,4,18,5,0.00037495

1,4,4,18,6,-0.0015489

1,4,4,18,7,0.00019352

1,4,4,18,8,-0.00094795

1,4,4,19,1,0.0032867

1,4,4,19,2,0.0022361

1,4,4,19,3,-0.0022035

1,4,4,19,4,-0.0016937

1,4,4,19,5,-5.6259e-05

1,4,4,19,6,-0.0011785

1,4,4,19,7,-2.3707e-05

1,4,4,19,8,-0.00089004

1,4,4,20,1,0.0015704

1,4,4,20,2,-0.0001338

1,4,4,20,3,-0.0012504

1,4,4,20,4,-0.0014965

1,4,4,20,5,0.00063306

1,4,4,20,6,-0.00074117

1,4,4,20,7,-0.00018733

1,4,4,20,8,-0.00056193

1,4,5,5,1,0.0098251

1,4,5,5,2,-0.0013816

1,4,5,5,3,-0.00095875

1,4,5,5,4,-0.0023584

1,4,5,5,5,0.00048445

1,4,5,5,6,-0.00083134

1,4,5,5,7,9.0473e-05

1,4,5,5,8,-0.00019278

1,4,5,6,1,0.0050473

1,4,5,6,2,0.00092371

1,4,5,6,3,-0.0030578

1,4,5,6,4,-0.0028186

1,4,5,6,5,0.0017504

1,4,5,6,6,-0.00032243

1,4,5,6,7,0.00070654

1,4,5,6,8,-0.00051406

1,4,5,7,1,0.0049281

1,4,5,7,2,0.00049971

1,4,5,7,3,-0.0035277

1,4,5,7,4,-0.0037385

1,4,5,7,5,0.0010534

1,4,5,7,6,-0.00092433

1,4,5,7,7,0.00034486

1,4,5,7,8,-0.00073208

1,4,5,8,1,0.0046212

1,4,5,8,2,0.0011296

1,4,5,8,3,-0.0037705

1,4,5,8,4,-0.0030949

1,4,5,8,5,0.0012832

1,4,5,8,6,-0.00056313

1,4,5,8,7,0.00015828

1,4,5,8,8,-0.00044654

1,4,5,9,1,0.0047956

1,4,5,9,2,0.001019

1,4,5,9,3,-0.0030076

1,4,5,9,4,-0.0024025

1,4,5,9,5,0.00011056

1,4,5,9,6,-0.00052065

1,4,5,9,7,8.6772e-06

1,4,5,9,8,-7.3598e-05

1,4,5,10,1,0.0073617

1,4,5,10,2,0.0016699

1,4,5,10,3,-0.002648

1,4,5,10,4,-0.0032951

1,4,5,10,5,0.00093934

1,4,5,10,6,-0.00098152

1,4,5,10,7,7.3049e-05

1,4,5,10,8,-0.00085538

1,4,5,11,1,0.0050567

1,4,5,11,2,0.002189

1,4,5,11,3,-0.0041894

1,4,5,11,4,-0.0033879

1,4,5,11,5,0.00025457

1,4,5,11,6,-0.0010554

1,4,5,11,7,0.00030077

1,4,5,11,8,-0.00097175

1,4,5,12,1,0.0026333

1,4,5,12,2,0.001179

1,4,5,12,3,-0.0034435

1,4,5,12,4,-0.0026445

1,4,5,12,5,0.001268

1,4,5,12,6,-0.00086546

1,4,5,12,7,6.3489e-05

1,4,5,12,8,0.00017952

1,4,5,13,1,0.0096309

1,4,5,13,2,-0.00025115

1,4,5,13,3,-0.0038824

1,4,5,13,4,-0.0030691

1,4,5,13,5,-0.00088802

1,4,5,13,6,0.00020744

1,4,5,13,7,5.3989e-05

1,4,5,13,8,-0.00035724

1,4,5,14,1,0.0062864

1,4,5,14,2,-0.0011996

1,4,5,14,3,-0.0040618

1,4,5,14,4,-0.0037654

1,4,5,14,5,-0.0014109

1,4,5,14,6,-0.0014367

1,4,5,14,7,0.00019674

1,4,5,14,8,-0.00058568

1,4,5,15,1,0.0023142

1,4,5,15,2,0.00028885

1,4,5,15,3,-0.003086

1,4,5,15,4,-0.0029723

1,4,5,15,5,-0.00064572

1,4,5,15,6,-0.0010779

1,4,5,15,7,-0.00070693

1,4,5,15,8,0.00025092

1,4,5,16,1,0.001648

1,4,5,16,2,-0.00029912

1,4,5,16,3,-0.0015428

1,4,5,16,4,-0.001065

1,4,5,16,5,0.0005731

1,4,5,16,6,-0.00099929

1,4,5,16,7,0.00055401

1,4,5,16,8,-0.00058322

1,4,5,17,1,0.010839

1,4,5,17,2,0.0024593

1,4,5,17,3,-0.0036164

1,4,5,17,4,-0.0040192

1,4,5,17,5,-0.0010253

1,4,5,17,6,-0.0015389

1,4,5,17,7,0.00023338

1,4,5,17,8,-0.00046016

1,4,5,18,1,0.0032446

1,4,5,18,2,0.001535

1,4,5,18,3,-0.0035174

1,4,5,18,4,-0.0035479

1,4,5,18,5,-0.00049798

1,4,5,18,6,-0.0019704

1,4,5,18,7,-0.00023733

1,4,5,18,8,-0.00065077

1,4,5,19,1,0.0033458

1,4,5,19,2,-0.00021018

1,4,5,19,3,-0.0037233

1,4,5,19,4,-0.0040293

1,4,5,19,5,-0.00016149

1,4,5,19,6,-0.001411

1,4,5,19,7,-5.4355e-05

1,4,5,19,8,-0.00073427

1,4,5,20,1,0.0015226

1,4,5,20,2,0.0010422

1,4,5,20,3,-0.0017578

1,4,5,20,4,-0.0031981

1,4,5,20,5,-0.00037997

1,4,5,20,6,-0.0010846

1,4,5,20,7,-0.00043089

1,4,5,20,8,-0.00086173

1,4,6,6,1,0.0036708

1,4,6,6,2,-0.00018065

1,4,6,6,3,-0.0043912

1,4,6,6,4,-0.0022239

1,4,6,6,5,-0.00066357

1,4,6,6,6,-0.00086494

1,4,6,6,7,3.3243e-05

1,4,6,6,8,-0.00073237

1,4,6,7,1,0.0034758

1,4,6,7,2,0.001067

1,4,6,7,3,-0.004573

1,4,6,7,4,-0.0031026

1,4,6,7,5,0.00070671

1,4,6,7,6,-0.0006075

1,4,6,7,7,0.00042953

1,4,6,7,8,-0.00029297

1,4,6,8,1,0.0039785

1,4,6,8,2,-0.00056598

1,4,6,8,3,-0.0061236

1,4,6,8,4,-0.0034058

1,4,6,8,5,-0.00038207

1,4,6,8,6,-0.0012269

1,4,6,8,7,0.00024555

1,4,6,8,8,-0.00094313

1,4,6,9,1,0.0038636

1,4,6,9,2,-2.4561e-05

1,4,6,9,3,-0.0041484

1,4,6,9,4,-0.0021945

1,4,6,9,5,-0.00023742

1,4,6,9,6,0.00010974

1,4,6,9,7,-0.00010728

1,4,6,9,8,-5.3447e-05

1,4,6,10,1,0.0041627

1,4,6,10,2,-0.00089483

1,4,6,10,3,-0.0059066

1,4,6,10,4,-0.0034948

1,4,6,10,5,0.0017244

1,4,6,10,6,-0.00092418

1,4,6,10,7,0.0004891

1,4,6,10,8,-0.00064838

1,4,6,11,1,0.0038162

1,4,6,11,2,9.2977e-05

1,4,6,11,3,-0.0046955

1,4,6,11,4,-0.0037053

1,4,6,11,5,0.00023929

1,4,6,11,6,-0.0001642

1,4,6,11,7,-6.5657e-05

1,4,6,11,8,-0.0010583

1,4,6,12,1,0.0018843

1,4,6,12,2,0.0010734

1,4,6,12,3,-0.0034548

1,4,6,12,4,-0.0039695

1,4,6,12,5,0.00012633

1,4,6,12,6,-0.00044658

1,4,6,12,7,0.00045183

1,4,6,12,8,-0.00085811

1,4,6,13,1,0.0054754

1,4,6,13,2,0.00099082

1,4,6,13,3,-0.00081018

1,4,6,13,4,-0.0012177

1,4,6,13,5,0.0016946

1,4,6,13,6,0.00080108

1,4,6,13,7,0.0016731

1,4,6,13,8,-0.0007704

1,4,6,14,1,0.0042316

1,4,6,14,2,0.00093852

1,4,6,14,3,-0.0016723

1,4,6,14,4,-0.0023279

1,4,6,14,5,0.0010263

1,4,6,14,6,0.00037616

1,4,6,14,7,0.0010388

1,4,6,14,8,-0.00076513

1,4,6,15,1,0.0021385

1,4,6,15,2,0.0014106

1,4,6,15,3,-0.0027988

1,4,6,15,4,-0.0020501

1,4,6,15,5,0.0009381

1,4,6,15,6,-3.0032e-05

1,4,6,15,7,-0.00026385

1,4,6,15,8,-0.0012576

1,4,6,16,1,0.0012136

1,4,6,16,2,0.00084684

1,4,6,16,3,-0.0036847

1,4,6,16,4,-0.0018845

1,4,6,16,5,0.00018283

1,4,6,16,6,-0.0013836

1,4,6,16,7,0.00072122

1,4,6,16,8,-0.0010769

1,4,6,17,1,0.0061249

1,4,6,17,2,0.0027622

1,4,6,17,3,-0.0014927

1,4,6,17,4,-0.0032062

1,4,6,17,5,0.0020878

1,4,6,17,6,7.7439e-05

1,4,6,17,7,0.00097814

1,4,6,17,8,-0.00044898

1,4,6,18,1,0.003369

1,4,6,18,2,0.0014137

1,4,6,18,3,-0.0030936

1,4,6,18,4,-0.0018799

1,4,6,18,5,0.00069325

1,4,6,18,6,0.00017216

1,4,6,18,7,0.00089494

1,4,6,18,8,4.4127e-05

1,4,6,19,1,0.0026106

1,4,6,19,2,-0.00012883

1,4,6,19,3,-0.0016123

1,4,6,19,4,-0.0029694

1,4,6,19,5,0.00039075

1,4,6,19,6,0.00036519

1,4,6,19,7,-0.00020172

1,4,6,19,8,-0.00042371

1,4,6,20,1,0.0011113

1,4,6,20,2,-0.00080316

1,4,6,20,3,-0.00259

1,4,6,20,4,-0.0024758

1,4,6,20,5,0.00017812

1,4,6,20,6,-5.2471e-05

1,4,6,20,7,-0.00059586

1,4,6,20,8,-0.00025198

1,4,7,7,1,0.0025648

1,4,7,7,2,0.00027711

1,4,7,7,3,-0.00033492

1,4,7,7,4,-0.0020612

1,4,7,7,5,0.00031154

1,4,7,7,6,0.00065401

1,4,7,7,7,-0.00030695

1,4,7,7,8,-4.996e-06

1,4,7,8,1,0.0040719

1,4,7,8,2,0.0002632

1,4,7,8,3,-0.0035303

1,4,7,8,4,-0.0027738

1,4,7,8,5,0.00070223

1,4,7,8,6,-0.00043248

1,4,7,8,7,0.00043424

1,4,7,8,8,-0.00065555

1,4,7,9,1,0.001876

1,4,7,9,2,7.664e-05

1,4,7,9,3,-0.005061

1,4,7,9,4,-0.0021254

1,4,7,9,5,-0.00079359

1,4,7,9,6,0.00013061

1,4,7,9,7,9.3594e-05

1,4,7,9,8,-0.0011452

1,4,7,10,1,0.0039714

1,4,7,10,2,0.00010889

1,4,7,10,3,-0.0044387

1,4,7,10,4,-0.0025639

1,4,7,10,5,0.00042307

1,4,7,10,6,0.000109

1,4,7,10,7,0.0002337

1,4,7,10,8,-9.619e-05

1,4,7,11,1,0.002323

1,4,7,11,2,-0.00073822

1,4,7,11,3,-0.0035587

1,4,7,11,4,-0.0025403

1,4,7,11,5,-0.0010522

1,4,7,11,6,-0.00080861

1,4,7,11,7,0.00024708

1,4,7,11,8,-0.0003539

1,4,7,12,1,0.0012006

1,4,7,12,2,0.00075609

1,4,7,12,3,-0.00063181

1,4,7,12,4,-0.0028853

1,4,7,12,5,0.0021679

1,4,7,12,6,-0.00033329

1,4,7,12,7,-0.00019223

1,4,7,12,8,-0.00053076

1,4,7,13,1,0.0047167

1,4,7,13,2,0.00028808

1,4,7,13,3,-0.0032892

1,4,7,13,4,-0.0032729

1,4,7,13,5,0.00053021

1,4,7,13,6,0.0009416

1,4,7,13,7,0.0003691

1,4,7,13,8,-0.00080514

1,4,7,14,1,0.0039744

1,4,7,14,2,-0.0012065

1,4,7,14,3,-0.0026286

1,4,7,14,4,-0.0040163

1,4,7,14,5,-0.00025723

1,4,7,14,6,-4.085e-05

1,4,7,14,7,0.00032219

1,4,7,14,8,-0.00061983

1,4,7,15,1,0.0015005

1,4,7,15,2,0.00075621

1,4,7,15,3,-0.0021547

1,4,7,15,4,-0.0028277

1,4,7,15,5,0.00031712

1,4,7,15,6,-0.0007535

1,4,7,15,7,0.00047555

1,4,7,15,8,-0.00099339

1,4,7,16,1,0.0016865

1,4,7,16,2,2.8232e-05

1,4,7,16,3,-0.0024728

1,4,7,16,4,-0.0024327

1,4,7,16,5,0.00053218

1,4,7,16,6,6.0772e-05

1,4,7,16,7,-0.00035581

1,4,7,16,8,8.2655e-05

1,4,7,17,1,0.0055347

1,4,7,17,2,0.0030965

1,4,7,17,3,-0.0029011

1,4,7,17,4,-0.0034252

1,4,7,17,5,0.00094156

1,4,7,17,6,-0.00067211

1,4,7,17,7,0.00069257

1,4,7,17,8,-0.0014946

1,4,7,18,1,0.0022321

1,4,7,18,2,0.00088005

1,4,7,18,3,-0.0023421

1,4,7,18,4,-0.0026561

1,4,7,18,5,0.00076489

1,4,7,18,6,0.00024648

1,4,7,18,7,0.00043912

1,4,7,18,8,-0.00069686

1,4,7,19,1,0.0028342

1,4,7,19,2,-3.0871e-05

1,4,7,19,3,-0.0034466

1,4,7,19,4,-0.0032287

1,4,7,19,5,-0.0001134

1,4,7,19,6,-0.0011966

1,4,7,19,7,0.00019293

1,4,7,19,8,-0.00069368

1,4,7,20,1,0.00067236

1,4,7,20,2,2.6147e-05

1,4,7,20,3,-0.0025801

1,4,7,20,4,-0.0038575

1,4,7,20,5,-0.00015826

1,4,7,20,6,-0.00091003

1,4,7,20,7,-0.00099947

1,4,7,20,8,-8.7044e-06

1,4,8,8,1,0.0064073

1,4,8,8,2,0.000388

1,4,8,8,3,-0.0024023

1,4,8,8,4,-0.00024563

1,4,8,8,5,-0.00016081

1,4,8,8,6,0.00055722

1,4,8,8,7,-0.00020685

1,4,8,8,8,7.2386e-05

1,4,8,9,1,0.0051681

1,4,8,9,2,-0.0021373

1,4,8,9,3,-0.0044862

1,4,8,9,4,-0.0007596

1,4,8,9,5,0.0003996

1,4,8,9,6,-0.00037399

1,4,8,9,7,0.00015736

1,4,8,9,8,-0.00024015

1,4,8,10,1,0.0055461

1,4,8,10,2,-0.0025686

1,4,8,10,3,-0.0039326

1,4,8,10,4,-0.0022811

1,4,8,10,5,0.00016559

1,4,8,10,6,-9.7386e-05

1,4,8,10,7,7.6966e-05

1,4,8,10,8,-0.00054618

1,4,8,11,1,0.0046769

1,4,8,11,2,-0.00021259

1,4,8,11,3,-0.0046674

1,4,8,11,4,-0.0033966

1,4,8,11,5,0.00049769

1,4,8,11,6,-0.0011321

1,4,8,11,7,0.00042138

1,4,8,11,8,-0.00055976

1,4,8,12,1,0.0031808

1,4,8,12,2,0.001321

1,4,8,12,3,-0.0051015

1,4,8,12,4,-0.002913

1,4,8,12,5,0.00077588

1,4,8,12,6,-0.00035818

1,4,8,12,7,-0.00013318

1,4,8,12,8,-0.00037823

1,4,8,13,1,0.0074129

1,4,8,13,2,0.00018531

1,4,8,13,3,-0.0029954

1,4,8,13,4,-0.0022216

1,4,8,13,5,0.00098812

1,4,8,13,6,0.00017416

1,4,8,13,7,0.00072494

1,4,8,13,8,-0.00063332

1,4,8,14,1,0.0054252

1,4,8,14,2,-0.00048883

1,4,8,14,3,-0.0026032

1,4,8,14,4,-0.0018654

1,4,8,14,5,0.00095979

1,4,8,14,6,0.00032161

1,4,8,14,7,0.00079179

1,4,8,14,8,-0.00097907

1,4,8,15,1,0.0019088

1,4,8,15,2,-7.4964e-05

1,4,8,15,3,-0.0043818

1,4,8,15,4,-0.0025915

1,4,8,15,5,-0.00045327

1,4,8,15,6,-0.00035597

1,4,8,15,7,0.00033119

1,4,8,15,8,0.0002567

1,4,8,16,1,0.0024126

1,4,8,16,2,0.0011807

1,4,8,16,3,-0.0029186

1,4,8,16,4,-0.0017415

1,4,8,16,5,0.00081171

1,4,8,16,6,-0.00079009

1,4,8,16,7,0.000637

1,4,8,16,8,-0.00075635

1,4,8,17,1,0.006406

1,4,8,17,2,0.0030345

1,4,8,17,3,-0.0021278

1,4,8,17,4,-0.0020752

1,4,8,17,5,0.0020808

1,4,8,17,6,0.00012429

1,4,8,17,7,0.0009345

1,4,8,17,8,-0.00018494

1,4,8,18,1,0.004643

1,4,8,18,2,0.0011168

1,4,8,18,3,-0.003866

1,4,8,18,4,-0.0031412

1,4,8,18,5,0.0018417

1,4,8,18,6,-0.00012635

1,4,8,18,7,0.00087171

1,4,8,18,8,-0.00070653

1,4,8,19,1,0.0030154

1,4,8,19,2,-0.00076825

1,4,8,19,3,-0.0039091

1,4,8,19,4,-0.0015436

1,4,8,19,5,0.00083086

1,4,8,19,6,1.6232e-05

1,4,8,19,7,0.00044395

1,4,8,19,8,-0.00068096

1,4,8,20,1,0.0011273

1,4,8,20,2,0.0012355

1,4,8,20,3,-0.0031555

1,4,8,20,4,-0.0033013

1,4,8,20,5,0.0013532

1,4,8,20,6,-0.00044537

1,4,8,20,7,7.6946e-05

1,4,8,20,8,-0.0008077

1,4,9,9,1,0.0057006

1,4,9,9,2,-0.0032577

1,4,9,9,3,-0.00093292

1,4,9,9,4,-0.00051052

1,4,9,9,5,-0.00037725

1,4,9,9,6,-0.00046643

1,4,9,9,7,0.00028105

1,4,9,9,8,-0.00048953

1,4,9,10,1,0.0047646

1,4,9,10,2,-0.00071326

1,4,9,10,3,-0.0038131

1,4,9,10,4,-0.0014192

1,4,9,10,5,-0.00079307

1,4,9,10,6,0.00055987

1,4,9,10,7,-0.00014306

1,4,9,10,8,-0.00030734

1,4,9,11,1,0.0045215

1,4,9,11,2,-8.0846e-06

1,4,9,11,3,-0.0043231

1,4,9,11,4,-0.0026546

1,4,9,11,5,-0.00078413

1,4,9,11,6,-0.00069792

1,4,9,11,7,-7.1076e-05

1,4,9,11,8,-0.00043773

1,4,9,12,1,0.0021166

1,4,9,12,2,0.00030012

1,4,9,12,3,-0.003667

1,4,9,12,4,-0.0016058

1,4,9,12,5,0.00060338

1,4,9,12,6,-0.00088172

1,4,9,12,7,0.00025389

1,4,9,12,8,-0.00044403

1,4,9,13,1,0.0071159

1,4,9,13,2,-0.0010915

1,4,9,13,3,-0.0022581

1,4,9,13,4,-0.0025548

1,4,9,13,5,0.00011093

1,4,9,13,6,-0.00059562

1,4,9,13,7,0.00068121

1,4,9,13,8,-0.00030393

1,4,9,14,1,0.0055136

1,4,9,14,2,-0.0014393

1,4,9,14,3,-0.0029189

1,4,9,14,4,-0.0031177

1,4,9,14,5,-0.00016434

1,4,9,14,6,-0.00083973

1,4,9,14,7,-0.00019251

1,4,9,14,8,-0.00072759

1,4,9,15,1,0.0018523

1,4,9,15,2,0.0012433

1,4,9,15,3,-0.0018502

1,4,9,15,4,-0.0020066

1,4,9,15,5,9.0213e-05

1,4,9,15,6,-0.0011833

1,4,9,15,7,0.00048008

1,4,9,15,8,-0.0004721

1,4,9,16,1,0.0028071

1,4,9,16,2,-0.0012229

1,4,9,16,3,-0.0022464

1,4,9,16,4,-0.0013388

1,4,9,16,5,-0.0008671

1,4,9,16,6,-0.00032848

1,4,9,16,7,-0.00054595

1,4,9,16,8,0.00022374

1,4,9,17,1,0.0081343

1,4,9,17,2,0.00071447

1,4,9,17,3,-0.0031919

1,4,9,17,4,-0.0030768

1,4,9,17,5,-0.00012447

1,4,9,17,6,-0.0012515

1,4,9,17,7,0.00032663

1,4,9,17,8,-0.00054308

1,4,9,18,1,0.0030415

1,4,9,18,2,0.00061072

1,4,9,18,3,-0.0026673

1,4,9,18,4,-0.0024186

1,4,9,18,5,0.00082613

1,4,9,18,6,-0.0011029

1,4,9,18,7,0.00078057

1,4,9,18,8,-0.00054901

1,4,9,19,1,0.0025322

1,4,9,19,2,0.00051717

1,4,9,19,3,-0.004175

1,4,9,19,4,-0.0020618

1,4,9,19,5,0.00051163

1,4,9,19,6,-0.00094979

1,4,9,19,7,-0.0001145

1,4,9,19,8,-0.00031081

1,4,9,20,1,0.0011598

1,4,9,20,2,0.00058773

1,4,9,20,3,-0.0025527

1,4,9,20,4,-0.0018402

1,4,9,20,5,-0.00025901

1,4,9,20,6,-0.0010227

1,4,9,20,7,0.00014259

1,4,9,20,8,-0.0003961

1,4,10,10,1,0.0069804

1,4,10,10,2,-0.0017432

1,4,10,10,3,-0.00047392

1,4,10,10,4,-0.00078237

1,4,10,10,5,0.00078

1,4,10,10,6,-0.00018444

1,4,10,10,7,0.00023314

1,4,10,10,8,9.3969e-05

1,4,10,11,1,0.0050444

1,4,10,11,2,0.0010797

1,4,10,11,3,-0.00093729

1,4,10,11,4,-0.0031011

1,4,10,11,5,0.00097367

1,4,10,11,6,7.3549e-05

1,4,10,11,7,0.0012191

1,4,10,11,8,-1.0375e-05

1,4,10,12,1,0.0019282

1,4,10,12,2,0.0004762

1,4,10,12,3,-0.0024834

1,4,10,12,4,-0.0034798

1,4,10,12,5,0.00016894

1,4,10,12,6,-0.00024045

1,4,10,12,7,-0.00061885

1,4,10,12,8,-0.00027611

1,4,10,13,1,0.0071654

1,4,10,13,2,0.0010191

1,4,10,13,3,-0.0039598

1,4,10,13,4,-0.0019262

1,4,10,13,5,0.00041268

1,4,10,13,6,-0.00038278

1,4,10,13,7,0.00049663

1,4,10,13,8,-0.0010697

1,4,10,14,1,0.0060269

1,4,10,14,2,0.00066961

1,4,10,14,3,-0.0026225

1,4,10,14,4,-0.0021396

1,4,10,14,5,0.00067423

1,4,10,14,6,-0.00050548

1,4,10,14,7,0.00081418

1,4,10,14,8,-0.0009619

1,4,10,15,1,0.0025215

1,4,10,15,2,0.00013474

1,4,10,15,3,-0.0029541

1,4,10,15,4,-0.0029894

1,4,10,15,5,0.00083255

1,4,10,15,6,-0.00073492

1,4,10,15,7,-6.6007e-05

1,4,10,15,8,-0.00017697

1,4,10,16,1,0.0020499

1,4,10,16,2,0.00054124

1,4,10,16,3,-0.0021236

1,4,10,16,4,-0.0016362

1,4,10,16,5,0.00054914

1,4,10,16,6,-0.0014441

1,4,10,16,7,2.8337e-06

1,4,10,16,8,-0.00049886

1,4,10,17,1,0.0092171

1,4,10,17,2,0.0031135

1,4,10,17,3,-0.0044033

1,4,10,17,4,-0.0028119

1,4,10,17,5,-1.7453e-05

1,4,10,17,6,8.6486e-05

1,4,10,17,7,0.00051959

1,4,10,17,8,-0.00055194

1,4,10,18,1,0.0035192

1,4,10,18,2,-0.0001008

1,4,10,18,3,-0.0034217

1,4,10,18,4,-0.004246

1,4,10,18,5,4.9914e-05

1,4,10,18,6,-0.0010002

1,4,10,18,7,0.00026203

1,4,10,18,8,-0.0010104

1,4,10,19,1,0.0046536

1,4,10,19,2,-0.0010266

1,4,10,19,3,-0.0051347

1,4,10,19,4,-0.0037037

1,4,10,19,5,-0.00040201

1,4,10,19,6,-0.00063242

1,4,10,19,7,0.00013562

1,4,10,19,8,-0.0010506

1,4,10,20,1,0.0015934

1,4,10,20,2,-0.0010006

1,4,10,20,3,-0.0028213

1,4,10,20,4,-0.0043105

1,4,10,20,5,0.00034292

1,4,10,20,6,-0.0014265

1,4,10,20,7,-0.00040854

1,4,10,20,8,0.00035948

1,4,11,11,1,0.0050847

1,4,11,11,2,0.001075

1,4,11,11,3,-0.0011653

1,4,11,11,4,-0.001268

1,4,11,11,5,0.00041467

1,4,11,11,6,0.00014999

1,4,11,11,7,-8.7527e-05

1,4,11,11,8,0.00012568

1,4,11,12,1,0.0017819

1,4,11,12,2,-0.0014368

1,4,11,12,3,-0.0028548

1,4,11,12,4,-0.0037179

1,4,11,12,5,5.0473e-06

1,4,11,12,6,-0.0012939

1,4,11,12,7,0.00016589

1,4,11,12,8,-0.0015486

1,4,11,13,1,0.005567

1,4,11,13,2,4.8962e-05

1,4,11,13,3,-0.001895

1,4,11,13,4,-0.0034592

1,4,11,13,5,0.00045341

1,4,11,13,6,0.0003134

1,4,11,13,7,0.00063179

1,4,11,13,8,-0.0012528

1,4,11,14,1,0.0044805

1,4,11,14,2,0.00011303

1,4,11,14,3,-0.0027351

1,4,11,14,4,-0.0032889

1,4,11,14,5,0.00037825

1,4,11,14,6,0.00050168

1,4,11,14,7,0.00011372

1,4,11,14,8,-0.00047778

1,4,11,15,1,0.002106

1,4,11,15,2,0.0011106

1,4,11,15,3,-0.0026578

1,4,11,15,4,-0.0028662

1,4,11,15,5,0.0003164

1,4,11,15,6,-6.3705e-05

1,4,11,15,7,0.00034025

1,4,11,15,8,-0.00080238

1,4,11,16,1,0.0022411

1,4,11,16,2,-4.2726e-05

1,4,11,16,3,-0.0018058

1,4,11,16,4,-0.002397

1,4,11,16,5,-0.00024273

1,4,11,16,6,-0.00071645

1,4,11,16,7,0.00024634

1,4,11,16,8,0.00016735

1,4,11,17,1,0.0079751

1,4,11,17,2,0.0025812

1,4,11,17,3,-0.0035299

1,4,11,17,4,-0.0037532

1,4,11,17,5,0.00044758

1,4,11,17,6,-0.0010352

1,4,11,17,7,0.00043262

1,4,11,17,8,-0.00091319

1,4,11,18,1,0.0031297

1,4,11,18,2,0.0002027

1,4,11,18,3,-0.0053091

1,4,11,18,4,-0.0030968

1,4,11,18,5,-0.00085353

1,4,11,18,6,-0.00032808

1,4,11,18,7,-6.7335e-05

1,4,11,18,8,-0.0012435

1,4,11,19,1,0.0028795

1,4,11,19,2,-0.0001677

1,4,11,19,3,-0.0052884

1,4,11,19,4,-0.0029845

1,4,11,19,5,-0.0010059

1,4,11,19,6,-0.0006029

1,4,11,19,7,-0.00084782

1,4,11,19,8,-0.00096617

1,4,11,20,1,0.0012157

1,4,11,20,2,-0.00047833

1,4,11,20,3,-0.0036886

1,4,11,20,4,-0.0037877

1,4,11,20,5,-0.00056003

1,4,11,20,6,0.0003459

1,4,11,20,7,-0.00054498

1,4,11,20,8,-0.0008325

1,4,12,12,1,0.0012937

1,4,12,12,2,0.00025759

1,4,12,12,3,-0.002338

1,4,12,12,4,-0.0024672

1,4,12,12,5,-0.00070978

1,4,12,12,6,-0.00088763

1,4,12,12,7,-0.00038831

1,4,12,12,8,-0.0010382

1,4,12,13,1,0.0017104

1,4,12,13,2,-0.00074271

1,4,12,13,3,-0.0030773

1,4,12,13,4,-0.0034755

1,4,12,13,5,0.00075607

1,4,12,13,6,-0.00079989

1,4,12,13,7,0.00075203

1,4,12,13,8,-0.00078976

1,4,12,14,1,0.0022545

1,4,12,14,2,0.0014653

1,4,12,14,3,-0.0034703

1,4,12,14,4,-0.0020644

1,4,12,14,5,0.00098431

1,4,12,14,6,0.00013387

1,4,12,14,7,0.00072193

1,4,12,14,8,-0.0012492

1,4,12,15,1,0.0013034

1,4,12,15,2,-0.00052743

1,4,12,15,3,-0.0032214

1,4,12,15,4,-0.0036805

1,4,12,15,5,0.00025174

1,4,12,15,6,0.00026591

1,4,12,15,7,-1.715e-05

1,4,12,15,8,-0.00055285

1,4,12,16,1,0.0014011

1,4,12,16,2,0.00088951

1,4,12,16,3,-0.0032072

1,4,12,16,4,-0.0042103

1,4,12,16,5,0.0005863

1,4,12,16,6,-0.0023501

1,4,12,16,7,-0.00094559

1,4,12,16,8,-0.00031656

1,4,12,17,1,0.0033106

1,4,12,17,2,0.0028494

1,4,12,17,3,-0.0024917

1,4,12,17,4,-0.003606

1,4,12,17,5,9.1148e-05

1,4,12,17,6,-0.00088395

1,4,12,17,7,0.0011818

1,4,12,17,8,-0.00059498

1,4,12,18,1,0.0017777

1,4,12,18,2,0.00036931

1,4,12,18,3,-0.0043531

1,4,12,18,4,-0.0034236

1,4,12,18,5,-0.00031764

1,4,12,18,6,-0.0011554

1,4,12,18,7,-0.00055674

1,4,12,18,8,-0.001369

1,4,12,19,1,0.0017889

1,4,12,19,2,4.172e-05

1,4,12,19,3,-0.0036903

1,4,12,19,4,-0.0028341

1,4,12,19,5,0.00060657

1,4,12,19,6,-0.0012645

1,4,12,19,7,0.00041665

1,4,12,19,8,-0.0010687

1,4,12,20,1,0.00071543

1,4,12,20,2,0.0010598

1,4,12,20,3,-0.0018523

1,4,12,20,4,-0.0028138

1,4,12,20,5,0.00026056

1,4,12,20,6,-0.0014265

1,4,12,20,7,0.00029111

1,4,12,20,8,-0.0022062

1,4,13,13,1,0.010786

1,4,13,13,2,-0.0015027

1,4,13,13,3,-0.0023936

1,4,13,13,4,-0.001456

1,4,13,13,5,-0.0010792

1,4,13,13,6,-0.00094908

1,4,13,13,7,0.00019624

1,4,13,13,8,-0.00071347

1,4,13,14,1,0.0086634

1,4,13,14,2,-0.0029986

1,4,13,14,3,-0.0049585

1,4,13,14,4,-0.0032104

1,4,13,14,5,-0.0021298

1,4,13,14,6,-0.0017405

1,4,13,14,7,3.024e-05

1,4,13,14,8,-0.0010156

1,4,13,15,1,0.0019498

1,4,13,15,2,0.00012356

1,4,13,15,3,-0.0045453

1,4,13,15,4,-0.002719

1,4,13,15,5,-0.00079262

1,4,13,15,6,-0.0014201

1,4,13,15,7,-0.00017667

1,4,13,15,8,-0.0012439

1,4,13,16,1,0.0021718

1,4,13,16,2,-0.0017668

1,4,13,16,3,-0.0029416

1,4,13,16,4,-0.0026837

1,4,13,16,5,-0.00036618

1,4,13,16,6,-0.00097854

1,4,13,16,7,0.00022684

1,4,13,16,8,-0.00031724

1,4,13,17,1,0.010907

1,4,13,17,2,0.00035745

1,4,13,17,3,-0.00513

1,4,13,17,4,-0.0039317

1,4,13,17,5,-0.0023038

1,4,13,17,6,-0.0015947

1,4,13,17,7,-0.00041412

1,4,13,17,8,-0.00064155

1,4,13,18,1,0.0047378

1,4,13,18,2,-0.000816

1,4,13,18,3,-0.0048981

1,4,13,18,4,-0.0028507

1,4,13,18,5,-0.0015331

1,4,13,18,6,-0.001742

1,4,13,18,7,-0.00015115

1,4,13,18,8,-0.0012494

1,4,13,19,1,0.0033203

1,4,13,19,2,-0.00032673

1,4,13,19,3,-0.0053543

1,4,13,19,4,-0.0024273

1,4,13,19,5,-0.00068245

1,4,13,19,6,-0.0015314

1,4,13,19,7,0.00052198

1,4,13,19,8,-0.00098568

1,4,13,20,1,0.0015011

1,4,13,20,2,-0.00073749

1,4,13,20,3,-0.0025046

1,4,13,20,4,-0.0036033

1,4,13,20,5,-1.2163e-05

1,4,13,20,6,-0.0005616

1,4,13,20,7,-0.00022044

1,4,13,20,8,-0.00041841

1,4,14,14,1,0.006293

1,4,14,14,2,-0.0023985

1,4,14,14,3,-0.0030766

1,4,14,14,4,-0.00141

1,4,14,14,5,-0.0018885

1,4,14,14,6,-0.00068055

1,4,14,14,7,-0.00031026

1,4,14,14,8,-0.000431

1,4,14,15,1,0.0019882

1,4,14,15,2,-3.8697e-05

1,4,14,15,3,-0.0039945

1,4,14,15,4,-0.0037161

1,4,14,15,5,-0.0019593

1,4,14,15,6,-0.0017445

1,4,14,15,7,-6.7535e-05

1,4,14,15,8,-0.00082596

1,4,14,16,1,0.0019857

1,4,14,16,2,-0.00079309

1,4,14,16,3,-0.0032966

1,4,14,16,4,-0.0017256

1,4,14,16,5,-0.0016299

1,4,14,16,6,-0.0015026

1,4,14,16,7,-0.00015512

1,4,14,16,8,-0.00077466

1,4,14,17,1,0.007512

1,4,14,17,2,-0.00044908

1,4,14,17,3,-0.0057982

1,4,14,17,4,-0.0046869

1,4,14,17,5,-0.0025798

1,4,14,17,6,-0.0031411

1,4,14,17,7,-0.00052845

1,4,14,17,8,-0.0010574

1,4,14,18,1,0.0027374

1,4,14,18,2,-0.00021736

1,4,14,18,3,-0.0054274

1,4,14,18,4,-0.0027791

1,4,14,18,5,-0.0019224

1,4,14,18,6,-0.0016527

1,4,14,18,7,-0.00065984

1,4,14,18,8,-0.0011185

1,4,14,19,1,0.0027619

1,4,14,19,2,-0.0020377

1,4,14,19,3,-0.0057846

1,4,14,19,4,-0.0030514

1,4,14,19,5,-0.0020172

1,4,14,19,6,-0.00086127

1,4,14,19,7,-0.00067434

1,4,14,19,8,-0.00048727

1,4,14,20,1,0.0015381

1,4,14,20,2,9.4739e-05

1,4,14,20,3,-0.0030758

1,4,14,20,4,-0.0033488

1,4,14,20,5,-0.00087204

1,4,14,20,6,-0.0011234

1,4,14,20,7,-0.00047436

1,4,14,20,8,-0.00035508

1,4,15,15,1,0.0013237

1,4,15,15,2,-0.00048947

1,4,15,15,3,-0.0030877

1,4,15,15,4,-0.001863

1,4,15,15,5,-0.00058887

1,4,15,15,6,-0.00067554

1,4,15,15,7,0.00010253

1,4,15,15,8,-0.00029174

1,4,15,16,1,0.00064729

1,4,15,16,2,0.00026778

1,4,15,16,3,-0.0020339

1,4,15,16,4,-0.0014376

1,4,15,16,5,-0.00025898

1,4,15,16,6,-0.0011066

1,4,15,16,7,0.00022207

1,4,15,16,8,-0.00068135

1,4,15,17,1,0.0032776

1,4,15,17,2,0.0016948

1,4,15,17,3,-0.0050914

1,4,15,17,4,-0.004863

1,4,15,17,5,-0.0017367

1,4,15,17,6,-0.002357

1,4,15,17,7,-0.00054492

1,4,15,17,8,-0.001186

1,4,15,18,1,0.0010151

1,4,15,18,2,9.6203e-05

1,4,15,18,3,-0.002342

1,4,15,18,4,-0.0038137

1,4,15,18,5,-0.00093092

1,4,15,18,6,-0.001467

1,4,15,18,7,-0.00018686

1,4,15,18,8,-0.0012161

1,4,15,19,1,0.0010255

1,4,15,19,2,-0.00075417

1,4,15,19,3,-0.002625

1,4,15,19,4,-0.0022659

1,4,15,19,5,-0.00069364

1,4,15,19,6,-0.00076868

1,4,15,19,7,-0.00077598

1,4,15,19,8,-0.00052755

1,4,15,20,1,0.00049296

1,4,15,20,2,-0.00017609

1,4,15,20,3,-0.0014805

1,4,15,20,4,-0.0020626

1,4,15,20,5,-0.0015373

1,4,15,20,6,-0.0012562

1,4,15,20,7,-0.00035136

1,4,15,20,8,0.00079725

1,4,16,16,1,3.1845e-12

1,4,16,16,2,-0.0049531

1,4,16,16,3,-0.0030225

1,4,16,16,4,-0.0030205

1,4,16,16,5,3.8474e-07

1,4,16,16,6,-0.0013799

1,4,16,16,7,-2.4203e-05

1,4,16,16,8,0.00063322

1,4,16,17,1,0.0026684

1,4,16,17,2,0.00045979

1,4,16,17,3,-0.003532

1,4,16,17,4,-0.0023082

1,4,16,17,5,-0.00092739

1,4,16,17,6,-0.0010384

1,4,16,17,7,0.00045789

1,4,16,17,8,-0.00036227

1,4,16,18,1,0.00088036

1,4,16,18,2,-0.0019716

1,4,16,18,3,-0.0031893

1,4,16,18,4,-0.0015601

1,4,16,18,5,-0.00080997

1,4,16,18,6,-0.0012321

1,4,16,18,7,0.00058643

1,4,16,18,8,-0.00011064

1,4,16,19,1,0.0010913

1,4,16,19,2,-0.00030483

1,4,16,19,3,-0.0028601

1,4,16,19,4,-0.0020827

1,4,16,19,5,-0.00029655

1,4,16,19,6,-0.0019832

1,4,16,19,7,-3.0189e-05

1,4,16,19,8,-0.00044804

1,4,16,20,1,0.0008192

1,4,16,20,2,0.00096044

1,4,16,20,3,-0.0033214

1,4,16,20,4,-0.00078284

1,4,16,20,5,9.6158e-05

1,4,16,20,6,-0.00010517

1,4,16,20,7,-0.0013982

1,4,16,20,8,0.00066062

1,4,17,17,1,0.011816

1,4,17,17,2,0.00053919

1,4,17,17,3,-0.0026973

1,4,17,17,4,-0.0026453

1,4,17,17,5,-0.0014182

1,4,17,17,6,-0.0015627

1,4,17,17,7,-0.00027874

1,4,17,17,8,-0.00070506

1,4,17,18,1,0.0055443

1,4,17,18,2,0.00056766

1,4,17,18,3,-0.005448

1,4,17,18,4,-0.0033823

1,4,17,18,5,-0.0024717

1,4,17,18,6,-0.0018628

1,4,17,18,7,-0.0011403

1,4,17,18,8,-0.00068741

1,4,17,19,1,0.0037611

1,4,17,19,2,-0.00049561

1,4,17,19,3,-0.0047703

1,4,17,19,4,-0.0034841

1,4,17,19,5,-0.0018997

1,4,17,19,6,-0.0018346

1,4,17,19,7,-0.00038224

1,4,17,19,8,-0.00073944

1,4,17,20,1,0.0018687

1,4,17,20,2,-0.00044185

1,4,17,20,3,-0.0041804

1,4,17,20,4,-0.0034908

1,4,17,20,5,-0.0015863

1,4,17,20,6,-0.0023229

1,4,17,20,7,-0.00067774

1,4,17,20,8,-0.0012083

1,4,18,18,1,0.0028107

1,4,18,18,2,-0.00095185

1,4,18,18,3,-0.0024556

1,4,18,18,4,-0.0013869

1,4,18,18,5,-0.0009201

1,4,18,18,6,-0.00067847

1,4,18,18,7,-0.00036703

1,4,18,18,8,-0.00014725

1,4,18,19,1,0.0026536

1,4,18,19,2,0.00067006

1,4,18,19,3,-0.003685

1,4,18,19,4,-0.003178

1,4,18,19,5,-0.0007023

1,4,18,19,6,-0.0022578

1,4,18,19,7,-0.00057182

1,4,18,19,8,-0.00058898

1,4,18,20,1,0.00077418

1,4,18,20,2,0.0002837

1,4,18,20,3,-0.0037621

1,4,18,20,4,-0.0023223

1,4,18,20,5,-0.00057649

1,4,18,20,6,-0.00090238

1,4,18,20,7,-7.4274e-05

1,4,18,20,8,-0.00026153

1,4,19,19,1,0.0028631

1,4,19,19,2,-0.0013257

1,4,19,19,3,-0.0035865

1,4,19,19,4,-0.00211

1,4,19,19,5,-0.0010557

1,4,19,19,6,-0.00098668

1,4,19,19,7,-0.00019076

1,4,19,19,8,-0.00071382

1,4,19,20,1,0.0010724

1,4,19,20,2,-0.00027105

1,4,19,20,3,-0.0039794

1,4,19,20,4,-0.0018272

1,4,19,20,5,-0.00078116

1,4,19,20,6,-0.00074809

1,4,19,20,7,-0.00098696

1,4,19,20,8,-0.00096895

1,4,20,20,1,0.00083083

1,4,20,20,2,-0.0010746

1,4,20,20,3,-0.0010363

1,4,20,20,4,-0.0012583

1,4,20,20,5,-0.00096783

1,4,20,20,6,-0.00043044

1,4,20,20,7,-0.00019338

1,4,20,20,8,-0.00097496

2,1,1,1,1,0.1091

2,1,1,1,2,-0.03482

2,1,1,1,3,-0.016895

2,1,1,1,4,-0.024601

2,1,1,1,5,0.01316

2,1,1,1,6,0.00071869

2,1,1,1,7,0.0093939

2,1,1,1,8,-0.015704

2,1,1,2,1,0.0044547

2,1,1,2,2,-0.015852

2,1,1,2,3,-0.060493

2,1,1,2,4,0.012007

2,1,1,2,5,0.010605

2,1,1,2,6,0.018347

2,1,1,2,7,-0.00062683

2,1,1,2,8,-0.024458

2,1,1,3,1,0.1369

2,1,1,3,2,0.011149

2,1,1,3,3,-0.13408

2,1,1,3,4,-0.049362

2,1,1,3,5,0.0098891

2,1,1,3,6,-0.0027214

2,1,1,3,7,-0.0075282

2,1,1,3,8,-0.026767

2,1,1,4,1,0.12747

2,1,1,4,2,0.06629

2,1,1,4,3,-0.095481

2,1,1,4,4,-0.011749

2,1,1,4,5,0.008027

2,1,1,4,6,0.01592

2,1,1,4,7,-0.0054943

2,1,1,4,8,-0.014115

2,1,1,5,1,0.15831

2,1,1,5,2,-0.012139

2,1,1,5,3,-0.037209

2,1,1,5,4,-0.040821

2,1,1,5,5,0.024625

2,1,1,5,6,0.011659

2,1,1,5,7,-0.0092153

2,1,1,5,8,-0.00086147

2,1,1,6,1,0.094148

2,1,1,6,2,0.016736

2,1,1,6,3,-0.081151

2,1,1,6,4,-0.049067

2,1,1,6,5,0.04409

2,1,1,6,6,0.0091548

2,1,1,6,7,-0.0080184

2,1,1,6,8,-0.015721

2,1,1,7,1,0.057894

2,1,1,7,2,0.015939

2,1,1,7,3,-0.069991

2,1,1,7,4,-0.065482

2,1,1,7,5,0.025816

2,1,1,7,6,-0.0014817

2,1,1,7,7,0.0076678

2,1,1,7,8,-0.019814

2,1,1,8,1,0.11821

2,1,1,8,2,0.019115

2,1,1,8,3,-0.084148

2,1,1,8,4,-0.010863

2,1,1,8,5,0.014921

2,1,1,8,6,-0.0052353

2,1,1,8,7,-0.014206

2,1,1,8,8,-0.023215

2,1,1,9,1,0.13155

2,1,1,9,2,0.019679

2,1,1,9,3,-0.083182

2,1,1,9,4,-0.0047074

2,1,1,9,5,0.01131

2,1,1,9,6,0.023151

2,1,1,9,7,-0.021425

2,1,1,9,8,-0.0027289

2,1,1,10,1,0.11348

2,1,1,10,2,-0.018218

2,1,1,10,3,-0.079765

2,1,1,10,4,-0.046347

2,1,1,10,5,0.0027666

2,1,1,10,6,0.012876

2,1,1,10,7,0.00050533

2,1,1,10,8,-0.01029

2,1,1,11,1,0.13045

2,1,1,11,2,0.052909

2,1,1,11,3,-0.080697

2,1,1,11,4,-0.080552

2,1,1,11,5,0.0074927

2,1,1,11,6,-0.0043606

2,1,1,11,7,-0.013753

2,1,1,11,8,-0.02773

2,1,1,12,1,0.032282

2,1,1,12,2,-0.04124

2,1,1,12,3,-0.050932

2,1,1,12,4,-0.054503

2,1,1,12,5,0.046879

2,1,1,12,6,-0.0016859

2,1,1,12,7,0.034349

2,1,1,12,8,-0.022748

2,1,1,13,1,0.19441

2,1,1,13,2,0.035435

2,1,1,13,3,-0.033994

2,1,1,13,4,0.010184

2,1,1,13,5,0.02258

2,1,1,13,6,0.062164

2,1,1,13,7,-0.0050288

2,1,1,13,8,0.0057265

2,1,1,14,1,0.17617

2,1,1,14,2,0.049346

2,1,1,14,3,-0.072044

2,1,1,14,4,0.010864

2,1,1,14,5,0.023053

2,1,1,14,6,0.03834

2,1,1,14,7,-0.0050118

2,1,1,14,8,0.00097591

2,1,1,15,1,0.090942

2,1,1,15,2,0.034145

2,1,1,15,3,-0.059236

2,1,1,15,4,0.0057721

2,1,1,15,5,-0.0066855

2,1,1,15,6,0.030065

2,1,1,15,7,-0.012267

2,1,1,15,8,-0.015766

2,1,1,16,1,0.058056

2,1,1,16,2,0.060793

2,1,1,16,3,-0.046643

2,1,1,16,4,-0.013463

2,1,1,16,5,0.00364

2,1,1,16,6,0.012049

2,1,1,16,7,-0.024471

2,1,1,16,8,-0.041763

2,1,1,17,1,0.209

2,1,1,17,2,0.070537

2,1,1,17,3,-0.044425

2,1,1,17,4,-0.0067231

2,1,1,17,5,0.0076212

2,1,1,17,6,0.034527

2,1,1,17,7,-0.0071615

2,1,1,17,8,-0.0011636

2,1,1,18,1,0.12356

2,1,1,18,2,0.068816

2,1,1,18,3,-0.065161

2,1,1,18,4,-0.01866

2,1,1,18,5,0.031967

2,1,1,18,6,0.015859

2,1,1,18,7,0.0022411

2,1,1,18,8,-0.0051618

2,1,1,19,1,0.10439

2,1,1,19,2,0.068587

2,1,1,19,3,-0.044381

2,1,1,19,4,-0.020704

2,1,1,19,5,0.0079156

2,1,1,19,6,0.027749

2,1,1,19,7,0.01764

2,1,1,19,8,0.0045234

2,1,1,20,1,0.056838

2,1,1,20,2,0.03713

2,1,1,20,3,-0.034952

2,1,1,20,4,-0.0020391

2,1,1,20,5,-0.0079041

2,1,1,20,6,0.013336

2,1,1,20,7,-0.01012

2,1,1,20,8,-0.003704

2,1,2,2,1,0.027916

2,1,2,2,2,-0.027163

2,1,2,2,3,0.017847

2,1,2,2,4,-0.0088674

2,1,2,2,5,0.0032577

2,1,2,2,6,-0.020018

2,1,2,2,7,0.0041897

2,1,2,2,8,0.0084961

2,1,2,3,1,0.045199

2,1,2,3,2,0.043784

2,1,2,3,3,-0.055105

2,1,2,3,4,-0.044478

2,1,2,3,5,0.0023339

2,1,2,3,6,-0.014463

2,1,2,3,7,-0.015632

2,1,2,3,8,-0.01415

2,1,2,4,1,2.8381e-08

2,1,2,4,2,0.091167

2,1,2,4,3,-0.036338

2,1,2,4,4,-0.017565

2,1,2,4,5,-0.0048064

2,1,2,4,6,-0.028004

2,1,2,4,7,-0.0082541

2,1,2,4,8,0.0033452

2,1,2,5,1,0.028886

2,1,2,5,2,0.021767

2,1,2,5,3,-0.026616

2,1,2,5,4,-0.011916

2,1,2,5,5,0.0027556

2,1,2,5,6,-0.010642

2,1,2,5,7,-0.001379

2,1,2,5,8,0.032338

2,1,2,6,1,0.035757

2,1,2,6,2,-0.018816

2,1,2,6,3,-0.042397

2,1,2,6,4,-0.0036857

2,1,2,6,5,-0.0055533

2,1,2,6,6,0.017885

2,1,2,6,7,-0.017754

2,1,2,6,8,0.0042801

2,1,2,7,1,0.00071645

2,1,2,7,2,0.049467

2,1,2,7,3,-0.052491

2,1,2,7,4,-0.066522

2,1,2,7,5,-0.023906

2,1,2,7,6,-0.01986

2,1,2,7,7,-0.025354

2,1,2,7,8,0.0027184

2,1,2,8,1,0.068632

2,1,2,8,2,0.027198

2,1,2,8,3,-0.057957

2,1,2,8,4,-0.015085

2,1,2,8,5,0.0058384

2,1,2,8,6,-0.0095887

2,1,2,8,7,0.012489

2,1,2,8,8,0.035698

2,1,2,9,1,0.027935

2,1,2,9,2,0.027083

2,1,2,9,3,-0.037119

2,1,2,9,4,0.010416

2,1,2,9,5,-0.015857

2,1,2,9,6,0.011556

2,1,2,9,7,0.0034245

2,1,2,9,8,0.015295

2,1,2,10,1,7.7831e-11

2,1,2,10,2,0.052725

2,1,2,10,3,-0.014551

2,1,2,10,4,-0.061108

2,1,2,10,5,0.019938

2,1,2,10,6,-0.038701

2,1,2,10,7,0.0052396

2,1,2,10,8,-0.0084766

2,1,2,11,1,0.021024

2,1,2,11,2,-0.023486

2,1,2,11,3,-0.059885

2,1,2,11,4,-0.024556

2,1,2,11,5,-0.00051757

2,1,2,11,6,-0.0079347

2,1,2,11,7,0.0099741

2,1,2,11,8,0.0077338

2,1,2,12,1,0.015133

2,1,2,12,2,0.021724

2,1,2,12,3,-0.02829

2,1,2,12,4,-0.06638

2,1,2,12,5,-0.0069833

2,1,2,12,6,-0.0010965

2,1,2,12,7,-0.022614

2,1,2,12,8,0.028733

2,1,2,13,1,0.044587

2,1,2,13,2,-0.023802

2,1,2,13,3,-0.034483

2,1,2,13,4,-0.026731

2,1,2,13,5,0.017585

2,1,2,13,6,0.0011695

2,1,2,13,7,0.0092532

2,1,2,13,8,0.026897

2,1,2,14,1,0.01871

2,1,2,14,2,0.0043816

2,1,2,14,3,-0.029292

2,1,2,14,4,-0.0081983

2,1,2,14,5,-0.0072261

2,1,2,14,6,0.020301

2,1,2,14,7,-0.018381

2,1,2,14,8,0.04909

2,1,2,15,1,0.022353

2,1,2,15,2,0.0051877

2,1,2,15,3,-0.036578

2,1,2,15,4,-0.041014

2,1,2,15,5,0.0010946

2,1,2,15,6,-0.0031828

2,1,2,15,7,-0.031826

2,1,2,15,8,0.015017

2,1,2,16,1,0.025526

2,1,2,16,2,0.010471

2,1,2,16,3,-0.036035

2,1,2,16,4,0.033037

2,1,2,16,5,-0.0050703

2,1,2,16,6,-0.013045

2,1,2,16,7,-0.0047253

2,1,2,16,8,0.018278

2,1,2,17,1,0.043668

2,1,2,17,2,0.039732

2,1,2,17,3,0.042572

2,1,2,17,4,-0.025267

2,1,2,17,5,0.022278

2,1,2,17,6,-0.0040264

2,1,2,17,7,0.0026385

2,1,2,17,8,0.021159

2,1,2,18,1,6.9352e-10

2,1,2,18,2,0.041804

2,1,2,18,3,0.0043148

2,1,2,18,4,-0.011365

2,1,2,18,5,0.010256

2,1,2,18,6,0.0089027

2,1,2,18,7,0.012203

2,1,2,18,8,0.034871

2,1,2,19,1,3.1856e-11

2,1,2,19,2,0.054672

2,1,2,19,3,-0.022153

2,1,2,19,4,0.011524

2,1,2,19,5,0.00047621

2,1,2,19,6,-0.010327

2,1,2,19,7,0.0096828

2,1,2,19,8,0.02638

2,1,2,20,1,6.1914e-11

2,1,2,20,2,0.044966

2,1,2,20,3,-0.0034153

2,1,2,20,4,-0.015968

2,1,2,20,5,-0.022417

2,1,2,20,6,-0.010503

2,1,2,20,7,-0.011686

2,1,2,20,8,0.028576

2,1,3,3,1,0.082211

2,1,3,3,2,0.048823

2,1,3,3,3,-0.053163

2,1,3,3,4,-0.037177

2,1,3,3,5,0.00032363

2,1,3,3,6,0.0020777

2,1,3,3,7,-0.019935

2,1,3,3,8,-0.010641

2,1,3,4,1,0.15506

2,1,3,4,2,0.039477

2,1,3,4,3,-0.069411

2,1,3,4,4,-0.032243

2,1,3,4,5,0.0204

2,1,3,4,6,-0.0087691

2,1,3,4,7,0.0019558

2,1,3,4,8,-0.02762

2,1,3,5,1,0.13606

2,1,3,5,2,0.078514

2,1,3,5,3,-0.082448

2,1,3,5,4,-0.041897

2,1,3,5,5,0.0088106

2,1,3,5,6,-5.4813e-05

2,1,3,5,7,-0.020628

2,1,3,5,8,-0.010181

2,1,3,6,1,0.076767

2,1,3,6,2,0.080207

2,1,3,6,3,-0.13886

2,1,3,6,4,-0.083044

2,1,3,6,5,0.019584

2,1,3,6,6,0.0057478

2,1,3,6,7,-0.014562

2,1,3,6,8,0.0060324

2,1,3,7,1,0.086212

2,1,3,7,2,0.030292

2,1,3,7,3,-0.033447

2,1,3,7,4,-0.054169

2,1,3,7,5,0.024582

2,1,3,7,6,0.01964

2,1,3,7,7,0.0079096

2,1,3,7,8,-0.01453

2,1,3,8,1,0.15046

2,1,3,8,2,0.055006

2,1,3,8,3,-0.18989

2,1,3,8,4,-0.058447

2,1,3,8,5,-0.0098682

2,1,3,8,6,-0.010081

2,1,3,8,7,-0.013154

2,1,3,8,8,-0.0082969

2,1,3,9,1,0.12043

2,1,3,9,2,0.058516

2,1,3,9,3,-0.075375

2,1,3,9,4,-0.044071

2,1,3,9,5,0.0017026

2,1,3,9,6,0.005093

2,1,3,9,7,-0.0012344

2,1,3,9,8,-0.0061217

2,1,3,10,1,0.12453

2,1,3,10,2,-0.022694

2,1,3,10,3,-0.156

2,1,3,10,4,-0.087899

2,1,3,10,5,-0.010338

2,1,3,10,6,-0.020463

2,1,3,10,7,-0.015244

2,1,3,10,8,-0.011463

2,1,3,11,1,0.10668

2,1,3,11,2,0.03126

2,1,3,11,3,-0.129

2,1,3,11,4,-0.051098

2,1,3,11,5,-0.017565

2,1,3,11,6,-0.014034

2,1,3,11,7,-0.034163

2,1,3,11,8,-0.012994

2,1,3,12,1,0.054402

2,1,3,12,2,0.039738

2,1,3,12,3,-0.094838

2,1,3,12,4,-0.068113

2,1,3,12,5,-9.114e-05

2,1,3,12,6,-0.011116

2,1,3,12,7,-0.015352

2,1,3,12,8,-0.016114

2,1,3,13,1,0.15208

2,1,3,13,2,0.082518

2,1,3,13,3,-0.010005

2,1,3,13,4,-0.036426

2,1,3,13,5,0.016704

2,1,3,13,6,0.018434

2,1,3,13,7,-0.017783

2,1,3,13,8,0.0064035

2,1,3,14,1,0.12746

2,1,3,14,2,0.078963

2,1,3,14,3,-0.050252

2,1,3,14,4,-0.034572

2,1,3,14,5,0.036029

2,1,3,14,6,-0.011543

2,1,3,14,7,-0.0026344

2,1,3,14,8,0.0026112

2,1,3,15,1,0.042849

2,1,3,15,2,0.0060257

2,1,3,15,3,-0.075489

2,1,3,15,4,-0.059033

2,1,3,15,5,0.016522

2,1,3,15,6,-0.030014

2,1,3,15,7,0.0048439

2,1,3,15,8,0.0034236

2,1,3,16,1,0.042539

2,1,3,16,2,0.048318

2,1,3,16,3,-0.075694

2,1,3,16,4,-0.036969

2,1,3,16,5,0.033732

2,1,3,16,6,-0.013149

2,1,3,16,7,-0.020104

2,1,3,16,8,0.010243

2,1,3,17,1,0.18326

2,1,3,17,2,0.12478

2,1,3,17,3,-0.063283

2,1,3,17,4,-0.037434

2,1,3,17,5,0.0030031

2,1,3,17,6,0.0078764

2,1,3,17,7,-0.020401

2,1,3,17,8,-0.02969

2,1,3,18,1,0.086026

2,1,3,18,2,0.038688

2,1,3,18,3,-0.058132

2,1,3,18,4,-0.044274

2,1,3,18,5,0.036971

2,1,3,18,6,-0.0054967

2,1,3,18,7,-0.01685

2,1,3,18,8,0.029264

2,1,3,19,1,0.052927

2,1,3,19,2,0.016224

2,1,3,19,3,-0.00092883

2,1,3,19,4,-0.042794

2,1,3,19,5,0.02719

2,1,3,19,6,0.0052105

2,1,3,19,7,-0.018106

2,1,3,19,8,0.01812

2,1,3,20,1,0.028886

2,1,3,20,2,0.015179

2,1,3,20,3,-0.010677

2,1,3,20,4,-0.05489

2,1,3,20,5,0.012229

2,1,3,20,6,-0.0095403

2,1,3,20,7,0.014955

2,1,3,20,8,-0.0040092

2,1,4,4,1,0.14158

2,1,4,4,2,-0.00044611

2,1,4,4,3,0.02817

2,1,4,4,4,0.0062348

2,1,4,4,5,0.0078024

2,1,4,4,6,0.011932

2,1,4,4,7,-0.0080972

2,1,4,4,8,0.029964

2,1,4,5,1,0.17693

2,1,4,5,2,0.048737

2,1,4,5,3,-0.0061019

2,1,4,5,4,-0.055888

2,1,4,5,5,0.002847

2,1,4,5,6,-0.016942

2,1,4,5,7,-0.023977

2,1,4,5,8,0.017634

2,1,4,6,1,0.13685

2,1,4,6,2,0.029287

2,1,4,6,3,-0.054256

2,1,4,6,4,-0.05987

2,1,4,6,5,0.0021977

2,1,4,6,6,-0.010633

2,1,4,6,7,-0.017092

2,1,4,6,8,-0.01349

2,1,4,7,1,0.086236

2,1,4,7,2,0.055966

2,1,4,7,3,-0.057362

2,1,4,7,4,-0.046483

2,1,4,7,5,-0.010601

2,1,4,7,6,0.0036832

2,1,4,7,7,-0.030072

2,1,4,7,8,-0.0085556

2,1,4,8,1,0.11813

2,1,4,8,2,0.042996

2,1,4,8,3,-0.083264

2,1,4,8,4,-0.022491

2,1,4,8,5,0.0087226

2,1,4,8,6,0.014251

2,1,4,8,7,0.019032

2,1,4,8,8,0.025984

2,1,4,9,1,0.12339

2,1,4,9,2,0.029708

2,1,4,9,3,-0.027376

2,1,4,9,4,-0.0053018

2,1,4,9,5,-0.0070538

2,1,4,9,6,-0.0036653

2,1,4,9,7,-0.023471

2,1,4,9,8,0.008652

2,1,4,10,1,0.16133

2,1,4,10,2,-0.0013329

2,1,4,10,3,-0.086552

2,1,4,10,4,-0.072069

2,1,4,10,5,-0.022054

2,1,4,10,6,-0.0081252

2,1,4,10,7,-0.012404

2,1,4,10,8,0.010273

2,1,4,11,1,0.090348

2,1,4,11,2,0.065266

2,1,4,11,3,-0.097662

2,1,4,11,4,-0.047333

2,1,4,11,5,-0.022517

2,1,4,11,6,-0.0012327

2,1,4,11,7,-0.01693

2,1,4,11,8,0.00052491

2,1,4,12,1,0.057317

2,1,4,12,2,0.047408

2,1,4,12,3,-0.028466

2,1,4,12,4,-0.048843

2,1,4,12,5,0.013543

2,1,4,12,6,-0.015483

2,1,4,12,7,0.00035565

2,1,4,12,8,0.00074968

2,1,4,13,1,0.15983

2,1,4,13,2,0.036127

2,1,4,13,3,-0.023521

2,1,4,13,4,-0.047633

2,1,4,13,5,0.001248

2,1,4,13,6,0.0038606

2,1,4,13,7,-0.018387

2,1,4,13,8,0.032901

2,1,4,14,1,0.15471

2,1,4,14,2,0.027547

2,1,4,14,3,-0.013295

2,1,4,14,4,-0.02311

2,1,4,14,5,-0.0049722

2,1,4,14,6,-0.00064829

2,1,4,14,7,-0.039866

2,1,4,14,8,0.02847

2,1,4,15,1,0.060528

2,1,4,15,2,0.0043088

2,1,4,15,3,0.0065473

2,1,4,15,4,-0.057733

2,1,4,15,5,0.0059158

2,1,4,15,6,-0.0091344

2,1,4,15,7,-0.027794

2,1,4,15,8,0.026308

2,1,4,16,1,0.015881

2,1,4,16,2,0.013431

2,1,4,16,3,0.011684

2,1,4,16,4,0.030779

2,1,4,16,5,0.010446

2,1,4,16,6,0.013506

2,1,4,16,7,-0.0053621

2,1,4,16,8,-0.012364

2,1,4,17,1,0.20215

2,1,4,17,2,0.085292

2,1,4,17,3,0.00076416

2,1,4,17,4,-0.028866

2,1,4,17,5,0.01328

2,1,4,17,6,-0.015002

2,1,4,17,7,-0.022473

2,1,4,17,8,0.019878

2,1,4,18,1,0.10081

2,1,4,18,2,0.021109

2,1,4,18,3,-0.012614

2,1,4,18,4,-0.026031

2,1,4,18,5,0.012834

2,1,4,18,6,-0.017676

2,1,4,18,7,-0.0044256

2,1,4,18,8,0.028245

2,1,4,19,1,0.085843

2,1,4,19,2,0.066709

2,1,4,19,3,-0.0039995

2,1,4,19,4,0.0023199

2,1,4,19,5,0.016658

2,1,4,19,6,-0.010553

2,1,4,19,7,-0.0072658

2,1,4,19,8,0.01757

2,1,4,20,1,0.038089

2,1,4,20,2,0.015221

2,1,4,20,3,0.019766

2,1,4,20,4,-0.016297

2,1,4,20,5,0.019039

2,1,4,20,6,-0.0016732

2,1,4,20,7,0.00045615

2,1,4,20,8,0.021968

2,1,5,5,1,0.22758

2,1,5,5,2,0.0034049

2,1,5,5,3,0.018666

2,1,5,5,4,-0.031309

2,1,5,5,5,0.0080922

2,1,5,5,6,-0.016633

2,1,5,5,7,-0.010436

2,1,5,5,8,0.0043691

2,1,5,6,1,0.11822

2,1,5,6,2,0.048613

2,1,5,6,3,-0.07397

2,1,5,6,4,-0.063841

2,1,5,6,5,0.0079071

2,1,5,6,6,-0.020516

2,1,5,6,7,-0.023645

2,1,5,6,8,-0.032164

2,1,5,7,1,0.13599

2,1,5,7,2,0.088298

2,1,5,7,3,-0.075717

2,1,5,7,4,-0.07402

2,1,5,7,5,0.0027286

2,1,5,7,6,-0.0051699

2,1,5,7,7,0.012311

2,1,5,7,8,-0.0058113

2,1,5,8,1,0.12028

2,1,5,8,2,0.049194

2,1,5,8,3,-0.067745

2,1,5,8,4,-0.062177

2,1,5,8,5,0.0182

2,1,5,8,6,-0.0081895

2,1,5,8,7,-0.012735

2,1,5,8,8,-0.013291

2,1,5,9,1,0.13907

2,1,5,9,2,0.069215

2,1,5,9,3,-0.0094899

2,1,5,9,4,-0.0088687

2,1,5,9,5,0.020284

2,1,5,9,6,0.0096447

2,1,5,9,7,-0.01095

2,1,5,9,8,0.025695

2,1,5,10,1,0.18735

2,1,5,10,2,0.096841

2,1,5,10,3,-0.069822

2,1,5,10,4,-0.0624

2,1,5,10,5,0.011444

2,1,5,10,6,-0.0038486

2,1,5,10,7,-0.032824

2,1,5,10,8,0.0054989

2,1,5,11,1,0.10064

2,1,5,11,2,0.063332

2,1,5,11,3,-0.085639

2,1,5,11,4,-0.054855

2,1,5,11,5,-0.0072745

2,1,5,11,6,-0.021337

2,1,5,11,7,-0.015452

2,1,5,11,8,-0.014805

2,1,5,12,1,0.072814

2,1,5,12,2,0.048246

2,1,5,12,3,-0.063612

2,1,5,12,4,-0.041438

2,1,5,12,5,0.022431

2,1,5,12,6,-0.013632

2,1,5,12,7,-0.01061

2,1,5,12,8,0.0080895

2,1,5,13,1,0.23336

2,1,5,13,2,0.060791

2,1,5,13,3,-0.02121

2,1,5,13,4,-0.0067593

2,1,5,13,5,0.0038567

2,1,5,13,6,0.015412

2,1,5,13,7,-0.0061491

2,1,5,13,8,0.034287

2,1,5,14,1,0.16386

2,1,5,14,2,0.033564

2,1,5,14,3,-0.059628

2,1,5,14,4,-0.029373

2,1,5,14,5,-0.026427

2,1,5,14,6,-0.01375

2,1,5,14,7,-0.010922

2,1,5,14,8,0.021822

2,1,5,15,1,0.07266

2,1,5,15,2,0.012908

2,1,5,15,3,-0.0027789

2,1,5,15,4,-0.025866

2,1,5,15,5,0.0066677

2,1,5,15,6,-0.0045409

2,1,5,15,7,-0.0095778

2,1,5,15,8,0.036639

2,1,5,16,1,0.038169

2,1,5,16,2,0.015795

2,1,5,16,3,0.027031

2,1,5,16,4,0.015275

2,1,5,16,5,0.014016

2,1,5,16,6,-0.019325

2,1,5,16,7,-0.0011746

2,1,5,16,8,-0.005551

2,1,5,17,1,0.25349

2,1,5,17,2,0.10479

2,1,5,17,3,-0.0019073

2,1,5,17,4,-0.053347

2,1,5,17,5,-0.013331

2,1,5,17,6,-0.031557

2,1,5,17,7,-0.013696

2,1,5,17,8,0.009071

2,1,5,18,1,0.078676

2,1,5,18,2,0.069353

2,1,5,18,3,-0.020912

2,1,5,18,4,-0.038716

2,1,5,18,5,0.0013034

2,1,5,18,6,-0.033077

2,1,5,18,7,0.00019147

2,1,5,18,8,0.021869

2,1,5,19,1,0.067216

2,1,5,19,2,0.053613

2,1,5,19,3,-0.017823

2,1,5,19,4,-0.042691

2,1,5,19,5,0.012554

2,1,5,19,6,-0.024108

2,1,5,19,7,-0.013532

2,1,5,19,8,0.012137

2,1,5,20,1,0.04312

2,1,5,20,2,0.05494

2,1,5,20,3,-0.025508

2,1,5,20,4,-0.026958

2,1,5,20,5,-0.03206

2,1,5,20,6,-0.014544

2,1,5,20,7,-0.028094

2,1,5,20,8,0.037022

2,1,6,6,1,0.089518

2,1,6,6,2,0.051682

2,1,6,6,3,-0.085677

2,1,6,6,4,-0.041714

2,1,6,6,5,-0.013126

2,1,6,6,6,-0.01148

2,1,6,6,7,-0.010233

2,1,6,6,8,-0.0224

2,1,6,7,1,0.078316

2,1,6,7,2,0.049209

2,1,6,7,3,-0.097315

2,1,6,7,4,-0.084082

2,1,6,7,5,0.026298

2,1,6,7,6,-0.0048391

2,1,6,7,7,-0.023877

2,1,6,7,8,0.0090056

2,1,6,8,1,0.095998

2,1,6,8,2,0.054137

2,1,6,8,3,-0.10532

2,1,6,8,4,-0.052765

2,1,6,8,5,0.007848

2,1,6,8,6,0.0057214

2,1,6,8,7,0.0061101

2,1,6,8,8,0.0035443

2,1,6,9,1,0.098139

2,1,6,9,2,0.058554

2,1,6,9,3,-0.076033

2,1,6,9,4,-0.01522

2,1,6,9,5,-0.015826

2,1,6,9,6,0.032924

2,1,6,9,7,-0.035743

2,1,6,9,8,0.010975

2,1,6,10,1,0.089844

2,1,6,10,2,0.0061191

2,1,6,10,3,-0.10748

2,1,6,10,4,-0.069293

2,1,6,10,5,0.048167

2,1,6,10,6,-0.018389

2,1,6,10,7,-0.00086422

2,1,6,10,8,-0.023215

2,1,6,11,1,0.083981

2,1,6,11,2,0.024192

2,1,6,11,3,-0.09143

2,1,6,11,4,-0.069261

2,1,6,11,5,0.012897

2,1,6,11,6,0.01018

2,1,6,11,7,-0.014785

2,1,6,11,8,-0.0067637

2,1,6,12,1,0.034589

2,1,6,12,2,0.071738

2,1,6,12,3,-0.028343

2,1,6,12,4,-0.093426

2,1,6,12,5,-0.011053

2,1,6,12,6,-0.0082994

2,1,6,12,7,-0.021082

2,1,6,12,8,-0.000342

2,1,6,13,1,0.12592

2,1,6,13,2,0.034416

2,1,6,13,3,-0.0032961

2,1,6,13,4,-0.026774

2,1,6,13,5,0.007981

2,1,6,13,6,0.038042

2,1,6,13,7,0.0092364

2,1,6,13,8,-0.0060986

2,1,6,14,1,0.11034

2,1,6,14,2,0.046975

2,1,6,14,3,-0.020719

2,1,6,14,4,-0.046559

2,1,6,14,5,0.019172

2,1,6,14,6,-0.00081131

2,1,6,14,7,-0.029836

2,1,6,14,8,0.0045129

2,1,6,15,1,0.045304

2,1,6,15,2,0.027773

2,1,6,15,3,-0.060839

2,1,6,15,4,-0.043917

2,1,6,15,5,0.032373

2,1,6,15,6,-0.022726

2,1,6,15,7,-0.043368

2,1,6,15,8,-0.031988

2,1,6,16,1,0.029604

2,1,6,16,2,0.041203

2,1,6,16,3,-0.022362

2,1,6,16,4,-0.025364

2,1,6,16,5,0.0081008

2,1,6,16,6,-0.014616

2,1,6,16,7,0.014028

2,1,6,16,8,-0.017328

2,1,6,17,1,0.12735

2,1,6,17,2,0.070887

2,1,6,17,3,-0.02772

2,1,6,17,4,-0.066531

2,1,6,17,5,0.030481

2,1,6,17,6,0.0092518

2,1,6,17,7,-0.01043

2,1,6,17,8,-0.0069288

2,1,6,18,1,0.073965

2,1,6,18,2,0.066711

2,1,6,18,3,-0.048391

2,1,6,18,4,-0.064193

2,1,6,18,5,0.0056941

2,1,6,18,6,0.0055055

2,1,6,18,7,-0.022168

2,1,6,18,8,-0.013336

2,1,6,19,1,0.070283

2,1,6,19,2,0.017927

2,1,6,19,3,-0.018618

2,1,6,19,4,-0.042905

2,1,6,19,5,0.0054623

2,1,6,19,6,0.0043771

2,1,6,19,7,0.0038538

2,1,6,19,8,0.0010644

2,1,6,20,1,0.031608

2,1,6,20,2,0.017728

2,1,6,20,3,-0.024844

2,1,6,20,4,-0.063544

2,1,6,20,5,0.011306

2,1,6,20,6,0.02861

2,1,6,20,7,-0.035166

2,1,6,20,8,0.033534

2,1,7,7,1,0.051581

2,1,7,7,2,0.022429

2,1,7,7,3,-0.030973

2,1,7,7,4,-0.068357

2,1,7,7,5,-0.02164

2,1,7,7,6,-0.0020404

2,1,7,7,7,-0.029149

2,1,7,7,8,-0.014881

2,1,7,8,1,0.087772

2,1,7,8,2,0.0068593

2,1,7,8,3,-0.087647

2,1,7,8,4,-0.077527

2,1,7,8,5,-0.015387

2,1,7,8,6,-0.015506

2,1,7,8,7,-0.014603

2,1,7,8,8,-0.01974

2,1,7,9,1,0.054547

2,1,7,9,2,0.042116

2,1,7,9,3,-0.11645

2,1,7,9,4,-0.054918

2,1,7,9,5,-0.020461

2,1,7,9,6,0.014263

2,1,7,9,7,-0.0034436

2,1,7,9,8,-0.00010556

2,1,7,10,1,0.10218

2,1,7,10,2,0.033657

2,1,7,10,3,-0.10006

2,1,7,10,4,-0.050388

2,1,7,10,5,-0.018437

2,1,7,10,6,0.029735

2,1,7,10,7,-0.044657

2,1,7,10,8,0.0026689

2,1,7,11,1,0.046607

2,1,7,11,2,0.012347

2,1,7,11,3,-0.037011

2,1,7,11,4,-0.059529

2,1,7,11,5,-0.015821

2,1,7,11,6,0.0044113

2,1,7,11,7,0.0034233

2,1,7,11,8,0.0062568

2,1,7,12,1,0.026469

2,1,7,12,2,0.0041317

2,1,7,12,3,-0.029039

2,1,7,12,4,-0.054297

2,1,7,12,5,0.021862

2,1,7,12,6,0.036903

2,1,7,12,7,-0.019496

2,1,7,12,8,0.0037391

2,1,7,13,1,0.11498

2,1,7,13,2,0.044399

2,1,7,13,3,-0.058228

2,1,7,13,4,-0.040968

2,1,7,13,5,0.0090432

2,1,7,13,6,0.029787

2,1,7,13,7,0.015831

2,1,7,13,8,0.012236

2,1,7,14,1,0.093862

2,1,7,14,2,0.010193

2,1,7,14,3,-0.042357

2,1,7,14,4,-0.098659

2,1,7,14,5,-0.011192

2,1,7,14,6,-0.0018945

2,1,7,14,7,-0.019579

2,1,7,14,8,0.0040425

2,1,7,15,1,0.035097

2,1,7,15,2,0.035024

2,1,7,15,3,-0.04194

2,1,7,15,4,-0.045141

2,1,7,15,5,-0.0029882

2,1,7,15,6,-0.0094441

2,1,7,15,7,0.0031964

2,1,7,15,8,0.010326

2,1,7,16,1,0.037497

2,1,7,16,2,-0.0042426

2,1,7,16,3,-0.047159

2,1,7,16,4,-0.022157

2,1,7,16,5,-0.0054574

2,1,7,16,6,0.043566

2,1,7,16,7,-0.013795

2,1,7,16,8,0.026628

2,1,7,17,1,0.13303

2,1,7,17,2,0.075665

2,1,7,17,3,-0.073421

2,1,7,17,4,-0.058543

2,1,7,17,5,0.0034938

2,1,7,17,6,-0.022601

2,1,7,17,7,-0.0014636

2,1,7,17,8,0.0071164

2,1,7,18,1,0.057037

2,1,7,18,2,0.033267

2,1,7,18,3,-0.043127

2,1,7,18,4,-0.053349

2,1,7,18,5,-0.0080284

2,1,7,18,6,0.0361

2,1,7,18,7,-0.0001251

2,1,7,18,8,0.056926

2,1,7,19,1,0.071616

2,1,7,19,2,0.031209

2,1,7,19,3,-0.064954

2,1,7,19,4,-0.060937

2,1,7,19,5,-0.0046268

2,1,7,19,6,-0.017429

2,1,7,19,7,-0.011693

2,1,7,19,8,0.012688

2,1,7,20,1,0.016205

2,1,7,20,2,-0.01497

2,1,7,20,3,-0.024975

2,1,7,20,4,-0.069301

2,1,7,20,5,0.029038

2,1,7,20,6,0.021544

2,1,7,20,7,0.015201

2,1,7,20,8,0.037793

2,1,8,8,1,0.15372

2,1,8,8,2,0.0827

2,1,8,8,3,-0.049766

2,1,8,8,4,-0.01332

2,1,8,8,5,0.0043372

2,1,8,8,6,-0.0052518

2,1,8,8,7,0.012796

2,1,8,8,8,-0.027405

2,1,8,9,1,0.13528

2,1,8,9,2,-0.013963

2,1,8,9,3,-0.070423

2,1,8,9,4,-0.0019405

2,1,8,9,5,0.017024

2,1,8,9,6,-0.013656

2,1,8,9,7,0.013798

2,1,8,9,8,-0.01179

2,1,8,10,1,0.1347

2,1,8,10,2,0.012239

2,1,8,10,3,-0.065713

2,1,8,10,4,-0.034256

2,1,8,10,5,-0.0026994

2,1,8,10,6,0.023563

2,1,8,10,7,-0.0098813

2,1,8,10,8,-0.002992

2,1,8,11,1,0.10437

2,1,8,11,2,0.023731

2,1,8,11,3,-0.12322

2,1,8,11,4,-0.068035

2,1,8,11,5,0.011375

2,1,8,11,6,-0.02452

2,1,8,11,7,0.0052294

2,1,8,11,8,-0.0071057

2,1,8,12,1,0.078702

2,1,8,12,2,0.050821

2,1,8,12,3,-0.062391

2,1,8,12,4,-0.055141

2,1,8,12,5,0.011454

2,1,8,12,6,0.011257

2,1,8,12,7,-0.0023163

2,1,8,12,8,-0.012337

2,1,8,13,1,0.15328

2,1,8,13,2,0.022195

2,1,8,13,3,-0.066376

2,1,8,13,4,-0.047521

2,1,8,13,5,-0.0035718

2,1,8,13,6,-0.00075527

2,1,8,13,7,-0.024095

2,1,8,13,8,-0.015269

2,1,8,14,1,0.1202

2,1,8,14,2,0.040449

2,1,8,14,3,-0.027959

2,1,8,14,4,-0.026362

2,1,8,14,5,0.042818

2,1,8,14,6,0.030513

2,1,8,14,7,-0.00046007

2,1,8,14,8,0.03334

2,1,8,15,1,0.046029

2,1,8,15,2,0.011827

2,1,8,15,3,-0.082344

2,1,8,15,4,-0.057383

2,1,8,15,5,-0.0094228

2,1,8,15,6,-0.0080721

2,1,8,15,7,-0.025074

2,1,8,15,8,0.019418

2,1,8,16,1,0.055823

2,1,8,16,2,0.048437

2,1,8,16,3,-0.024063

2,1,8,16,4,-0.0059465

2,1,8,16,5,0.014901

2,1,8,16,6,-0.017876

2,1,8,16,7,-0.0058036

2,1,8,16,8,-0.0010259

2,1,8,17,1,0.17041

2,1,8,17,2,0.092748

2,1,8,17,3,-0.046572

2,1,8,17,4,-0.03831

2,1,8,17,5,0.020436

2,1,8,17,6,0.002646

2,1,8,17,7,-0.014037

2,1,8,17,8,0.01377

2,1,8,18,1,0.11032

2,1,8,18,2,0.048602

2,1,8,18,3,-0.049445

2,1,8,18,4,-0.047194

2,1,8,18,5,0.044065

2,1,8,18,6,-0.032728

2,1,8,18,7,0.0080417

2,1,8,18,8,-0.020712

2,1,8,19,1,0.059092

2,1,8,19,2,0.011507

2,1,8,19,3,-0.075412

2,1,8,19,4,-0.018869

2,1,8,19,5,0.016136

2,1,8,19,6,0.02666

2,1,8,19,7,0.0016523

2,1,8,19,8,0.011716

2,1,8,20,1,0.035129

2,1,8,20,2,0.054275

2,1,8,20,3,-0.021175

2,1,8,20,4,-0.038102

2,1,8,20,5,0.039203

2,1,8,20,6,-0.0073757

2,1,8,20,7,0.01646

2,1,8,20,8,-0.018447

2,1,9,9,1,0.12315

2,1,9,9,2,-0.01319

2,1,9,9,3,0.0032141

2,1,9,9,4,-0.0084831

2,1,9,9,5,-0.0071754

2,1,9,9,6,-0.012609

2,1,9,9,7,0.0047401

2,1,9,9,8,-0.011082

2,1,9,10,1,0.11357

2,1,9,10,2,0.024974

2,1,9,10,3,-0.033572

2,1,9,10,4,-0.026519

2,1,9,10,5,0.0057765

2,1,9,10,6,0.0083154

2,1,9,10,7,-0.00064833

2,1,9,10,8,-0.00076575

2,1,9,11,1,0.11705

2,1,9,11,2,0.020785

2,1,9,11,3,-0.072463

2,1,9,11,4,-0.048856

2,1,9,11,5,-0.02951

2,1,9,11,6,0.01029

2,1,9,11,7,-0.035534

2,1,9,11,8,0.011553

2,1,9,12,1,0.059631

2,1,9,12,2,0.049184

2,1,9,12,3,-0.076186

2,1,9,12,4,0.00055841

2,1,9,12,5,-0.00039237

2,1,9,12,6,-0.014542

2,1,9,12,7,-0.0037611

2,1,9,12,8,-0.011316

2,1,9,13,1,0.16207

2,1,9,13,2,0.063845

2,1,9,13,3,3.6342e-06

2,1,9,13,4,0.0032192

2,1,9,13,5,0.0088448

2,1,9,13,6,-0.0049422

2,1,9,13,7,-0.031115

2,1,9,13,8,0.017835

2,1,9,14,1,0.12744

2,1,9,14,2,-0.028926

2,1,9,14,3,-0.027449

2,1,9,14,4,-0.041879

2,1,9,14,5,-0.0010982

2,1,9,14,6,-0.01899

2,1,9,14,7,-0.028468

2,1,9,14,8,0.032722

2,1,9,15,1,0.042121

2,1,9,15,2,0.054257

2,1,9,15,3,-0.0314

2,1,9,15,4,-0.016182

2,1,9,15,5,0.0071043

2,1,9,15,6,-0.022289

2,1,9,15,7,-0.011345

2,1,9,15,8,0.01378

2,1,9,16,1,0.066221

2,1,9,16,2,-0.02152

2,1,9,16,3,-0.01038

2,1,9,16,4,0.019877

2,1,9,16,5,-0.003841

2,1,9,16,6,0.017794

2,1,9,16,7,-0.013317

2,1,9,16,8,0.044306

2,1,9,17,1,0.20713

2,1,9,17,2,0.061701

2,1,9,17,3,-0.032742

2,1,9,17,4,-0.037009

2,1,9,17,5,-0.010494

2,1,9,17,6,-0.032545

2,1,9,17,7,-0.021961

2,1,9,17,8,0.017363

2,1,9,18,1,0.071789

2,1,9,18,2,0.040741

2,1,9,18,3,-0.01212

2,1,9,18,4,-0.027329

2,1,9,18,5,0.024057

2,1,9,18,6,-0.016879

2,1,9,18,7,0.01403

2,1,9,18,8,0.024801

2,1,9,19,1,0.055683

2,1,9,19,2,0.02317

2,1,9,19,3,-0.024901

2,1,9,19,4,0.0020105

2,1,9,19,5,0.032495

2,1,9,19,6,-0.011016

2,1,9,19,7,0.019715

2,1,9,19,8,0.014458

2,1,9,20,1,0.02574

2,1,9,20,2,0.020671

2,1,9,20,3,-0.010782

2,1,9,20,4,-0.023013

2,1,9,20,5,-0.0023393

2,1,9,20,6,-0.036715

2,1,9,20,7,-0.0056007

2,1,9,20,8,0.014516

2,1,10,10,1,0.14337

2,1,10,10,2,-0.060571

2,1,10,10,3,-0.023824

2,1,10,10,4,-0.011233

2,1,10,10,5,0.001658

2,1,10,10,6,-7.0818e-06

2,1,10,10,7,-0.0076882

2,1,10,10,8,-0.0066563

2,1,10,11,1,0.11123

2,1,10,11,2,0.069163

2,1,10,11,3,0.0077834

2,1,10,11,4,-0.080734

2,1,10,11,5,0.031794

2,1,10,11,6,-0.014745

2,1,10,11,7,0.0088632

2,1,10,11,8,0.010557

2,1,10,12,1,0.044893

2,1,10,12,2,-0.0021124

2,1,10,12,3,-0.032753

2,1,10,12,4,-0.072448

2,1,10,12,5,-0.010109

2,1,10,12,6,-0.0072628

2,1,10,12,7,-0.0096428

2,1,10,12,8,-0.017235

2,1,10,13,1,0.15604

2,1,10,13,2,0.034323

2,1,10,13,3,-0.070408

2,1,10,13,4,-0.045512

2,1,10,13,5,-0.0055576

2,1,10,13,6,-0.0086553

2,1,10,13,7,-0.012839

2,1,10,13,8,-0.013222

2,1,10,14,1,0.14668

2,1,10,14,2,0.066963

2,1,10,14,3,-0.064123

2,1,10,14,4,-0.036587

2,1,10,14,5,-0.0019469

2,1,10,14,6,0.0033269

2,1,10,14,7,0.014414

2,1,10,14,8,-0.0058774

2,1,10,15,1,0.058704

2,1,10,15,2,0.012417

2,1,10,15,3,-0.046576

2,1,10,15,4,-0.079182

2,1,10,15,5,0.0071857

2,1,10,15,6,-0.0051137

2,1,10,15,7,-0.030673

2,1,10,15,8,0.019182

2,1,10,16,1,0.052114

2,1,10,16,2,0.034505

2,1,10,16,3,-0.051749

2,1,10,16,4,-0.017507

2,1,10,16,5,0.016504

2,1,10,16,6,0.013916

2,1,10,16,7,0.037131

2,1,10,16,8,0.020314

2,1,10,17,1,0.20555

2,1,10,17,2,0.1173

2,1,10,17,3,-0.072068

2,1,10,17,4,-0.038193

2,1,10,17,5,0.0060942

2,1,10,17,6,0.020595

2,1,10,17,7,-0.0066065

2,1,10,17,8,0.02247

2,1,10,18,1,0.084234

2,1,10,18,2,0.03701

2,1,10,18,3,-0.057332

2,1,10,18,4,-0.084792

2,1,10,18,5,0.0059105

2,1,10,18,6,-0.0058118

2,1,10,18,7,-0.014982

2,1,10,18,8,0.0017712

2,1,10,19,1,0.10914

2,1,10,19,2,0.011621

2,1,10,19,3,-0.065686

2,1,10,19,4,-0.04104

2,1,10,19,5,-0.0097782

2,1,10,19,6,0.0014104

2,1,10,19,7,0.0071864

2,1,10,19,8,-0.012771

2,1,10,20,1,0.054566

2,1,10,20,2,0.0045158

2,1,10,20,3,-0.03562

2,1,10,20,4,-0.079908

2,1,10,20,5,0.0098467

2,1,10,20,6,-0.025814

2,1,10,20,7,0.0039992

2,1,10,20,8,0.027962

2,1,11,11,1,0.11924

2,1,11,11,2,0.026852

2,1,11,11,3,-0.042202

2,1,11,11,4,-0.03232

2,1,11,11,5,-0.003949

2,1,11,11,6,0.003152

2,1,11,11,7,-0.012731

2,1,11,11,8,-0.01257

2,1,11,12,1,0.044868

2,1,11,12,2,-0.012975

2,1,11,12,3,-0.067639

2,1,11,12,4,-0.082268

2,1,11,12,5,-0.00804

2,1,11,12,6,-0.01117

2,1,11,12,7,-0.033129

2,1,11,12,8,-0.035833

2,1,11,13,1,0.12745

2,1,11,13,2,0.019749

2,1,11,13,3,-0.053542

2,1,11,13,4,-0.067167

2,1,11,13,5,-0.0040204

2,1,11,13,6,-0.013989

2,1,11,13,7,-0.0064206

2,1,11,13,8,-0.0028788

2,1,11,14,1,0.11166

2,1,11,14,2,0.036569

2,1,11,14,3,-0.073044

2,1,11,14,4,-0.057006

2,1,11,14,5,-0.0081305

2,1,11,14,6,0.028916

2,1,11,14,7,-0.023952

2,1,11,14,8,0.02698

2,1,11,15,1,0.024437

2,1,11,15,2,0.014141

2,1,11,15,3,-0.055816

2,1,11,15,4,-0.0414

2,1,11,15,5,0.025113

2,1,11,15,6,-0.00037152

2,1,11,15,7,0.015487

2,1,11,15,8,0.010094

2,1,11,16,1,0.057514

2,1,11,16,2,-0.0092546

2,1,11,16,3,-0.021295

2,1,11,16,4,-0.045857

2,1,11,16,5,-0.0093311

2,1,11,16,6,-0.015244

2,1,11,16,7,0.0051724

2,1,11,16,8,0.013552

2,1,11,17,1,0.17564

2,1,11,17,2,0.071386

2,1,11,17,3,-0.084608

2,1,11,17,4,-0.073746

2,1,11,17,5,-0.018817

2,1,11,17,6,-0.0059499

2,1,11,17,7,-0.024124

2,1,11,17,8,0.0033411

2,1,11,18,1,0.069056

2,1,11,18,2,0.0101

2,1,11,18,3,-0.10743

2,1,11,18,4,-0.049816

2,1,11,18,5,-0.039408

2,1,11,18,6,0.0053563

2,1,11,18,7,-0.010188

2,1,11,18,8,-0.0078753

2,1,11,19,1,0.068895

2,1,11,19,2,0.035546

2,1,11,19,3,-0.094921

2,1,11,19,4,-0.028226

2,1,11,19,5,-0.0033494

2,1,11,19,6,0.0093035

2,1,11,19,7,-0.015572

2,1,11,19,8,-0.016092

2,1,11,20,1,0.030728

2,1,11,20,2,-0.0004757

2,1,11,20,3,-0.048515

2,1,11,20,4,-0.071678

2,1,11,20,5,-0.010544

2,1,11,20,6,0.028621

2,1,11,20,7,0.0073947

2,1,11,20,8,-0.006765

2,1,12,12,1,0.030886

2,1,12,12,2,0.020587

2,1,12,12,3,-0.014422

2,1,12,12,4,-0.048262

2,1,12,12,5,-0.014773

2,1,12,12,6,0.0097365

2,1,12,12,7,-0.0074297

2,1,12,12,8,-0.0060581

2,1,12,13,1,0.033991

2,1,12,13,2,-0.0012264

2,1,12,13,3,-0.050794

2,1,12,13,4,-0.054258

2,1,12,13,5,-0.0050691

2,1,12,13,6,0.010091

2,1,12,13,7,0.01874

2,1,12,13,8,0.020715

2,1,12,14,1,0.05465

2,1,12,14,2,0.044028

2,1,12,14,3,-0.071597

2,1,12,14,4,-0.038234

2,1,12,14,5,0.003361

2,1,12,14,6,0.034322

2,1,12,14,7,-0.014793

2,1,12,14,8,0.01327

2,1,12,15,1,0.031273

2,1,12,15,2,0.0064254

2,1,12,15,3,-0.067703

2,1,12,15,4,-0.065536

2,1,12,15,5,0.0084291

2,1,12,15,6,0.017259

2,1,12,15,7,-0.017426

2,1,12,15,8,0.010568

2,1,12,16,1,0.029784

2,1,12,16,2,0.025672

2,1,12,16,3,-0.052546

2,1,12,16,4,-0.09166

2,1,12,16,5,0.032511

2,1,12,16,6,-0.025624

2,1,12,16,7,-0.018423

2,1,12,16,8,0.043524

2,1,12,17,1,0.072195

2,1,12,17,2,0.070931

2,1,12,17,3,-0.071335

2,1,12,17,4,-0.080013

2,1,12,17,5,-0.025667

2,1,12,17,6,-0.014807

2,1,12,17,7,0.017767

2,1,12,17,8,0.010815

2,1,12,18,1,0.041529

2,1,12,18,2,0.021343

2,1,12,18,3,-0.062863

2,1,12,18,4,-0.063896

2,1,12,18,5,0.029221

2,1,12,18,6,0.0011897

2,1,12,18,7,0.0073038

2,1,12,18,8,0.026745

2,1,12,19,1,0.039609

2,1,12,19,2,0.010448

2,1,12,19,3,-0.078649

2,1,12,19,4,-0.032463

2,1,12,19,5,0.0078082

2,1,12,19,6,0.0012196

2,1,12,19,7,-0.024914

2,1,12,19,8,0.020419

2,1,12,20,1,0.012411

2,1,12,20,2,0.028123

2,1,12,20,3,-0.024632

2,1,12,20,4,-0.019891

2,1,12,20,5,0.012796

2,1,12,20,6,-0.033782

2,1,12,20,7,0.0064551

2,1,12,20,8,-0.038792

2,1,13,13,1,0.22933

2,1,13,13,2,0.0064821

2,1,13,13,3,-0.017842

2,1,13,13,4,-0.0015325

2,1,13,13,5,-0.0062993

2,1,13,13,6,-0.0024893

2,1,13,13,7,-0.0011336

2,1,13,13,8,0.0067213

2,1,13,14,1,0.21713

2,1,13,14,2,0.0047019

2,1,13,14,3,-0.064095

2,1,13,14,4,-0.018042

2,1,13,14,5,-0.025426

2,1,13,14,6,-0.0092622

2,1,13,14,7,-0.010231

2,1,13,14,8,0.030016

2,1,13,15,1,0.051948

2,1,13,15,2,0.027924

2,1,13,15,3,-0.051405

2,1,13,15,4,-0.030217

2,1,13,15,5,-0.0032067

2,1,13,15,6,-0.018367

2,1,13,15,7,-0.022313

2,1,13,15,8,0.02832

2,1,13,16,1,0.040239

2,1,13,16,2,-0.0033663

2,1,13,16,3,-0.026313

2,1,13,16,4,-0.01807

2,1,13,16,5,-0.0046333

2,1,13,16,6,0.0028277

2,1,13,16,7,-0.004863

2,1,13,16,8,0.02859

2,1,13,17,1,0.25557

2,1,13,17,2,0.059786

2,1,13,17,3,-0.039999

2,1,13,17,4,-0.042937

2,1,13,17,5,-0.031685

2,1,13,17,6,-0.020989

2,1,13,17,7,-0.020603

2,1,13,17,8,0.013455

2,1,13,18,1,0.12893

2,1,13,18,2,0.018724

2,1,13,18,3,-0.074254

2,1,13,18,4,-0.021249

2,1,13,18,5,-0.020581

2,1,13,18,6,-0.032819

2,1,13,18,7,-0.0042567

2,1,13,18,8,0.023941

2,1,13,19,1,0.067926

2,1,13,19,2,0.022519

2,1,13,19,3,-0.068003

2,1,13,19,4,-0.0051652

2,1,13,19,5,-0.0071826

2,1,13,19,6,-0.019802

2,1,13,19,7,-0.003811

2,1,13,19,8,0.016633

2,1,13,20,1,0.039661

2,1,13,20,2,-0.001671

2,1,13,20,3,-0.018268

2,1,13,20,4,-0.021938

2,1,13,20,5,0.0089443

2,1,13,20,6,0.0059591

2,1,13,20,7,0.028653

2,1,13,20,8,0.032147

2,1,14,14,1,0.14929

2,1,14,14,2,0.0078468

2,1,14,14,3,-0.039883

2,1,14,14,4,-0.00013272

2,1,14,14,5,-0.017834

2,1,14,14,6,-0.0028303

2,1,14,14,7,0.0098892

2,1,14,14,8,0.016141

2,1,14,15,1,0.052152

2,1,14,15,2,0.0020653

2,1,14,15,3,-0.056107

2,1,14,15,4,-0.062824

2,1,14,15,5,-0.034285

2,1,14,15,6,-0.021382

2,1,14,15,7,0.028767

2,1,14,15,8,0.033378

2,1,14,16,1,0.057912

2,1,14,16,2,0.012739

2,1,14,16,3,-0.024171

2,1,14,16,4,-0.018852

2,1,14,16,5,-0.020311

2,1,14,16,6,-0.022487

2,1,14,16,7,-0.00077079

2,1,14,16,8,0.0017254

2,1,14,17,1,0.19056

2,1,14,17,2,0.050692

2,1,14,17,3,-0.061684

2,1,14,17,4,-0.04767

2,1,14,17,5,-0.018871

2,1,14,17,6,-0.039919

2,1,14,17,7,-0.002371

2,1,14,17,8,0.0093339

2,1,14,18,1,0.096048

2,1,14,18,2,0.058159

2,1,14,18,3,-0.087097

2,1,14,18,4,-0.01511

2,1,14,18,5,-0.026656

2,1,14,18,6,-0.0083966

2,1,14,18,7,-0.0045349

2,1,14,18,8,0.021883

2,1,14,19,1,0.057578

2,1,14,19,2,-0.036922

2,1,14,19,3,-0.074085

2,1,14,19,4,-0.033402

2,1,14,19,5,-0.017512

2,1,14,19,6,-0.012709

2,1,14,19,7,-0.012808

2,1,14,19,8,0.036413

2,1,14,20,1,0.038107

2,1,14,20,2,0.019298

2,1,14,20,3,-0.019659

2,1,14,20,4,-0.030015

2,1,14,20,5,0.0053846

2,1,14,20,6,-0.020459

2,1,14,20,7,-0.011862

2,1,14,20,8,0.030652

2,1,15,15,1,0.034084

2,1,15,15,2,0.017757

2,1,15,15,3,-0.0042474

2,1,15,15,4,-0.0078356

2,1,15,15,5,0.0025212

2,1,15,15,6,-0.0083769

2,1,15,15,7,-0.020314

2,1,15,15,8,0.018153

2,1,15,16,1,0.012784

2,1,15,16,2,0.0064357

2,1,15,16,3,-0.024166

2,1,15,16,4,0.012319

2,1,15,16,5,-0.00036902

2,1,15,16,6,-0.021652

2,1,15,16,7,0.0035148

2,1,15,16,8,0.018625

2,1,15,17,1,0.074001

2,1,15,17,2,0.046187

2,1,15,17,3,-0.069783

2,1,15,17,4,-0.064487

2,1,15,17,5,-0.02609

2,1,15,17,6,-0.011754

2,1,15,17,7,-0.035793

2,1,15,17,8,0.034118

2,1,15,18,1,0.025307

2,1,15,18,2,0.022889

2,1,15,18,3,-0.010719

2,1,15,18,4,-0.064801

2,1,15,18,5,-0.016547

2,1,15,18,6,-0.0012226

2,1,15,18,7,-0.0047861

2,1,15,18,8,0.036822

2,1,15,19,1,0.021258

2,1,15,19,2,0.011727

2,1,15,19,3,-0.023311

2,1,15,19,4,0.0031701

2,1,15,19,5,0.0081528

2,1,15,19,6,-0.044741

2,1,15,19,7,-0.0043522

2,1,15,19,8,0.013724

2,1,15,20,1,0.0091747

2,1,15,20,2,-0.0036258

2,1,15,20,3,-0.014295

2,1,15,20,4,-0.0324

2,1,15,20,5,-0.012472

2,1,15,20,6,-0.038724

2,1,15,20,7,0.0023634

2,1,15,20,8,0.044132

2,1,16,16,1,2.6093e-11

2,1,16,16,2,-0.079308

2,1,16,16,3,0.022133

2,1,16,16,4,-0.01918

2,1,16,16,5,0.031562

2,1,16,16,6,-0.015258

2,1,16,16,7,0.019681

2,1,16,16,8,0.011231

2,1,16,17,1,0.064138

2,1,16,17,2,0.0065324

2,1,16,17,3,-0.0048706

2,1,16,17,4,-0.019505

2,1,16,17,5,0.012965

2,1,16,17,6,-0.0050404

2,1,16,17,7,0.025024

2,1,16,17,8,0.018196

2,1,16,18,1,0.035577

2,1,16,18,2,0.016788

2,1,16,18,3,-0.016953

2,1,16,18,4,0.0080706

2,1,16,18,5,0.0062867

2,1,16,18,6,-0.01381

2,1,16,18,7,-0.0043967

2,1,16,18,8,0.022298

2,1,16,19,1,0.017718

2,1,16,19,2,0.024827

2,1,16,19,3,-0.027892

2,1,16,19,4,0.0084083

2,1,16,19,5,0.011951

2,1,16,19,6,-0.031564

2,1,16,19,7,-0.0082388

2,1,16,19,8,-0.0026891

2,1,16,20,1,0.016582

2,1,16,20,2,0.022622

2,1,16,20,3,-0.070711

2,1,16,20,4,0.0060113

2,1,16,20,5,0.012951

2,1,16,20,6,-0.021432

2,1,16,20,7,0.0089378

2,1,16,20,8,0.024883

2,1,17,17,1,0.25752

2,1,17,17,2,0.0448

2,1,17,17,3,-0.0039242

2,1,17,17,4,-0.024587

2,1,17,17,5,-0.0116

2,1,17,17,6,-0.018895

2,1,17,17,7,-0.0012778

2,1,17,17,8,0.0064802

2,1,17,18,1,0.13201

2,1,17,18,2,0.057926

2,1,17,18,3,-0.050005

2,1,17,18,4,-0.026312

2,1,17,18,5,-0.032618

2,1,17,18,6,-0.027271

2,1,17,18,7,-0.009909

2,1,17,18,8,0.029555

2,1,17,19,1,0.079645

2,1,17,19,2,0.018703

2,1,17,19,3,-0.040797

2,1,17,19,4,-0.011253

2,1,17,19,5,0.0011302

2,1,17,19,6,-0.021597

2,1,17,19,7,0.024207

2,1,17,19,8,0.032993

2,1,17,20,1,0.044084

2,1,17,20,2,0.020174

2,1,17,20,3,-0.025271

2,1,17,20,4,-0.018171

2,1,17,20,5,-0.038644

2,1,17,20,6,-0.027122

2,1,17,20,7,-0.0046524

2,1,17,20,8,0.016369

2,1,18,18,1,0.069417

2,1,18,18,2,-0.0030374

2,1,18,18,3,-0.030437

2,1,18,18,4,-0.0046606

2,1,18,18,5,-0.011107

2,1,18,18,6,-0.00038016

2,1,18,18,7,0.00030016

2,1,18,18,8,0.030263

2,1,18,19,1,0.063273

2,1,18,19,2,0.052332

2,1,18,19,3,-0.046188

2,1,18,19,4,-0.030115

2,1,18,19,5,-0.004295

2,1,18,19,6,-0.027648

2,1,18,19,7,-0.0016263

2,1,18,19,8,0.019814

2,1,18,20,1,0.019966

2,1,18,20,2,0.0094019

2,1,18,20,3,-0.069239

2,1,18,20,4,-0.028181

2,1,18,20,5,-0.0029449

2,1,18,20,6,-0.01996

2,1,18,20,7,0.0036429

2,1,18,20,8,0.041741

2,1,19,19,1,0.06

2,1,19,19,2,0.0047341

2,1,19,19,3,-0.021865

2,1,19,19,4,-0.018954

2,1,19,19,5,-0.016718

2,1,19,19,6,-0.002196

2,1,19,19,7,0.0057679

2,1,19,19,8,0.012176

2,1,19,20,1,0.021394

2,1,19,20,2,-0.0028016

2,1,19,20,3,-0.045928

2,1,19,20,4,-0.013234

2,1,19,20,5,-0.0075898

2,1,19,20,6,-0.004923

2,1,19,20,7,-0.0056732

2,1,19,20,8,0.022135

2,1,20,20,1,0.010973

2,1,20,20,2,0.0070579

2,1,20,20,3,-0.00048774

2,1,20,20,4,-0.020172

2,1,20,20,5,-0.0059259

2,1,20,20,6,0.010503

2,1,20,20,7,-0.01301

2,1,20,20,8,0.011583

2,2,1,1,1,0.094684

2,2,1,1,2,-0.040935

2,2,1,1,3,-0.027038

2,2,1,1,4,-0.035281

2,2,1,1,5,-0.005498

2,2,1,1,6,-0.012793

2,2,1,1,7,-0.0062927

2,2,1,1,8,-0.023645

2,2,1,2,1,0.010136

2,2,1,2,2,-0.007746

2,2,1,2,3,-0.041459

2,2,1,2,4,-0.013881

2,2,1,2,5,-0.01853

2,2,1,2,6,-0.00077084

2,2,1,2,7,-0.0232

2,2,1,2,8,-0.031642

2,2,1,3,1,0.081473

2,2,1,3,2,-0.0022913

2,2,1,3,3,-0.11137

2,2,1,3,4,-0.050656

2,2,1,3,5,-0.019351

2,2,1,3,6,-0.0082251

2,2,1,3,7,-0.0095531

2,2,1,3,8,-0.030331

2,2,1,4,1,0.075155

2,2,1,4,2,0.013687

2,2,1,4,3,-0.064347

2,2,1,4,4,-0.023371

2,2,1,4,5,-0.0093787

2,2,1,4,6,0.0022799

2,2,1,4,7,-0.013022

2,2,1,4,8,-0.022787

2,2,1,5,1,0.12966

2,2,1,5,2,-0.0076002

2,2,1,5,3,-0.05266

2,2,1,5,4,-0.043489

2,2,1,5,5,-0.00081964

2,2,1,5,6,-0.011516

2,2,1,5,7,-0.014838

2,2,1,5,8,-0.023385

2,2,1,6,1,0.061875

2,2,1,6,2,-0.011223

2,2,1,6,3,-0.07478

2,2,1,6,4,-0.054235

2,2,1,6,5,-0.0073054

2,2,1,6,6,-0.010124

2,2,1,6,7,-0.022884

2,2,1,6,8,-0.034812

2,2,1,7,1,0.042151

2,2,1,7,2,-0.015544

2,2,1,7,3,-0.11295

2,2,1,7,4,-0.086842

2,2,1,7,5,-0.031913

2,2,1,7,6,-0.026517

2,2,1,7,7,-0.025711

2,2,1,7,8,-0.028007

2,2,1,8,1,0.080085

2,2,1,8,2,-0.015356

2,2,1,8,3,-0.086248

2,2,1,8,4,-0.02754

2,2,1,8,5,-0.016548

2,2,1,8,6,-0.017573

2,2,1,8,7,-0.013977

2,2,1,8,8,-0.03461

2,2,1,9,1,0.073802

2,2,1,9,2,-0.0029072

2,2,1,9,3,-0.065529

2,2,1,9,4,-0.030244

2,2,1,9,5,-0.012149

2,2,1,9,6,-0.00069

2,2,1,9,7,-0.010975

2,2,1,9,8,-0.019495

2,2,1,10,1,0.071407

2,2,1,10,2,-0.028436

2,2,1,10,3,-0.084395

2,2,1,10,4,-0.049604

2,2,1,10,5,-0.016997

2,2,1,10,6,-0.010167

2,2,1,10,7,-0.0042633

2,2,1,10,8,-0.023203

2,2,1,11,1,0.081083

2,2,1,11,2,0.0038126

2,2,1,11,3,-0.073912

2,2,1,11,4,-0.080082

2,2,1,11,5,-0.01553

2,2,1,11,6,-0.016363

2,2,1,11,7,-0.021351

2,2,1,11,8,-0.034585

2,2,1,12,1,0.036529

2,2,1,12,2,-0.013737

2,2,1,12,3,-0.070036

2,2,1,12,4,-0.057645

2,2,1,12,5,0.0036134

2,2,1,12,6,0.010202

2,2,1,12,7,0.008441

2,2,1,12,8,-0.01673

2,2,1,13,1,0.12754

2,2,1,13,2,0.027777

2,2,1,13,3,-0.036044

2,2,1,13,4,0.0050766

2,2,1,13,5,0.0073443

2,2,1,13,6,0.033079

2,2,1,13,7,0.00086874

2,2,1,13,8,-0.0032718

2,2,1,14,1,0.11655

2,2,1,14,2,0.0055339

2,2,1,14,3,-0.058503

2,2,1,14,4,-0.012316

2,2,1,14,5,-0.0023076

2,2,1,14,6,0.013046

2,2,1,14,7,-0.012132

2,2,1,14,8,-0.017474

2,2,1,15,1,0.062097

2,2,1,15,2,0.020324

2,2,1,15,3,-0.038704

2,2,1,15,4,0.0010782

2,2,1,15,5,-0.014752

2,2,1,15,6,0.015969

2,2,1,15,7,0.011636

2,2,1,15,8,-0.018769

2,2,1,16,1,0.043179

2,2,1,16,2,0.041428

2,2,1,16,3,-0.073643

2,2,1,16,4,-0.016093

2,2,1,16,5,-0.013844

2,2,1,16,6,-0.0027772

2,2,1,16,7,-0.016766

2,2,1,16,8,-0.054097

2,2,1,17,1,0.15878

2,2,1,17,2,0.062107

2,2,1,17,3,-0.047816

2,2,1,17,4,-0.02159

2,2,1,17,5,-0.014247

2,2,1,17,6,0.0080333

2,2,1,17,7,-0.014268

2,2,1,17,8,-0.021788

2,2,1,18,1,0.071173

2,2,1,18,2,0.048167

2,2,1,18,3,-0.074562

2,2,1,18,4,-0.029887

2,2,1,18,5,-0.005503

2,2,1,18,6,0.0062518

2,2,1,18,7,-0.011102

2,2,1,18,8,-0.018092

2,2,1,19,1,0.063138

2,2,1,19,2,0.045484

2,2,1,19,3,-0.061114

2,2,1,19,4,-0.03376

2,2,1,19,5,-0.014807

2,2,1,19,6,0.0032479

2,2,1,19,7,-0.0058722

2,2,1,19,8,-0.012085

2,2,1,20,1,0.020451

2,2,1,20,2,-0.0088621

2,2,1,20,3,-0.057107

2,2,1,20,4,-0.013985

2,2,1,20,5,-0.016509

2,2,1,20,6,0.0014456

2,2,1,20,7,-0.016144

2,2,1,20,8,-0.0038267

2,2,2,2,1,2.4103e-10

2,2,2,2,2,-0.020898

2,2,2,2,3,-0.0081041

2,2,2,2,4,-0.018118

2,2,2,2,5,-0.011337

2,2,2,2,6,-0.026254

2,2,2,2,7,-0.012742

2,2,2,2,8,-0.0063053

2,2,2,3,1,0.0044692

2,2,2,3,2,0.0251

2,2,2,3,3,-0.069509

2,2,2,3,4,-0.047455

2,2,2,3,5,-0.024252

2,2,2,3,6,-0.031261

2,2,2,3,7,-0.020865

2,2,2,3,8,-0.023155

2,2,2,4,1,3.0619e-08

2,2,2,4,2,0.027632

2,2,2,4,3,-0.039673

2,2,2,4,4,-0.038342

2,2,2,4,5,-0.023505

2,2,2,4,6,-0.026635

2,2,2,4,7,-0.028515

2,2,2,4,8,-0.0020514

2,2,2,5,1,2.8573e-11

2,2,2,5,2,0.014972

2,2,2,5,3,-0.031701

2,2,2,5,4,-0.024827

2,2,2,5,5,-0.015241

2,2,2,5,6,-0.013768

2,2,2,5,7,-0.01148

2,2,2,5,8,0.005992

2,2,2,6,1,0.026271

2,2,2,6,2,-0.020538

2,2,2,6,3,-0.059684

2,2,2,6,4,-0.006659

2,2,2,6,5,-0.026196

2,2,2,6,6,-0.0033615

2,2,2,6,7,-0.024673

2,2,2,6,8,-0.014129

2,2,2,7,1,0.0079021

2,2,2,7,2,0.020032

2,2,2,7,3,-0.042469

2,2,2,7,4,-0.067793

2,2,2,7,5,-0.03354

2,2,2,7,6,-0.05075

2,2,2,7,7,-0.03193

2,2,2,7,8,-0.021209

2,2,2,8,1,0.027286

2,2,2,8,2,-0.0056827

2,2,2,8,3,-0.053455

2,2,2,8,4,-0.033015

2,2,2,8,5,-0.023418

2,2,2,8,6,-0.022838

2,2,2,8,7,-0.025312

2,2,2,8,8,0.011823

2,2,2,9,1,0.0064609

2,2,2,9,2,0.011969

2,2,2,9,3,-0.047128

2,2,2,9,4,-0.014904

2,2,2,9,5,-0.031808

2,2,2,9,6,-0.013927

2,2,2,9,7,-0.026636

2,2,2,9,8,-0.007058

2,2,2,10,1,3.609e-11

2,2,2,10,2,0.012872

2,2,2,10,3,-0.035787

2,2,2,10,4,-0.046761

2,2,2,10,5,-0.015672

2,2,2,10,6,-0.032809

2,2,2,10,7,-0.015559

2,2,2,10,8,-0.021596

2,2,2,11,1,0.0071298

2,2,2,11,2,-0.027126

2,2,2,11,3,-0.054373

2,2,2,11,4,-0.061112

2,2,2,11,5,-0.034003

2,2,2,11,6,-0.033049

2,2,2,11,7,-0.020281

2,2,2,11,8,-0.014433

2,2,2,12,1,0.014819

2,2,2,12,2,0.01405

2,2,2,12,3,-0.025226

2,2,2,12,4,-0.064091

2,2,2,12,5,0.0037711

2,2,2,12,6,-0.024668

2,2,2,12,7,-0.010163

2,2,2,12,8,0.0021848

2,2,2,13,1,0.029303

2,2,2,13,2,-0.038007

2,2,2,13,3,-0.049749

2,2,2,13,4,-0.040511

2,2,2,13,5,-0.029509

2,2,2,13,6,-0.020357

2,2,2,13,7,-0.017666

2,2,2,13,8,0.004939

2,2,2,14,1,0.014387

2,2,2,14,2,-0.0028235

2,2,2,14,3,-0.031969

2,2,2,14,4,-0.021916

2,2,2,14,5,-0.029563

2,2,2,14,6,-0.017042

2,2,2,14,7,-0.019308

2,2,2,14,8,0.0073099

2,2,2,15,1,0.010311

2,2,2,15,2,-0.008567

2,2,2,15,3,-0.03033

2,2,2,15,4,-0.044604

2,2,2,15,5,-0.019794

2,2,2,15,6,-0.024544

2,2,2,15,7,-0.03712

2,2,2,15,8,0.00058533

2,2,2,16,1,0.0022519

2,2,2,16,2,-0.0017663

2,2,2,16,3,-0.036919

2,2,2,16,4,0.015809

2,2,2,16,5,-0.038014

2,2,2,16,6,-0.010991

2,2,2,16,7,-0.013175

2,2,2,16,8,0.0055262

2,2,2,17,1,0.0325

2,2,2,17,2,-0.0095712

2,2,2,17,3,-0.028328

2,2,2,17,4,-0.031228

2,2,2,17,5,-0.02124

2,2,2,17,6,-0.026568

2,2,2,17,7,-0.010211

2,2,2,17,8,-0.0029787

2,2,2,18,1,4.1649e-11

2,2,2,18,2,0.017944

2,2,2,18,3,-0.028336

2,2,2,18,4,-0.036813

2,2,2,18,5,-0.017403

2,2,2,18,6,-0.0084002

2,2,2,18,7,-0.016219

2,2,2,18,8,0.019988

2,2,2,19,1,7.72e-12

2,2,2,19,2,0.01007

2,2,2,19,3,-0.033334

2,2,2,19,4,-0.019736

2,2,2,19,5,-0.016663

2,2,2,19,6,-0.021876

2,2,2,19,7,0.00042078

2,2,2,19,8,0.01456

2,2,2,20,1,3.2154e-11

2,2,2,20,2,0.0080973

2,2,2,20,3,-0.018996

2,2,2,20,4,-0.0064589

2,2,2,20,5,-0.017291

2,2,2,20,6,-0.013218

2,2,2,20,7,-0.011825

2,2,2,20,8,0.010051

2,2,3,3,1,0.052529

2,2,3,3,2,0.033576

2,2,3,3,3,-0.045122

2,2,3,3,4,-0.025934

2,2,3,3,5,0.00037968

2,2,3,3,6,-0.0048596

2,2,3,3,7,-0.015711

2,2,3,3,8,-0.0053045

2,2,3,4,1,0.092473

2,2,3,4,2,0.040811

2,2,3,4,3,-0.065772

2,2,3,4,4,-0.034307

2,2,3,4,5,-0.0037047

2,2,3,4,6,-0.015738

2,2,3,4,7,-0.0099265

2,2,3,4,8,-0.023511

2,2,3,5,1,0.10667

2,2,3,5,2,0.033673

2,2,3,5,3,-0.067282

2,2,3,5,4,-0.043898

2,2,3,5,5,-0.022198

2,2,3,5,6,-0.01131

2,2,3,5,7,-0.034911

2,2,3,5,8,-0.023714

2,2,3,6,1,0.0575

2,2,3,6,2,0.01774

2,2,3,6,3,-0.12734

2,2,3,6,4,-0.07908

2,2,3,6,5,-0.015349

2,2,3,6,6,-0.021279

2,2,3,6,7,-0.021138

2,2,3,6,8,-0.013577

2,2,3,7,1,0.054554

2,2,3,7,2,0.026366

2,2,3,7,3,-0.041142

2,2,3,7,4,-0.032211

2,2,3,7,5,0.0099372

2,2,3,7,6,0.01954

2,2,3,7,7,-0.0037561

2,2,3,7,8,-0.013171

2,2,3,8,1,0.080027

2,2,3,8,2,-0.016783

2,2,3,8,3,-0.15235

2,2,3,8,4,-0.061819

2,2,3,8,5,-0.037431

2,2,3,8,6,-0.035721

2,2,3,8,7,-0.032166

2,2,3,8,8,-0.028918

2,2,3,9,1,0.077739

2,2,3,9,2,0.022079

2,2,3,9,3,-0.09051

2,2,3,9,4,-0.043172

2,2,3,9,5,-0.030202

2,2,3,9,6,-0.016916

2,2,3,9,7,-0.015908

2,2,3,9,8,-0.023499

2,2,3,10,1,0.072136

2,2,3,10,2,-0.036657

2,2,3,10,3,-0.13286

2,2,3,10,4,-0.084063

2,2,3,10,5,-0.0374

2,2,3,10,6,-0.028069

2,2,3,10,7,-0.02561

2,2,3,10,8,-0.027106

2,2,3,11,1,0.061723

2,2,3,11,2,-0.016977

2,2,3,11,3,-0.11635

2,2,3,11,4,-0.068947

2,2,3,11,5,-0.039767

2,2,3,11,6,-0.027283

2,2,3,11,7,-0.02719

2,2,3,11,8,-0.015188

2,2,3,12,1,0.037085

2,2,3,12,2,0.02643

2,2,3,12,3,-0.075092

2,2,3,12,4,-0.078631

2,2,3,12,5,-0.029801

2,2,3,12,6,-0.031273

2,2,3,12,7,-0.041747

2,2,3,12,8,-0.037135

2,2,3,13,1,0.074316

2,2,3,13,2,0.038083

2,2,3,13,3,-0.026306

2,2,3,13,4,-0.040066

2,2,3,13,5,-0.016619

2,2,3,13,6,0.0026558

2,2,3,13,7,-0.02106

2,2,3,13,8,-0.007443

2,2,3,14,1,0.073073

2,2,3,14,2,0.049294

2,2,3,14,3,-0.053174

2,2,3,14,4,-0.045011

2,2,3,14,5,-0.0044166

2,2,3,14,6,-0.0088674

2,2,3,14,7,-0.0070646

2,2,3,14,8,-0.013532

2,2,3,15,1,0.030673

2,2,3,15,2,-0.0059378

2,2,3,15,3,-0.054504

2,2,3,15,4,-0.053548

2,2,3,15,5,-0.011796

2,2,3,15,6,-0.0306

2,2,3,15,7,-0.020552

2,2,3,15,8,-0.0087027

2,2,3,16,1,0.022259

2,2,3,16,2,0.010168

2,2,3,16,3,-0.041058

2,2,3,16,4,-0.029671

2,2,3,16,5,-0.005876

2,2,3,16,6,-0.016234

2,2,3,16,7,-0.011541

2,2,3,16,8,0.0041137

2,2,3,17,1,0.11157

2,2,3,17,2,0.061506

2,2,3,17,3,-0.045887

2,2,3,17,4,-0.0392

2,2,3,17,5,-0.0046952

2,2,3,17,6,0.0038256

2,2,3,17,7,-0.015784

2,2,3,17,8,-0.01877

2,2,3,18,1,0.045162

2,2,3,18,2,0.0259

2,2,3,18,3,-0.042125

2,2,3,18,4,-0.046048

2,2,3,18,5,0.012775

2,2,3,18,6,-0.011915

2,2,3,18,7,-0.012248

2,2,3,18,8,-0.0090641

2,2,3,19,1,0.034485

2,2,3,19,2,0.010051

2,2,3,19,3,-0.021459

2,2,3,19,4,-0.058198

2,2,3,19,5,-0.0079344

2,2,3,19,6,-0.019457

2,2,3,19,7,-0.023912

2,2,3,19,8,-0.0068886

2,2,3,20,1,0.021755

2,2,3,20,2,0.0074809

2,2,3,20,3,-0.033651

2,2,3,20,4,-0.059424

2,2,3,20,5,0.007382

2,2,3,20,6,-0.0122

2,2,3,20,7,0.015479

2,2,3,20,8,0.0080016

2,2,4,4,1,0.10734

2,2,4,4,2,-0.0098949

2,2,4,4,3,-0.0084685

2,2,4,4,4,-0.016268

2,2,4,4,5,-0.017017

2,2,4,4,6,-0.0051855

2,2,4,4,7,-0.01703

2,2,4,4,8,0.0049991

2,2,4,5,1,0.088662

2,2,4,5,2,-0.011352

2,2,4,5,3,-0.035325

2,2,4,5,4,-0.06786

2,2,4,5,5,-0.022816

2,2,4,5,6,-0.0397

2,2,4,5,7,-0.037821

2,2,4,5,8,-0.016247

2,2,4,6,1,0.071962

2,2,4,6,2,-0.025108

2,2,4,6,3,-0.071008

2,2,4,6,4,-0.052846

2,2,4,6,5,-0.012387

2,2,4,6,6,-0.022404

2,2,4,6,7,-0.026465

2,2,4,6,8,-0.0080954

2,2,4,7,1,0.046751

2,2,4,7,2,0.0069926

2,2,4,7,3,-0.060609

2,2,4,7,4,-0.049687

2,2,4,7,5,-0.025292

2,2,4,7,6,-0.00761

2,2,4,7,7,-0.019097

2,2,4,7,8,-0.021795

2,2,4,8,1,0.061118

2,2,4,8,2,0.0089543

2,2,4,8,3,-0.068511

2,2,4,8,4,-0.031308

2,2,4,8,5,-0.0039063

2,2,4,8,6,0.0044296

2,2,4,8,7,-0.005377

2,2,4,8,8,0.0066293

2,2,4,9,1,0.06577

2,2,4,9,2,-0.0050441

2,2,4,9,3,-0.053919

2,2,4,9,4,-0.021446

2,2,4,9,5,-0.035602

2,2,4,9,6,-0.02875

2,2,4,9,7,-0.023816

2,2,4,9,8,-0.017389

2,2,4,10,1,0.10482

2,2,4,10,2,-0.041494

2,2,4,10,3,-0.080757

2,2,4,10,4,-0.076391

2,2,4,10,5,-0.044473

2,2,4,10,6,-0.036032

2,2,4,10,7,-0.031263

2,2,4,10,8,-0.0091208

2,2,4,11,1,0.042196

2,2,4,11,2,0.017028

2,2,4,11,3,-0.094487

2,2,4,11,4,-0.06688

2,2,4,11,5,-0.042448

2,2,4,11,6,-0.01741

2,2,4,11,7,-0.032735

2,2,4,11,8,-0.013927

2,2,4,12,1,0.031767

2,2,4,12,2,0.021311

2,2,4,12,3,-0.030839

2,2,4,12,4,-0.056015

2,2,4,12,5,-0.011344

2,2,4,12,6,-0.018102

2,2,4,12,7,-0.0069257

2,2,4,12,8,-0.0062578

2,2,4,13,1,0.089938

2,2,4,13,2,-0.01721

2,2,4,13,3,-0.052693

2,2,4,13,4,-0.060783

2,2,4,13,5,-0.036858

2,2,4,13,6,-0.036743

2,2,4,13,7,-0.03678

2,2,4,13,8,0.00265

2,2,4,14,1,0.096443

2,2,4,14,2,-0.023032

2,2,4,14,3,-0.04995

2,2,4,14,4,-0.039666

2,2,4,14,5,-0.030801

2,2,4,14,6,-0.01604

2,2,4,14,7,-0.053023

2,2,4,14,8,0.0016683

2,2,4,15,1,0.029887

2,2,4,15,2,0.0083701

2,2,4,15,3,-0.014132

2,2,4,15,4,-0.051928

2,2,4,15,5,-0.0184

2,2,4,15,6,-0.014086

2,2,4,15,7,-0.027575

2,2,4,15,8,-0.0015834

2,2,4,16,1,0.016538

2,2,4,16,2,-0.032119

2,2,4,16,3,-0.025738

2,2,4,16,4,-0.029593

2,2,4,16,5,-0.014355

2,2,4,16,6,-0.027052

2,2,4,16,7,-0.011794

2,2,4,16,8,-0.018221

2,2,4,17,1,0.11979

2,2,4,17,2,0.0065662

2,2,4,17,3,-0.028062

2,2,4,17,4,-0.060122

2,2,4,17,5,-0.021095

2,2,4,17,6,-0.044888

2,2,4,17,7,-0.044169

2,2,4,17,8,-0.0065155

2,2,4,18,1,0.064166

2,2,4,18,2,0.0020937

2,2,4,18,3,-0.030153

2,2,4,18,4,-0.029196

2,2,4,18,5,-0.017154

2,2,4,18,6,-0.020387

2,2,4,18,7,-0.025185

2,2,4,18,8,-0.0030452

2,2,4,19,1,0.047362

2,2,4,19,2,0.03841

2,2,4,19,3,-0.021042

2,2,4,19,4,-0.0017315

2,2,4,19,5,-0.02056

2,2,4,19,6,-0.020948

2,2,4,19,7,-0.0056286

2,2,4,19,8,-0.0091312

2,2,4,20,1,0.023225

2,2,4,20,2,-0.0077363

2,2,4,20,3,-0.005539

2,2,4,20,4,-0.024709

2,2,4,20,5,-0.009062

2,2,4,20,6,-0.0089778

2,2,4,20,7,-0.0092682

2,2,4,20,8,0.018654

2,2,5,5,1,0.13442

2,2,5,5,2,0.0020313

2,2,5,5,3,-0.0091643

2,2,5,5,4,-0.028716

2,2,5,5,5,-0.010687

2,2,5,5,6,-0.01626

2,2,5,5,7,-0.016258

2,2,5,5,8,-0.011033

2,2,5,6,1,0.070079

2,2,5,6,2,0.012722

2,2,5,6,3,-0.073637

2,2,5,6,4,-0.058471

2,2,5,6,5,-0.023219

2,2,5,6,6,-0.030647

2,2,5,6,7,-0.028172

2,2,5,6,8,-0.038254

2,2,5,7,1,0.078098

2,2,5,7,2,0.0096469

2,2,5,7,3,-0.065584

2,2,5,7,4,-0.07431

2,2,5,7,5,-0.0082922

2,2,5,7,6,-0.021737

2,2,5,7,7,-0.008851

2,2,5,7,8,-0.024704

2,2,5,8,1,0.070036

2,2,5,8,2,0.039129

2,2,5,8,3,-0.063261

2,2,5,8,4,-0.061029

2,2,5,8,5,-0.0025968

2,2,5,8,6,-0.012533

2,2,5,8,7,-0.01869

2,2,5,8,8,-0.020321

2,2,5,9,1,0.062081

2,2,5,9,2,0.018023

2,2,5,9,3,-0.027354

2,2,5,9,4,-0.02153

2,2,5,9,5,-0.016728

2,2,5,9,6,-0.0058142

2,2,5,9,7,-0.031458

2,2,5,9,8,-0.00087526

2,2,5,10,1,0.11209

2,2,5,10,2,0.043123

2,2,5,10,3,-0.063721

2,2,5,10,4,-0.060465

2,2,5,10,5,-0.01763

2,2,5,10,6,-0.021281

2,2,5,10,7,-0.02895

2,2,5,10,8,-0.025197

2,2,5,11,1,0.068958

2,2,5,11,2,0.021099

2,2,5,11,3,-0.11662

2,2,5,11,4,-0.073144

2,2,5,11,5,-0.045897

2,2,5,11,6,-0.049592

2,2,5,11,7,-0.044028

2,2,5,11,8,-0.04366

2,2,5,12,1,0.039913

2,2,5,12,2,0.021571

2,2,5,12,3,-0.048059

2,2,5,12,4,-0.055245

2,2,5,12,5,0.0039122

2,2,5,12,6,-0.013182

2,2,5,12,7,-0.021639

2,2,5,12,8,0.0020507

2,2,5,13,1,0.13528

2,2,5,13,2,0.016985

2,2,5,13,3,-0.057011

2,2,5,13,4,-0.043101

2,2,5,13,5,-0.037916

2,2,5,13,6,-0.028544

2,2,5,13,7,-0.02841

2,2,5,13,8,-0.0036895

2,2,5,14,1,0.089305

2,2,5,14,2,-0.024798

2,2,5,14,3,-0.068759

2,2,5,14,4,-0.057788

2,2,5,14,5,-0.049181

2,2,5,14,6,-0.042162

2,2,5,14,7,-0.0317

2,2,5,14,8,-0.011673

2,2,5,15,1,0.036022

2,2,5,15,2,-0.0088335

2,2,5,15,3,-0.0049385

2,2,5,15,4,-0.039425

2,2,5,15,5,-0.015184

2,2,5,15,6,-0.01997

2,2,5,15,7,-0.016178

2,2,5,15,8,0.010927

2,2,5,16,1,0.023704

2,2,5,16,2,-0.023613

2,2,5,16,3,-0.0093361

2,2,5,16,4,-0.02393

2,2,5,16,5,-0.011535

2,2,5,16,6,-0.042089

2,2,5,16,7,-0.017375

2,2,5,16,8,-0.033113

2,2,5,17,1,0.1566

2,2,5,17,2,0.031401

2,2,5,17,3,-0.045292

2,2,5,17,4,-0.067363

2,2,5,17,5,-0.04588

2,2,5,17,6,-0.054059

2,2,5,17,7,-0.039263

2,2,5,17,8,-0.024931

2,2,5,18,1,0.03111

2,2,5,18,2,0.0061554

2,2,5,18,3,-0.040813

2,2,5,18,4,-0.058023

2,2,5,18,5,-0.034235

2,2,5,18,6,-0.05231

2,2,5,18,7,-0.028778

2,2,5,18,8,-0.015942

2,2,5,19,1,0.03409

2,2,5,19,2,0.011297

2,2,5,19,3,-0.036863

2,2,5,19,4,-0.048752

2,2,5,19,5,-0.028204

2,2,5,19,6,-0.040087

2,2,5,19,7,-0.029903

2,2,5,19,8,-0.015772

2,2,5,20,1,0.022077

2,2,5,20,2,0.0057802

2,2,5,20,3,-0.0067092

2,2,5,20,4,-0.03541

2,2,5,20,5,-0.025403

2,2,5,20,6,-0.02108

2,2,5,20,7,-0.028728

2,2,5,20,8,0.0049613

2,2,6,6,1,0.0627

2,2,6,6,2,-0.0037668

2,2,6,6,3,-0.090906

2,2,6,6,4,-0.049781

2,2,6,6,5,-0.037044

2,2,6,6,6,-0.025362

2,2,6,6,7,-0.02487

2,2,6,6,8,-0.025639

2,2,6,7,1,0.056783

2,2,6,7,2,0.0094385

2,2,6,7,3,-0.09251

2,2,6,7,4,-0.065026

2,2,6,7,5,-0.013854

2,2,6,7,6,-0.019364

2,2,6,7,7,-0.029222

2,2,6,7,8,-0.014672

2,2,6,8,1,0.063057

2,2,6,8,2,-0.00092734

2,2,6,8,3,-0.094503

2,2,6,8,4,-0.067871

2,2,6,8,5,-0.024109

2,2,6,8,6,-0.025927

2,2,6,8,7,-0.014198

2,2,6,8,8,-0.03137

2,2,6,9,1,0.051885

2,2,6,9,2,0.016652

2,2,6,9,3,-0.063503

2,2,6,9,4,-0.0335

2,2,6,9,5,-0.032931

2,2,6,9,6,0.0096696

2,2,6,9,7,-0.037237

2,2,6,9,8,0.0043986

2,2,6,10,1,0.062043

2,2,6,10,2,-0.013825

2,2,6,10,3,-0.096638

2,2,6,10,4,-0.076319

2,2,6,10,5,0.00027379

2,2,6,10,6,-0.021147

2,2,6,10,7,-0.018404

2,2,6,10,8,-0.026679

2,2,6,11,1,0.05652

2,2,6,11,2,0.010312

2,2,6,11,3,-0.08234

2,2,6,11,4,-0.068805

2,2,6,11,5,-0.01096

2,2,6,11,6,-0.00083984

2,2,6,11,7,-0.021144

2,2,6,11,8,-0.017965

2,2,6,12,1,0.031049

2,2,6,12,2,0.026105

2,2,6,12,3,-0.073743

2,2,6,12,4,-0.085433

2,2,6,12,5,-0.030295

2,2,6,12,6,-0.029727

2,2,6,12,7,-0.022234

2,2,6,12,8,-0.010294

2,2,6,13,1,0.080921

2,2,6,13,2,0.014717

2,2,6,13,3,-0.025973

2,2,6,13,4,-0.032625

2,2,6,13,5,-0.013688

2,2,6,13,6,0.002545

2,2,6,13,7,-0.0057877

2,2,6,13,8,-0.028019

2,2,6,14,1,0.060303

2,2,6,14,2,0.016558

2,2,6,14,3,-0.034814

2,2,6,14,4,-0.043581

2,2,6,14,5,-0.010444

2,2,6,14,6,-0.0012023

2,2,6,14,7,-0.013312

2,2,6,14,8,-0.0082877

2,2,6,15,1,0.030162

2,2,6,15,2,0.025984

2,2,6,15,3,-0.04131

2,2,6,15,4,-0.046017

2,2,6,15,5,-0.0097123

2,2,6,15,6,-0.001101

2,2,6,15,7,-0.043269

2,2,6,15,8,-0.029695

2,2,6,16,1,0.023412

2,2,6,16,2,0.012375

2,2,6,16,3,-0.037926

2,2,6,16,4,-0.038159

2,2,6,16,5,-0.023418

2,2,6,16,6,-0.022235

2,2,6,16,7,-0.02219

2,2,6,16,8,-0.0078864

2,2,6,17,1,0.080734

2,2,6,17,2,0.048024

2,2,6,17,3,-0.045223

2,2,6,17,4,-0.061242

2,2,6,17,5,0.0068767

2,2,6,17,6,0.00080419

2,2,6,17,7,-0.010211

2,2,6,17,8,-0.025373

2,2,6,18,1,0.04637

2,2,6,18,2,0.023398

2,2,6,18,3,-0.066391

2,2,6,18,4,-0.039388

2,2,6,18,5,-0.014251

2,2,6,18,6,-0.0079167

2,2,6,18,7,-0.019661

2,2,6,18,8,-0.014227

2,2,6,19,1,0.036077

2,2,6,19,2,0.01214

2,2,6,19,3,-0.025485

2,2,6,19,4,-0.058498

2,2,6,19,5,-0.0091068

2,2,6,19,6,-0.0029192

2,2,6,19,7,-0.0070727

2,2,6,19,8,-0.017358

2,2,6,20,1,0.020226

2,2,6,20,2,-0.00028624

2,2,6,20,3,-0.042237

2,2,6,20,4,-0.049699

2,2,6,20,5,-0.01133

2,2,6,20,6,0.0059742

2,2,6,20,7,-0.020846

2,2,6,20,8,0.0060259

2,2,7,7,1,0.033308

2,2,7,7,2,0.01178

2,2,7,7,3,-0.027838

2,2,7,7,4,-0.049997

2,2,7,7,5,-0.017837

2,2,7,7,6,-0.007481

2,2,7,7,7,-0.025939

2,2,7,7,8,-0.020286

2,2,7,8,1,0.056858

2,2,7,8,2,-0.013113

2,2,7,8,3,-0.076265

2,2,7,8,4,-0.067632

2,2,7,8,5,-0.03128

2,2,7,8,6,-0.020681

2,2,7,8,7,-0.037273

2,2,7,8,8,-0.036842

2,2,7,9,1,0.014545

2,2,7,9,2,0.0046553

2,2,7,9,3,-0.082637

2,2,7,9,4,-0.047376

2,2,7,9,5,-0.032334

2,2,7,9,6,-0.0061735

2,2,7,9,7,-0.011144

2,2,7,9,8,-0.010334

2,2,7,10,1,0.053939

2,2,7,10,2,0.010608

2,2,7,10,3,-0.095822

2,2,7,10,4,-0.058761

2,2,7,10,5,-0.034586

2,2,7,10,6,0.0015712

2,2,7,10,7,-0.048263

2,2,7,10,8,-0.017573

2,2,7,11,1,0.027672

2,2,7,11,2,-0.0093151

2,2,7,11,3,-0.067168

2,2,7,11,4,-0.088916

2,2,7,11,5,-0.044518

2,2,7,11,6,-0.020121

2,2,7,11,7,-0.012519

2,2,7,11,8,-0.015845

2,2,7,12,1,0.015585

2,2,7,12,2,0.012716

2,2,7,12,3,-0.0086809

2,2,7,12,4,-0.060186

2,2,7,12,5,0.0069092

2,2,7,12,6,-0.0046131

2,2,7,12,7,-0.01947

2,2,7,12,8,-0.014036

2,2,7,13,1,0.084304

2,2,7,13,2,0.00050759

2,2,7,13,3,-0.074348

2,2,7,13,4,-0.063226

2,2,7,13,5,-0.022239

2,2,7,13,6,0.0067334

2,2,7,13,7,-0.015555

2,2,7,13,8,-0.0019512

2,2,7,14,1,0.058123

2,2,7,14,2,-0.029189

2,2,7,14,3,-0.052438

2,2,7,14,4,-0.086216

2,2,7,14,5,-0.033821

2,2,7,14,6,-0.017083

2,2,7,14,7,-0.014793

2,2,7,14,8,-0.012537

2,2,7,15,1,0.021679

2,2,7,15,2,0.016278

2,2,7,15,3,-0.042547

2,2,7,15,4,-0.034567

2,2,7,15,5,-0.005775

2,2,7,15,6,-0.018603

2,2,7,15,7,0.0027138

2,2,7,15,8,-0.00666

2,2,7,16,1,0.028148

2,2,7,16,2,-0.014842

2,2,7,16,3,-0.068184

2,2,7,16,4,-0.052211

2,2,7,16,5,-0.038812

2,2,7,16,6,-0.010827

2,2,7,16,7,-0.033157

2,2,7,16,8,-0.0075801

2,2,7,17,1,0.082838

2,2,7,17,2,0.032595

2,2,7,17,3,-0.081947

2,2,7,17,4,-0.076785

2,2,7,17,5,-0.037473

2,2,7,17,6,-0.029022

2,2,7,17,7,-0.027013

2,2,7,17,8,-0.023876

2,2,7,18,1,0.027829

2,2,7,18,2,0.002267

2,2,7,18,3,-0.058049

2,2,7,18,4,-0.055654

2,2,7,18,5,-0.013526

2,2,7,18,6,0.0055336

2,2,7,18,7,-0.021962

2,2,7,18,8,0.029899

2,2,7,19,1,0.040185

2,2,7,19,2,-0.0060629

2,2,7,19,3,-0.06862

2,2,7,19,4,-0.050749

2,2,7,19,5,-0.034751

2,2,7,19,6,-0.01097

2,2,7,19,7,-0.013619

2,2,7,19,8,-0.01064

2,2,7,20,1,0.0017949

2,2,7,20,2,-0.012127

2,2,7,20,3,-0.041576

2,2,7,20,4,-0.038816

2,2,7,20,5,-0.0016749

2,2,7,20,6,0.0038374

2,2,7,20,7,-0.0010607

2,2,7,20,8,0.030609

2,2,8,8,1,0.099416

2,2,8,8,2,0.0074256

2,2,8,8,3,-0.0487

2,2,8,8,4,-0.015209

2,2,8,8,5,-0.01537

2,2,8,8,6,-0.0090769

2,2,8,8,7,-0.0088354

2,2,8,8,8,-0.021106

2,2,8,9,1,0.064721

2,2,8,9,2,-0.036882

2,2,8,9,3,-0.059649

2,2,8,9,4,-0.019232

2,2,8,9,5,-0.0048622

2,2,8,9,6,-0.028653

2,2,8,9,7,-0.010026

2,2,8,9,8,-0.015621

2,2,8,10,1,0.083457

2,2,8,10,2,-0.023984

2,2,8,10,3,-0.083414

2,2,8,10,4,-0.04848

2,2,8,10,5,-0.018985

2,2,8,10,6,-0.0080275

2,2,8,10,7,-0.018513

2,2,8,10,8,-0.01738

2,2,8,11,1,0.072422

2,2,8,11,2,-0.0070746

2,2,8,11,3,-0.09048

2,2,8,11,4,-0.071519

2,2,8,11,5,-0.028442

2,2,8,11,6,-0.032421

2,2,8,11,7,-0.018582

2,2,8,11,8,-0.027194

2,2,8,12,1,0.049962

2,2,8,12,2,0.020952

2,2,8,12,3,-0.072254

2,2,8,12,4,-0.051051

2,2,8,12,5,-0.0049986

2,2,8,12,6,-0.0037222

2,2,8,12,7,-0.010195

2,2,8,12,8,-0.014127

2,2,8,13,1,0.10527

2,2,8,13,2,0.0047516

2,2,8,13,3,-0.064001

2,2,8,13,4,-0.055927

2,2,8,13,5,-0.021744

2,2,8,13,6,-0.019368

2,2,8,13,7,-0.024088

2,2,8,13,8,-0.020148

2,2,8,14,1,0.070121

2,2,8,14,2,0.024852

2,2,8,14,3,-0.052287

2,2,8,14,4,-0.046857

2,2,8,14,5,-0.0081179

2,2,8,14,6,0.0033862

2,2,8,14,7,-0.014328

2,2,8,14,8,-0.00098785

2,2,8,15,1,0.032135

2,2,8,15,2,-0.0013037

2,2,8,15,3,-0.078585

2,2,8,15,4,-0.047465

2,2,8,15,5,-0.029414

2,2,8,15,6,-0.011433

2,2,8,15,7,-0.014873

2,2,8,15,8,0.0145

2,2,8,16,1,0.029183

2,2,8,16,2,0.025393

2,2,8,16,3,-0.048323

2,2,8,16,4,-0.033082

2,2,8,16,5,-0.0054188

2,2,8,16,6,-0.016953

2,2,8,16,7,-0.0040985

2,2,8,16,8,-0.010938

2,2,8,17,1,0.1052

2,2,8,17,2,0.069942

2,2,8,17,3,-0.047923

2,2,8,17,4,-0.037132

2,2,8,17,5,0.0069806

2,2,8,17,6,-0.0079646

2,2,8,17,7,-0.0037021

2,2,8,17,8,0.0016699

2,2,8,18,1,0.062838

2,2,8,18,2,0.0044594

2,2,8,18,3,-0.076471

2,2,8,18,4,-0.061548

2,2,8,18,5,-0.0018171

2,2,8,18,6,-0.021825

2,2,8,18,7,0.0038651

2,2,8,18,8,-0.029819

2,2,8,19,1,0.036624

2,2,8,19,2,-0.018449

2,2,8,19,3,-0.057793

2,2,8,19,4,-0.040473

2,2,8,19,5,-0.0092769

2,2,8,19,6,0.0020661

2,2,8,19,7,-0.002655

2,2,8,19,8,0.0050079

2,2,8,20,1,0.01955

2,2,8,20,2,0.025771

2,2,8,20,3,-0.044333

2,2,8,20,4,-0.061982

2,2,8,20,5,0.016001

2,2,8,20,6,-0.01808

2,2,8,20,7,0.0079316

2,2,8,20,8,-0.014254

2,2,9,9,1,0.080606

2,2,9,9,2,-0.041756

2,2,9,9,3,-0.0097242

2,2,9,9,4,-0.021656

2,2,9,9,5,-0.023231

2,2,9,9,6,-0.01992

2,2,9,9,7,-0.011843

2,2,9,9,8,-0.019369

2,2,9,10,1,0.060518

2,2,9,10,2,-0.0024422

2,2,9,10,3,-0.068878

2,2,9,10,4,-0.043659

2,2,9,10,5,-0.028581

2,2,9,10,6,-0.005021

2,2,9,10,7,-0.0082115

2,2,9,10,8,-0.0064081

2,2,9,11,1,0.0665

2,2,9,11,2,0.0055993

2,2,9,11,3,-0.071255

2,2,9,11,4,-0.048451

2,2,9,11,5,-0.048001

2,2,9,11,6,-0.0060237

2,2,9,11,7,-0.030724

2,2,9,11,8,-0.0094553

2,2,9,12,1,0.033371

2,2,9,12,2,0.019193

2,2,9,12,3,-0.05509

2,2,9,12,4,-0.030131

2,2,9,12,5,-0.0075088

2,2,9,12,6,-0.0108

2,2,9,12,7,0.010494

2,2,9,12,8,0.0007683

2,2,9,13,1,0.090713

2,2,9,13,2,-0.011651

2,2,9,13,3,-0.031939

2,2,9,13,4,-0.038

2,2,9,13,5,-0.016653

2,2,9,13,6,-0.01595

2,2,9,13,7,-0.026415

2,2,9,13,8,0.01602

2,2,9,14,1,0.064192

2,2,9,14,2,-0.023942

2,2,9,14,3,-0.048427

2,2,9,14,4,-0.053677

2,2,9,14,5,-0.031672

2,2,9,14,6,-0.02525

2,2,9,14,7,-0.031781

2,2,9,14,8,0.016411

2,2,9,15,1,0.026004

2,2,9,15,2,0.015834

2,2,9,15,3,-0.034234

2,2,9,15,4,-0.017121

2,2,9,15,5,-0.016691

2,2,9,15,6,-0.022161

2,2,9,15,7,-0.011548

2,2,9,15,8,-0.0049352

2,2,9,16,1,0.03999

2,2,9,16,2,-0.024542

2,2,9,16,3,-0.024913

2,2,9,16,4,-0.014688

2,2,9,16,5,-0.040795

2,2,9,16,6,-0.005469

2,2,9,16,7,-0.028553

2,2,9,16,8,0.0023699

2,2,9,17,1,0.11557

2,2,9,17,2,0.010339

2,2,9,17,3,-0.054158

2,2,9,17,4,-0.048866

2,2,9,17,5,-0.028121

2,2,9,17,6,-0.038325

2,2,9,17,7,-0.020963

2,2,9,17,8,-0.0088105

2,2,9,18,1,0.039032

2,2,9,18,2,0.009656

2,2,9,18,3,-0.04261

2,2,9,18,4,-0.037225

2,2,9,18,5,-0.021398

2,2,9,18,6,-0.026747

2,2,9,18,7,-0.0082275

2,2,9,18,8,0.0039279

2,2,9,19,1,0.025812

2,2,9,19,2,-0.0057572

2,2,9,19,3,-0.054028

2,2,9,19,4,-0.033474

2,2,9,19,5,-0.016446

2,2,9,19,6,-0.021558

2,2,9,19,7,-0.010058

2,2,9,19,8,0.0012637

2,2,9,20,1,0.014969

2,2,9,20,2,0.0056998

2,2,9,20,3,-0.035378

2,2,9,20,4,-0.020988

2,2,9,20,5,-0.027044

2,2,9,20,6,-0.026721

2,2,9,20,7,-0.021461

2,2,9,20,8,0.013668

2,2,10,10,1,0.094324

2,2,10,10,2,-0.034654

2,2,10,10,3,-0.030244

2,2,10,10,4,-0.026733

2,2,10,10,5,-0.013963

2,2,10,10,6,-0.013188

2,2,10,10,7,-0.017194

2,2,10,10,8,-0.015938

2,2,10,11,1,0.079172

2,2,10,11,2,0.029517

2,2,10,11,3,-0.036353

2,2,10,11,4,-0.057273

2,2,10,11,5,-0.011326

2,2,10,11,6,-0.012889

2,2,10,11,7,0.0029497

2,2,10,11,8,0.00058729

2,2,10,12,1,0.030865

2,2,10,12,2,0.008742

2,2,10,12,3,-0.067491

2,2,10,12,4,-0.066411

2,2,10,12,5,-0.014917

2,2,10,12,6,-0.0092412

2,2,10,12,7,-0.014596

2,2,10,12,8,-0.021515

2,2,10,13,1,0.096319

2,2,10,13,2,0.019651

2,2,10,13,3,-0.082986

2,2,10,13,4,-0.052677

2,2,10,13,5,-0.025332

2,2,10,13,6,-0.030002

2,2,10,13,7,-0.017307

2,2,10,13,8,-0.023685

2,2,10,14,1,0.076553

2,2,10,14,2,-0.0041252

2,2,10,14,3,-0.061761

2,2,10,14,4,-0.049025

2,2,10,14,5,-0.022068

2,2,10,14,6,-0.020013

2,2,10,14,7,-0.0076618

2,2,10,14,8,-0.018394

2,2,10,15,1,0.036572

2,2,10,15,2,0.020504

2,2,10,15,3,-0.053171

2,2,10,15,4,-0.049912

2,2,10,15,5,0.0063227

2,2,10,15,6,0.0045039

2,2,10,15,7,-0.013098

2,2,10,15,8,0.019698

2,2,10,16,1,0.025057

2,2,10,16,2,-0.0032237

2,2,10,16,3,-0.033794

2,2,10,16,4,-0.014804

2,2,10,16,5,0.00021405

2,2,10,16,6,-0.0073813

2,2,10,16,7,-0.00062331

2,2,10,16,8,0.0041918

2,2,10,17,1,0.11703

2,2,10,17,2,0.030621

2,2,10,17,3,-0.091037

2,2,10,17,4,-0.071854

2,2,10,17,5,-0.035594

2,2,10,17,6,-0.015476

2,2,10,17,7,-0.022906

2,2,10,17,8,-0.012867

2,2,10,18,1,0.04094

2,2,10,18,2,-0.00763

2,2,10,18,3,-0.082302

2,2,10,18,4,-0.087259

2,2,10,18,5,-0.033196

2,2,10,18,6,-0.031086

2,2,10,18,7,-0.024921

2,2,10,18,8,-0.018157

2,2,10,19,1,0.073738

2,2,10,19,2,-0.021893

2,2,10,19,3,-0.087969

2,2,10,19,4,-0.071976

2,2,10,19,5,-0.036057

2,2,10,19,6,-0.026916

2,2,10,19,7,-0.014024

2,2,10,19,8,-0.018502

2,2,10,20,1,0.027056

2,2,10,20,2,-0.018585

2,2,10,20,3,-0.060636

2,2,10,20,4,-0.075272

2,2,10,20,5,-0.020859

2,2,10,20,6,-0.015813

2,2,10,20,7,-0.014305

2,2,10,20,8,0.031073

2,2,11,11,1,0.079604

2,2,11,11,2,0.00098697

2,2,11,11,3,-0.04715

2,2,11,11,4,-0.042664

2,2,11,11,5,-0.018278

2,2,11,11,6,-0.017601

2,2,11,11,7,-0.023208

2,2,11,11,8,-0.010906

2,2,11,12,1,0.024799

2,2,11,12,2,-0.022993

2,2,11,12,3,-0.06382

2,2,11,12,4,-0.07359

2,2,11,12,5,-0.033161

2,2,11,12,6,-0.023681

2,2,11,12,7,-0.030127

2,2,11,12,8,-0.034617

2,2,11,13,1,0.065065

2,2,11,13,2,-0.046503

2,2,11,13,3,-0.058352

2,2,11,13,4,-0.082204

2,2,11,13,5,-0.042158

2,2,11,13,6,-0.019578

2,2,11,13,7,-0.022035

2,2,11,13,8,-0.02123

2,2,11,14,1,0.074337

2,2,11,14,2,-0.014833

2,2,11,14,3,-0.078161

2,2,11,14,4,-0.072406

2,2,11,14,5,-0.045316

2,2,11,14,6,-0.010828

2,2,11,14,7,-0.028859

2,2,11,14,8,-0.0064999

2,2,11,15,1,0.03093

2,2,11,15,2,0.013345

2,2,11,15,3,-0.056438

2,2,11,15,4,-0.059715

2,2,11,15,5,-0.011083

2,2,11,15,6,-0.0079855

2,2,11,15,7,-0.014835

2,2,11,15,8,0.0017247

2,2,11,16,1,0.03027

2,2,11,16,2,-0.015375

2,2,11,16,3,-0.040491

2,2,11,16,4,-0.053565

2,2,11,16,5,-0.013109

2,2,11,16,6,-0.023158

2,2,11,16,7,0.0020319

2,2,11,16,8,-0.0085569

2,2,11,17,1,0.1192

2,2,11,17,2,0.013629

2,2,11,17,3,-0.11606

2,2,11,17,4,-0.086925

2,2,11,17,5,-0.06162

2,2,11,17,6,-0.041842

2,2,11,17,7,-0.044243

2,2,11,17,8,-0.02715

2,2,11,18,1,0.043745

2,2,11,18,2,-0.025909

2,2,11,18,3,-0.11279

2,2,11,18,4,-0.083762

2,2,11,18,5,-0.067121

2,2,11,18,6,-0.016304

2,2,11,18,7,-0.033195

2,2,11,18,8,-0.017186

2,2,11,19,1,0.044386

2,2,11,19,2,-0.013198

2,2,11,19,3,-0.084919

2,2,11,19,4,-0.055663

2,2,11,19,5,-0.04235

2,2,11,19,6,-0.012014

2,2,11,19,7,-0.016301

2,2,11,19,8,-0.019846

2,2,11,20,1,0.019422

2,2,11,20,2,-0.011434

2,2,11,20,3,-0.06908

2,2,11,20,4,-0.059847

2,2,11,20,5,-0.023028

2,2,11,20,6,0.023

2,2,11,20,7,-0.0050251

2,2,11,20,8,7.1589e-05

2,2,12,12,1,0.025645

2,2,12,12,2,0.0065868

2,2,12,12,3,-0.048945

2,2,12,12,4,-0.024846

2,2,12,12,5,-0.017633

2,2,12,12,6,-0.0036183

2,2,12,12,7,-0.010076

2,2,12,12,8,-0.010568

2,2,12,13,1,0.021598

2,2,12,13,2,-0.019052

2,2,12,13,3,-0.067829

2,2,12,13,4,-0.058418

2,2,12,13,5,-0.016134

2,2,12,13,6,-0.016584

2,2,12,13,7,-0.0090863

2,2,12,13,8,-0.0026803

2,2,12,14,1,0.030131

2,2,12,14,2,0.027808

2,2,12,14,3,-0.06657

2,2,12,14,4,-0.045767

2,2,12,14,5,-0.0039659

2,2,12,14,6,0.0002992

2,2,12,14,7,-0.019026

2,2,12,14,8,-0.0091308

2,2,12,15,1,0.020369

2,2,12,15,2,-0.011863

2,2,12,15,3,-0.050494

2,2,12,15,4,-0.054777

2,2,12,15,5,-0.0011486

2,2,12,15,6,0.0060975

2,2,12,15,7,-0.0053467

2,2,12,15,8,0.00028383

2,2,12,16,1,0.021944

2,2,12,16,2,0.022791

2,2,12,16,3,-0.045979

2,2,12,16,4,-0.08589

2,2,12,16,5,-0.025481

2,2,12,16,6,-0.037354

2,2,12,16,7,-0.016959

2,2,12,16,8,-0.0070215

2,2,12,17,1,0.044561

2,2,12,17,2,0.036671

2,2,12,17,3,-0.069446

2,2,12,17,4,-0.079592

2,2,12,17,5,-0.039163

2,2,12,17,6,-0.033623

2,2,12,17,7,-0.021617

2,2,12,17,8,0.00063432

2,2,12,18,1,0.02528

2,2,12,18,2,0.0075759

2,2,12,18,3,-0.076641

2,2,12,18,4,-0.069509

2,2,12,18,5,-0.0077942

2,2,12,18,6,-0.0066824

2,2,12,18,7,-0.0055222

2,2,12,18,8,0.010777

2,2,12,19,1,0.02591

2,2,12,19,2,-0.01255

2,2,12,19,3,-0.070322

2,2,12,19,4,-0.024614

2,2,12,19,5,-0.015372

2,2,12,19,6,-0.0057179

2,2,12,19,7,-0.018997

2,2,12,19,8,0.010564

2,2,12,20,1,0.011159

2,2,12,20,2,0.0137

2,2,12,20,3,-0.030687

2,2,12,20,4,-0.030483

2,2,12,20,5,-0.0057952

2,2,12,20,6,-0.041154

2,2,12,20,7,-0.01885

2,2,12,20,8,-0.026057

2,2,13,13,1,0.14931

2,2,13,13,2,-0.030654

2,2,13,13,3,-0.041936

2,2,13,13,4,-0.036277

2,2,13,13,5,-0.033039

2,2,13,13,6,-0.034036

2,2,13,13,7,-0.016215

2,2,13,13,8,-0.021026

2,2,13,14,1,0.11772

2,2,13,14,2,-0.04332

2,2,13,14,3,-0.067765

2,2,13,14,4,-0.053041

2,2,13,14,5,-0.04839

2,2,13,14,6,-0.037361

2,2,13,14,7,-0.030997

2,2,13,14,8,-0.01157

2,2,13,15,1,0.030489

2,2,13,15,2,0.01585

2,2,13,15,3,-0.070572

2,2,13,15,4,-0.03728

2,2,13,15,5,-0.029898

2,2,13,15,6,-0.037634

2,2,13,15,7,-0.029033

2,2,13,15,8,-0.0033372

2,2,13,16,1,0.024642

2,2,13,16,2,-0.03366

2,2,13,16,3,-0.038964

2,2,13,16,4,-0.043212

2,2,13,16,5,-0.033151

2,2,13,16,6,-0.023929

2,2,13,16,7,-0.021647

2,2,13,16,8,-0.0036128

2,2,13,17,1,0.15089

2,2,13,17,2,-0.01304

2,2,13,17,3,-0.072938

2,2,13,17,4,-0.064734

2,2,13,17,5,-0.059394

2,2,13,17,6,-0.048204

2,2,13,17,7,-0.038406

2,2,13,17,8,-0.020344

2,2,13,18,1,0.059308

2,2,13,18,2,-0.023301

2,2,13,18,3,-0.070276

2,2,13,18,4,-0.042432

2,2,13,18,5,-0.045606

2,2,13,18,6,-0.042806

2,2,13,18,7,-0.018173

2,2,13,18,8,-0.0081619

2,2,13,19,1,0.040658

2,2,13,19,2,-0.011881

2,2,13,19,3,-0.076911

2,2,13,19,4,-0.040111

2,2,13,19,5,-0.031388

2,2,13,19,6,-0.034452

2,2,13,19,7,-0.0098402

2,2,13,19,8,0.0018293

2,2,13,20,1,0.020816

2,2,13,20,2,-0.014072

2,2,13,20,3,-0.030034

2,2,13,20,4,-0.037863

2,2,13,20,5,-0.0096465

2,2,13,20,6,-0.017494

2,2,13,20,7,0.0038797

2,2,13,20,8,0.022829

2,2,14,14,1,0.065337

2,2,14,14,2,-0.024937

2,2,14,14,3,-0.04045

2,2,14,14,4,-0.021561

2,2,14,14,5,-0.031669

2,2,14,14,6,-0.012136

2,2,14,14,7,-0.0054184

2,2,14,14,8,0.0030827

2,2,14,15,1,0.026777

2,2,14,15,2,-0.0087942

2,2,14,15,3,-0.053618

2,2,14,15,4,-0.058159

2,2,14,15,5,-0.045495

2,2,14,15,6,-0.03179

2,2,14,15,7,-0.011187

2,2,14,15,8,0.012916

2,2,14,16,1,0.026636

2,2,14,16,2,-0.0013195

2,2,14,16,3,-0.03704

2,2,14,16,4,-0.013579

2,2,14,16,5,-0.042482

2,2,14,16,6,-0.037583

2,2,14,16,7,-0.0083471

2,2,14,16,8,-0.027727

2,2,14,17,1,0.093857

2,2,14,17,2,-0.02546

2,2,14,17,3,-0.095976

2,2,14,17,4,-0.082589

2,2,14,17,5,-0.057062

2,2,14,17,6,-0.06911

2,2,14,17,7,-0.038328

2,2,14,17,8,-0.028492

2,2,14,18,1,0.039071

2,2,14,18,2,-0.014167

2,2,14,18,3,-0.07784

2,2,14,18,4,-0.038298

2,2,14,18,5,-0.045502

2,2,14,18,6,-0.030242

2,2,14,18,7,-0.020828

2,2,14,18,8,0.00012175

2,2,14,19,1,0.027494

2,2,14,19,2,-0.039597

2,2,14,19,3,-0.086767

2,2,14,19,4,-0.048224

2,2,14,19,5,-0.052538

2,2,14,19,6,-0.026103

2,2,14,19,7,-0.027055

2,2,14,19,8,0.00067674

2,2,14,20,1,0.024579

2,2,14,20,2,0.0054133

2,2,14,20,3,-0.047857

2,2,14,20,4,-0.044466

2,2,14,20,5,-0.027483

2,2,14,20,6,-0.032338

2,2,14,20,7,-0.019128

2,2,14,20,8,0.017685

2,2,15,15,1,0.018353

2,2,15,15,2,3.5124e-05

2,2,15,15,3,-0.041345

2,2,15,15,4,-0.020835

2,2,15,15,5,-0.015525

2,2,15,15,6,-0.014512

2,2,15,15,7,-0.019214

2,2,15,15,8,0.021206

2,2,15,16,1,0.0088971

2,2,15,16,2,0.0025439

2,2,15,16,3,-0.012417

2,2,15,16,4,-0.0068299

2,2,15,16,5,-0.014957

2,2,15,16,6,-0.020157

2,2,15,16,7,0.0026513

2,2,15,16,8,0.011855

2,2,15,17,1,0.043185

2,2,15,17,2,0.0037125

2,2,15,17,3,-0.076793

2,2,15,17,4,-0.077516

2,2,15,17,5,-0.053303

2,2,15,17,6,-0.0399

2,2,15,17,7,-0.05213

2,2,15,17,8,-0.00014498

2,2,15,18,1,0.0084793

2,2,15,18,2,-0.0023356

2,2,15,18,3,-0.023104

2,2,15,18,4,-0.047346

2,2,15,18,5,-0.028536

2,2,15,18,6,-0.011506

2,2,15,18,7,-0.01681

2,2,15,18,8,0.011571

2,2,15,19,1,0.014932

2,2,15,19,2,-0.0072913

2,2,15,19,3,-0.040675

2,2,15,19,4,-0.019041

2,2,15,19,5,-0.016812

2,2,15,19,6,-0.019148

2,2,15,19,7,-0.016399

2,2,15,19,8,0.0075601

2,2,15,20,1,0.0048426

2,2,15,20,2,0.0031412

2,2,15,20,3,-0.018573

2,2,15,20,4,-0.029174

2,2,15,20,5,-0.03239

2,2,15,20,6,-0.022719

2,2,15,20,7,-0.0051671

2,2,15,20,8,0.03157

2,2,16,16,1,1.1332e-11

2,2,16,16,2,-0.095312

2,2,16,16,3,-0.033839

2,2,16,16,4,-0.050644

2,2,16,16,5,-0.025913

2,2,16,16,6,-0.040626

2,2,16,16,7,-0.014383

2,2,16,16,8,-0.022491

2,2,16,17,1,0.031861

2,2,16,17,2,0.00949

2,2,16,17,3,-0.045634

2,2,16,17,4,-0.047868

2,2,16,17,5,-0.021376

2,2,16,17,6,-0.041748

2,2,16,17,7,-0.012381

2,2,16,17,8,-0.011056

2,2,16,18,1,0.013452

2,2,16,18,2,-0.026825

2,2,16,18,3,-0.06273

2,2,16,18,4,-0.015966

2,2,16,18,5,-0.033713

2,2,16,18,6,-0.019649

2,2,16,18,7,-0.012429

2,2,16,18,8,0.0015587

2,2,16,19,1,0.01482

2,2,16,19,2,0.014311

2,2,16,19,3,-0.052508

2,2,16,19,4,-0.029674

2,2,16,19,5,-0.017561

2,2,16,19,6,-0.04462

2,2,16,19,7,-0.0026746

2,2,16,19,8,-0.030422

2,2,16,20,1,0.010415

2,2,16,20,2,0.012207

2,2,16,20,3,-0.047649

2,2,16,20,4,-0.010497

2,2,16,20,5,-0.017022

2,2,16,20,6,-0.017944

2,2,16,20,7,-0.0078099

2,2,16,20,8,0.014741

2,2,17,17,1,0.1448

2,2,17,17,2,0.003948

2,2,17,17,3,-0.036276

2,2,17,17,4,-0.038922

2,2,17,17,5,-0.024523

2,2,17,17,6,-0.032628

2,2,17,17,7,-0.015487

2,2,17,17,8,-0.010851

2,2,17,18,1,0.070564

2,2,17,18,2,0.010899

2,2,17,18,3,-0.070033

2,2,17,18,4,-0.044627

2,2,17,18,5,-0.046718

2,2,17,18,6,-0.035743

2,2,17,18,7,-0.024238

2,2,17,18,8,-0.0069855

2,2,17,19,1,0.054721

2,2,17,19,2,0.0017459

2,2,17,19,3,-0.064435

2,2,17,19,4,-0.038711

2,2,17,19,5,-0.043448

2,2,17,19,6,-0.035784

2,2,17,19,7,-0.023561

2,2,17,19,8,0.00047777

2,2,17,20,1,0.02479

2,2,17,20,2,-0.010731

2,2,17,20,3,-0.042535

2,2,17,20,4,-0.041716

2,2,17,20,5,-0.026338

2,2,17,20,6,-0.041813

2,2,17,20,7,-0.015903

2,2,17,20,8,-0.0016516

2,2,18,18,1,0.049018

2,2,18,18,2,-0.020014

2,2,18,18,3,-0.036662

2,2,18,18,4,-0.024088

2,2,18,18,5,-0.024079

2,2,18,18,6,-0.016074

2,2,18,18,7,-0.014403

2,2,18,18,8,0.010082

2,2,18,19,1,0.03705

2,2,18,19,2,0.0050099

2,2,18,19,3,-0.050616

2,2,18,19,4,-0.052142

2,2,18,19,5,-0.034683

2,2,18,19,6,-0.04832

2,2,18,19,7,-0.022972

2,2,18,19,8,0.003871

2,2,18,20,1,0.009124

2,2,18,20,2,-0.00037631

2,2,18,20,3,-0.055267

2,2,18,20,4,-0.034891

2,2,18,20,5,-0.031464

2,2,18,20,6,-0.021561

2,2,18,20,7,-0.0068261

2,2,18,20,8,0.01949

2,2,19,19,1,0.02964

2,2,19,19,2,-0.030362

2,2,19,19,3,-0.046177

2,2,19,19,4,-0.036977

2,2,19,19,5,-0.028779

2,2,19,19,6,-0.019374

2,2,19,19,7,-0.0061596

2,2,19,19,8,0.0031046

2,2,19,20,1,0.012669

2,2,19,20,2,-0.0025903

2,2,19,20,3,-0.040918

2,2,19,20,4,-0.025289

2,2,19,20,5,-0.0091807

2,2,19,20,6,-0.0061215

2,2,19,20,7,-0.010212

2,2,19,20,8,0.011538

2,2,20,20,1,0.012434

2,2,20,20,2,-0.014073

2,2,20,20,3,-0.028678

2,2,20,20,4,-0.017053

2,2,20,20,5,-0.012802

2,2,20,20,6,0.0045198

2,2,20,20,7,-0.0040532

2,2,20,20,8,0.0034798

2,3,1,1,1,0.0057132

2,3,1,1,2,-0.0021031

2,3,1,1,3,-0.00036653

2,3,1,1,4,-0.001414

2,3,1,1,5,0.00048643

2,3,1,1,6,0.00014046

2,3,1,1,7,0.00018465

2,3,1,1,8,-0.00067176

2,3,1,2,1,1.5014e-09

2,3,1,2,2,-0.00087671

2,3,1,2,3,-0.0015991

2,3,1,2,4,0.00074349

2,3,1,2,5,0.00043201

2,3,1,2,6,-0.00030331

2,3,1,2,7,-0.00094295

2,3,1,2,8,-0.00012263

2,3,1,3,1,0.0046663

2,3,1,3,2,-8.6473e-05

2,3,1,3,3,-0.0074798

2,3,1,3,4,-0.0019631

2,3,1,3,5,-4.7775e-05

2,3,1,3,6,-4.8712e-05

2,3,1,3,7,-0.0009393

2,3,1,3,8,-0.0011338

2,3,1,4,1,0.0044597

2,3,1,4,2,0.0015809

2,3,1,4,3,-0.0041364

2,3,1,4,4,-0.00024721

2,3,1,4,5,-4.2988e-05

2,3,1,4,6,0.0015536

2,3,1,4,7,-0.0014571

2,3,1,4,8,0.00094629

2,3,1,5,1,0.0065037

2,3,1,5,2,-0.0019846

2,3,1,5,3,-0.0015649

2,3,1,5,4,-0.0020845

2,3,1,5,5,0.00061652

2,3,1,5,6,-0.00034407

2,3,1,5,7,-0.0016136

2,3,1,5,8,0.00064442

2,3,1,6,1,0.0030336

2,3,1,6,2,-0.001214

2,3,1,6,3,-0.0033421

2,3,1,6,4,-0.0028421

2,3,1,6,5,0.0016379

2,3,1,6,6,-0.00047588

2,3,1,6,7,-0.00083643

2,3,1,6,8,-0.00073188

2,3,1,7,1,0.0024638

2,3,1,7,2,5.326e-06

2,3,1,7,3,-0.0042973

2,3,1,7,4,-0.0034726

2,3,1,7,5,0.00059271

2,3,1,7,6,0.00034943

2,3,1,7,7,-0.0010333

2,3,1,7,8,0.00053659

2,3,1,8,1,0.0061847

2,3,1,8,2,-0.00093588

2,3,1,8,3,-0.0028796

2,3,1,8,4,-0.00025663

2,3,1,8,5,0.00038941

2,3,1,8,6,0.00015342

2,3,1,8,7,-0.001638

2,3,1,8,8,-0.00045092

2,3,1,9,1,0.0038632

2,3,1,9,2,-0.00021874

2,3,1,9,3,-0.0047847

2,3,1,9,4,-0.00092921

2,3,1,9,5,-0.00012309

2,3,1,9,6,0.00071354

2,3,1,9,7,-0.0020963

2,3,1,9,8,0.0010899

2,3,1,10,1,0.0053542

2,3,1,10,2,-0.0021771

2,3,1,10,3,-0.0051968

2,3,1,10,4,-0.001586

2,3,1,10,5,-0.00072094

2,3,1,10,6,0.00041315

2,3,1,10,7,-0.00050648

2,3,1,10,8,-9.2838e-05

2,3,1,11,1,0.0070605

2,3,1,11,2,0.0011809

2,3,1,11,3,-0.0038034

2,3,1,11,4,-0.0044182

2,3,1,11,5,0.00017827

2,3,1,11,6,-0.0004367

2,3,1,11,7,-0.0017154

2,3,1,11,8,7.9916e-05

2,3,1,12,1,0.0014194

2,3,1,12,2,-0.0025992

2,3,1,12,3,-0.0051622

2,3,1,12,4,-0.0024364

2,3,1,12,5,0.00044961

2,3,1,12,6,0.0012903

2,3,1,12,7,0.0008676

2,3,1,12,8,-0.00014761

2,3,1,13,1,0.008246

2,3,1,13,2,0.0009023

2,3,1,13,3,-0.0015791

2,3,1,13,4,0.00056903

2,3,1,13,5,0.00088687

2,3,1,13,6,0.0030139

2,3,1,13,7,-0.00078546

2,3,1,13,8,0.0015261

2,3,1,14,1,0.0076264

2,3,1,14,2,0.000334

2,3,1,14,3,-0.0034577

2,3,1,14,4,4.2287e-05

2,3,1,14,5,0.00028067

2,3,1,14,6,0.0021604

2,3,1,14,7,-0.0019429

2,3,1,14,8,0.0018272

2,3,1,15,1,0.0039384

2,3,1,15,2,0.00072663

2,3,1,15,3,-0.0035592

2,3,1,15,4,0.00026685

2,3,1,15,5,-0.0014624

2,3,1,15,6,0.0016132

2,3,1,15,7,-0.00043741

2,3,1,15,8,0.00081933

2,3,1,16,1,0.004283

2,3,1,16,2,0.0036332

2,3,1,16,3,-0.0017624

2,3,1,16,4,-0.00065903

2,3,1,16,5,0.0011096

2,3,1,16,6,0.00092431

2,3,1,16,7,-0.0018983

2,3,1,16,8,-0.0016352

2,3,1,17,1,0.011019

2,3,1,17,2,0.0025256

2,3,1,17,3,-0.0019992

2,3,1,17,4,-0.0011294

2,3,1,17,5,-0.00018485

2,3,1,17,6,0.0004969

2,3,1,17,7,-0.0015855

2,3,1,17,8,0.00034677

2,3,1,18,1,0.0047698

2,3,1,18,2,0.0021709

2,3,1,18,3,-0.0043592

2,3,1,18,4,-0.0016279

2,3,1,18,5,0.00034346

2,3,1,18,6,0.00054177

2,3,1,18,7,-0.0011938

2,3,1,18,8,0.0011589

2,3,1,19,1,0.005483

2,3,1,19,2,0.0039026

2,3,1,19,3,-0.0023715

2,3,1,19,4,-0.0016001

2,3,1,19,5,-0.0001368

2,3,1,19,6,0.001431

2,3,1,19,7,-0.00065099

2,3,1,19,8,0.0014959

2,3,1,20,1,0.0016166

2,3,1,20,2,0.001346

2,3,1,20,3,-0.0022252

2,3,1,20,4,-0.00037769

2,3,1,20,5,-0.0011785

2,3,1,20,6,0.00016658

2,3,1,20,7,-0.0013675

2,3,1,20,8,-0.00017521

2,3,2,2,1,0.00068491

2,3,2,2,2,-0.0021521

2,3,2,2,3,-0.00021014

2,3,2,2,4,-9.537e-05

2,3,2,2,5,-0.0010533

2,3,2,2,6,-0.0014091

2,3,2,2,7,-0.0017527

2,3,2,2,8,0.00255

2,3,2,3,1,0.00052797

2,3,2,3,2,0.0027582

2,3,2,3,3,-0.003605

2,3,2,3,4,-0.0011854

2,3,2,3,5,-0.0013671

2,3,2,3,6,-0.001028

2,3,2,3,7,-0.0027493

2,3,2,3,8,7.761e-05

2,3,2,4,1,8.4181e-12

2,3,2,4,2,0.0028746

2,3,2,4,3,-0.0039335

2,3,2,4,4,-0.0017352

2,3,2,4,5,-0.002764

2,3,2,4,6,-0.0032926

2,3,2,4,7,-0.0030688

2,3,2,4,8,0.0026722

2,3,2,5,1,1.1116e-11

2,3,2,5,2,0.0001958

2,3,2,5,3,-0.0019529

2,3,2,5,4,-0.00093718

2,3,2,5,5,-0.00105

2,3,2,5,6,-0.0012545

2,3,2,5,7,-0.0026786

2,3,2,5,8,0.0039289

2,3,2,6,1,0.00028795

2,3,2,6,2,-0.0023551

2,3,2,6,3,-0.0045169

2,3,2,6,4,0.00030533

2,3,2,6,5,-0.0012771

2,3,2,6,6,0.00056381

2,3,2,6,7,-0.0015975

2,3,2,6,8,0.0016124

2,3,2,7,1,0.00042801

2,3,2,7,2,0.0022534

2,3,2,7,3,-0.0017435

2,3,2,7,4,-0.0038464

2,3,2,7,5,-0.0025668

2,3,2,7,6,-0.0015964

2,3,2,7,7,-0.0031232

2,3,2,7,8,0.0010424

2,3,2,8,1,0.0019723

2,3,2,8,2,-0.00026656

2,3,2,8,3,-0.0034932

2,3,2,8,4,-0.00098237

2,3,2,8,5,-0.0016951

2,3,2,8,6,-0.00091328

2,3,2,8,7,-0.0014998

2,3,2,8,8,0.0029652

2,3,2,9,1,4.6687e-08

2,3,2,9,2,0.00036961

2,3,2,9,3,-0.0040056

2,3,2,9,4,0.00012079

2,3,2,9,5,-0.0039125

2,3,2,9,6,-0.00032135

2,3,2,9,7,-0.0033892

2,3,2,9,8,0.0027861

2,3,2,10,1,3.5643e-12

2,3,2,10,2,-0.00019349

2,3,2,10,3,-0.0009723

2,3,2,10,4,-0.0032865

2,3,2,10,5,0.00053706

2,3,2,10,6,-0.0028108

2,3,2,10,7,-0.001241

2,3,2,10,8,0.0003529

2,3,2,11,1,0.0016676

2,3,2,11,2,-0.0040321

2,3,2,11,3,-0.0029283

2,3,2,11,4,-0.0022236

2,3,2,11,5,-0.00075458

2,3,2,11,6,-0.00070074

2,3,2,11,7,-0.00033752

2,3,2,11,8,0.0023594

2,3,2,12,1,0.00058716

2,3,2,12,2,0.00010038

2,3,2,12,3,-0.0018147

2,3,2,12,4,-0.0046093

2,3,2,12,5,-0.0014095

2,3,2,12,6,-0.00060571

2,3,2,12,7,-0.0023963

2,3,2,12,8,0.0033874

2,3,2,13,1,0.0012085

2,3,2,13,2,-0.0047256

2,3,2,13,3,-0.003478

2,3,2,13,4,-0.0030851

2,3,2,13,5,-0.0017872

2,3,2,13,6,-0.0011393

2,3,2,13,7,-0.0027515

2,3,2,13,8,0.00424

2,3,2,14,1,4.1265e-10

2,3,2,14,2,-0.0022801

2,3,2,14,3,-0.0040646

2,3,2,14,4,-0.0023628

2,3,2,14,5,-0.0035473

2,3,2,14,6,0.00010313

2,3,2,14,7,-0.0040498

2,3,2,14,8,0.0047231

2,3,2,15,1,0.0006331

2,3,2,15,2,-0.00072113

2,3,2,15,3,-0.0029702

2,3,2,15,4,-0.003117

2,3,2,15,5,-0.0016063

2,3,2,15,6,-0.0014819

2,3,2,15,7,-0.0040081

2,3,2,15,8,0.0021647

2,3,2,16,1,0.00029914

2,3,2,16,2,-0.0004236

2,3,2,16,3,-0.0021169

2,3,2,16,4,0.0015374

2,3,2,16,5,-0.0012165

2,3,2,16,6,-0.00020696

2,3,2,16,7,-0.0020549

2,3,2,16,8,0.0025019

2,3,2,17,1,0.00064162

2,3,2,17,2,-0.00022313

2,3,2,17,3,-0.00078173

2,3,2,17,4,-0.0020319

2,3,2,17,5,-0.0017662

2,3,2,17,6,-0.0023852

2,3,2,17,7,-0.0031765

2,3,2,17,8,0.0038554

2,3,2,18,1,2.0955e-10

2,3,2,18,2,-0.00060243

2,3,2,18,3,-0.0015163

2,3,2,18,4,-0.0026658

2,3,2,18,5,-0.0030431

2,3,2,18,6,-0.0018459

2,3,2,18,7,-0.0036622

2,3,2,18,8,0.0043178

2,3,2,19,1,1.0047e-12

2,3,2,19,2,0.00046721

2,3,2,19,3,-0.0016672

2,3,2,19,4,0.00047911

2,3,2,19,5,-0.00078863

2,3,2,19,6,-0.0015059

2,3,2,19,7,-0.0017643

2,3,2,19,8,0.0036564

2,3,2,20,1,1.5725e-12

2,3,2,20,2,0.00147

2,3,2,20,3,-0.0016542

2,3,2,20,4,-0.0022309

2,3,2,20,5,-0.0023377

2,3,2,20,6,-0.0015859

2,3,2,20,7,-0.002893

2,3,2,20,8,0.0038572

2,3,3,3,1,0.0027882

2,3,3,3,2,-0.00099299

2,3,3,3,3,-0.0039533

2,3,3,3,4,-0.0017691

2,3,3,3,5,-0.00074065

2,3,3,3,6,-0.00050556

2,3,3,3,7,-0.0018045

2,3,3,3,8,-0.00041391

2,3,3,4,1,0.0064831

2,3,3,4,2,-0.0011004

2,3,3,4,3,-0.004891

2,3,3,4,4,-0.0023062

2,3,3,4,5,5.0544e-05

2,3,3,4,6,-0.00097113

2,3,3,4,7,-0.0011694

2,3,3,4,8,-5.2415e-05

2,3,3,5,1,0.0054589

2,3,3,5,2,0.00017519

2,3,3,5,3,-0.0042662

2,3,3,5,4,-0.0019817

2,3,3,5,5,-0.0014157

2,3,3,5,6,0.00083747

2,3,3,5,7,-0.0024448

2,3,3,5,8,0.0011504

2,3,3,6,1,0.0039026

2,3,3,6,2,-0.00010605

2,3,3,6,3,-0.0073939

2,3,3,6,4,-0.0034799

2,3,3,6,5,-0.00058796

2,3,3,6,6,0.0004214

2,3,3,6,7,-0.0016997

2,3,3,6,8,0.00081123

2,3,3,7,1,0.0039178

2,3,3,7,2,-0.00072539

2,3,3,7,3,-0.002618

2,3,3,7,4,-0.0031308

2,3,3,7,5,0.0015634

2,3,3,7,6,0.0012886

2,3,3,7,7,-0.00090728

2,3,3,7,8,0.00061012

2,3,3,8,1,0.0063693

2,3,3,8,2,-0.0018351

2,3,3,8,3,-0.0091008

2,3,3,8,4,-0.0020607

2,3,3,8,5,-0.00079374

2,3,3,8,6,-0.0012041

2,3,3,8,7,-0.001058

2,3,3,8,8,-0.00033309

2,3,3,9,1,0.0053404

2,3,3,9,2,0.00080351

2,3,3,9,3,-0.005433

2,3,3,9,4,-0.0029223

2,3,3,9,5,-0.0011201

2,3,3,9,6,-0.0014745

2,3,3,9,7,-0.0014458

2,3,3,9,8,-0.00021446

2,3,3,10,1,0.0050612

2,3,3,10,2,-0.0044249

2,3,3,10,3,-0.0089462

2,3,3,10,4,-0.0049664

2,3,3,10,5,-0.0017821

2,3,3,10,6,-0.0024706

2,3,3,10,7,-0.0010442

2,3,3,10,8,-6.0609e-05

2,3,3,11,1,0.0036436

2,3,3,11,2,-0.0023971

2,3,3,11,3,-0.0059906

2,3,3,11,4,-0.0025582

2,3,3,11,5,1.1912e-05

2,3,3,11,6,-0.0014498

2,3,3,11,7,-0.0013581

2,3,3,11,8,0.0010382

2,3,3,12,1,0.0026824

2,3,3,12,2,-0.00010706

2,3,3,12,3,-0.0054396

2,3,3,12,4,-0.003791

2,3,3,12,5,-0.0014872

2,3,3,12,6,-0.0013952

2,3,3,12,7,-0.0026788

2,3,3,12,8,-0.00040055

2,3,3,13,1,0.0054125

2,3,3,13,2,0.0020979

2,3,3,13,3,-0.0012468

2,3,3,13,4,-0.0025402

2,3,3,13,5,0.00034837

2,3,3,13,6,7.4072e-05

2,3,3,13,7,-0.0015011

2,3,3,13,8,0.0015965

2,3,3,14,1,0.0056372

2,3,3,14,2,0.0027798

2,3,3,14,3,-0.0034247

2,3,3,14,4,-0.002206

2,3,3,14,5,0.00043201

2,3,3,14,6,4.3855e-05

2,3,3,14,7,-0.0017074

2,3,3,14,8,0.0020116

2,3,3,15,1,0.0015738

2,3,3,15,2,-0.00075921

2,3,3,15,3,-0.0035416

2,3,3,15,4,-0.003374

2,3,3,15,5,0.0013692

2,3,3,15,6,-0.002833

2,3,3,15,7,-0.00096554

2,3,3,15,8,0.00017461

2,3,3,16,1,0.0019178

2,3,3,16,2,0.0011688

2,3,3,16,3,-0.0046913

2,3,3,16,4,-0.00078702

2,3,3,16,5,0.0004823

2,3,3,16,6,-0.00035292

2,3,3,16,7,-0.0018065

2,3,3,16,8,0.0014181

2,3,3,17,1,0.0080938

2,3,3,17,2,0.0028983

2,3,3,17,3,-0.0041031

2,3,3,17,4,-0.0015769

2,3,3,17,5,-0.00088907

2,3,3,17,6,-0.00024102

2,3,3,17,7,-0.0016025

2,3,3,17,8,2.5395e-05

2,3,3,18,1,0.0033286

2,3,3,18,2,0.00050777

2,3,3,18,3,-0.0045335

2,3,3,18,4,-0.0040792

2,3,3,18,5,-2.8975e-05

2,3,3,18,6,-0.0010548

2,3,3,18,7,-0.0019975

2,3,3,18,8,0.0018416

2,3,3,19,1,0.0022729

2,3,3,19,2,-0.001219

2,3,3,19,3,-0.00046249

2,3,3,19,4,-0.0019742

2,3,3,19,5,0.00057679

2,3,3,19,6,0.00014174

2,3,3,19,7,-0.0023011

2,3,3,19,8,0.001174

2,3,3,20,1,0.00162

2,3,3,20,2,-0.0006293

2,3,3,20,3,-0.002583

2,3,3,20,4,-0.004127

2,3,3,20,5,-0.00032792

2,3,3,20,6,-0.0012939

2,3,3,20,7,-0.001201

2,3,3,20,8,0.00011739

2,3,4,4,1,0.0053135

2,3,4,4,2,-0.0013126

2,3,4,4,3,0.00014384

2,3,4,4,4,-0.00079284

2,3,4,4,5,-0.00025109

2,3,4,4,6,-0.00032719

2,3,4,4,7,-0.0028685

2,3,4,4,8,0.0028319

2,3,4,5,1,0.0070292

2,3,4,5,2,0.0020869

2,3,4,5,3,-0.0025753

2,3,4,5,4,-0.0037689

2,3,4,5,5,-0.00070642

2,3,4,5,6,-0.0021557

2,3,4,5,7,-0.0035126

2,3,4,5,8,0.0027984

2,3,4,6,1,0.0052198

2,3,4,6,2,-0.0010562

2,3,4,6,3,-0.0050526

2,3,4,6,4,-0.0038639

2,3,4,6,5,-0.0017039

2,3,4,6,6,-0.0017397

2,3,4,6,7,-0.0028994

2,3,4,6,8,0.00011929

2,3,4,7,1,0.0035446

2,3,4,7,2,0.0011558

2,3,4,7,3,-0.0032131

2,3,4,7,4,-0.0032054

2,3,4,7,5,-0.00095461

2,3,4,7,6,-0.0010263

2,3,4,7,7,-0.0021644

2,3,4,7,8,-0.00033948

2,3,4,8,1,0.0040503

2,3,4,8,2,-0.0017336

2,3,4,8,3,-0.0046015

2,3,4,8,4,-0.0011126

2,3,4,8,5,-0.00018911

2,3,4,8,6,0.0010195

2,3,4,8,7,-0.00046518

2,3,4,8,8,0.0030218

2,3,4,9,1,0.0031032

2,3,4,9,2,-0.0019351

2,3,4,9,3,-0.0029373

2,3,4,9,4,-0.0012472

2,3,4,9,5,-0.0018645

2,3,4,9,6,-0.0014625

2,3,4,9,7,-0.0037384

2,3,4,9,8,0.0031083

2,3,4,10,1,0.0046345

2,3,4,10,2,-0.0045404

2,3,4,10,3,-0.0047916

2,3,4,10,4,-0.0044996

2,3,4,10,5,-0.0020105

2,3,4,10,6,-0.0017938

2,3,4,10,7,-0.0020187

2,3,4,10,8,0.001515

2,3,4,11,1,0.00047353

2,3,4,11,2,-0.00061422

2,3,4,11,3,-0.0055322

2,3,4,11,4,-0.0036965

2,3,4,11,5,-0.0021181

2,3,4,11,6,-0.001018

2,3,4,11,7,-0.0021363

2,3,4,11,8,0.0018204

2,3,4,12,1,0.0024169

2,3,4,12,2,6.6165e-05

2,3,4,12,3,-0.0015787

2,3,4,12,4,-0.0037628

2,3,4,12,5,0.0010462

2,3,4,12,6,-0.0028217

2,3,4,12,7,0.00011506

2,3,4,12,8,0.0010937

2,3,4,13,1,0.0048066

2,3,4,13,2,-0.002816

2,3,4,13,3,-0.0035897

2,3,4,13,4,-0.0045986

2,3,4,13,5,-0.0012591

2,3,4,13,6,-0.00053973

2,3,4,13,7,-0.0028116

2,3,4,13,8,0.0049757

2,3,4,14,1,0.0054522

2,3,4,14,2,-0.00084991

2,3,4,14,3,-0.0033382

2,3,4,14,4,-0.0021513

2,3,4,14,5,-0.0013699

2,3,4,14,6,-0.00083552

2,3,4,14,7,-0.004514

2,3,4,14,8,0.0046906

2,3,4,15,1,0.0019916

2,3,4,15,2,-0.00017347

2,3,4,15,3,-0.0013278

2,3,4,15,4,-0.0035893

2,3,4,15,5,-0.0016745

2,3,4,15,6,-0.0027272

2,3,4,15,7,-0.0042017

2,3,4,15,8,0.0036918

2,3,4,16,1,0.00043502

2,3,4,16,2,0.00059507

2,3,4,16,3,0.0019414

2,3,4,16,4,0.0018309

2,3,4,16,5,0.00098686

2,3,4,16,6,-0.00052884

2,3,4,16,7,-0.00025205

2,3,4,16,8,0.00016225

2,3,4,17,1,0.0091928

2,3,4,17,2,0.0014665

2,3,4,17,3,-0.00093281

2,3,4,17,4,-0.0022333

2,3,4,17,5,-0.0010136

2,3,4,17,6,-0.0021406

2,3,4,17,7,-0.0041795

2,3,4,17,8,0.0036708

2,3,4,18,1,0.0038094

2,3,4,18,2,-0.0014617

2,3,4,18,3,-0.0022501

2,3,4,18,4,-0.0032628

2,3,4,18,5,-0.00090831

2,3,4,18,6,-0.0026202

2,3,4,18,7,-0.0036593

2,3,4,18,8,0.0036594

2,3,4,19,1,0.0030313

2,3,4,19,2,0.001292

2,3,4,19,3,-0.0025814

2,3,4,19,4,-0.0010566

2,3,4,19,5,-7.7363e-05

2,3,4,19,6,-0.0010625

2,3,4,19,7,-0.002037

2,3,4,19,8,0.0028339

2,3,4,20,1,0.0015149

2,3,4,20,2,-0.00081811

2,3,4,20,3,-0.00088523

2,3,4,20,4,-0.003236

2,3,4,20,5,0.00032646

2,3,4,20,6,-0.00030984

2,3,4,20,7,-0.0017552

2,3,4,20,8,0.0041729

2,3,5,5,1,0.0074881

2,3,5,5,2,0.00036654

2,3,5,5,3,-0.00098597

2,3,5,5,4,-0.0021084

2,3,5,5,5,-0.001301

2,3,5,5,6,-0.0017816

2,3,5,5,7,-0.0025673

2,3,5,5,8,0.001027

2,3,5,6,1,0.0045903

2,3,5,6,2,-0.00051235

2,3,5,6,3,-0.0053475

2,3,5,6,4,-0.0028448

2,3,5,6,5,-0.0013679

2,3,5,6,6,-0.0020417

2,3,5,6,7,-0.0022209

2,3,5,6,8,-0.0015198

2,3,5,7,1,0.0053747

2,3,5,7,2,-1.4612e-05

2,3,5,7,3,-0.0033213

2,3,5,7,4,-0.004297

2,3,5,7,5,-0.0015152

2,3,5,7,6,-0.00096903

2,3,5,7,7,-0.0005257

2,3,5,7,8,-0.00022611

2,3,5,8,1,0.0033093

2,3,5,8,2,-0.00015777

2,3,5,8,3,-0.0035387

2,3,5,8,4,-0.0032414

2,3,5,8,5,-0.00014509

2,3,5,8,6,-0.0014281

2,3,5,8,7,-0.0021912

2,3,5,8,8,-5.4592e-05

2,3,5,9,1,0.0052714

2,3,5,9,2,0.00031664

2,3,5,9,3,-0.0024634

2,3,5,9,4,-0.0015535

2,3,5,9,5,-0.00010689

2,3,5,9,6,-0.00081511

2,3,5,9,7,-0.0034331

2,3,5,9,8,0.0041907

2,3,5,10,1,0.0072456

2,3,5,10,2,0.0028805

2,3,5,10,3,-0.0040258

2,3,5,10,4,-0.003117

2,3,5,10,5,-0.00064461

2,3,5,10,6,-0.0015639

2,3,5,10,7,-0.002771

2,3,5,10,8,-4.7496e-05

2,3,5,11,1,0.0046526

2,3,5,11,2,0.0019531

2,3,5,11,3,-0.0049568

2,3,5,11,4,-0.0030771

2,3,5,11,5,-0.00047064

2,3,5,11,6,-0.0017089

2,3,5,11,7,-0.0013891

2,3,5,11,8,0.00041331

2,3,5,12,1,0.0025785

2,3,5,12,2,-4.2088e-05

2,3,5,12,3,-0.0044383

2,3,5,12,4,-0.0022859

2,3,5,12,5,0.00046954

2,3,5,12,6,-0.0023013

2,3,5,12,7,-0.0029594

2,3,5,12,8,0.0027433

2,3,5,13,1,0.0073251

2,3,5,13,2,0.0014809

2,3,5,13,3,-0.002332

2,3,5,13,4,-0.00226

2,3,5,13,5,-0.0017844

2,3,5,13,6,-0.00025988

2,3,5,13,7,-0.0028029

2,3,5,13,8,0.005019

2,3,5,14,1,0.0059537

2,3,5,14,2,-0.0054579

2,3,5,14,3,-0.0045372

2,3,5,14,4,-0.0042882

2,3,5,14,5,-0.0041614

2,3,5,14,6,-0.0030093

2,3,5,14,7,-0.0038438

2,3,5,14,8,0.0046791

2,3,5,15,1,0.0029221

2,3,5,15,2,-0.00078897

2,3,5,15,3,-0.0015508

2,3,5,15,4,-0.0042425

2,3,5,15,5,-0.0015174

2,3,5,15,6,-0.0023059

2,3,5,15,7,-0.0038254

2,3,5,15,8,0.0030096

2,3,5,16,1,0.0022711

2,3,5,16,2,0.00054019

2,3,5,16,3,0.00074536

2,3,5,16,4,0.0010236

2,3,5,16,5,0.00088612

2,3,5,16,6,-0.0022002

2,3,5,16,7,-0.0010853

2,3,5,16,8,0.00058311

2,3,5,17,1,0.010448

2,3,5,17,2,0.0033014

2,3,5,17,3,-0.0037209

2,3,5,17,4,-0.0052566

2,3,5,17,5,-0.0033898

2,3,5,17,6,-0.0039303

2,3,5,17,7,-0.004814

2,3,5,17,8,0.0024209

2,3,5,18,1,0.0028479

2,3,5,18,2,0.00090463

2,3,5,18,3,-0.0046799

2,3,5,18,4,-0.0052611

2,3,5,18,5,-0.0039512

2,3,5,18,6,-0.0048486

2,3,5,18,7,-0.0045387

2,3,5,18,8,0.0025165

2,3,5,19,1,0.0018954

2,3,5,19,2,0.0018137

2,3,5,19,3,-0.0025232

2,3,5,19,4,-0.0049858

2,3,5,19,5,-0.00028303

2,3,5,19,6,-0.0031277

2,3,5,19,7,-0.0049573

2,3,5,19,8,0.0021848

2,3,5,20,1,0.0013987

2,3,5,20,2,0.0012723

2,3,5,20,3,-0.003519

2,3,5,20,4,-0.0030004

2,3,5,20,5,-0.0046604

2,3,5,20,6,-0.0026398

2,3,5,20,7,-0.0060734

2,3,5,20,8,0.0044033

2,3,6,6,1,0.0046379

2,3,6,6,2,0.0014575

2,3,6,6,3,-0.0036206

2,3,6,6,4,-0.0014365

2,3,6,6,5,-0.00038032

2,3,6,6,6,-0.00025144

2,3,6,6,7,-0.00021801

2,3,6,6,8,-0.00023763

2,3,6,7,1,0.0034428

2,3,6,7,2,-0.00036076

2,3,6,7,3,-0.0069267

2,3,6,7,4,-0.0041108

2,3,6,7,5,0.00031524

2,3,6,7,6,-0.0014663

2,3,6,7,7,-0.0025071

2,3,6,7,8,0.00062468

2,3,6,8,1,0.0043086

2,3,6,8,2,-0.00014065

2,3,6,8,3,-0.0051341

2,3,6,8,4,-0.0015315

2,3,6,8,5,-0.00035826

2,3,6,8,6,-0.0001878

2,3,6,8,7,-8.457e-05

2,3,6,8,8,0.00099391

2,3,6,9,1,0.0045923

2,3,6,9,2,0.001906

2,3,6,9,3,-0.0036686

2,3,6,9,4,-0.00093404

2,3,6,9,5,-0.0019435

2,3,6,9,6,0.0019678

2,3,6,9,7,-0.0035165

2,3,6,9,8,0.0020953

2,3,6,10,1,0.0040018

2,3,6,10,2,-0.0023989

2,3,6,10,3,-0.0060499

2,3,6,10,4,-0.0039923

2,3,6,10,5,0.0017434

2,3,6,10,6,-0.0017726

2,3,6,10,7,-0.0012023

2,3,6,10,8,-0.00067394

2,3,6,11,1,0.0038628

2,3,6,11,2,-0.0024456

2,3,6,11,3,-0.0042949

2,3,6,11,4,-0.0034603

2,3,6,11,5,0.00077105

2,3,6,11,6,0.00018512

2,3,6,11,7,1.6241e-05

2,3,6,11,8,0.000661

2,3,6,12,1,0.0022216

2,3,6,12,2,0.0020006

2,3,6,12,3,-0.0016552

2,3,6,12,4,-0.0042318

2,3,6,12,5,-0.0011976

2,3,6,12,6,-0.0012371

2,3,6,12,7,-0.00090372

2,3,6,12,8,-9.5668e-06

2,3,6,13,1,0.006195

2,3,6,13,2,0.0019923

2,3,6,13,3,0.00035336

2,3,6,13,4,-0.0010805

2,3,6,13,5,0.00068971

2,3,6,13,6,0.0015619

2,3,6,13,7,0.00031156

2,3,6,13,8,0.0012296

2,3,6,14,1,0.0049794

2,3,6,14,2,0.0011515

2,3,6,14,3,-0.0018909

2,3,6,14,4,-0.0021221

2,3,6,14,5,-0.00096679

2,3,6,14,6,1.8089e-05

2,3,6,14,7,-0.0028357

2,3,6,14,8,0.00043273

2,3,6,15,1,0.0017442

2,3,6,15,2,-0.0001195

2,3,6,15,3,-0.0029327

2,3,6,15,4,-0.0018355

2,3,6,15,5,0.0015752

2,3,6,15,6,-0.00025319

2,3,6,15,7,-0.0039104

2,3,6,15,8,-0.00070655

2,3,6,16,1,0.00096313

2,3,6,16,2,0.0019332

2,3,6,16,3,-0.00061568

2,3,6,16,4,-0.00035067

2,3,6,16,5,0.00086172

2,3,6,16,6,-0.00022391

2,3,6,16,7,0.0010095

2,3,6,16,8,0.0011921

2,3,6,17,1,0.0047273

2,3,6,17,2,0.00038353

2,3,6,17,3,-0.0023754

2,3,6,17,4,-0.0030356

2,3,6,17,5,0.00030437

2,3,6,17,6,0.00048127

2,3,6,17,7,-0.001564

2,3,6,17,8,0.00027342

2,3,6,18,1,0.0028112

2,3,6,18,2,0.0028776

2,3,6,18,3,-0.004511

2,3,6,18,4,-0.003202

2,3,6,18,5,-0.00069666

2,3,6,18,6,-0.00012735

2,3,6,18,7,-0.0023156

2,3,6,18,8,0.0013227

2,3,6,19,1,0.0023631

2,3,6,19,2,-0.0010505

2,3,6,19,3,-0.0018365

2,3,6,19,4,-0.0026582

2,3,6,19,5,-0.00070768

2,3,6,19,6,-0.00042138

2,3,6,19,7,-0.00045995

2,3,6,19,8,0.00096561

2,3,6,20,1,0.0014739

2,3,6,20,2,5.6388e-05

2,3,6,20,3,-0.0023208

2,3,6,20,4,-0.0054969

2,3,6,20,5,-0.00037108

2,3,6,20,6,5.2016e-06

2,3,6,20,7,-0.0023925

2,3,6,20,8,0.0026319

2,3,7,7,1,0.0025034

2,3,7,7,2,0.0002406

2,3,7,7,3,-0.0028229

2,3,7,7,4,-0.0037238

2,3,7,7,5,-0.0017361

2,3,7,7,6,-0.00059091

2,3,7,7,7,-0.0024163

2,3,7,7,8,-0.0011465

2,3,7,8,1,0.0045564

2,3,7,8,2,-0.0012022

2,3,7,8,3,-0.0047914

2,3,7,8,4,-0.0038013

2,3,7,8,5,-0.00074881

2,3,7,8,6,-0.00064771

2,3,7,8,7,-0.0026853

2,3,7,8,8,0.00053171

2,3,7,9,1,0.00060797

2,3,7,9,2,-0.0024167

2,3,7,9,3,-0.004968

2,3,7,9,4,-0.0027488

2,3,7,9,5,-0.00052519

2,3,7,9,6,-0.00034791

2,3,7,9,7,-0.00090971

2,3,7,9,8,0.0017072

2,3,7,10,1,0.0036415

2,3,7,10,2,-0.00066791

2,3,7,10,3,-0.0065359

2,3,7,10,4,-0.0027062

2,3,7,10,5,-0.0014682

2,3,7,10,6,0.0016984

2,3,7,10,7,-0.0026924

2,3,7,10,8,0.00046716

2,3,7,11,1,0.0015348

2,3,7,11,2,-0.0018594

2,3,7,11,3,-0.0031784

2,3,7,11,4,-0.0032112

2,3,7,11,5,-0.0013898

2,3,7,11,6,0.00058389

2,3,7,11,7,0.00012583

2,3,7,11,8,0.0006712

2,3,7,12,1,0.00067684

2,3,7,12,2,-0.0013371

2,3,7,12,3,-0.0011752

2,3,7,12,4,-0.0039865

2,3,7,12,5,-0.0003302

2,3,7,12,6,0.00095333

2,3,7,12,7,-0.0035365

2,3,7,12,8,0.0014592

2,3,7,13,1,0.0052132

2,3,7,13,2,-0.0018073

2,3,7,13,3,-0.004983

2,3,7,13,4,-0.0029846

2,3,7,13,5,-0.00064072

2,3,7,13,6,0.001121

2,3,7,13,7,0.00036051

2,3,7,13,8,0.0019696

2,3,7,14,1,0.0039106

2,3,7,14,2,-0.0033773

2,3,7,14,3,-0.0036696

2,3,7,14,4,-0.0061628

2,3,7,14,5,-0.0016754

2,3,7,14,6,-0.00020405

2,3,7,14,7,-0.002105

2,3,7,14,8,0.001155

2,3,7,15,1,0.0016674

2,3,7,15,2,0.00096989

2,3,7,15,3,-0.0025008

2,3,7,15,4,-0.0025377

2,3,7,15,5,-0.0004693

2,3,7,15,6,-0.0011981

2,3,7,15,7,-0.0010095

2,3,7,15,8,0.0012266

2,3,7,16,1,0.0012031

2,3,7,16,2,-0.00075615

2,3,7,16,3,-0.0034571

2,3,7,16,4,-0.00055163

2,3,7,16,5,0.0002261

2,3,7,16,6,0.0023417

2,3,7,16,7,-0.0023741

2,3,7,16,8,0.0027381

2,3,7,17,1,0.0051492

2,3,7,17,2,-0.0012515

2,3,7,17,3,-0.0046198

2,3,7,17,4,-0.0039208

2,3,7,17,5,-0.0015652

2,3,7,17,6,-0.0012143

2,3,7,17,7,-0.0023287

2,3,7,17,8,0.0015384

2,3,7,18,1,0.00050513

2,3,7,18,2,-0.00093174

2,3,7,18,3,-0.0028392

2,3,7,18,4,-0.0036089

2,3,7,18,5,-0.0014078

2,3,7,18,6,0.00061272

2,3,7,18,7,-0.0020589

2,3,7,18,8,0.0048271

2,3,7,19,1,0.0028651

2,3,7,19,2,-0.00055927

2,3,7,19,3,-0.0064887

2,3,7,19,4,-0.0045518

2,3,7,19,5,-0.0010716

2,3,7,19,6,-0.0016426

2,3,7,19,7,-0.0028725

2,3,7,19,8,0.0014132

2,3,7,20,1,0.00053662

2,3,7,20,2,-0.0025998

2,3,7,20,3,-0.0020294

2,3,7,20,4,-0.0042911

2,3,7,20,5,0.00091397

2,3,7,20,6,0.0010624

2,3,7,20,7,0.00056071

2,3,7,20,8,0.0031939

2,3,8,8,1,0.0069318

2,3,8,8,2,0.0037561

2,3,8,8,3,-0.0014786

2,3,8,8,4,-0.000322

2,3,8,8,5,0.00039321

2,3,8,8,6,-0.00023718

2,3,8,8,7,0.00019955

2,3,8,8,8,-0.00068576

2,3,8,9,1,0.0059536

2,3,8,9,2,-0.0046623

2,3,8,9,3,-0.0025924

2,3,8,9,4,-0.00029214

2,3,8,9,5,0.0010644

2,3,8,9,6,-0.0009962

2,3,8,9,7,4.0863e-05

2,3,8,9,8,0.00056894

2,3,8,10,1,0.0055727

2,3,8,10,2,-0.0017672

2,3,8,10,3,-0.0053712

2,3,8,10,4,-0.0014806

2,3,8,10,5,-0.00058872

2,3,8,10,6,0.000588

2,3,8,10,7,-0.0017667

2,3,8,10,8,0.00042403

2,3,8,11,1,0.00483

2,3,8,11,2,-0.0027834

2,3,8,11,3,-0.0060743

2,3,8,11,4,-0.0048194

2,3,8,11,5,-0.00043732

2,3,8,11,6,-0.0015689

2,3,8,11,7,-0.00063224

2,3,8,11,8,0.00029497

2,3,8,12,1,0.0040554

2,3,8,12,2,0.0010887

2,3,8,12,3,-0.0039828

2,3,8,12,4,-0.0019505

2,3,8,12,5,-0.00023549

2,3,8,12,6,0.00101

2,3,8,12,7,-0.0011112

2,3,8,12,8,0.0016164

2,3,8,13,1,0.0061222

2,3,8,13,2,-0.0023486

2,3,8,13,3,-0.0045232

2,3,8,13,4,-0.0037035

2,3,8,13,5,-0.0012314

2,3,8,13,6,-0.00096214

2,3,8,13,7,-0.0029882

2,3,8,13,8,-9.4451e-05

2,3,8,14,1,0.004548

2,3,8,14,2,0.00018356

2,3,8,14,3,-0.0023681

2,3,8,14,4,-0.0028001

2,3,8,14,5,0.00042372

2,3,8,14,6,0.0012942

2,3,8,14,7,-0.0013152

2,3,8,14,8,0.002718

2,3,8,15,1,0.0021376

2,3,8,15,2,-0.0010537

2,3,8,15,3,-0.00543

2,3,8,15,4,-0.0031755

2,3,8,15,5,-0.0022096

2,3,8,15,6,-0.0018139

2,3,8,15,7,-0.0024151

2,3,8,15,8,0.0017407

2,3,8,16,1,0.0038319

2,3,8,16,2,0.0032709

2,3,8,16,3,-0.0034718

2,3,8,16,4,-0.0011347

2,3,8,16,5,0.00090301

2,3,8,16,6,-0.001993

2,3,8,16,7,-0.0014259

2,3,8,16,8,0.00038581

2,3,8,17,1,0.006398

2,3,8,17,2,0.002815

2,3,8,17,3,-0.0035809

2,3,8,17,4,-0.0021788

2,3,8,17,5,-4.0723e-05

2,3,8,17,6,-0.00051224

2,3,8,17,7,-0.0019636

2,3,8,17,8,0.0025152

2,3,8,18,1,0.0047329

2,3,8,18,2,-0.0011643

2,3,8,18,3,-0.0025358

2,3,8,18,4,-0.0041194

2,3,8,18,5,0.00040654

2,3,8,18,6,-0.0017369

2,3,8,18,7,-0.0018953

2,3,8,18,8,-0.00012079

2,3,8,19,1,0.0021298

2,3,8,19,2,-0.003233

2,3,8,19,3,-0.0038715

2,3,8,19,4,-0.0032947

2,3,8,19,5,6.4723e-06

2,3,8,19,6,0.00069433

2,3,8,19,7,-0.0013642

2,3,8,19,8,0.0017086

2,3,8,20,1,0.0016711

2,3,8,20,2,0.0021311

2,3,8,20,3,-0.0038766

2,3,8,20,4,-0.0047043

2,3,8,20,5,0.00083021

2,3,8,20,6,-0.0013186

2,3,8,20,7,-4.942e-05

2,3,8,20,8,-0.0014526

2,3,9,9,1,0.0038431

2,3,9,9,2,-0.004241

2,3,9,9,3,-0.00095465

2,3,9,9,4,-0.002298

2,3,9,9,5,-0.0023787

2,3,9,9,6,-0.0015192

2,3,9,9,7,-0.0019041

2,3,9,9,8,-3.7024e-05

2,3,9,10,1,0.0040074

2,3,9,10,2,-0.0016621

2,3,9,10,3,-0.0030085

2,3,9,10,4,-0.0020391

2,3,9,10,5,-2.5365e-05

2,3,9,10,6,0.00015917

2,3,9,10,7,-0.00076549

2,3,9,10,8,0.0019605

2,3,9,11,1,0.0048311

2,3,9,11,2,-0.001988

2,3,9,11,3,-0.0052341

2,3,9,11,4,-0.0032395

2,3,9,11,5,-0.0024584

2,3,9,11,6,0.00075957

2,3,9,11,7,-0.0028762

2,3,9,11,8,0.0027711

2,3,9,12,1,0.0022323

2,3,9,12,2,0.00016325

2,3,9,12,3,-0.0051009

2,3,9,12,4,-0.00014559

2,3,9,12,5,0.00056246

2,3,9,12,6,-0.0022688

2,3,9,12,7,-0.00063724

2,3,9,12,8,0.00053039

2,3,9,13,1,0.0055923

2,3,9,13,2,0.00087065

2,3,9,13,3,-0.0013829

2,3,9,13,4,-0.0013076

2,3,9,13,5,-0.00097407

2,3,9,13,6,-0.0012362

2,3,9,13,7,-0.004244

2,3,9,13,8,0.0039517

2,3,9,14,1,0.0042611

2,3,9,14,2,-0.0040911

2,3,9,14,3,-0.0048746

2,3,9,14,4,-0.0045165

2,3,9,14,5,-0.0020261

2,3,9,14,6,-0.0023069

2,3,9,14,7,-0.0047849

2,3,9,14,8,0.0055415

2,3,9,15,1,0.0015786

2,3,9,15,2,0.0022121

2,3,9,15,3,-0.001567

2,3,9,15,4,-0.0030269

2,3,9,15,5,-0.00025795

2,3,9,15,6,-0.00093267

2,3,9,15,7,-0.0031364

2,3,9,15,8,0.0025299

2,3,9,16,1,0.0033268

2,3,9,16,2,-0.0022439

2,3,9,16,3,-0.00094543

2,3,9,16,4,0.0025253

2,3,9,16,5,-0.0004912

2,3,9,16,6,0.0014253

2,3,9,16,7,-0.0015965

2,3,9,16,8,0.0042416

2,3,9,17,1,0.0097178

2,3,9,17,2,0.0031402

2,3,9,17,3,-0.0050742

2,3,9,17,4,-0.00479

2,3,9,17,5,-0.0035317

2,3,9,17,6,-0.0042732

2,3,9,17,7,-0.0052025

2,3,9,17,8,0.0027308

2,3,9,18,1,0.0024119

2,3,9,18,2,-0.00042886

2,3,9,18,3,-0.0033672

2,3,9,18,4,-0.0038924

2,3,9,18,5,-0.00049557

2,3,9,18,6,-0.0017859

2,3,9,18,7,-0.0027841

2,3,9,18,8,0.0033849

2,3,9,19,1,0.0018841

2,3,9,19,2,-4.531e-05

2,3,9,19,3,-0.003663

2,3,9,19,4,-0.0025432

2,3,9,19,5,0.00046502

2,3,9,19,6,-0.0014556

2,3,9,19,7,-0.0013469

2,3,9,19,8,0.0024515

2,3,9,20,1,0.0013007

2,3,9,20,2,0.00014104

2,3,9,20,3,-0.00084354

2,3,9,20,4,-0.0031872

2,3,9,20,5,-0.0005087

2,3,9,20,6,-0.0028309

2,3,9,20,7,-0.0029259

2,3,9,20,8,0.002443

2,3,10,10,1,0.0072372

2,3,10,10,2,-0.0063844

2,3,10,10,3,-0.0016823

2,3,10,10,4,-0.0014842

2,3,10,10,5,1.4557e-05

2,3,10,10,6,-0.00074307

2,3,10,10,7,-0.0008438

2,3,10,10,8,-0.00030225

2,3,10,11,1,0.0059384

2,3,10,11,2,0.0026538

2,3,10,11,3,-0.0013486

2,3,10,11,4,-0.0034177

2,3,10,11,5,0.00097402

2,3,10,11,6,-0.00054563

2,3,10,11,7,-0.0001109

2,3,10,11,8,0.0019039

2,3,10,12,1,0.001926

2,3,10,12,2,-0.0029956

2,3,10,12,3,-0.0053892

2,3,10,12,4,-0.0029861

2,3,10,12,5,-0.0014081

2,3,10,12,6,-0.0005774

2,3,10,12,7,-0.0012592

2,3,10,12,8,0.00079067

2,3,10,13,1,0.0056506

2,3,10,13,2,-0.00090093

2,3,10,13,3,-0.005127

2,3,10,13,4,-0.0030889

2,3,10,13,5,-0.0015403

2,3,10,13,6,-0.0012841

2,3,10,13,7,-0.0023864

2,3,10,13,8,-4.6552e-05

2,3,10,14,1,0.0055744

2,3,10,14,2,-6.7202e-05

2,3,10,14,3,-0.0051882

2,3,10,14,4,-0.0023256

2,3,10,14,5,-0.00098686

2,3,10,14,6,6.1986e-05

2,3,10,14,7,-0.00091204

2,3,10,14,8,0.001315

2,3,10,15,1,0.0026961

2,3,10,15,2,0.00077901

2,3,10,15,3,-0.0060683

2,3,10,15,4,-0.0044329

2,3,10,15,5,-0.00080986

2,3,10,15,6,-0.00060979

2,3,10,15,7,-0.0025123

2,3,10,15,8,0.0021138

2,3,10,16,1,0.002377

2,3,10,16,2,0.0010947

2,3,10,16,3,-0.0027139

2,3,10,16,4,0.00065322

2,3,10,16,5,0.0019432

2,3,10,16,6,0.0017407

2,3,10,16,7,0.0020824

2,3,10,16,8,0.0025174

2,3,10,17,1,0.0089115

2,3,10,17,2,0.0019035

2,3,10,17,3,-0.004964

2,3,10,17,4,-0.0022856

2,3,10,17,5,-0.00074788

2,3,10,17,6,7.3449e-05

2,3,10,17,7,-0.0011852

2,3,10,17,8,0.0023205

2,3,10,18,1,0.0030087

2,3,10,18,2,-0.00011782

2,3,10,18,3,-0.0029274

2,3,10,18,4,-0.0056754

2,3,10,18,5,-0.00032757

2,3,10,18,6,-0.0013643

2,3,10,18,7,-0.0020486

2,3,10,18,8,0.0011506

2,3,10,19,1,0.004255

2,3,10,19,2,-0.0031107

2,3,10,19,3,-0.0040869

2,3,10,19,4,-0.0024672

2,3,10,19,5,-0.00032268

2,3,10,19,6,-0.00094491

2,3,10,19,7,-0.00052733

2,3,10,19,8,0.0009866

2,3,10,20,1,0.0020723

2,3,10,20,2,-0.0045016

2,3,10,20,3,-0.0033465

2,3,10,20,4,-0.0044034

2,3,10,20,5,-0.00039893

2,3,10,20,6,-0.0012699

2,3,10,20,7,-0.0014218

2,3,10,20,8,0.003986

2,3,11,11,1,0.0061615

2,3,11,11,2,-0.0004263

2,3,11,11,3,-0.0027676

2,3,11,11,4,-0.0025216

2,3,11,11,5,-5.7894e-05

2,3,11,11,6,-0.00046413

2,3,11,11,7,-0.0013948

2,3,11,11,8,5.6218e-05

2,3,11,12,1,0.0020606

2,3,11,12,2,-0.0027319

2,3,11,12,3,-0.0044207

2,3,11,12,4,-0.0057685

2,3,11,12,5,-0.0015163

2,3,11,12,6,-0.0013281

2,3,11,12,7,-0.0022629

2,3,11,12,8,-0.0012222

2,3,11,13,1,0.0040152

2,3,11,13,2,-0.0039526

2,3,11,13,3,-0.003751

2,3,11,13,4,-0.0051951

2,3,11,13,5,-0.0018917

2,3,11,13,6,7.7104e-05

2,3,11,13,7,-0.0020823

2,3,11,13,8,0.0018493

2,3,11,14,1,0.0047249

2,3,11,14,2,-0.0012692

2,3,11,14,3,-0.0043947

2,3,11,14,4,-0.0034144

2,3,11,14,5,-0.00046513

2,3,11,14,6,0.0006657

2,3,11,14,7,-0.0020854

2,3,11,14,8,0.0026976

2,3,11,15,1,0.00057461

2,3,11,15,2,-0.0012922

2,3,11,15,3,-0.0033569

2,3,11,15,4,-0.0032968

2,3,11,15,5,-0.00012752

2,3,11,15,6,0.00045931

2,3,11,15,7,-0.0015608

2,3,11,15,8,0.0021115

2,3,11,16,1,0.0039306

2,3,11,16,2,-0.0010233

2,3,11,16,3,-0.00025748

2,3,11,16,4,-0.0024855

2,3,11,16,5,-1.9976e-05

2,3,11,16,6,-0.00019916

2,3,11,16,7,-0.00011213

2,3,11,16,8,0.0021309

2,3,11,17,1,0.0087885

2,3,11,17,2,0.0017697

2,3,11,17,3,-0.0067019

2,3,11,17,4,-0.0044984

2,3,11,17,5,-0.0023663

2,3,11,17,6,-0.0011715

2,3,11,17,7,-0.0028269

2,3,11,17,8,0.0016882

2,3,11,18,1,0.0025645

2,3,11,18,2,-0.001538

2,3,11,18,3,-0.0062443

2,3,11,18,4,-0.0049539

2,3,11,18,5,-0.0039369

2,3,11,18,6,-0.00064738

2,3,11,18,7,-0.0031887

2,3,11,18,8,-2.8748e-05

2,3,11,19,1,0.0029414

2,3,11,19,2,-0.00069823

2,3,11,19,3,-0.0051678

2,3,11,19,4,-0.0022449

2,3,11,19,5,-0.00059154

2,3,11,19,6,0.00045889

2,3,11,19,7,-0.0026417

2,3,11,19,8,0.00072047

2,3,11,20,1,0.00094771

2,3,11,20,2,-0.0034292

2,3,11,20,3,-0.0046549

2,3,11,20,4,-0.0047375

2,3,11,20,5,-0.0016705

2,3,11,20,6,0.0023303

2,3,11,20,7,-0.00041778

2,3,11,20,8,0.001107

2,3,12,12,1,0.0019793

2,3,12,12,2,-0.0013122

2,3,12,12,3,-0.0008192

2,3,12,12,4,-0.0023958

2,3,12,12,5,-0.001088

2,3,12,12,6,0.00042055

2,3,12,12,7,-0.00093676

2,3,12,12,8,0.00014292

2,3,12,13,1,0.00079197

2,3,12,13,2,-0.0035423

2,3,12,13,3,-0.0018662

2,3,12,13,4,-0.0040076

2,3,12,13,5,-0.0016806

2,3,12,13,6,-0.00073146

2,3,12,13,7,-0.0011343

2,3,12,13,8,0.0022997

2,3,12,14,1,0.0019567

2,3,12,14,2,0.0012217

2,3,12,14,3,-0.0068069

2,3,12,14,4,-0.003342

2,3,12,14,5,0.00060163

2,3,12,14,6,0.001856

2,3,12,14,7,-0.0012808

2,3,12,14,8,0.0023487

2,3,12,15,1,0.00097479

2,3,12,15,2,-0.0024393

2,3,12,15,3,-0.0042266

2,3,12,15,4,-0.0035545

2,3,12,15,5,-0.00088162

2,3,12,15,6,-0.001059

2,3,12,15,7,-0.0014729

2,3,12,15,8,0.001585

2,3,12,16,1,0.0014016

2,3,12,16,2,-0.0001646

2,3,12,16,3,-0.0032539

2,3,12,16,4,-0.0057315

2,3,12,16,5,0.0023211

2,3,12,16,6,-0.0016079

2,3,12,16,7,-0.0024185

2,3,12,16,8,0.0053432

2,3,12,17,1,0.0029688

2,3,12,17,2,0.0014867

2,3,12,17,3,-0.005503

2,3,12,17,4,-0.0051844

2,3,12,17,5,-0.0020781

2,3,12,17,6,-0.0018832

2,3,12,17,7,-0.0012024

2,3,12,17,8,0.002158

2,3,12,18,1,0.0018502

2,3,12,18,2,0.00011807

2,3,12,18,3,-0.0040744

2,3,12,18,4,-0.0043628

2,3,12,18,5,0.00013514

2,3,12,18,6,-0.00085491

2,3,12,18,7,-0.0013075

2,3,12,18,8,0.0016281

2,3,12,19,1,0.0014926

2,3,12,19,2,-0.00097596

2,3,12,19,3,-0.0049062

2,3,12,19,4,-0.0021773

2,3,12,19,5,0.00048991

2,3,12,19,6,-0.0013193

2,3,12,19,7,-0.0029826

2,3,12,19,8,0.0018332

2,3,12,20,1,0.0004448

2,3,12,20,2,0.0010668

2,3,12,20,3,-0.00015032

2,3,12,20,4,-0.0016048

2,3,12,20,5,0.00026471

2,3,12,20,6,-0.002993

2,3,12,20,7,-0.001943

2,3,12,20,8,-0.0018982

2,3,13,13,1,0.0084611

2,3,13,13,2,-0.0024011

2,3,13,13,3,-0.0026826

2,3,13,13,4,-0.0019545

2,3,13,13,5,-0.001587

2,3,13,13,6,-0.0015019

2,3,13,13,7,-0.0018287

2,3,13,13,8,0.0013036

2,3,13,14,1,0.006314

2,3,13,14,2,-0.0097099

2,3,13,14,3,-0.0079998

2,3,13,14,4,-0.0050067

2,3,13,14,5,-0.0049967

2,3,13,14,6,-0.0024153

2,3,13,14,7,-0.0050402

2,3,13,14,8,0.0048222

2,3,13,15,1,0.0014904

2,3,13,15,2,-0.0012842

2,3,13,15,3,-0.0060425

2,3,13,15,4,-0.0044371

2,3,13,15,5,-0.002439

2,3,13,15,6,-0.0022107

2,3,13,15,7,-0.0043414

2,3,13,15,8,0.0041736

2,3,13,16,1,0.0013867

2,3,13,16,2,-0.0031562

2,3,13,16,3,-0.0015423

2,3,13,16,4,-0.0026329

2,3,13,16,5,0.00065794

2,3,13,16,6,-0.00055555

2,3,13,16,7,-0.0023178

2,3,13,16,8,0.0050658

2,3,13,17,1,0.010725

2,3,13,17,2,-0.0034521

2,3,13,17,3,-0.0059788

2,3,13,17,4,-0.0069459

2,3,13,17,5,-0.0055821

2,3,13,17,6,-0.0040928

2,3,13,17,7,-0.0057592

2,3,13,17,8,0.0025839

2,3,13,18,1,0.0029632

2,3,13,18,2,-0.0042241

2,3,13,18,3,-0.0086829

2,3,13,18,4,-0.0056273

2,3,13,18,5,-0.0044563

2,3,13,18,6,-0.0040631

2,3,13,18,7,-0.0043308

2,3,13,18,8,0.0048928

2,3,13,19,1,0.0016287

2,3,13,19,2,-0.0029577

2,3,13,19,3,-0.0070635

2,3,13,19,4,-0.004327

2,3,13,19,5,-0.0030692

2,3,13,19,6,-0.0028702

2,3,13,19,7,-0.0038181

2,3,13,19,8,0.0037975

2,3,13,20,1,0.00088376

2,3,13,20,2,-0.0031153

2,3,13,20,3,-0.0032917

2,3,13,20,4,-0.0035335

2,3,13,20,5,-0.0012729

2,3,13,20,6,0.00057041

2,3,13,20,7,-0.0016644

2,3,13,20,8,0.0056553

2,3,14,14,1,0.005344

2,3,14,14,2,-0.0045679

2,3,14,14,3,-0.0056983

2,3,14,14,4,-0.0020712

2,3,14,14,5,-0.0035354

2,3,14,14,6,-0.0018848

2,3,14,14,7,-0.0020696

2,3,14,14,8,0.0019457

2,3,14,15,1,0.0016044

2,3,14,15,2,-0.0042932

2,3,14,15,3,-0.0063826

2,3,14,15,4,-0.0064444

2,3,14,15,5,-0.0041804

2,3,14,15,6,-0.0034591

2,3,14,15,7,-0.0013943

2,3,14,15,8,0.0049578

2,3,14,16,1,0.0023498

2,3,14,16,2,-0.0011524

2,3,14,16,3,-0.002359

2,3,14,16,4,0.00057103

2,3,14,16,5,-0.00049845

2,3,14,16,6,-0.001272

2,3,14,16,7,-6.1501e-05

2,3,14,16,8,0.0022194

2,3,14,17,1,0.006157

2,3,14,17,2,-0.0046411

2,3,14,17,3,-0.0087212

2,3,14,17,4,-0.0070026

2,3,14,17,5,-0.0045614

2,3,14,17,6,-0.0053732

2,3,14,17,7,-0.0046976

2,3,14,17,8,0.0028938

2,3,14,18,1,0.0026943

2,3,14,18,2,-0.0044304

2,3,14,18,3,-0.009661

2,3,14,18,4,-0.0050873

2,3,14,18,5,-0.0048368

2,3,14,18,6,-0.0031907

2,3,14,18,7,-0.0041324

2,3,14,18,8,0.0042922

2,3,14,19,1,0.0012714

2,3,14,19,2,-0.008422

2,3,14,19,3,-0.008215

2,3,14,19,4,-0.0059009

2,3,14,19,5,-0.0038236

2,3,14,19,6,-0.0030401

2,3,14,19,7,-0.0037541

2,3,14,19,8,0.0042126

2,3,14,20,1,0.0017125

2,3,14,20,2,-0.00066186

2,3,14,20,3,-0.0049028

2,3,14,20,4,-0.0037848

2,3,14,20,5,-0.00243

2,3,14,20,6,-0.0024882

2,3,14,20,7,-0.0040906

2,3,14,20,8,0.0035993

2,3,15,15,1,0.0013834

2,3,15,15,2,-0.0010138

2,3,15,15,3,-0.0014396

2,3,15,15,4,-0.00097745

2,3,15,15,5,-0.00034974

2,3,15,15,6,-0.0015126

2,3,15,15,7,-0.0029824

2,3,15,15,8,0.0030687

2,3,15,16,1,0.00053059

2,3,15,16,2,-2.2152e-05

2,3,15,16,3,-0.0011233

2,3,15,16,4,-0.00045496

2,3,15,16,5,0.00023234

2,3,15,16,6,-0.0024631

2,3,15,16,7,-0.0015945

2,3,15,16,8,0.002233

2,3,15,17,1,0.0024896

2,3,15,17,2,-0.0010891

2,3,15,17,3,-0.0072176

2,3,15,17,4,-0.0061972

2,3,15,17,5,-0.0045539

2,3,15,17,6,-0.0029561

2,3,15,17,7,-0.0048598

2,3,15,17,8,0.0050337

2,3,15,18,1,1.1338e-11

2,3,15,18,2,-0.0033117

2,3,15,18,3,-0.0020422

2,3,15,18,4,-0.0068904

2,3,15,18,5,-0.0045185

2,3,15,18,6,-0.0023929

2,3,15,18,7,-0.0046389

2,3,15,18,8,0.0060208

2,3,15,19,1,0.0010113

2,3,15,19,2,-0.00058256

2,3,15,19,3,-0.0037475

2,3,15,19,4,-0.0034086

2,3,15,19,5,-0.0015681

2,3,15,19,6,-0.0026127

2,3,15,19,7,-0.002979

2,3,15,19,8,0.0033043

2,3,15,20,1,3.3117e-06

2,3,15,20,2,-0.00038751

2,3,15,20,3,-0.0021684

2,3,15,20,4,-0.003399

2,3,15,20,5,-0.0027238

2,3,15,20,6,-0.0041247

2,3,15,20,7,-0.0017868

2,3,15,20,8,0.0043855

2,3,16,16,1,1.8198e-12

2,3,16,16,2,-0.0040313

2,3,16,16,3,0.0027169

2,3,16,16,4,-0.00021956

2,3,16,16,5,0.0038107

2,3,16,16,6,0.00082425

2,3,16,16,7,0.0027175

2,3,16,16,8,0.0024667

2,3,16,17,1,0.0032058

2,3,16,17,2,-0.0012803

2,3,16,17,3,-0.00077003

2,3,16,17,4,-0.0031669

2,3,16,17,5,0.00031946

2,3,16,17,6,-0.0003778

2,3,16,17,7,-0.00029796

2,3,16,17,8,0.0038697

2,3,16,18,1,0.0011859

2,3,16,18,2,0.00049315

2,3,16,18,3,-0.0010081

2,3,16,18,4,0.00064045

2,3,16,18,5,0.00019939

2,3,16,18,6,-0.0017441

2,3,16,18,7,-0.001035

2,3,16,18,8,0.0035275

2,3,16,19,1,0.001123

2,3,16,19,2,0.0012907

2,3,16,19,3,-0.0006874

2,3,16,19,4,0.0010528

2,3,16,19,5,0.0015761

2,3,16,19,6,-0.0020547

2,3,16,19,7,-0.0012107

2,3,16,19,8,0.0012845

2,3,16,20,1,0.00088491

2,3,16,20,2,0.0020128

2,3,16,20,3,-0.0048389

2,3,16,20,4,-0.00084244

2,3,16,20,5,0.00018976

2,3,16,20,6,-0.0012547

2,3,16,20,7,-0.00029264

2,3,16,20,8,0.0025847

2,3,17,17,1,0.012481

2,3,17,17,2,0.00070979

2,3,17,17,3,-0.002308

2,3,17,17,4,-0.0025799

2,3,17,17,5,-0.0024418

2,3,17,17,6,-0.0025818

2,3,17,17,7,-0.0026215

2,3,17,17,8,0.0014196

2,3,17,18,1,0.0046045

2,3,17,18,2,-0.0029864

2,3,17,18,3,-0.0082031

2,3,17,18,4,-0.006112

2,3,17,18,5,-0.0056049

2,3,17,18,6,-0.0052881

2,3,17,18,7,-0.0057126

2,3,17,18,8,0.0038137

2,3,17,19,1,0.0023898

2,3,17,19,2,-0.0031544

2,3,17,19,3,-0.0047848

2,3,17,19,4,-0.0041072

2,3,17,19,5,-0.0031415

2,3,17,19,6,-0.0027796

2,3,17,19,7,-0.0027668

2,3,17,19,8,0.0045556

2,3,17,20,1,0.0013797

2,3,17,20,2,-0.0041675

2,3,17,20,3,-0.0036441

2,3,17,20,4,-0.0044452

2,3,17,20,5,-0.003975

2,3,17,20,6,-0.0035866

2,3,17,20,7,-0.0026631

2,3,17,20,8,0.00385

2,3,18,18,1,0.0026654

2,3,18,18,2,-0.003167

2,3,18,18,3,-0.0049195

2,3,18,18,4,-0.0039003

2,3,18,18,5,-0.0039196

2,3,18,18,6,-0.0020382

2,3,18,18,7,-0.0027326

2,3,18,18,8,0.0023211

2,3,18,19,1,0.0020911

2,3,18,19,2,0.0010546

2,3,18,19,3,-0.0057271

2,3,18,19,4,-0.0052348

2,3,18,19,5,-0.00091558

2,3,18,19,6,-0.0035083

2,3,18,19,7,-0.0025111

2,3,18,19,8,0.0039608

2,3,18,20,1,0.00064007

2,3,18,20,2,-0.001995

2,3,18,20,3,-0.0078435

2,3,18,20,4,-0.0039816

2,3,18,20,5,-0.0033921

2,3,18,20,6,-0.0020368

2,3,18,20,7,-0.0031504

2,3,18,20,8,0.0048499

2,3,19,19,1,0.0024754

2,3,19,19,2,-0.00017048

2,3,19,19,3,-0.0027778

2,3,19,19,4,-0.0012779

2,3,19,19,5,-0.001489

2,3,19,19,6,-0.00096129

2,3,19,19,7,-0.00066067

2,3,19,19,8,0.0016127

2,3,19,20,1,0.00085106

2,3,19,20,2,-0.0022438

2,3,19,20,3,-0.0047187

2,3,19,20,4,-0.0041225

2,3,19,20,5,-0.0014893

2,3,19,20,6,-0.0020678

2,3,19,20,7,-0.00083917

2,3,19,20,8,0.0041909

2,3,20,20,1,0.0005742

2,3,20,20,2,-0.0021606

2,3,20,20,3,-0.0017971

2,3,20,20,4,-0.0013509

2,3,20,20,5,-0.0010963

2,3,20,20,6,0.00091119

2,3,20,20,7,-0.0011117

2,3,20,20,8,0.0011716

2,4,1,1,1,0.0045619

2,4,1,1,2,-0.0027341

2,4,1,1,3,-0.0016544

2,4,1,1,4,-0.0011173

2,4,1,1,5,0.00022254

2,4,1,1,6,-0.00027517

2,4,1,1,7,6.8295e-07

2,4,1,1,8,-0.00017954

2,4,1,2,1,3.4081e-09

2,4,1,2,2,-0.000312

2,4,1,2,3,-0.0025796

2,4,1,2,4,-5.661e-05

2,4,1,2,5,0.00055898

2,4,1,2,6,0.00056322

2,4,1,2,7,0.00015494

2,4,1,2,8,-0.00020776

2,4,1,3,1,0.0050468

2,4,1,3,2,0.00038954

2,4,1,3,3,-0.0068017

2,4,1,3,4,-0.0022778

2,4,1,3,5,0.00015133

2,4,1,3,6,-0.00029558

2,4,1,3,7,5.7672e-05

2,4,1,3,8,8.3759e-05

2,4,1,4,1,0.005142

2,4,1,4,2,0.00096665

2,4,1,4,3,-0.003431

2,4,1,4,4,-0.0013169

2,4,1,4,5,0.00042371

2,4,1,4,6,0.00030363

2,4,1,4,7,1.6313e-05

2,4,1,4,8,0.00047225

2,4,1,5,1,0.0073006

2,4,1,5,2,-0.00070102

2,4,1,5,3,-0.0018299

2,4,1,5,4,-0.0014649

2,4,1,5,5,0.0013219

2,4,1,5,6,0.00032992

2,4,1,5,7,0.00032435

2,4,1,5,8,0.0009612

2,4,1,6,1,0.0039232

2,4,1,6,2,-0.0013604

2,4,1,6,3,-0.0046118

2,4,1,6,4,-0.0015542

2,4,1,6,5,0.00087465

2,4,1,6,6,0.000597

2,4,1,6,7,-0.00017833

2,4,1,6,8,0.00011782

2,4,1,7,1,0.0024909

2,4,1,7,2,0.00060379

2,4,1,7,3,-0.004308

2,4,1,7,4,-0.0036273

2,4,1,7,5,0.00058492

2,4,1,7,6,-0.0012137

2,4,1,7,7,0.00045922

2,4,1,7,8,-7.3142e-05

2,4,1,8,1,0.0052524

2,4,1,8,2,-0.00064989

2,4,1,8,3,-0.0046648

2,4,1,8,4,-0.0010325

2,4,1,8,5,8.4779e-05

2,4,1,8,6,-0.00033633

2,4,1,8,7,-8.6603e-05

2,4,1,8,8,-9.2917e-05

2,4,1,9,1,0.0062979

2,4,1,9,2,-0.00056432

2,4,1,9,3,-0.0037797

2,4,1,9,4,-0.0011483

2,4,1,9,5,0.00052842

2,4,1,9,6,0.00054156

2,4,1,9,7,9.3107e-05

2,4,1,9,8,0.00069705

2,4,1,10,1,0.0051219

2,4,1,10,2,-0.001752

2,4,1,10,3,-0.0033946

2,4,1,10,4,-0.0025904

2,4,1,10,5,0.00041324

2,4,1,10,6,-0.00019362

2,4,1,10,7,0.00060532

2,4,1,10,8,0.00040774

2,4,1,11,1,0.0043897

2,4,1,11,2,1.7471e-06

2,4,1,11,3,-0.0041774

2,4,1,11,4,-0.0030059

2,4,1,11,5,0.00026844

2,4,1,11,6,-0.00021835

2,4,1,11,7,-0.00028695

2,4,1,11,8,-0.0001829

2,4,1,12,1,0.0020465

2,4,1,12,2,-0.0011208

2,4,1,12,3,-0.0034127

2,4,1,12,4,-0.0022879

2,4,1,12,5,0.0013134

2,4,1,12,6,0.00067414

2,4,1,12,7,0.0014561

2,4,1,12,8,7.6337e-05

2,4,1,13,1,0.0078791

2,4,1,13,2,0.0010735

2,4,1,13,3,-0.002405

2,4,1,13,4,0.00080167

2,4,1,13,5,0.0012798

2,4,1,13,6,0.0023475

2,4,1,13,7,0.00053695

2,4,1,13,8,0.00093312

2,4,1,14,1,0.0069238

2,4,1,14,2,0.0003984

2,4,1,14,3,-0.00342

2,4,1,14,4,-0.00022174

2,4,1,14,5,0.0010906

2,4,1,14,6,0.0010346

2,4,1,14,7,-6.9746e-05

2,4,1,14,8,0.00025772

2,4,1,15,1,0.0032028

2,4,1,15,2,0.00067559

2,4,1,15,3,-0.0021061

2,4,1,15,4,7.9526e-05

2,4,1,15,5,-0.00013039

2,4,1,15,6,0.0006213

2,4,1,15,7,0.00071457

2,4,1,15,8,0.00013992

2,4,1,16,1,0.0032763

2,4,1,16,2,0.00084605

2,4,1,16,3,-0.0028246

2,4,1,16,4,-0.00016602

2,4,1,16,5,0.00046981

2,4,1,16,6,0.00013861

2,4,1,16,7,-9.5586e-05

2,4,1,16,8,-0.00061899

2,4,1,17,1,0.0092042

2,4,1,17,2,0.0021667

2,4,1,17,3,-0.0025048

2,4,1,17,4,-0.00016354

2,4,1,17,5,0.00024532

2,4,1,17,6,0.0012907

2,4,1,17,7,0.00031995

2,4,1,17,8,0.00032174

2,4,1,18,1,0.0043224

2,4,1,18,2,0.0019387

2,4,1,18,3,-0.0038574

2,4,1,18,4,-0.00065389

2,4,1,18,5,0.00076869

2,4,1,18,6,0.00055761

2,4,1,18,7,0.00039841

2,4,1,18,8,0.00021571

2,4,1,19,1,0.0036124

2,4,1,19,2,0.0023186

2,4,1,19,3,-0.003429

2,4,1,19,4,-0.0011482

2,4,1,19,5,0.00043288

2,4,1,19,6,0.0008388

2,4,1,19,7,0.0012883

2,4,1,19,8,0.0003323

2,4,1,20,1,0.0017063

2,4,1,20,2,0.00026398

2,4,1,20,3,-0.0020975

2,4,1,20,4,-0.00050584

2,4,1,20,5,-0.00020646

2,4,1,20,6,0.00085805

2,4,1,20,7,-0.0004831

2,4,1,20,8,0.0010597

2,4,2,2,1,5.9998e-11

2,4,2,2,2,-0.00070132

2,4,2,2,3,-0.00013568

2,4,2,2,4,-0.00085738

2,4,2,2,5,0.00022544

2,4,2,2,6,-0.00082015

2,4,2,2,7,0.00026597

2,4,2,2,8,0.0010384

2,4,2,3,1,0.0015829

2,4,2,3,2,0.00072732

2,4,2,3,3,-0.0037575

2,4,2,3,4,-0.0017243

2,4,2,3,5,0.00014309

2,4,2,3,6,-0.0010605

2,4,2,3,7,0.00015747

2,4,2,3,8,0.00053626

2,4,2,4,1,4.6586e-10

2,4,2,4,2,0.0037441

2,4,2,4,3,-0.00187

2,4,2,4,4,-0.0015875

2,4,2,4,5,0.00022584

2,4,2,4,6,-0.0007115

2,4,2,4,7,-9.5496e-06

2,4,2,4,8,0.0021579

2,4,2,5,1,9.7029e-10

2,4,2,5,2,0.0013116

2,4,2,5,3,-0.0014986

2,4,2,5,4,-0.00088

2,4,2,5,5,0.00033347

2,4,2,5,6,-0.0004457

2,4,2,5,7,0.00061402

2,4,2,5,8,0.0022066

2,4,2,6,1,0.002007

2,4,2,6,2,-0.0014137

2,4,2,6,3,-0.0032572

2,4,2,6,4,-0.00017984

2,4,2,6,5,-0.00025411

2,4,2,6,6,0.00032453

2,4,2,6,7,-0.00037766

2,4,2,6,8,0.00063619

2,4,2,7,1,0.00012218

2,4,2,7,2,0.0017787

2,4,2,7,3,-0.0020992

2,4,2,7,4,-0.0029002

2,4,2,7,5,-0.00061075

2,4,2,7,6,-0.0015621

2,4,2,7,7,0.00056204

2,4,2,7,8,0.00083328

2,4,2,8,1,0.0020525

2,4,2,8,2,0.00030215

2,4,2,8,3,-0.002468

2,4,2,8,4,-0.0010375

2,4,2,8,5,0.00021614

2,4,2,8,6,-0.00029713

2,4,2,8,7,0.00020242

2,4,2,8,8,0.0027068

2,4,2,9,1,0.00091194

2,4,2,9,2,0.00065296

2,4,2,9,3,-0.0022699

2,4,2,9,4,8.1854e-05

2,4,2,9,5,-0.00015168

2,4,2,9,6,-0.00022152

2,4,2,9,7,0.00052034

2,4,2,9,8,0.0017587

2,4,2,10,1,7.0685e-09

2,4,2,10,2,0.0018895

2,4,2,10,3,-0.0020592

2,4,2,10,4,-0.00229

2,4,2,10,5,0.00038996

2,4,2,10,6,-0.0011145

2,4,2,10,7,0.00013641

2,4,2,10,8,0.0007528

2,4,2,11,1,0.00027973

2,4,2,11,2,0.0004848

2,4,2,11,3,-0.0026946

2,4,2,11,4,-0.0024292

2,4,2,11,5,-0.00022685

2,4,2,11,6,-0.00083438

2,4,2,11,7,0.00028622

2,4,2,11,8,0.0010969

2,4,2,12,1,0.0013744

2,4,2,12,2,0.00073871

2,4,2,12,3,-0.0015118

2,4,2,12,4,-0.0036269

2,4,2,12,5,0.00098793

2,4,2,12,6,-0.00085838

2,4,2,12,7,-9.2449e-05

2,4,2,12,8,0.0012812

2,4,2,13,1,0.0027383

2,4,2,13,2,-0.0022699

2,4,2,13,3,-0.002468

2,4,2,13,4,-0.00090688

2,4,2,13,5,-5.8958e-06

2,4,2,13,6,-0.00026572

2,4,2,13,7,0.000741

2,4,2,13,8,0.0023724

2,4,2,14,1,0.0011295

2,4,2,14,2,0.00031478

2,4,2,14,3,-0.0017433

2,4,2,14,4,-0.0018854

2,4,2,14,5,-0.00052108

2,4,2,14,6,-0.00069469

2,4,2,14,7,0.00010512

2,4,2,14,8,0.0022938

2,4,2,15,1,0.00059254

2,4,2,15,2,-8.0114e-05

2,4,2,15,3,-0.0017859

2,4,2,15,4,-0.0017681

2,4,2,15,5,-0.00058406

2,4,2,15,6,-9.2789e-06

2,4,2,15,7,-0.00060669

2,4,2,15,8,0.0017815

2,4,2,16,1,0.00067274

2,4,2,16,2,-0.0010374

2,4,2,16,3,-0.0020071

2,4,2,16,4,0.0001173

2,4,2,16,5,-0.00099292

2,4,2,16,6,-0.00085994

2,4,2,16,7,0.0004778

2,4,2,16,8,0.0013828

2,4,2,17,1,0.0021532

2,4,2,17,2,0.0010712

2,4,2,17,3,-0.00079853

2,4,2,17,4,-0.0018462

2,4,2,17,5,0.00048664

2,4,2,17,6,-0.00056052

2,4,2,17,7,0.00091563

2,4,2,17,8,0.0023249

2,4,2,18,1,2.027e-10

2,4,2,18,2,0.0020101

2,4,2,18,3,-0.0020314

2,4,2,18,4,-0.0012204

2,4,2,18,5,0.00025649

2,4,2,18,6,0.00015588

2,4,2,18,7,0.00054852

2,4,2,18,8,0.0028114

2,4,2,19,1,1.1808e-11

2,4,2,19,2,0.0024451

2,4,2,19,3,-0.0020634

2,4,2,19,4,-0.0010703

2,4,2,19,5,0.00034194

2,4,2,19,6,-0.00070262

2,4,2,19,7,0.0013221

2,4,2,19,8,0.0025757

2,4,2,20,1,3.9968e-09

2,4,2,20,2,0.00014176

2,4,2,20,3,-0.00096417

2,4,2,20,4,0.00017088

2,4,2,20,5,-0.00030184

2,4,2,20,6,-0.00027799

2,4,2,20,7,0.00022905

2,4,2,20,8,0.0017511

2,4,3,3,1,0.004117

2,4,3,3,2,0.0016319

2,4,3,3,3,-0.0023801

2,4,3,3,4,-0.0012237

2,4,3,3,5,0.00028154

2,4,3,3,6,-5.6336e-05

2,4,3,3,7,-0.000346

2,4,3,3,8,0.00033166

2,4,3,4,1,0.0067347

2,4,3,4,2,0.0025635

2,4,3,4,3,-0.0024279

2,4,3,4,4,-0.001438

2,4,3,4,5,0.00093406

2,4,3,4,6,-0.00068074

2,4,3,4,7,8.377e-05

2,4,3,4,8,-0.00013272

2,4,3,5,1,0.0061639

2,4,3,5,2,0.0013404

2,4,3,5,3,-0.0032062

2,4,3,5,4,-0.0023787

2,4,3,5,5,0.00022134

2,4,3,5,6,-0.00062348

2,4,3,5,7,-0.0006378

2,4,3,5,8,0.00059461

2,4,3,6,1,0.00405

2,4,3,6,2,0.0017725

2,4,3,6,3,-0.0076134

2,4,3,6,4,-0.0041614

2,4,3,6,5,0.0008573

2,4,3,6,6,-0.00054249

2,4,3,6,7,0.00010705

2,4,3,6,8,0.00069623

2,4,3,7,1,0.0037417

2,4,3,7,2,0.001112

2,4,3,7,3,-0.0019548

2,4,3,7,4,-0.001983

2,4,3,7,5,0.0012965

2,4,3,7,6,0.00019665

2,4,3,7,7,0.0009388

2,4,3,7,8,2.1831e-05

2,4,3,8,1,0.0058109

2,4,3,8,2,0.00027822

2,4,3,8,3,-0.0087049

2,4,3,8,4,-0.0033286

2,4,3,8,5,-0.00074426

2,4,3,8,6,-0.00098511

2,4,3,8,7,-0.00078829

2,4,3,8,8,0.00058399

2,4,3,9,1,0.0053405

2,4,3,9,2,0.0017361

2,4,3,9,3,-0.0048914

2,4,3,9,4,-0.0020637

2,4,3,9,5,-0.00036383

2,4,3,9,6,-0.0002904

2,4,3,9,7,0.00022756

2,4,3,9,8,0.00024585

2,4,3,10,1,0.0051627

2,4,3,10,2,-0.0015362

2,4,3,10,3,-0.0067337

2,4,3,10,4,-0.0039261

2,4,3,10,5,0.00010162

2,4,3,10,6,-0.00099594

2,4,3,10,7,0.0003159

2,4,3,10,8,0.00047272

2,4,3,11,1,0.0049799

2,4,3,11,2,0.00029237

2,4,3,11,3,-0.005819

2,4,3,11,4,-0.003134

2,4,3,11,5,-0.00095022

2,4,3,11,6,-0.001069

2,4,3,11,7,-0.00052071

2,4,3,11,8,0.00059817

2,4,3,12,1,0.0025862

2,4,3,12,2,0.0010352

2,4,3,12,3,-0.0045235

2,4,3,12,4,-0.0031698

2,4,3,12,5,-0.0001755

2,4,3,12,6,-0.00040131

2,4,3,12,7,-0.0010776

2,4,3,12,8,0.0004115

2,4,3,13,1,0.0057204

2,4,3,13,2,0.0016233

2,4,3,13,3,-0.00021636

2,4,3,13,4,-0.0021429

2,4,3,13,5,0.00072618

2,4,3,13,6,0.00080458

2,4,3,13,7,6.4475e-05

2,4,3,13,8,0.0010792

2,4,3,14,1,0.0052676

2,4,3,14,2,0.0014513

2,4,3,14,3,-0.0018826

2,4,3,14,4,-0.0015296

2,4,3,14,5,0.0010479

2,4,3,14,6,-3.0607e-05

2,4,3,14,7,0.00074378

2,4,3,14,8,0.00078598

2,4,3,15,1,0.0019876

2,4,3,15,2,-0.0001408

2,4,3,15,3,-0.0034958

2,4,3,15,4,-0.002073

2,4,3,15,5,0.00036359

2,4,3,15,6,-0.0011331

2,4,3,15,7,-9.0357e-05

2,4,3,15,8,0.0012459

2,4,3,16,1,0.0017614

2,4,3,16,2,0.00073651

2,4,3,16,3,-0.0022892

2,4,3,16,4,-0.0016748

2,4,3,16,5,0.00092211

2,4,3,16,6,-0.0010615

2,4,3,16,7,0.00027127

2,4,3,16,8,0.0010457

2,4,3,17,1,0.0077735

2,4,3,17,2,0.0028205

2,4,3,17,3,-0.0015732

2,4,3,17,4,-0.0019697

2,4,3,17,5,0.00069652

2,4,3,17,6,0.00044439

2,4,3,17,7,0.00019471

2,4,3,17,8,0.00014559

2,4,3,18,1,0.0033791

2,4,3,18,2,0.00098092

2,4,3,18,3,-0.0021923

2,4,3,18,4,-0.0020501

2,4,3,18,5,0.0016575

2,4,3,18,6,-0.00020669

2,4,3,18,7,0.00012188

2,4,3,18,8,0.0011849

2,4,3,19,1,0.0022027

2,4,3,19,2,-0.00071312

2,4,3,19,3,-0.0015236

2,4,3,19,4,-0.0030015

2,4,3,19,5,0.0010976

2,4,3,19,6,-0.0011064

2,4,3,19,7,-0.00020409

2,4,3,19,8,0.0015867

2,4,3,20,1,0.0014119

2,4,3,20,2,0.00021345

2,4,3,20,3,-0.0019062

2,4,3,20,4,-0.0024412

2,4,3,20,5,0.00082148

2,4,3,20,6,-0.00074024

2,4,3,20,7,0.001578

2,4,3,20,8,0.00083781

2,4,4,4,1,0.0080807

2,4,4,4,2,-0.00043831

2,4,4,4,3,0.00020513

2,4,4,4,4,-0.00079711

2,4,4,4,5,-0.00012808

2,4,4,4,6,-0.00063834

2,4,4,4,7,-0.00017993

2,4,4,4,8,0.0012268

2,4,4,5,1,0.0069828

2,4,4,5,2,0.00012809

2,4,4,5,3,-0.0002425

2,4,4,5,4,-0.0024551

2,4,4,5,5,-0.00026161

2,4,4,5,6,-0.001509

2,4,4,5,7,-0.00040247

2,4,4,5,8,0.0021274

2,4,4,6,1,0.0053559

2,4,4,6,2,-0.00051598

2,4,4,6,3,-0.002669

2,4,4,6,4,-0.0020653

2,4,4,6,5,0.0010119

2,4,4,6,6,-0.00080431

2,4,4,6,7,9.0062e-05

2,4,4,6,8,0.00064998

2,4,4,7,1,0.0038502

2,4,4,7,2,0.00080824

2,4,4,7,3,-0.0018666

2,4,4,7,4,-0.0021112

2,4,4,7,5,-0.00018599

2,4,4,7,6,-0.00040677

2,4,4,7,7,3.5031e-07

2,4,4,7,8,0.00031149

2,4,4,8,1,0.0049914

2,4,4,8,2,0.00078175

2,4,4,8,3,-0.0031341

2,4,4,8,4,-0.0013788

2,4,4,8,5,0.00072365

2,4,4,8,6,0.00042776

2,4,4,8,7,0.00081098

2,4,4,8,8,0.0021929

2,4,4,9,1,0.0054086

2,4,4,9,2,0.00036268

2,4,4,9,3,-0.0026932

2,4,4,9,4,-0.00063555

2,4,4,9,5,-0.0012594

2,4,4,9,6,-0.0010945

2,4,4,9,7,-0.00065065

2,4,4,9,8,0.001895

2,4,4,10,1,0.0076579

2,4,4,10,2,-0.00089686

2,4,4,10,3,-0.0034281

2,4,4,10,4,-0.0027597

2,4,4,10,5,-0.00095972

2,4,4,10,6,-0.0011053

2,4,4,10,7,-0.00058975

2,4,4,10,8,0.0018287

2,4,4,11,1,0.0043827

2,4,4,11,2,0.0022954

2,4,4,11,3,-0.0031612

2,4,4,11,4,-0.0026408

2,4,4,11,5,-0.0008575

2,4,4,11,6,-0.0010473

2,4,4,11,7,-0.00034879

2,4,4,11,8,0.0013868

2,4,4,12,1,0.0019014

2,4,4,12,2,0.0015526

2,4,4,12,3,-0.001455

2,4,4,12,4,-0.0017478

2,4,4,12,5,0.00037107

2,4,4,12,6,-0.00086384

2,4,4,12,7,0.0010311

2,4,4,12,8,0.0012968

2,4,4,13,1,0.0072546

2,4,4,13,2,-2.5531e-05

2,4,4,13,3,-0.00086231

2,4,4,13,4,-0.0021123

2,4,4,13,5,-0.00042092

2,4,4,13,6,-0.00095279

2,4,4,13,7,-0.00059766

2,4,4,13,8,0.0021935

2,4,4,14,1,0.0065817

2,4,4,14,2,-0.0011149

2,4,4,14,3,-0.001525

2,4,4,14,4,-0.0014245

2,4,4,14,5,-0.00068516

2,4,4,14,6,-0.00042272

2,4,4,14,7,-0.0013933

2,4,4,14,8,0.0021453

2,4,4,15,1,0.0022115

2,4,4,15,2,0.00031363

2,4,4,15,3,-2.1584e-05

2,4,4,15,4,-0.0023906

2,4,4,15,5,-0.00014156

2,4,4,15,6,-0.00067434

2,4,4,15,7,-0.00045566

2,4,4,15,8,0.0017535

2,4,4,16,1,0.0017802

2,4,4,16,2,1.3206e-05

2,4,4,16,3,-0.0010747

2,4,4,16,4,-0.00037376

2,4,4,16,5,0.00017273

2,4,4,16,6,-0.00069807

2,4,4,16,7,0.00050987

2,4,4,16,8,0.0012466

2,4,4,17,1,0.0089631

2,4,4,17,2,0.00034819

2,4,4,17,3,-0.00071034

2,4,4,17,4,-0.0031269

2,4,4,17,5,-0.00084361

2,4,4,17,6,-0.002181

2,4,4,17,7,-0.0007238

2,4,4,17,8,0.0020815

2,4,4,18,1,0.0042545

2,4,4,18,2,0.0001154

2,4,4,18,3,-0.00091609

2,4,4,18,4,-0.0010458

2,4,4,18,5,3.0886e-05

2,4,4,18,6,-0.0012315

2,4,4,18,7,0.00011395

2,4,4,18,8,0.0018927

2,4,4,19,1,0.0033498

2,4,4,19,2,0.0016282

2,4,4,19,3,-0.0014839

2,4,4,19,4,-0.00096049

2,4,4,19,5,-0.00072539

2,4,4,19,6,-0.0015124

2,4,4,19,7,-0.00061596

2,4,4,19,8,0.0022669

2,4,4,20,1,0.0016962

2,4,4,20,2,0.00025051

2,4,4,20,3,0.00022286

2,4,4,20,4,-0.00049821

2,4,4,20,5,0.00067209

2,4,4,20,6,-0.0010206

2,4,4,20,7,0.00026921

2,4,4,20,8,0.0019411

2,4,5,5,1,0.010504

2,4,5,5,2,-0.0014057

2,4,5,5,3,7.7745e-05

2,4,5,5,4,-0.0016892

2,4,5,5,5,-0.00016373

2,4,5,5,6,-0.00085407

2,4,5,5,7,-0.00035835

2,4,5,5,8,0.00097931

2,4,5,6,1,0.0051708

2,4,5,6,2,0.00049673

2,4,5,6,3,-0.0030571

2,4,5,6,4,-0.0030976

2,4,5,6,5,0.00081951

2,4,5,6,6,-0.0010022

2,4,5,6,7,-8.6214e-05

2,4,5,6,8,8.5715e-05

2,4,5,7,1,0.0049796

2,4,5,7,2,0.00094733

2,4,5,7,3,-0.0026049

2,4,5,7,4,-0.0032662

2,4,5,7,5,0.00080377

2,4,5,7,6,-0.00090388

2,4,5,7,7,0.00074422

2,4,5,7,8,0.0011182

2,4,5,8,1,0.00481

2,4,5,8,2,0.0010645

2,4,5,8,3,-0.0032391

2,4,5,8,4,-0.002786

2,4,5,8,5,0.00092931

2,4,5,8,6,-0.00071234

2,4,5,8,7,-8.7155e-05

2,4,5,8,8,0.00091783

2,4,5,9,1,0.0054929

2,4,5,9,2,0.00089647

2,4,5,9,3,-0.0017323

2,4,5,9,4,-0.001127

2,4,5,9,5,-0.00044717

2,4,5,9,6,-0.00027142

2,4,5,9,7,-0.00087419

2,4,5,9,8,0.0030864

2,4,5,10,1,0.007395

2,4,5,10,2,0.0022187

2,4,5,10,3,-0.0019938

2,4,5,10,4,-0.0027314

2,4,5,10,5,0.00025007

2,4,5,10,6,-0.000595

2,4,5,10,7,-0.00020328

2,4,5,10,8,0.00086216

2,4,5,11,1,0.005258

2,4,5,11,2,0.0011288

2,4,5,11,3,-0.0042817

2,4,5,11,4,-0.0034401

2,4,5,11,5,-0.0009191

2,4,5,11,6,-0.0017597

2,4,5,11,7,-0.00037747

2,4,5,11,8,0.00042878

2,4,5,12,1,0.0028391

2,4,5,12,2,0.0017579

2,4,5,12,3,-0.0028335

2,4,5,12,4,-0.0026706

2,4,5,12,5,0.00097067

2,4,5,12,6,-0.00062667

2,4,5,12,7,0.00034484

2,4,5,12,8,0.001986

2,4,5,13,1,0.0098723

2,4,5,13,2,-0.00014007

2,4,5,13,3,-0.0026031

2,4,5,13,4,-0.0017467

2,4,5,13,5,-0.0010154

2,4,5,13,6,-0.00020652

2,4,5,13,7,-0.00021812

2,4,5,13,8,0.002482

2,4,5,14,1,0.0070183

2,4,5,14,2,-0.0006646

2,4,5,14,3,-0.0028445

2,4,5,14,4,-0.0023802

2,4,5,14,5,-0.0013366

2,4,5,14,6,-0.0012874

2,4,5,14,7,0.0003263

2,4,5,14,8,0.001882

2,4,5,15,1,0.002488

2,4,5,15,2,5.1018e-05

2,4,5,15,3,-0.0012466

2,4,5,15,4,-0.0016007

2,4,5,15,5,-0.00033429

2,4,5,15,6,-0.00081262

2,4,5,15,7,9.6696e-06

2,4,5,15,8,0.0026464

2,4,5,16,1,0.0014886

2,4,5,16,2,-0.00069578

2,4,5,16,3,-0.00027953

2,4,5,16,4,-0.00085629

2,4,5,16,5,-7.1341e-05

2,4,5,16,6,-0.0012021

2,4,5,16,7,0.00043219

2,4,5,16,8,0.0013289

2,4,5,17,1,0.010602

2,4,5,17,2,0.00079877

2,4,5,17,3,-0.0015734

2,4,5,17,4,-0.0031026

2,4,5,17,5,-0.0015278

2,4,5,17,6,-0.0017463

2,4,5,17,7,-1.0003e-05

2,4,5,17,8,0.0018039

2,4,5,18,1,0.0032011

2,4,5,18,2,0.0013023

2,4,5,18,3,-0.0014724

2,4,5,18,4,-0.0018913

2,4,5,18,5,-0.00057664

2,4,5,18,6,-0.0017189

2,4,5,18,7,0.00029744

2,4,5,18,8,0.0020348

2,4,5,19,1,0.0031159

2,4,5,19,2,2.2247e-05

2,4,5,19,3,-0.0015947

2,4,5,19,4,-0.0023925

2,4,5,19,5,-0.00033379

2,4,5,19,6,-0.0014008

2,4,5,19,7,5.1801e-05

2,4,5,19,8,0.0028622

2,4,5,20,1,0.0015029

2,4,5,20,2,0.00086591

2,4,5,20,3,-0.00034351

2,4,5,20,4,-0.0020383

2,4,5,20,5,-0.00039398

2,4,5,20,6,-0.001089

2,4,5,20,7,-0.00036484

2,4,5,20,8,0.0022442

2,4,6,6,1,0.0043638

2,4,6,6,2,0.00044621

2,4,6,6,3,-0.0046708

2,4,6,6,4,-0.0021427

2,4,6,6,5,-0.00074977

2,4,6,6,6,-0.00050963

2,4,6,6,7,-0.0003385

2,4,6,6,8,-0.00018954

2,4,6,7,1,0.0034896

2,4,6,7,2,0.001375

2,4,6,7,3,-0.0044801

2,4,6,7,4,-0.0032481

2,4,6,7,5,0.00032451

2,4,6,7,6,-0.0001957

2,4,6,7,7,-0.00014042

2,4,6,7,8,0.0011678

2,4,6,8,1,0.0043351

2,4,6,8,2,0.00035158

2,4,6,8,3,-0.0058807

2,4,6,8,4,-0.0026159

2,4,6,8,5,-0.00017357

2,4,6,8,6,-0.00047913

2,4,6,8,7,0.00046139

2,4,6,8,8,0.00039685

2,4,6,9,1,0.0043122

2,4,6,9,2,0.00047144

2,4,6,9,3,-0.0030663

2,4,6,9,4,-0.0013307

2,4,6,9,5,-0.00021501

2,4,6,9,6,0.00096694

2,4,6,9,7,-0.00071218

2,4,6,9,8,0.0019898

2,4,6,10,1,0.004596

2,4,6,10,2,-0.00025718

2,4,6,10,3,-0.0053496

2,4,6,10,4,-0.0030528

2,4,6,10,5,0.0015747

2,4,6,10,6,-0.00074054

2,4,6,10,7,4.3326e-05

2,4,6,10,8,0.00036888

2,4,6,11,1,0.004031

2,4,6,11,2,0.0010911

2,4,6,11,3,-0.0040075

2,4,6,11,4,-0.0034304

2,4,6,11,5,0.00020718

2,4,6,11,6,0.00021605

2,4,6,11,7,-5.4576e-05

2,4,6,11,8,8.8408e-05

2,4,6,12,1,0.0021161

2,4,6,12,2,0.0018358

2,4,6,12,3,-0.0033719

2,4,6,12,4,-0.0045294

2,4,6,12,5,-0.00044891

2,4,6,12,6,-0.00053093

2,4,6,12,7,-0.00062563

2,4,6,12,8,0.00094136

2,4,6,13,1,0.0056039

2,4,6,13,2,0.00032831

2,4,6,13,3,-0.00099209

2,4,6,13,4,-0.0011597

2,4,6,13,5,0.00065357

2,4,6,13,6,0.00047772

2,4,6,13,7,0.00065217

2,4,6,13,8,-5.2687e-05

2,4,6,14,1,0.0043535

2,4,6,14,2,0.00084297

2,4,6,14,3,-0.0014577

2,4,6,14,4,-0.002096

2,4,6,14,5,0.00057981

2,4,6,14,6,0.00060333

2,4,6,14,7,0.00015729

2,4,6,14,8,0.0012299

2,4,6,15,1,0.0019718

2,4,6,15,2,0.0010404

2,4,6,15,3,-0.0030284

2,4,6,15,4,-0.0019709

2,4,6,15,5,0.0002085

2,4,6,15,6,-0.0003445

2,4,6,15,7,-0.0011803

2,4,6,15,8,-6.7599e-05

2,4,6,16,1,0.001569

2,4,6,16,2,0.00094861

2,4,6,16,3,-0.0036171

2,4,6,16,4,-0.0018127

2,4,6,16,5,9.9961e-05

2,4,6,16,6,-0.00112

2,4,6,16,7,0.00070764

2,4,6,16,8,0.00058411

2,4,6,17,1,0.0062309

2,4,6,17,2,0.0020567

2,4,6,17,3,-0.0017057

2,4,6,17,4,-0.0034302

2,4,6,17,5,0.0010269

2,4,6,17,6,0.00024682

2,4,6,17,7,0.00028223

2,4,6,17,8,9.2097e-05

2,4,6,18,1,0.0032243

2,4,6,18,2,0.000885

2,4,6,18,3,-0.0031962

2,4,6,18,4,-0.001404

2,4,6,18,5,7.4998e-05

2,4,6,18,6,0.00015248

2,4,6,18,7,-7.6185e-05

2,4,6,18,8,0.00067891

2,4,6,19,1,0.0026601

2,4,6,19,2,0.0003176

2,4,6,19,3,-0.00092134

2,4,6,19,4,-0.0027219

2,4,6,19,5,0.00036048

2,4,6,19,6,0.00030475

2,4,6,19,7,0.00022414

2,4,6,19,8,0.00034908

2,4,6,20,1,0.0012496

2,4,6,20,2,-0.00044115

2,4,6,20,3,-0.0020399

2,4,6,20,4,-0.0019989

2,4,6,20,5,-2.4752e-05

2,4,6,20,6,0.00035679

2,4,6,20,7,-0.00067052

2,4,6,20,8,0.0019168

2,4,7,7,1,0.0029482

2,4,7,7,2,0.00068463

2,4,7,7,3,-0.00017294

2,4,7,7,4,-0.0020429

2,4,7,7,5,-0.00035468

2,4,7,7,6,0.00026833

2,4,7,7,7,-0.00081525

2,4,7,7,8,7.0687e-05

2,4,7,8,1,0.0040011

2,4,7,8,2,-0.00029589

2,4,7,8,3,-0.0032369

2,4,7,8,4,-0.0031715

2,4,7,8,5,-6.3735e-05

2,4,7,8,6,-0.00085022

2,4,7,8,7,-0.0002971

2,4,7,8,8,5.708e-05

2,4,7,9,1,0.002152

2,4,7,9,2,0.0011177

2,4,7,9,3,-0.0042863

2,4,7,9,4,-0.0013169

2,4,7,9,5,-0.0010666

2,4,7,9,6,0.00044763

2,4,7,9,7,0.00023651

2,4,7,9,8,0.0012524

2,4,7,10,1,0.0040482

2,4,7,10,2,0.00083438

2,4,7,10,3,-0.0039207

2,4,7,10,4,-0.0028199

2,4,7,10,5,-0.00062105

2,4,7,10,6,0.00039031

2,4,7,10,7,-0.0011563

2,4,7,10,8,0.00088599

2,4,7,11,1,0.0025664

2,4,7,11,2,6.7595e-05

2,4,7,11,3,-0.0019264

2,4,7,11,4,-0.002466

2,4,7,11,5,-0.0013028

2,4,7,11,6,-0.00060681

2,4,7,11,7,0.00062518

2,4,7,11,8,0.0015211

2,4,7,12,1,0.0012049

2,4,7,12,2,0.00077624

2,4,7,12,3,-0.00029275

2,4,7,12,4,-0.0029474

2,4,7,12,5,0.0017009

2,4,7,12,6,-0.00031661

2,4,7,12,7,-0.00018808

2,4,7,12,8,0.0012977

2,4,7,13,1,0.0049418

2,4,7,13,2,0.00032104

2,4,7,13,3,-0.0030374

2,4,7,13,4,-0.002916

2,4,7,13,5,-0.00017773

2,4,7,13,6,0.00082046

2,4,7,13,7,0.00040366

2,4,7,13,8,0.001997

2,4,7,14,1,0.0041615

2,4,7,14,2,-0.001038

2,4,7,14,3,-0.0019014

2,4,7,14,4,-0.0033363

2,4,7,14,5,-0.00091208

2,4,7,14,6,-0.00015353

2,4,7,14,7,0.0001497

2,4,7,14,8,0.001702

2,4,7,15,1,0.0014402

2,4,7,15,2,0.00065098

2,4,7,15,3,-0.0017246

2,4,7,15,4,-0.0019085

2,4,7,15,5,8.5363e-05

2,4,7,15,6,-0.00058106

2,4,7,15,7,0.0017921

2,4,7,15,8,0.00087913

2,4,7,16,1,0.0019066

2,4,7,16,2,6.0516e-05

2,4,7,16,3,-0.0021214

2,4,7,16,4,-0.0021536

2,4,7,16,5,0.00060422

2,4,7,16,6,0.00045017

2,4,7,16,7,0.00022657

2,4,7,16,8,0.002405

2,4,7,17,1,0.0056666

2,4,7,17,2,0.0018205

2,4,7,17,3,-0.0031074

2,4,7,17,4,-0.0034616

2,4,7,17,5,-0.00066588

2,4,7,17,6,-0.0013605

2,4,7,17,7,0.00012629

2,4,7,17,8,0.0010233

2,4,7,18,1,0.002371

2,4,7,18,2,0.00068536

2,4,7,18,3,-0.0022013

2,4,7,18,4,-0.0024915

2,4,7,18,5,7.0446e-05

2,4,7,18,6,0.00049373

2,4,7,18,7,7.5531e-05

2,4,7,18,8,0.0029939

2,4,7,19,1,0.0030757

2,4,7,19,2,-0.00014057

2,4,7,19,3,-0.0027776

2,4,7,19,4,-0.0021721

2,4,7,19,5,-0.00010294

2,4,7,19,6,-0.00056611

2,4,7,19,7,0.0010223

2,4,7,19,8,0.001339

2,4,7,20,1,0.0006443

2,4,7,20,2,0.00034042

2,4,7,20,3,-0.0012904

2,4,7,20,4,-0.0026596

2,4,7,20,5,0.00058466

2,4,7,20,6,-0.00029894

2,4,7,20,7,0.0010967

2,4,7,20,8,0.0019945

2,4,8,8,1,0.0070691

2,4,8,8,2,0.00083103

2,4,8,8,3,-0.0022927

2,4,8,8,4,-0.00028672

2,4,8,8,5,-0.00022713

2,4,8,8,6,0.00011602

2,4,8,8,7,-1.1671e-05

2,4,8,8,8,-6.4732e-05

2,4,8,9,1,0.0054284

2,4,8,9,2,-0.0014716

2,4,8,9,3,-0.0039518

2,4,8,9,4,-0.00040616

2,4,8,9,5,0.00049171

2,4,8,9,6,-0.00084225

2,4,8,9,7,0.0006368

2,4,8,9,8,0.00096135

2,4,8,10,1,0.0059936

2,4,8,10,2,-0.0014346

2,4,8,10,3,-0.0039447

2,4,8,10,4,-0.0018262

2,4,8,10,5,-0.00026368

2,4,8,10,6,0.00034556

2,4,8,10,7,5.6663e-05

2,4,8,10,8,0.00052061

2,4,8,11,1,0.0047995

2,4,8,11,2,0.00022096

2,4,8,11,3,-0.0041741

2,4,8,11,4,-0.0029424

2,4,8,11,5,0.00041139

2,4,8,11,6,-0.0012653

2,4,8,11,7,0.00029856

2,4,8,11,8,0.00076479

2,4,8,12,1,0.0030337

2,4,8,12,2,0.0013047

2,4,8,12,3,-0.0043957

2,4,8,12,4,-0.0024586

2,4,8,12,5,0.00055748

2,4,8,12,6,-0.0004512

2,4,8,12,7,8.1456e-07

2,4,8,12,8,0.00036633

2,4,8,13,1,0.0077897

2,4,8,13,2,3.1154e-06

2,4,8,13,3,-0.0031782

2,4,8,13,4,-0.0023038

2,4,8,13,5,1.2697e-05

2,4,8,13,6,-0.00044317

2,4,8,13,7,-0.00062495

2,4,8,13,8,0.00079801

2,4,8,14,1,0.0057026

2,4,8,14,2,0.00018026

2,4,8,14,3,-0.0022374

2,4,8,14,4,-0.0015934

2,4,8,14,5,0.0005638

2,4,8,14,6,0.00067659

2,4,8,14,7,0.00028931

2,4,8,14,8,0.0015269

2,4,8,15,1,0.0018985

2,4,8,15,2,5.3819e-05

2,4,8,15,3,-0.0042236

2,4,8,15,4,-0.0021662

2,4,8,15,5,-0.00084722

2,4,8,15,6,-0.00031709

2,4,8,15,7,0.00017157

2,4,8,15,8,0.0018892

2,4,8,16,1,0.0020845

2,4,8,16,2,0.00017743

2,4,8,16,3,-0.0028071

2,4,8,16,4,-0.0018411

2,4,8,16,5,0.00070068

2,4,8,16,6,-0.00089774

2,4,8,16,7,0.00046217

2,4,8,16,8,0.00093159

2,4,8,17,1,0.0073556

2,4,8,17,2,0.0028378

2,4,8,17,3,-0.0022353

2,4,8,17,4,-0.0022554

2,4,8,17,5,0.0012402

2,4,8,17,6,-0.00028943

2,4,8,17,7,0.00028403

2,4,8,17,8,0.0016472

2,4,8,18,1,0.0043884

2,4,8,18,2,0.00049627

2,4,8,18,3,-0.0036147

2,4,8,18,4,-0.0029607

2,4,8,18,5,0.0013789

2,4,8,18,6,-0.00074091

2,4,8,18,7,0.00062064

2,4,8,18,8,0.00065352

2,4,8,19,1,0.003103

2,4,8,19,2,-0.00032556

2,4,8,19,3,-0.0032855

2,4,8,19,4,-0.00090224

2,4,8,19,5,0.00075687

2,4,8,19,6,0.00041288

2,4,8,19,7,0.0006955

2,4,8,19,8,0.0017137

2,4,8,20,1,0.001185

2,4,8,20,2,0.0010237

2,4,8,20,3,-0.00249

2,4,8,20,4,-0.0023559

2,4,8,20,5,0.0015115

2,4,8,20,6,-0.000562

2,4,8,20,7,0.0011993

2,4,8,20,8,0.00083932

2,4,9,9,1,0.0063292

2,4,9,9,2,-0.0015108

2,4,9,9,3,-4.7371e-05

2,4,9,9,4,0.0003526

2,4,9,9,5,-0.00023696

2,4,9,9,6,-0.0001499

2,4,9,9,7,0.00036631

2,4,9,9,8,0.00097384

2,4,9,10,1,0.0053853

2,4,9,10,2,0.00054916

2,4,9,10,3,-0.0034831

2,4,9,10,4,-0.00099538

2,4,9,10,5,-0.0004059

2,4,9,10,6,0.00022016

2,4,9,10,7,0.00056389

2,4,9,10,8,0.0011487

2,4,9,11,1,0.0048093

2,4,9,11,2,0.00063741

2,4,9,11,3,-0.0037504

2,4,9,11,4,-0.0017266

2,4,9,11,5,-0.0013436

2,4,9,11,6,6.4727e-05

2,4,9,11,7,-0.00033988

2,4,9,11,8,0.001711

2,4,9,12,1,0.0022619

2,4,9,12,2,0.0012572

2,4,9,12,3,-0.0030078

2,4,9,12,4,-0.00084677

2,4,9,12,5,0.00052479

2,4,9,12,6,1.5026e-05

2,4,9,12,7,0.0011963

2,4,9,12,8,0.0018827

2,4,9,13,1,0.0070756

2,4,9,13,2,-0.00059152

2,4,9,13,3,-0.00097782

2,4,9,13,4,-0.0009336

2,4,9,13,5,9.5265e-05

2,4,9,13,6,-0.00045618

2,4,9,13,7,0.00024709

2,4,9,13,8,0.0029713

2,4,9,14,1,0.0056399

2,4,9,14,2,-0.0014201

2,4,9,14,3,-0.0014652

2,4,9,14,4,-0.0014453

2,4,9,14,5,-0.00022926

2,4,9,14,6,-0.00077317

2,4,9,14,7,-0.00034071

2,4,9,14,8,0.0030169

2,4,9,15,1,0.0017761

2,4,9,15,2,0.00025034

2,4,9,15,3,-0.001504

2,4,9,15,4,-0.00083294

2,4,9,15,5,-0.00053182

2,4,9,15,6,-0.0013638

2,4,9,15,7,-0.00020106

2,4,9,15,8,0.0018429

2,4,9,16,1,0.0028632

2,4,9,16,2,-0.0011232

2,4,9,16,3,-0.0014832

2,4,9,16,4,-0.0008412

2,4,9,16,5,-0.00089342

2,4,9,16,6,-0.00028978

2,4,9,16,7,0.00011424

2,4,9,16,8,0.001953

2,4,9,17,1,0.007874

2,4,9,17,2,6.7829e-05

2,4,9,17,3,-0.0022825

2,4,9,17,4,-0.00165

2,4,9,17,5,-0.00046321

2,4,9,17,6,-0.0013444

2,4,9,17,7,3.1614e-05

2,4,9,17,8,0.0025638

2,4,9,18,1,0.0029952

2,4,9,18,2,0.00076103

2,4,9,18,3,-0.0015656

2,4,9,18,4,-0.00087564

2,4,9,18,5,0.00041118

2,4,9,18,6,-0.0011152

2,4,9,18,7,0.0010207

2,4,9,18,8,0.0023027

2,4,9,19,1,0.0024262

2,4,9,19,2,0.00012943

2,4,9,19,3,-0.0027771

2,4,9,19,4,-0.00075941

2,4,9,19,5,0.00027752

2,4,9,19,6,-0.0010521

2,4,9,19,7,-4.2806e-05

2,4,9,19,8,0.002547

2,4,9,20,1,0.0011844

2,4,9,20,2,0.00064367

2,4,9,20,3,-0.0020054

2,4,9,20,4,-0.0010076

2,4,9,20,5,-0.00049529

2,4,9,20,6,-0.0013473

2,4,9,20,7,0.00013678

2,4,9,20,8,0.0020013

2,4,10,10,1,0.0070583

2,4,10,10,2,-0.0017838

2,4,10,10,3,-0.00059217

2,4,10,10,4,-0.0006898

2,4,10,10,5,0.000213

2,4,10,10,6,-0.00045161

2,4,10,10,7,-2.641e-05

2,4,10,10,8,6.6547e-05

2,4,10,11,1,0.005408

2,4,10,11,2,0.0015613

2,4,10,11,3,-0.00012146

2,4,10,11,4,-0.0026397

2,4,10,11,5,0.00089923

2,4,10,11,6,-0.00014824

2,4,10,11,7,0.0013743

2,4,10,11,8,0.0014863

2,4,10,12,1,0.0021404

2,4,10,12,2,0.00062498

2,4,10,12,3,-0.0018984

2,4,10,12,4,-0.0036451

2,4,10,12,5,8.4398e-05

2,4,10,12,6,-0.00062104

2,4,10,12,7,5.7682e-05

2,4,10,12,8,0.00017707

2,4,10,13,1,0.0075731

2,4,10,13,2,0.00094267

2,4,10,13,3,-0.0037943

2,4,10,13,4,-0.0014987

2,4,10,13,5,-0.00010581

2,4,10,13,6,-0.00074304

2,4,10,13,7,0.00029624

2,4,10,13,8,0.0010262

2,4,10,14,1,0.0058173

2,4,10,14,2,0.00038502

2,4,10,14,3,-0.0021079

2,4,10,14,4,-0.0017883

2,4,10,14,5,8.5147e-05

2,4,10,14,6,-0.00062547

2,4,10,14,7,0.00080587

2,4,10,14,8,0.0012003

2,4,10,15,1,0.0025284

2,4,10,15,2,-3.5649e-05

2,4,10,15,3,-0.0022354

2,4,10,15,4,-0.0019715

2,4,10,15,5,0.0008171

2,4,10,15,6,-0.00042773

2,4,10,15,7,4.4956e-06

2,4,10,15,8,0.0022229

2,4,10,16,1,0.0018408

2,4,10,16,2,-0.00010976

2,4,10,16,3,-0.0019417

2,4,10,16,4,-0.001362

2,4,10,16,5,0.00036491

2,4,10,16,6,-0.0012251

2,4,10,16,7,0.00044316

2,4,10,16,8,0.0015516

2,4,10,17,1,0.0088692

2,4,10,17,2,0.0016915

2,4,10,17,3,-0.0040532

2,4,10,17,4,-0.0026425

2,4,10,17,5,-0.00087151

2,4,10,17,6,0.00012136

2,4,10,17,7,0.00017247

2,4,10,17,8,0.0015635

2,4,10,18,1,0.0034236

2,4,10,18,2,-0.00025392

2,4,10,18,3,-0.0034367

2,4,10,18,4,-0.0036819

2,4,10,18,5,-0.0002985

2,4,10,18,6,-0.00088479

2,4,10,18,7,0.00029296

2,4,10,18,8,0.001131

2,4,10,19,1,0.0048842

2,4,10,19,2,-0.00061668

2,4,10,19,3,-0.0041986

2,4,10,19,4,-0.0027325

2,4,10,19,5,-0.00056624

2,4,10,19,6,-0.00045216

2,4,10,19,7,0.00088444

2,4,10,19,8,0.00084121

2,4,10,20,1,0.0020075

2,4,10,20,2,-0.00022785

2,4,10,20,3,-0.0024143

2,4,10,20,4,-0.0038099

2,4,10,20,5,0.00013722

2,4,10,20,6,-0.0014639

2,4,10,20,7,-1.9166e-05

2,4,10,20,8,0.0025202

2,4,11,11,1,0.0052

2,4,11,11,2,0.0007536

2,4,11,11,3,-0.0015996

2,4,11,11,4,-0.0014965

2,4,11,11,5,-0.00038633

2,4,11,11,6,-0.00053189

2,4,11,11,7,-0.00044393

2,4,11,11,8,0.00046141

2,4,11,12,1,0.001809

2,4,11,12,2,-0.00092484

2,4,11,12,3,-0.0023364

2,4,11,12,4,-0.0029892

2,4,11,12,5,-0.0005126

2,4,11,12,6,-0.00056807

2,4,11,12,7,-0.00043386

2,4,11,12,8,-0.00059395

2,4,11,13,1,0.0059139

2,4,11,13,2,-0.00043549

2,4,11,13,3,-0.001894

2,4,11,13,4,-0.0030864

2,4,11,13,5,-0.00034327

2,4,11,13,6,-0.00016022

2,4,11,13,7,6.0131e-05

2,4,11,13,8,0.0010665

2,4,11,14,1,0.0048738

2,4,11,14,2,-0.00086225

2,4,11,14,3,-0.0031992

2,4,11,14,4,-0.0030316

2,4,11,14,5,-0.00089653

2,4,11,14,6,0.00032682

2,4,11,14,7,-0.00072177

2,4,11,14,8,0.0023437

2,4,11,15,1,0.0020458

2,4,11,15,2,0.00079653

2,4,11,15,3,-0.0022203

2,4,11,15,4,-0.0021601

2,4,11,15,5,0.000152

2,4,11,15,6,-0.0001264

2,4,11,15,7,0.00052163

2,4,11,15,8,0.0011163

2,4,11,16,1,0.0021851

2,4,11,16,2,-0.00039708

2,4,11,16,3,-0.0015155

2,4,11,16,4,-0.0021277

2,4,11,16,5,-0.00057777

2,4,11,16,6,-0.001001

2,4,11,16,7,0.00090274

2,4,11,16,8,0.0013898

2,4,11,17,1,0.0079219

2,4,11,17,2,0.0010335

2,4,11,17,3,-0.0041032

2,4,11,17,4,-0.0037994

2,4,11,17,5,-0.0015685

2,4,11,17,6,-0.0013446

2,4,11,17,7,-0.0004081

2,4,11,17,8,0.0010767

2,4,11,18,1,0.0032388

2,4,11,18,2,-0.0004856

2,4,11,18,3,-0.0051668

2,4,11,18,4,-0.0029903

2,4,11,18,5,-0.0016247

2,4,11,18,6,-0.00038444

2,4,11,18,7,-8.0519e-05

2,4,11,18,8,0.0012582

2,4,11,19,1,0.0030843

2,4,11,19,2,-2.4489e-05

2,4,11,19,3,-0.004029

2,4,11,19,4,-0.0018354

2,4,11,19,5,-0.00094659

2,4,11,19,6,0.0001521

2,4,11,19,7,0.00024235

2,4,11,19,8,0.00074913

2,4,11,20,1,0.0012146

2,4,11,20,2,-0.00028922

2,4,11,20,3,-0.0032489

2,4,11,20,4,-0.0023368

2,4,11,20,5,-0.00046097

2,4,11,20,6,0.0012263

2,4,11,20,7,2.4647e-05

2,4,11,20,8,0.00090151

2,4,12,12,1,0.0014639

2,4,12,12,2,0.00030908

2,4,12,12,3,-0.0018896

2,4,12,12,4,-0.0016951

2,4,12,12,5,-0.00049778

2,4,12,12,6,0.00014144

2,4,12,12,7,0.00038557

2,4,12,12,8,2.76e-05

2,4,12,13,1,0.0019395

2,4,12,13,2,-0.00035459

2,4,12,13,3,-0.0028047

2,4,12,13,4,-0.0031548

2,4,12,13,5,0.00044171

2,4,12,13,6,-0.00061516

2,4,12,13,7,0.00092235

2,4,12,13,8,0.0015889

2,4,12,14,1,0.0024376

2,4,12,14,2,0.0011127

2,4,12,14,3,-0.0035963

2,4,12,14,4,-0.0017533

2,4,12,14,5,-0.00044441

2,4,12,14,6,0.00017411

2,4,12,14,7,-8.8429e-05

2,4,12,14,8,0.0010625

2,4,12,15,1,0.0013417

2,4,12,15,2,-0.00067053

2,4,12,15,3,-0.0029769

2,4,12,15,4,-0.0029702

2,4,12,15,5,0.00073538

2,4,12,15,6,0.00098738

2,4,12,15,7,0.00050703

2,4,12,15,8,0.0014945

2,4,12,16,1,0.0013152

2,4,12,16,2,0.00056214

2,4,12,16,3,-0.0028774

2,4,12,16,4,-0.004141

2,4,12,16,5,0.00018298

2,4,12,16,6,-0.0018441

2,4,12,16,7,-1.0677e-06

2,4,12,16,8,0.0016366

2,4,12,17,1,0.0031714

2,4,12,17,2,0.0016825

2,4,12,17,3,-0.0032706

2,4,12,17,4,-0.0037033

2,4,12,17,5,-0.0011423

2,4,12,17,6,-0.0012454

2,4,12,17,7,0.00057914

2,4,12,17,8,0.0016264

2,4,12,18,1,0.0018075

2,4,12,18,2,0.00041537

2,4,12,18,3,-0.0035272

2,4,12,18,4,-0.0029057

2,4,12,18,5,-7.5178e-05

2,4,12,18,6,-0.0005479

2,4,12,18,7,0.0003407

2,4,12,18,8,0.0023644

2,4,12,19,1,0.0017213

2,4,12,19,2,0.00029455

2,4,12,19,3,-0.0034414

2,4,12,19,4,-0.0016228

2,4,12,19,5,0.00031432

2,4,12,19,6,3.6945e-05

2,4,12,19,7,0.00030573

2,4,12,19,8,0.0019011

2,4,12,20,1,0.00067784

2,4,12,20,2,0.0010078

2,4,12,20,3,-0.0014449

2,4,12,20,4,-0.0021224

2,4,12,20,5,0.00056259

2,4,12,20,6,-0.0015592

2,4,12,20,7,0.00030809

2,4,12,20,8,0.00023351

2,4,13,13,1,0.011636

2,4,13,13,2,-0.00038445

2,4,13,13,3,-0.0015014

2,4,13,13,4,-0.00048572

2,4,13,13,5,-0.00097323

2,4,13,13,6,-0.00069663

2,4,13,13,7,0.0002123

2,4,13,13,8,0.00070558

2,4,13,14,1,0.0094827

2,4,13,14,2,-0.0007683

2,4,13,14,3,-0.0032237

2,4,13,14,4,-0.0013041

2,4,13,14,5,-0.001752

2,4,13,14,6,-0.0012114

2,4,13,14,7,0.00032369

2,4,13,14,8,0.0020349

2,4,13,15,1,0.0021488

2,4,13,15,2,0.00059837

2,4,13,15,3,-0.00323

2,4,13,15,4,-0.00083889

2,4,13,15,5,-0.00045467

2,4,13,15,6,-0.001784

2,4,13,15,7,0.00016785

2,4,13,15,8,0.0019776

2,4,13,16,1,0.0024034

2,4,13,16,2,-0.0011369

2,4,13,16,3,-0.0018518

2,4,13,16,4,-0.0017115

2,4,13,16,5,-0.00074009

2,4,13,16,6,-0.0007663

2,4,13,16,7,6.3984e-05

2,4,13,16,8,0.0019308

2,4,13,17,1,0.010229

2,4,13,17,2,0.00075067

2,4,13,17,3,-0.0033243

2,4,13,17,4,-0.0022456

2,4,13,17,5,-0.0019449

2,4,13,17,6,-0.0014624

2,4,13,17,7,-0.00010811

2,4,13,17,8,0.0020692

2,4,13,18,1,0.0049859

2,4,13,18,2,-2.3747e-05

2,4,13,18,3,-0.0032925

2,4,13,18,4,-0.0011347

2,4,13,18,5,-0.001063

2,4,13,18,6,-0.0015262

2,4,13,18,7,0.00072227

2,4,13,18,8,0.0020253

2,4,13,19,1,0.0034271

2,4,13,19,2,-0.00046823

2,4,13,19,3,-0.0033682

2,4,13,19,4,-0.00078412

2,4,13,19,5,-0.00067349

2,4,13,19,6,-0.0013163

2,4,13,19,7,0.0011259

2,4,13,19,8,0.002314

2,4,13,20,1,0.0014319

2,4,13,20,2,-0.00050997

2,4,13,20,3,-0.00086358

2,4,13,20,4,-0.0014906

2,4,13,20,5,0.00080741

2,4,13,20,6,-0.00061491

2,4,13,20,7,0.001148

2,4,13,20,8,0.0022873

2,4,14,14,1,0.0061004

2,4,14,14,2,-0.00034378

2,4,14,14,3,-0.0016316

2,4,14,14,4,8.652e-05

2,4,14,14,5,-0.0011037

2,4,14,14,6,7.8042e-05

2,4,14,14,7,0.00070928

2,4,14,14,8,0.0016503

2,4,14,15,1,0.0021084

2,4,14,15,2,0.00020876

2,4,14,15,3,-0.0024652

2,4,14,15,4,-0.0018715

2,4,14,15,5,-0.0014209

2,4,14,15,6,-0.0014893

2,4,14,15,7,0.00072108

2,4,14,15,8,0.0021464

2,4,14,16,1,0.0021114

2,4,14,16,2,-0.00039167

2,4,14,16,3,-0.0025037

2,4,14,16,4,-0.0013786

2,4,14,16,5,-0.0017654

2,4,14,16,6,-0.0019389

2,4,14,16,7,0.00012628

2,4,14,16,8,0.00062057

2,4,14,17,1,0.0075241

2,4,14,17,2,-0.00012399

2,4,14,17,3,-0.0037372

2,4,14,17,4,-0.0029826

2,4,14,17,5,-0.0021309

2,4,14,17,6,-0.003038

2,4,14,17,7,5.8711e-06

2,4,14,17,8,0.001501

2,4,14,18,1,0.0031871

2,4,14,18,2,0.0008056

2,4,14,18,3,-0.0034468

2,4,14,18,4,-0.00053375

2,4,14,18,5,-0.0012765

2,4,14,18,6,-0.00095284

2,4,14,18,7,0.00047597

2,4,14,18,8,0.0021219

2,4,14,19,1,0.0028742

2,4,14,19,2,-0.0010479

2,4,14,19,3,-0.0038569

2,4,14,19,4,-0.0012063

2,4,14,19,5,-0.0016685

2,4,14,19,6,-0.00092026

2,4,14,19,7,-0.00012741

2,4,14,19,8,0.0025084

2,4,14,20,1,0.0015861

2,4,14,20,2,0.00055391

2,4,14,20,3,-0.0021823

2,4,14,20,4,-0.0016766

2,4,14,20,5,-0.00058684

2,4,14,20,6,-0.00095921

2,4,14,20,7,7.829e-06

2,4,14,20,8,0.0029131

2,4,15,15,1,0.0015288

2,4,15,15,2,-9.4222e-05

2,4,15,15,3,-0.0020233

2,4,15,15,4,-0.0009672

2,4,15,15,5,-0.00070342

2,4,15,15,6,-0.00068078

2,4,15,15,7,-0.00033859

2,4,15,15,8,0.0017061

2,4,15,16,1,0.00062758

2,4,15,16,2,0.00030486

2,4,15,16,3,-0.0012206

2,4,15,16,4,-0.00060663

2,4,15,16,5,-0.00025569

2,4,15,16,6,-0.0013956

2,4,15,16,7,0.0008853

2,4,15,16,8,0.0015767

2,4,15,17,1,0.0031533

2,4,15,17,2,0.00081861

2,4,15,17,3,-0.003434

2,4,15,17,4,-0.0036533

2,4,15,17,5,-0.0018228

2,4,15,17,6,-0.0023108

2,4,15,17,7,-0.00073939

2,4,15,17,8,0.0023303

2,4,15,18,1,0.0012881

2,4,15,18,2,0.00074493

2,4,15,18,3,-0.001622

2,4,15,18,4,-0.0021894

2,4,15,18,5,-0.00089695

2,4,15,18,6,-0.0010411

2,4,15,18,7,0.00026028

2,4,15,18,8,0.0020113

2,4,15,19,1,0.00090758

2,4,15,19,2,-0.00095362

2,4,15,19,3,-0.0019434

2,4,15,19,4,-0.00053209

2,4,15,19,5,-0.00075766

2,4,15,19,6,-0.00099851

2,4,15,19,7,0.00036494

2,4,15,19,8,0.0020709

2,4,15,20,1,0.00053586

2,4,15,20,2,6.1146e-06

2,4,15,20,3,-0.00078149

2,4,15,20,4,-0.0011963

2,4,15,20,5,-0.0010978

2,4,15,20,6,-0.0012385

2,4,15,20,7,0.00094118

2,4,15,20,8,0.0026313

2,4,16,16,1,1.7612e-10

2,4,16,16,2,-0.0038256

2,4,16,16,3,-0.0014896

2,4,16,16,4,-0.0018314

2,4,16,16,5,0.00011762

2,4,16,16,6,-0.0016309

2,4,16,16,7,0.00011906

2,4,16,16,8,0.0003759

2,4,16,17,1,0.0026095

2,4,16,17,2,0.00023297

2,4,16,17,3,-0.0021265

2,4,16,17,4,-0.0011448

2,4,16,17,5,-0.00081441

2,4,16,17,6,-0.0012697

2,4,16,17,7,0.00065609

2,4,16,17,8,0.0018795

2,4,16,18,1,0.0010673

2,4,16,18,2,-0.0013155

2,4,16,18,3,-0.0021516

2,4,16,18,4,-0.00019024

2,4,16,18,5,-0.00085913

2,4,16,18,6,-0.00131

2,4,16,18,7,0.00063199

2,4,16,18,8,0.0017036

2,4,16,19,1,0.0008629

2,4,16,19,2,-0.00068473

2,4,16,19,3,-0.0017505

2,4,16,19,4,-0.00095183

2,4,16,19,5,-3.6767e-05

2,4,16,19,6,-0.0022217

2,4,16,19,7,0.0008837

2,4,16,19,8,0.00082155

2,4,16,20,1,0.00080928

2,4,16,20,2,0.0010126

2,4,16,20,3,-0.0020405

2,4,16,20,4,-5.86e-05

2,4,16,20,5,-0.00035913

2,4,16,20,6,-0.00063811

2,4,16,20,7,0.00025653

2,4,16,20,8,0.0018461

2,4,17,17,1,0.01088

2,4,17,17,2,0.00050771

2,4,17,17,3,-0.0015061

2,4,17,17,4,-0.0017278

2,4,17,17,5,-0.00099098

2,4,17,17,6,-0.0013418

2,4,17,17,7,0.00013031

2,4,17,17,8,0.001014

2,4,17,18,1,0.0054638

2,4,17,18,2,0.0017217

2,4,17,18,3,-0.003243

2,4,17,18,4,-0.0010373

2,4,17,18,5,-0.0015934

2,4,17,18,6,-0.0014333

2,4,17,18,7,0.00033754

2,4,17,18,8,0.0023674

2,4,17,19,1,0.0041318

2,4,17,19,2,0.00013881

2,4,17,19,3,-0.0030111

2,4,17,19,4,-0.0013017

2,4,17,19,5,-0.0015279

2,4,17,19,6,-0.0013963

2,4,17,19,7,0.00034514

2,4,17,19,8,0.002565

2,4,17,20,1,0.0018874

2,4,17,20,2,0.00027227

2,4,17,20,3,-0.002255

2,4,17,20,4,-0.0014757

2,4,17,20,5,-0.0011477

2,4,17,20,6,-0.001995

2,4,17,20,7,0.00041799

2,4,17,20,8,0.0016997

2,4,18,18,1,0.0027162

2,4,18,18,2,-0.00048798

2,4,18,18,3,-0.0015434

2,4,18,18,4,-0.00030671

2,4,18,18,5,-0.00054228

2,4,18,18,6,-0.00055224

2,4,18,18,7,-1.4173e-05

2,4,18,18,8,0.0017514

2,4,18,19,1,0.0027124

2,4,18,19,2,0.00047925

2,4,18,19,3,-0.0026514

2,4,18,19,4,-0.0014977

2,4,18,19,5,-0.00076265

2,4,18,19,6,-0.0022138

2,4,18,19,7,0.00016566

2,4,18,19,8,0.0021024

2,4,18,20,1,0.0008468

2,4,18,20,2,0.00078236

2,4,18,20,3,-0.0024199

2,4,18,20,4,-0.0011416

2,4,18,20,5,-0.00069094

2,4,18,20,6,-0.00089856

2,4,18,20,7,0.00057485

2,4,18,20,8,0.002324

2,4,19,19,1,0.0025026

2,4,19,19,2,-0.0008415

2,4,19,19,3,-0.0022192

2,4,19,19,4,-0.00096584

2,4,19,19,5,-0.00076451

2,4,19,19,6,-0.00047317

2,4,19,19,7,0.00015942

2,4,19,19,8,0.0013818

2,4,19,20,1,0.0011304

2,4,19,20,2,0.00030835

2,4,19,20,3,-0.0024784

2,4,19,20,4,-0.00051497

2,4,19,20,5,-0.00015243

2,4,19,20,6,-0.00069066

2,4,19,20,7,9.7228e-05

2,4,19,20,8,0.0016223

2,4,20,20,1,0.00086666

2,4,20,20,2,-0.00021527

2,4,20,20,3,-0.00023518

2,4,20,20,4,-0.00069149

2,4,20,20,5,-0.00034198

2,4,20,20,6,-0.00026629

2,4,20,20,7,-0.0001349

2,4,20,20,8,0.0008623
