# Supplementary material for: On the Importance of the Distance Measures Used to Train and Test Knowledge-Based Potentials for Proteins
Source: PLoS One. 2014 Nov 20;9(11):e109335. doi: 10.1371/journal.pone.0109335 (PMC4239004; doi:10.1371/journal.pone.0109335)
Supplement: Readme Force Field S1 — (RTF) [file pone.0109335.s004.rtf]

The file ”SupportingMaterialData” contains the estimated parameters for the two distance-based knowledge based potentials PPD and PPE. These have been saved in a sparse format with six columns. The first column indicates whether the model is PPD (1) or PPE (2). The second column refers to which distance measures we have used in the training of the pair potential, either RMSD (1), MT (2), GDT-TS (3) or Q (4). In the third and fourth column the type of amino acid is given: GLY (1), PRO (2) ASP (3) GLU (4) ALA (5) ASN (6) GLN (7) SER (8) THR (9) LYS (10) ARG (11) HIS (12) VAL (13) ILE (14) MET (15) CYS (16) LEU (17) PHE (18) TYR (19) TRP (20). Our pair potentials are spanned by uniform cubic b-splines with compact support between 1Å and 12Å and 8 degrees of freedom which we refer to by the number 1 to 8 in the fifth column. Finally, the parameter value can be found in the six column.
